# Tandem Pd-Catalyzed Cyclization/Coupling of Non-Terminal Acetylenic Activated Methylenes with (Hetero)Aryl Bromides †

**DOI:** 10.3390/molecules27030630

**Published:** 2022-01-19

**Authors:** Aleksandra Błocka, Wojciech Chaładaj

**Affiliations:** Institute of Organic Chemistry, Polish Academy of Sciences, Kasprzaka 44/52, 01-224 Warsaw, Poland; aleksandra.blocka@icho.edu.pl

**Keywords:** palladium, homogeneous catalysis, alkynes, cross-coupling, tandem reactions

## Abstract

We report a new method for a tandem Pd-catalyzed intramolecular addition of active methylene compounds to internal alkynes followed by coupling with aryl and heteroaryl bromides. Highly substituted vinylidenecyclopentanes were obtained with good yields, complete selectivity, and excellent functional group tolerance. A plausible mechanism, supported by DFT calculations, involves the oxidative addition of bromoarene to Pd(0), followed by cyclization and reductive elimination. The excellent regio- and stereoselectivity arises from the *5-exo-dig* intramolecular addition of the enol form of the substrate to alkyne activated by the π-acidic Pd(II) center, postulated as the rate-determining step.

## 1. Introduction

**Scheme 1:**
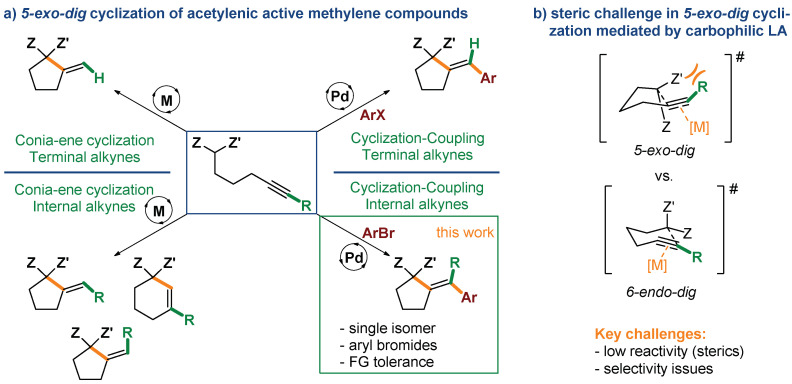
Strategies and challenges in methodologies involving *5-exo-dig* cyclization of acetylenic active methylene compounds.

Cyclization of alkynes and alkenes bearing a tethered nucleophilic group constitutes a direct and effective strategy for the construction of a range of carbo- and heterocyclic scaffolds [1,2,3,4,5,6,7,8,9,10]. Since the seminal work by Conia [11,12] on thermal cyclization of unsaturated carbonyl compounds, a range of catalytic methods towards carbocyclic motifs, featuring excellent atom economy and high efficiency under mild conditions were developed (Scheme 1a) [13]. One of the most effective of such strategies relies on the use of transition metal complexes exhibiting carbophilic Lewis acidic character (e.g., Au, Pd, Pt, Cu, Ag), capable of coordination to the C-C multiple bond, and in consequence, its activation for nucleophilic attack [14,15,16,17,18,19,20,21,22]. This approach offers another opportunity for harnessing the reactivity of the metal–vinyl intermediate for further functionalization via cross-coupling, generating higher molecular complexity in a single step [23,24,25,26,27,28]. In this regard, palladium complexes are catalysts of choice due to the combination of sufficient π-acidity with redox activity for driving both cyclization and cross-coupling [1,4,29].

This strategy was first realized by Balme in the early 1990s for acetylenic keto esters and malonates with simple aryl iodides [30,31,32]. More recently, employing a catalyst based on a bulky, electron-rich monophosphine, we have solved one of the major limitations of the reaction, disclosing mild and functional group-tolerant conditions suitable for considerably less reactive (hetero)aryl bromides and chlorides [33,34]. However, one of the remaining challenges of both *5-exo-dig* cycloisomerization and cyclization/coupling processes catalyzed by carbophilic Lewis acids is the low reactivity of substrates derived from internal alkynes. As the challenge arises from steric interactions of metal catalyst with a substituent at the alkyne terminus, shorter homologues cyclizing through a *5-endo-dig* manifold, and thus lacking such constrains, undergo facile cyclization [16,26,28,35]. Therefore, only few protocols for Conia-ene *5-exo-dig* cyclization of nonterminal acetylenes were reported to date [16,36,37,38]. Moreover, as observed for Au-catalyzed cycloisomerization of nonterminal acetylenic β-keto esters, competition between *5-exo-dig* and *6-endo-dig* cyclization manifolds could be expected, which ultimately could compromise the selectivity of the transformation (Scheme 1b) [16,37].

Herein, we present a protocol for effective Pd-catalyzed tandem *5-exo-dig* carbocyclization of nonterminal acetylenic active methylene compounds with subsequent coupling with aryl and heteroaryl bromides. The present method exhibits high efficiency, excellent functional group tolerance and selectivity towards a single isomer of the cyclization/coupling product.

## 2. Results and Discussion

First, in the quest for a method suitable for challenging substrates bearing an internal alkyene motif, a benchmark reaction of dimethyl hex-4-yn-1-ylmalonate **1** with bromobenzene was evaluated over a range of Pd-catalysts and reaction conditions (Table 1) [39]. Third generation Buchwald-type palladacyclic precatalysts were chosen as a platform for testing of phosphine ligands due to availability, moisture and oxygen insensitivity, and rapid and clean activation under mildly basic conditions [40]. Initial screening of palladium complexes based on various mono- and bi-dentate phosphines (See Table S1 in the Supporting Information for details) revealed that bulky, electron-rich monophosphines, catalysts of choice for analogous terminal acetylenic active methylene compounds, exhibit poor or at best moderate efficiency in the model reaction involving internal alkyne (Table 1, entries 1–3). For instance, employment of 2 mol% of Pd-complexes with XPhos or RuPhos at 60 °C provided only 31% and 0% of expected cyclization/coupling product **2**, respectively. It sharply contrasts the full conversion and ~90% yields observed for the reaction of dimethyl pent-4-yn-1-ylmalonate, a terminal analogue of **1**. Despite the moderate yield of the transformation, the product was isolated as a single isomer, constituting a promising starting point for further optimization. Increase of the temperature to 80 °C only slightly improved the efficiency of the transformation catalyzed by the Pd/XPhos system (Table 1, entries 1 and 2). Generally, bidentate phosphines exhibited even lower efficiency, except for diphenyl-2-pyridylphosphine (DPPPY), which performed best of all tested ligands. Further evaluation of the experimental variables delivered satisfactory conditions (See Tables S2–S6 in the Supporting Information for details) involving the use of potassium phosphate in DMF. Employment of less polar solvents, as well as strong organic bases, resulted in considerably worse results (entries 7–11).

**Scheme 2:**
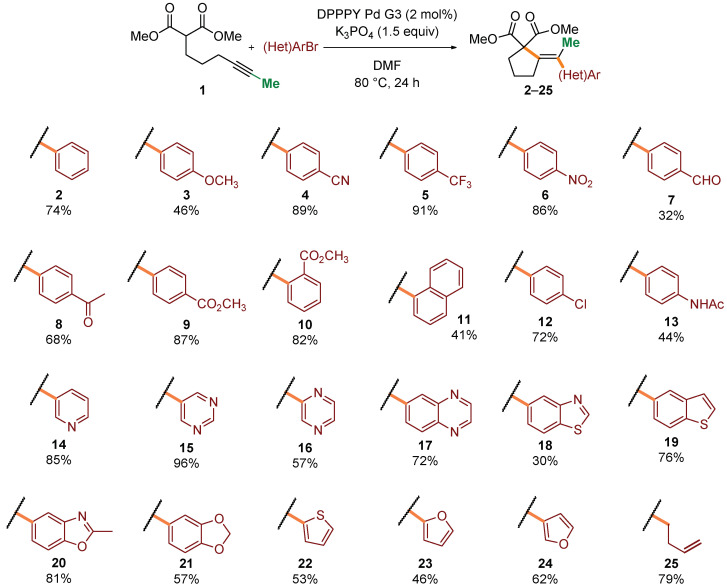
Scope in respect to aryl bromides. Standard conditions: Dimethyl 2-(hex-4-yn-1-yl)malonate (0.4 mmol), (hetero)aryl bromide (0.5 mmol, 1.25 equiv), DPPPY Pd G3 (5.1 mg, 8.0 μmol, 2 mol%), K_3_PO_4_ (127 mg, 0.6 mmol, 1.5 equiv), DMF (1.0 mL), 80 °C, 24 h. Isolated yields of pure products (after column chromatography) were reported.

Having established satisfactory conditions for a benchmark reaction, we proceeded to the investigation of the scope of the process. A range of structurally and electronically diverse aryl and heteroaryl bromides were initially tested in the reaction with malonate **1** (Scheme 2). Generally, electron-deficient coupling partners provided higher yields of desired products **4**–**10** (up to 91%). Steric hindrance caused by shift of a substituent to the *ortho* position seemed to have little influence on the overall efficiency of the process (**9** and **10**). Various functional groups, including amides, ketones, and aldehydes were well tolerated (**7**–**10**, **14**). Moreover, a range of heteroaryl bromides, including medicinally relevant N-heterocycles, were competent reaction partners, generally delivering products **14**–**24** in very good yields. Finally, vinyl bromide was also compatible with the reaction conditions, giving rise to diene **25** in 79% yield.

**Scheme 3:**
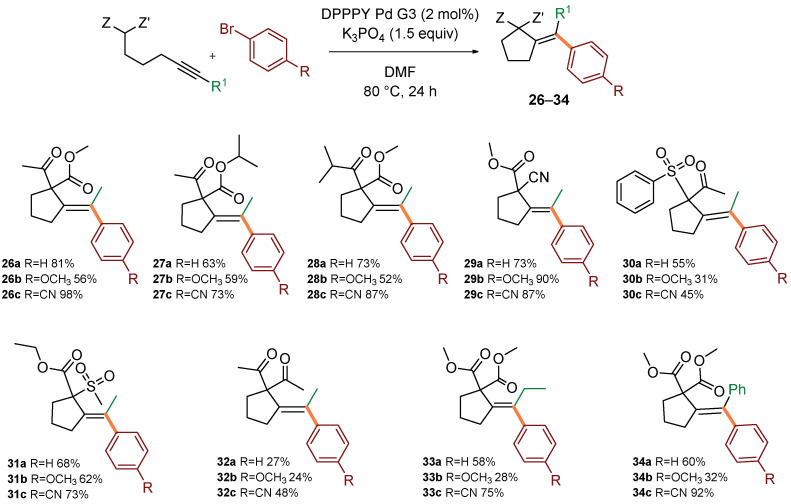
Scope in respect to acetylenic active methylenes. Standard conditions: acetylenic active methylene compound (0.4 mmol), (hetero)aryl bromide (0.5 mmol, 1.25 equiv), DPPPY Pd G3 (5.1 mg, 8.0 μmol, 2 mol%), K_3_PO_4_ (127 mg, 0.6 mmol, 1.5 equiv), DMF (1.0 mL), 80 ^°^C, 24 h. Isolated yields of pure products (after column chromatography) were reported.

Next, the scope in respect to various actetylenic active methylenes was investigated in reactions with three prototypical aryl bromides of various electronic properties: bromobenzene, *p*-methoxybromobenzene, and *p*-cyanobromobenzene (Scheme 3). Generally, β-keto esters (leading to **26**–**28**), cyanomalonates (leading to **29**), sulfones (leading to **30**–**31**) reacted with similar efficiency to model malonate **1**, contrary to β-diketones which reacted in moderate yields (leading to **32**). It could be speculated that low reactivity of dikentones arises from lower nucleophilicity of the enol form. In most cases, better yields were observed for the reaction with electron-deficient aryl bromides. Change of the substituent at the alkyne terminus to ethyl or phenyl was also tolerated, although slightly lower yields were observed (leading to **33**–**34**).

**Figure 1:**
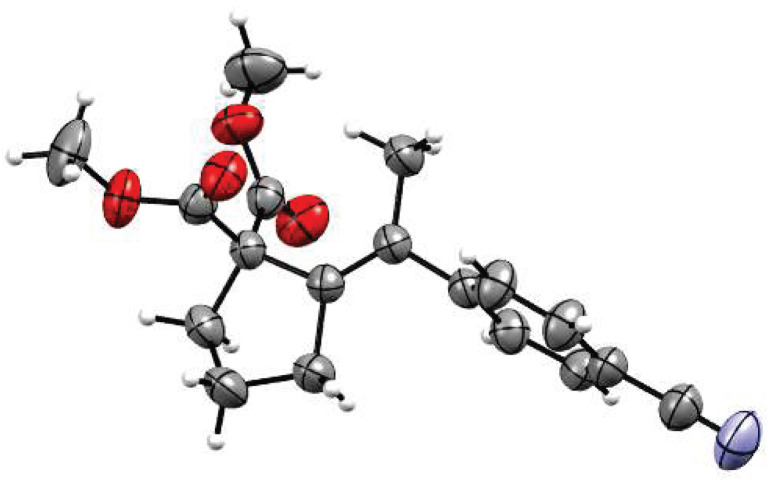
X-ray molecular structure of **4**.

As mentioned, all reactions proceeded with complete regio- and stereoselectivity, delivering products as single isomers. The structure was unambiguously confirmed by X-ray crystallographic analysis of a representative compound **4** (Figure 1). Mechanistically, the stereoselectivity of the transformation implies involvement of *anti* carbometalation of the alkyne. Such selectivity could be achieved only if the C-nucleophilic fragment of the molecule (e.g., enolate) attacks the alkyne motif activated through coordination of the carbophilic Pd(II) centre. Other scenarios involving either simultaneous coordination of enolate and alkyne moieties, or initial insertion of alkyne to aryl-Pd species would preferentially lead to *syn* dicarbofunctionalization products.

**Scheme 4:**
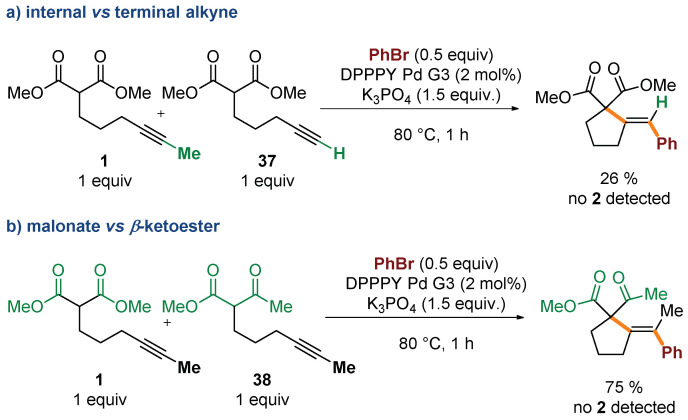
Competition experiments.

**Figure 2:**
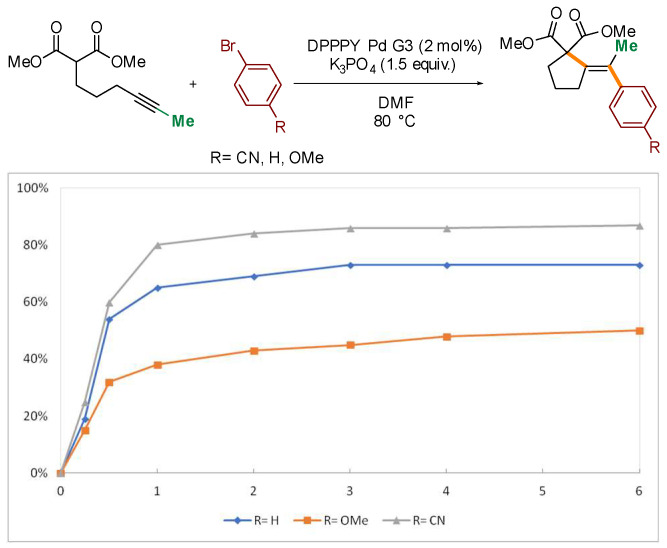
Kinetic profiles for the reaction of **1** with electronically varied bromoarenes.

Several control experiments were conducted to gain additional insight into the mechanism of the process. Competition experiments confirmed dramatic difference in reactivity of substrates based on internal and terminal alkynes (Scheme 4a). Reaction of bromobenzene with an equimolar mixture of **1** and its terminal analogue **37** delivered exclusively the product of cyclization/coupling of the latter, without even traces of **2** detected by GC. Similarly, no consumption of **1** was observed in competition experiments with the structurally related β-keto ester **38** (Scheme 4b). Such a huge difference in the reactivity of malonates and β-keto esters, differing in acidity by only ca. 1 pKa unit, ref. [41] could suggest that deprotonation of the starting material is not involved in the rate-determining step (*vide infra*). The impact of the electronic nature of the electrophilic partner on the reaction progress was also investigated through a comparison of the reactivity of **1** with three electronically distinct bromoarenes–bromobenzene, p-bromoanisole, and p-bromobenzonitrile. Higher rates were observed for more electron-deficient partners (Figure 2).

Density functional theory (DFT) calculations were conducted to elaborate the detailed reaction mechanism. All calculations were performed using Gaussian 16 package [42]. Structures of minima and transition states were optimized employing B3LYP and LANL2DZ basis sets for Pd, 6-31G(d) basis set for the other atoms, D3 version of Grimme’s empirical dispersion correction [43] and solvation (DMF) with SMD model [44]. Frequency analysis was performed at the same level to provide correction to thermodynamic functions and confirm the nature of optimized structures (minima and transition states featured zero or one imaginary frequency, respectively). Single point energies were calculated at M06 level of theory employing SDD basis set for Pd, 6-311++g(d,p) basis set for the other atoms and solvation (DMF) with SMD model [44]. Various conformers of intermediates and transition states were investigated and only the lowest energy conformers are shown in the work.

**Figure 3:**
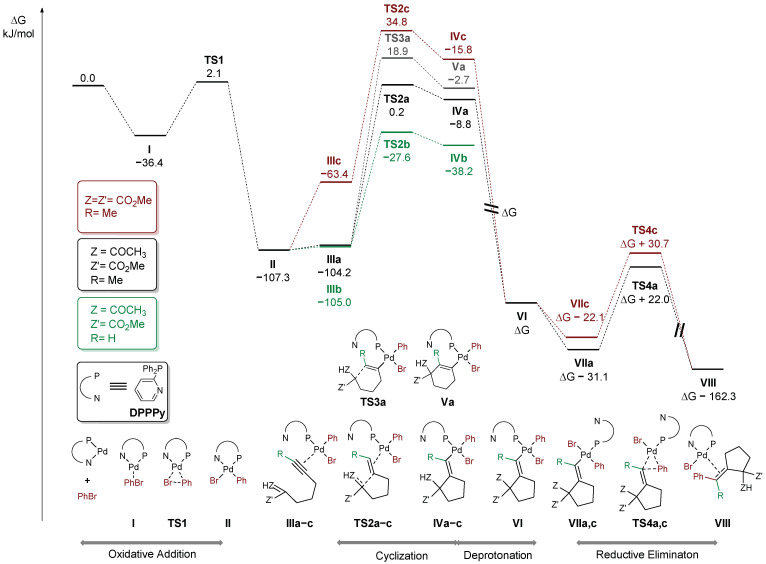
Gibbs free energy profile.

The free energy profile of the postulated pathway for model reaction of β-ketoester **38** with bromobenzene (black) is shown in Figure 3. Initially, bromobenzene undergoes facile oxidative addition to palladium(0) species (ΔG^‡^ = 38.5 kJ/mol), leading to Pd(II) intermediate **II**. Then, alkyne coordinates to a Pd(II) centre ultimately leading to a complex **III**, with enol form of the substrate coordinated in the conformation pre-organized for the cyclization, featuring ΔG^‡^ = 107.5 kJ/mol. Due to attack of the C-nucleophilic center of the enol from the opposite side of the alkyne in respect to the coordinated metal centre, the carbopalladation can proceed only in the *anti* fashion. The cyclization of the terminal analogue proceeds through a considerably lower barrier (ΔG^‡^ = 79.7 kJ/mol, green path). It is fully consistent with previously discussed competition experiments (Scheme 1a) and known examples of Conia-ene reactions catalyzed by carbophilic Lewis acids [37]. Interestingly, steric hindrance caused by the substituent at the alkyne moiety seems to have little if any effect on the coordination of the C-C triple bond to the Pd(II) species (cf. intermediates **IIIa** and **IIIb**). On the contrary, cyclization of the related malonate **1** (red path) is considerably more difficult than β-ketoester **38** (ΔG^‡^ = 142.1 vs. 107.5 kJ/mol) which is also in line with the competition experiment depicted in Scheme 1b. A significant fraction of the barrier arises from unfavorable enolization of the malonate, which is reflected in endergonic formation of intermediate **IIIc**. An alternative path involving deprotonation prior to the cyclization was also considered. However, the calculated barriers for the cyclization involving deprotonated malonate and keto ester fragments exhibited similar magnitudes (ΔG^‡^ = 41.5 and 40.4 kJ/mol, respectively). Taking into account the small difference in acidity (ca. 1 pKa unit at ambient temperature), formation of at least some amount of both products should be observed in the competition experiment (see Scheme 1b). Therefore, this path is less probable, at least if the reaction is carried out with a relatively weak, insoluble base (K_3_PO_4_). The other important factor associated with the cyclization step is the observed preference of *5-exo-dig* over *6-endo-dig* manifold which determines the excellent regioselectivity of the overall process. In fact, the calculated barrier for *6-endo-dig* carbocyclization (grey path) is higher by 18.7 kJ/mol than the observed *5-exo-dig* (black path). Furthermore, the vinyl–palladium intermediate **IVa**, bearing a protonated ketone moiety undergoes facile deprotonation to **VIa**. It then undergoes isomerization to **VIIa**, with vinyl and phenyl ligands in the *cis* position required for reductive elimination, which proceeds with ΔG^‡^ = 53.1 kJ/mol (through a transition state **TS4a**). Reductive elimination from the malonate analogue (red path) is similarly easy (ΔG^‡^ = 52.8 kJ/mol).

As previously mentioned, the reaction with electron-deficient bromoarenes proceeds with slightly higher rates (see Figure 2). Presumably, substituent at the arene moiety perturbs the Lewis acidity of the Pd(II) centre and thus exerts impact on the facility of the cyclization step, considered rate-determining. Free energies of activation for the cyclization step were calculated for a series of three electronically distinct arylpalladium species bearing CN, H, and OMe at the *para* position of the phenyl moiety (Table 1). The lowest barrier was found for the complex with the most electron-deficient arene. To complete the mechanistic picture of the overall transformation, barriers for reductive elimination were also calculated and are summarized in Table 2.

## 3. Conclusions

In conclusion, we developed a broadly applicable method for the carbocyclization-coupling of active methylene compounds bearing internal alkyne motif with aryl and heteroaryl bromides. The tandem Pd-catalyzed process exhibits excellent regio- and stereoselectivity, functional group tolerance, and high efficiency of the challenging *5-exo-dig* intramolecular addition to nonterminal alkynes activated by a carbophilic Lewis acid. The mechanistic investigations, involving computational studies, support a mechanism involving oxidative addition, cyclization, and reductive elimination. The *5-exo-dig* intramolecular nucleophilic addition of the enol form of the substrate to alkyne activated through coordination to the Pd(II) centre was identified as the rate- and configuration-determining step.

## 4. Experimental Section

**General procedure for Pd-catalyzed carbocyclization-coupling of aryl bromides with acetylenic active methylene compounds:** In a drybox, a 4 mL screw-cap vial was charged with DPPPY Pd G3 (5.10 mg, 8 mmol), aryl halide (0.5 mmol), K_3_PO_4_ (127.2 mg, 0.6 mmol), DMF (1 mL), and a magnetic stirring bar. Then, acetylenic active methylene compound (e.g., dimethyl 2-(hex-4-yn-1-yl)malonate) was added (0.4 mmol), the vial was tightly sealed and removed from drybox. The reaction mixture was stirred for 24 h at 80 °C in a heating block, then cooled to room temperature, quenched with 20 mL of a saturated NH_4_Cl solution, added to 10 mL of water, and extracted with MTBE (3 × 10 mL). The combined organic phases were dried with Na_2_SO_4_, filtered, and concentrated. The crude product was purified by column chromatography on silica gel.

**Dimethyl (E)-2-(1-phenylethylidene)cyclopentane-1,1-dicarboxylate** was prepared in a reaction of dimethyl 2-(hex-4-yn-1-yl)malonate and bromobenzene following a general procedure (98 mg, yield 85%). Product was isolated as an oil after column chromatography on silica gel (15g, hex/AcOEt 9:1). ^1^H NMR (400 MHz, CDCl_3_) δ 7.34–7.28 (m, 2H), 7.23–7.16 (m, 3H), 3.79 (s, 6H), 2.42 (t, *J* = 6.8 Hz, 2H), 2.29–2.22 (m, 2H), 1.96 (t, *J* = 2.0 Hz, 3H), 1.64 (p, *J* = 7.0 Hz, 2H);^13^C NMR (101 MHz, CDCl_3_) δ 172.0, 145.2, 135.8, 135.5, 128.1, 127.3, 126.3, 63.4, 52.5, 39.2, 33.5, 24.8, 22.3; IR(CH_2_Cl_2_): 2952, 2879, 2850, 1732, 1599, 1436, 1252, 1157, 1074, 988, 919, 764, 700, 429 cm^−1^; MS (EI): *m*/*z* (%) = 289(9), 288(43) [M^+^], 256(27), 230(24), 229(97), 228(59), 197(23), 170(24), 169(100), 168(27), 141(27), 128(16), 115(17), 105(7), 91(27), 77(10), 59(11); HRMS (EI): *m*/*z* calcd for C_17_H_20_O_4_ 288.1362; found 288.1364.

## Figures and Tables

**Table 1:**
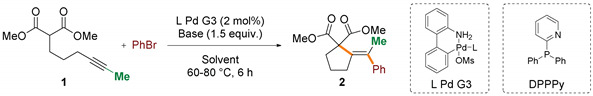
Evaluation of the reaction conditions ^1^.

Entry	Catalyst	Solvent, Base	Temp	Yield ^2^
1	XPhos Pd G3	DMF, K_3_PO_4_	60 °C	31%
2	XPhos Pd G3	DMF, K_3_PO_4_	80 °C	41%
3	RuPhos Pd G3	DMF, K_3_PO_4_	60 °C	0 %
4	BINAP Pd G3	DMF, K_3_PO_4_	60 °C	38%
**5**	DPPE Pd G3	DMF, K_3_PO_4_	60 °C	16%
6	DPPPY Pd G3	DMF, K_3_PO_4_	60 °C	77%
**7**	**DPPPY Pd G3**	**DMF, K_3_PO_4_**	**80 °C**	**89%**
8	DPPPY Pd G3	DMF, MeOK	80 °C	0%
9	DPPPY Pd G3	DMF, KHMDS	80 °C	28%
10	DPPPY Pd G3	DMF, NaOH	80 °C	40%
11	DPPPY Pd G3	THF, K_3_PO_4_	80 °C	20%
11	DPPPY Pd G3	Toluene, K_3_PO_4_	80 °C	0%

^1^ Conditions: L Pd G3 (2 mol%), dimethyl 2-(hex-4-yn-1-yl)malonate (0.100 mmol, 1 equiv.), PhBr (0.150 mmol, 1.5 equiv.), K_3_PO_4_ (0.150 mmol, 1.5 equiv.), DMF (0.5 mL), 60–80 °C, 6 h. ^2^ Determined by GC with mesitylene as an internal standard. Optimal conditions in bold.

**Table 2:**
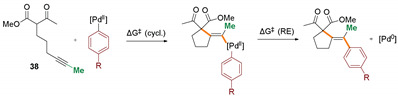
Free energy of activation for cyclization and reductive elimination steps in the reaction of **38** with aryl bromides.

Entry	R	ΔG^‡^ (cycl.)	ΔG^‡^ (RE)
1	H	107.5 kJ/mol	53.1 kJ/mol
2	CN	101.3 kJ/mol	38.8 kJ/mol
3	OMe	107.9 kJ/mol	50.6 kJ/mol

## Supplementary Materials

The following supporting information can be downloaded. Details of optimization and control experiments, experimental procedures and characterization of all compounds, crystallographic data, details of DFT calculations (atom coordinates, energies and corrections to thermodynamic functions for calculated structures).
