# Supplementary material for: Tandem Pd-Catalyzed Cyclization/Coupling of Non-Terminal Acetylenic Activated Methylenes with (Hetero)Aryl Bromides
Source: Molecules. 2022 Jan 19;27(3):630. doi: 10.3390/molecules27030630 (PMC8839394; doi:10.3390/molecules27030630)
Supplement: Supplementary file 1 [file molecules-27-00630-s001.zip › molecules-1546419-supplementary.pdf]

Supporting Information

Tandem Pd-catalyzed cyclization/coupling of non-terminal acetylenic activated methylenes with (hetero)aryl bromides.

Aleksandra Błocka, Wojciech Chaładaj*

Institute of Organic Chemistry Polish Academy of Sciences
Kasprzaka 44/52, 01-224 Warsaw, Poland

e-mail: wojciech.chaladaj@icho.edu.pl

Table of contents

Table of contents 2
General Information 3
Evaluation of reaction conditions for Pd-catalyzed cyclization/coupling of dimethyl 2-(hex-4-yn-1-yl)malonate with bromobenzene. 4
General procedure for the substrate preparation 7
General procedure for Pd-catalyzed carbocyclization/coupling of nonterminal acetylenic active methylene compounds with aryl bromides 8
Kinetic profiles for the reaction of 1 with electronically varied bromoarenes. 23
X-Ray Diffraction Data of Compound 4 24
NMR signal assignment for compound 2 25
Computational methods 26
Copies of ^{1}H and ^{13}C{^{1}H} NMR spectra of isolated compounds 71
References 127

General Information

All the manipulations were performed in a nitrogen-filled glovebox or under an argon atmosphere using Schlenk techniques, unless mentioned otherwise. Flash chromatography was performed using Merck silica gel 60 (230-400 mesh). TLC analysis of reaction mixtures was performed on Merck silica gel 60 F254 TLC plates and visualized with cerium molybdate stain (Hanessian's stain). ^{1}H, $^{13}C\{1H\}$, and ^{19}F NMR spectra were recorded with a Bruker AV 400 spectrometer. ^{1}H and ^{13}C chemical shifts are given in ppm relative to TMS. The solvent signals were used as references ($CDCl_3$ δ_H = 7.26 ppm, δ_C = 77.0 ppm) and the chemical shift converted to the TMS scale. Coupling constants (J) are reported in Hz, and the following abbreviations were used to denote multiplets: s = singlet, d = doublet, t = triplet, q = quartet, quint = quintet, m = multiplet (denotes complex pattern), dd = doublet of doublets, dt = doublet of triplets and br = broad signal. Infrared spectra were recorded with a Jasco FTIR-6200 spectometer. Electron ionization high-resolution mass spectra (EI-HR) were recorded with an Autospec Premier (Waters Inc) mass spectrometer using the narrow-range high-voltage scan technique with low-boiling perfluorokerosene (PFK) as internal standard. Samples were introduced by using a heated direct insertion probe. Electrospray ionization high-resolution mass spectra (ESI-HR) were recorded with MALDISynapt G2-S HDMS (Waters Inc) mass spectrometer equipped with an electrospray ion source and q-TOF type mass analyzer. ESI-MS spectra were recorded in the positive ion mode (the source parameters: capillary voltage 3.15 kV, sampling cone 25 V, source temperature 120 °C, desolvation temperature 150 °C). GC analyses were performed on TRACE™ Ultra Gas Chromatograph (Thermo Scientific) equipped with FID detector and Restek Rtx-1 column (30m, 0.32 mmID, 0.5 μm df). The following temperature program was used: 100 °C (2 min), 20 °C/min to 310 °C (2 min). Unless otherwise noted, all commercially available compounds (ABCR, Acros, Fluorochem, TCI, Sigma-Aldrich, Strem) were used as received. Phosphine ligands were purchased from Aldrich, $Pd(OAc)_2$ was purchased Strem. Precatalyst DPPPY Pd G3 was prepared following Buchwald's procedure,[1] and showed similar reactivity to the commercial sample purchased from Strem.

Evaluation of reaction conditions for Pd-catalyzed cyclization/coupling of dimethyl 2-(hex-4-yn-1-yl)malonate with bromobenzene.

General procedure for evaluation of reaction conditions: In a glovebox, to a 4-mL screw-capped vial containing catalyst (typically 2 mol%) following reagents were added: dimethyl 2-(hex-4-yn-1-yl)malonate (21.2 mg, 0.100 mmol), bromobenzene (23.6 mg, 0.150 mmol), base (0.150 mmol) and solvent (0.5 ml). Then, magnetic stirring bar was placed and the vial was sealed with a cap containing a PTFE septum. The reaction mixture was stirred at 60 °C for 6 h and then cooled to room temperature. The mixture was diluted with MTBE (2 mL) quenched with sat. aqueous NH_4Cl (0.5 mL) and mesitylene (25 µl) was added as a internal standard.

Table S1. Effect of catalyst[a]

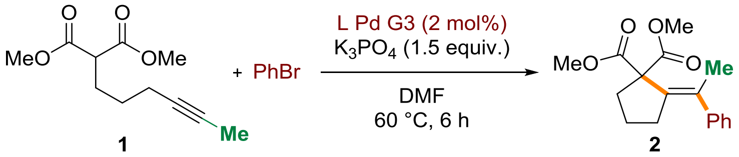

Entry	Catalyst	Yield[b]
1	XPhos PdG3	31%
2	XPhos PdG3	41%[c]
3	RuPhos PdG3	0%
4	SPhos PdG3	0%
5	CyJohnPhos PdG3	30%
6	DavePhos PdG3	0%
7	BrettPhos PdG3	24%
8	t-BuXPhos PdG3	0%
9	PCy_3 PdG3	8%
10	PPh_3 PdG3	67%
11	$P(o\text{-}tol)_3$ PdG3	41%
**12**	**DPPPy PdG3**	**77%**
**13**	**DPPPy PdG3**	**89%**
12	MonoPhos PdG3	13%
13	DPPE PdG3	16%
14	DPPF PdG3	11%
15	DPPB PdG3	29%
16	BINAP PdG3	38%

[a]Conditions: L Pd G3 (2 mol%), dimethyl 2-(hex-4-yn-1-yl)malonate **1** (0.100 mmol, 1 equiv.), bromobenzene (0.150 mmol, 1.5 equiv.), K_3PO_4 (0.150 mmol, 1.5 equiv.), DMF (0.5 mL), 60°C, 6h. [b] Yield was determined by GC with mesitylene as an internal standard.

Table S2. Effect of solvent[a]

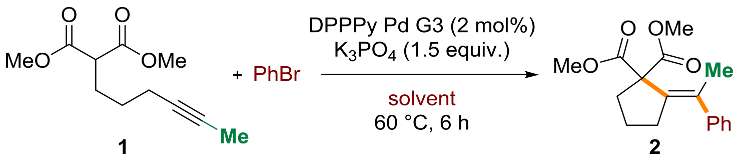

Entry	Solvent	Yield[b]
1	MeCN	56%
2	DMSO	61%
3	MeOH	17%
4	THF	20%
5	Toluene	0%
6	Dioxane	0%
7	DCE	0%
8	Acetone	45%
9	DMF	77%

[a] Conditions: DPPPy Pd G3 (2 mol%), dimethyl 2-(hex-4-yn-1-yl)malonate **1** (0.100 mmol, 1 equiv.), bromobenzene (0.150 mmol, 1.5 equiv.), K_3PO_4 (0.150 mmol, 1.5 equiv.), solvent (0.5 mL), 60°C, 6h. [b] Yield was determined by GC with mesitylene as an internal standard.

Table S3. Effect of base[a]

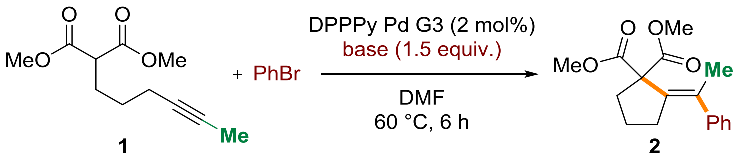

Entry	Base	Yield[b]
1	*t*-BuOK	66%
2	*t*-BuOLi	23%
3	*t*-BuONa	51%
4	LiHMDS	51%
5	KHMDS	28%
6	MeOK	0%
7	AcONa	0%
8	Ba_2CO_3	0%
9	KOH	34%
10	NaOH	40%
11	Cs_2CO_3	57%
12	AcOCs	14%
13	K_3PO_4	64%
14	K_2CO_3	23%

[a]Conditions DPPPy Pd G3 (2 mol%), dimethyl 2-(hex-4-yn-1-yl)malonate **1** (0.100 mmol, 1 equiv.), bromobenzene (0.150 mmol, 1.5 equiv.), base (0.150 mmol, 1.5 equiv.), DMF (0.5 mL), 60°C, 6h. [b] Yield was determined by GC with mesitylene as an internal standard.

Table S4. Effect of stoichometry of reagents[a]

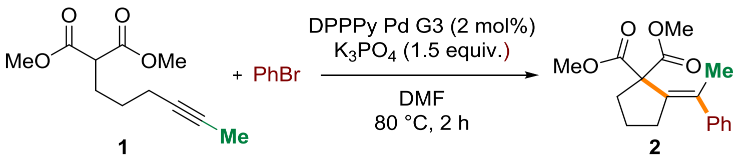

Entry	Amount of malonate	Amout of PhBr	Amount of K_3PO_4	Yield[b]
1	0.5eq	1.5eq	1.5eq	49%
2	1eq	1.5eq	1.5eq	78%
3	2eq	1.5eq	1.5eq	53%
4	1eq	0.75eq	1.5eq	43%
5	1eq	3eq	1.5eq	75%
6	1eq	1.5eq	0.75eq	46%
7	1eq	1.5eq	3eq	78%

[a]Conditions: DPPPy Pd G3 (2 mol%), DMF (0.5 mL), 80 °C, 2h. [b] Yield was determined by GC with mesitylene as an internal standard.

Table S5. Effect of catalyst loading[a]

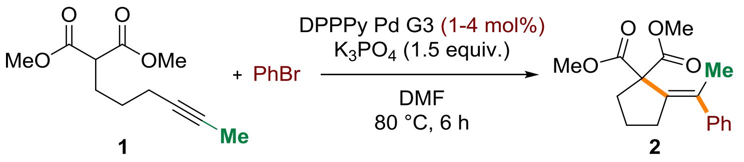

Entry	Catalyst loading	Yield[b]
1	1 mol%	36%
2	2 mol%	43%
3	4 mol%	42%

[a]Conditions: DPPPy Pd G3 (2 mol%), dimethyl 2-(hex-4-yn-1-yl)malonate **1** (0.100 mmol, 1 equiv.), bromobenzene (0.150 mmol, 1.5 equiv.), K_3PO_4 (0.150 mmol, 1.5 equiv.), DMF (0.5 mL), 80°C, 6h. [b] Yield was determined by GC with mesitylene as an internal standard.

Table S6. Effect of base[a]

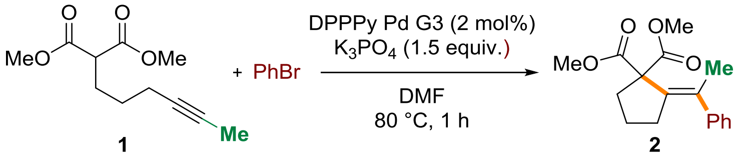

Entry	Amount of solvent	Yield[b]
1	0.25 ml	36%
2	0.5 ml	43%
3	1 ml	37%

[a]Conditions: DPPPy Pd G3 (2 mol%), dimethyl 2-(hex-4-yn-1-yl)malonate **1** (0.100 mmol, 1 equiv.), bromobenzene (0.150 mmol, 1.5 equiv.), K_3PO_4 (0.150 mmol, 1.5 equiv.), DMF (0.25-1 mL), 80°C, 1h. [b]Yield was determined by GC with mesitylene as an internal standard.

General procedure for the substrate preparation

To a suspension of sodium hydride (15 mmol, 1.5 equiv., 60% in mineral oil) in dry DMF (15 ml), malonate ester (cyanoacetate or malononitrile) (12 mmol, 1.2 equiv.) was added dropwise at 0°C. Mixture was stirred for 30 minutes, next 6-iodohex-2-yne was added dropwise (10 mmol, 1.0 equiv.). Mixture was heated at 60 °C for 16 hours, then cooled down and quenched with diethyl ether/water/NH_4Cl mixture. Aqueous layer was washed with diethyl ether (3x30 ml), then combined organic phases was dried over sodium sulfate. Product was isolated as colorless oil after column chromatography. The isolated product was further purified by distillation under reduced pressure.

Methyl 2-cyanooct-6-ynoate (39) prepared in reaction of methyl 2-cyanoacetate following general procedure (870 mg, 4.85 mmol, yield 49%). ^{1}H NMR (400 MHz, $CDCl_3$) δ 3.75 (d, *J* = 1.5 Hz, 2H), 3.53 (dd, *J* = 8.2, 6.0 Hz, 1H), 2.18 – 2.10 (m, 2H), 2.05 – 1.90 (m, 2H), 1.71 – 1.66 (m, 3H), 1.64 – 1.53 (m, 2H).^{13}C NMR (101 MHz, $CDCl_3$) δ 166.2, 116.0, 76.6, 76.4, 53.0, 36.6, 28.6, 25.6, 17.6, 3.0.

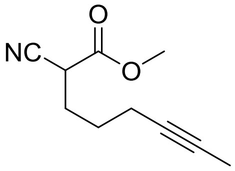

Methyl 2-isobutyryloct-6-ynoate (40) prepared in reaction of methyl 4-methyl-3-oxopentanoate following general procedure (691 mg, 3.10 mmol, yield 26%). ^{1}H NMR (400 MHz, $CDCl_3$) δ 3.66 – 3.61 (m, 3H), 3.61 – 3.55 (m, 1H), 2.77 – 2.66 (m, 1H), 2.10 – 2.02 (m, 2H), 1.89 – 1.79 (m, 2H), 1.70 – 1.63 (m, 3H), 1.42 – 1.31 (m, 2H), 1.06 – 0.96 (m, 6H).^{13}C NMR (101 MHz, $CDCl_3$) δ 208.6, 170.0, 76.7, 76.0, 56.2, 52.1, 40.3, 27.4, 26.7, 18.34, 18.1, 17.9, 3.2.

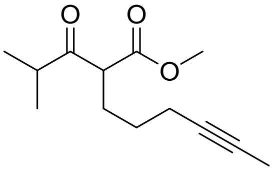

Isopropyl 2-acetyloct-6-ynoate (41) prepared in reaction of isopropyl 3-oxobutanoate following general procedure (2.10 g, 9.38 mmol, yield 78%). ^{1}H NMR (400 MHz, , $CDCl_3$) δ 5.04 – 4.94 (m, 1H), 3.36 – 3.28 (m, 1H), 2.20 – 2.12 (m, 3H), 2.12 – 2.03 (m, 2H), 1.91 – 1.81 (m, 2H), 1.73 – 1.66 (m, 3H), 1.47 – 1.34 (m, 2H), 1.23 – 1.15 (m, 6H).^{13}C NMR (101 MHz, $CDCl_3$) δ 202.8, 169.1, 78.0, 76.0, 68.7, 59.5, 28.5, 27.1, 26.5, 21.5, 21.4, 18.3, 3.2.

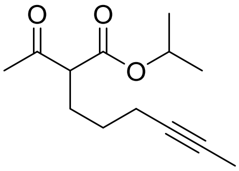

3-(phenylsulfonyl)non-7-yn-2-one (42) prepared in reaction of 1-(phenylsulfonyl) propan-2-one (3.96g, 20.00 mmol) with 6-iodohex-2-yne (3.75 g, 18.00 mmol), K_2CO_3 (2eq, 4.96g) in dry acetone 100 ml (3.06 g, 10.99 mmol, yield 61 %).^{1}H NMR (400 MHz, $CDCl_3$) δ 7.78 – 7.72 (m, 2H), 7.64 – 7.58 (m, 1H), 7.53 – 7.47 (m, 2H), 4.13 – 4.07 (m, 1H), 2.34 (s, 3H), 2.02 (tq, *J* = 7.0, 2.6 Hz, 2H), 1.98 – 1.90 (m, 2H), 1.64 (t, *J* = 2.6 Hz, 3H), 1.37 – 1.28 (m, 2H). ^{13}C NMR (101 MHz, $CDCl_3$) δ 199.8, 136.6, 134.1, 128.9, 77.3, 76.5, 75.1, 31.1, 25.8, 25.7, 18.1, 3.1.

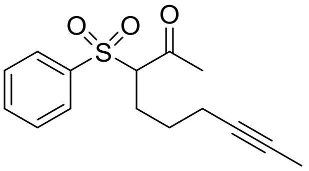

Ethyl 2-(methylsulfonyl)oct-6-ynoate (43) prepared in reaction of ethyl 2-(methylsulfonyl) acetate following general procedure (2.40 g, 9.76 mmol, yield 54%). ^{1}H NMR (400 MHz, $CDCl_3$) δ 4.34 – 4.18 (m, 2H), 3.75 (dd, *J* = 11.1, 3.9 Hz, 1H), 2.98 (d, *J* = 0.8 Hz, 3H), 2.25 – 2.02 (m, 4H), 1.73 (t, *J* = 2.6 Hz, 3H), 1.60 – 1.48 (m, 2H), 1.30 (t, *J* = 7.1 Hz, 3H).^{13}C NMR (101 MHz, $CDCl_3$) δ 166.5, 77.3, 76.8, 69.2, 62.5, 38.3, 26.2, 26.2, 18.2, 13.9, 3.3.

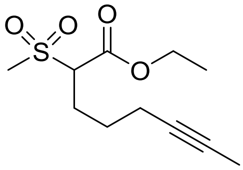

General procedure for Pd-catalyzed carbocyclization/coupling of nonterminal acetylenic active methylene compounds with aryl bromides

In a drybox, a 4 ml screw-cap vial was charged with DPPPY Pd G3 (5.10 mg, 8 mmol), (hetero)aryl halide (0.5 mmol), K_3PO_4 (127.2 mg, 0.6 mmol), DMF (1 ml), and a magnetic stirring bar. Then, acetylenic active methylene compound (e.g. dimethyl 2-(hex-4-yn-1-yl)malonate) was added (0.4 mmol), the vial was tightly sealed and removed from drybox. The reaction mixture was stirred for 24 h at 80 °C in a heating block, then cooled to room temperature, quenched with 20 ml of an NH_4Cl solution, added to 10 ml of water, and extracted with MTBE (3 x 10 ml). The combined organic phases were dried with Na_2SO_4, filtered, and concentrated. The crude product was purified by column chromatography on silica gel.

Scope and characterization of new compounds:

Dimethyl *(E)*-2-(1-phenylethylidene)cyclopentane-1,1-dicarboxylate (2). Prepared in reaction of dimethyl 2-(hex-4-yn-1-yl)malonate (84.9 mg, 0.500 mmol) and bromobenzene (78.5 mg, 0.500 mmol) under general procedure (98.1 mg, 0.340 mmol, yield 85%). Product was isolated as oil after column chromatography on silica gel (15g, hex/AcOEt 9:1). ^{1}H NMR (400 MHz, $CDCl_3$) δ 7.34 – 7.28 (m, 2H), 7.23 – 7.16 (m, 3H), 3.79 (s, 6H), 2.42 (t, J = 6.8 Hz, 2H), 2.29 – 2.22 (m, 2H), 1.96 (t, J = 2.0 Hz, 3H), 1.64 (p, J = 7.0 Hz, 2H). ^{13}C NMR (101 MHz, $CDCl_3$) δ 172.0, 145.2, 135.8, 135.5, 128.1, 127.3, 126.3, 63.4, 52.5, 39.2, 33.5, 24.8, 22.3. IR(CH_2Cl_2): 2952, 2879, 2850, 1732, 1599, 1436, 1252, 1157, 1074, 988, 919, 764, 700, 429 cm^{-1}; MS (EI): m/z (%) = 289(9), 288(43) [M$^+$], 256(27), 230(24), 229(97), 228(59), 197(23), 170(24), 169(100), 168(27), 141(27), 128(16), 115(17), 105(7), 91(27), 77(10), 59(11); HRMS (EI): m/z calcd for $C_{17}H_{20}O_4$ 288.1362; found 288.1364.

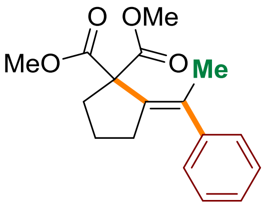

Dimethyl *(E)*-2-(1-(4-methoxyphenyl)ethylidene)cyclopentane-1,1-dicarboxylate (3). Prepared in reaction of dimethyl 2-(hex-4-yn-1-yl)malonate (84.9 mg, 0.400 mmol) and 4-bromoanisole (93.6 mg, 0.500 mmol) under general procedure (94.3 mg, 0.296 mmol, yield 74%). Product was isolated as oil after column chromatography on silica gel (15g, hex/AcOEt 8:2). ^{1}H NMR (400 MHz, $CDCl_3$) δ 7.14 – 7.09 (m, 2H), 6.87 – 6.82 (m, 2H), 3.80 (s, 3H), 3.78 (s, 6H), 2.40 (t, J = 6.8 Hz, 2H), 2.31 – 2.25 (m, 2H), 1.93 (t, J = 2.0 Hz, 3H), 1.64 (p, J = 7.0 Hz, 2H). ^{13}C NMR (101 MHz, $CDCl_3$) δ 172.1, 158.0, 137.6, 135.7, 135.0, 128.5, 113.5, 63.4, 55.2, 52.5, 39.3, 33.7, 24.8, 22.4. IR(CH_2Cl_2): 3463, 2841, 2050, 1729, 1607, 1512, 1573, 1436, 1376, 1247, 1157, 1121, 1068, 1036, 987, 869, 834, 715, 640, 600, 521 cm^{-1}. MS (EI): m/z (%) = 319(9), 318(34), 260(29), 259(100), 258(13), 227(16), 200(10), 199(37), 184(9), 171(8), 121(10), 115(6), 91(6), 59(8). HRMS (EI): m/z calcd for $C_{18}H_{22}O_5$ 318.1467; found 318.1475.

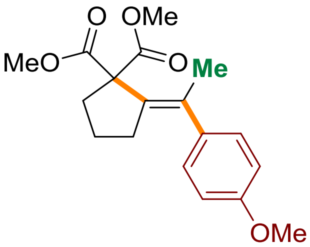

Dimethyl *(E)*-2-(1-(4-cyanophenyl)ethylidene)cyclopentane-1,1-dicarboxylate (4). Prepared in reaction of dimethyl 2-(hex-4-yn-1-yl)malonate (84.9 mg, 0.400 mmol) and 4-bromobenzonitrile (91.0 mg, 0.500 mmol) under general procedure (111.5 mg, 0.367 mmol, yield 89%). Product was isolated as oil after column chromatography on silica gel (15g, hex/AcOEt 9:1→8:2). ^{1}H NMR (400 MHz, $CDCl_3$) δ 7.61 – 7.56 (m, 2H), 7.27 (dd, J = 8.1, 1.5 Hz, 2H), 3.77 (s, 6H), 2.39 (t, J = 6.8 Hz, 2H), 2.18 (ddt, J = 7.2, 5.1, 2.0 Hz, 2H), 1.91 (t, J = 2.0 Hz, 3H), 1.63 (p, J = 7.0 Hz, 2H); ^{13}C NMR (101 MHz, $CDCl_3$) δ 171.5, 149.9, 137.5, 133.9, 132.1, 128.2, 118.8, 110.2, 63.5, 52.6, 39.0, 33.5, 24.7, 21.8; IR(CH_2Cl_2): 3640, 3463, 2953, 2880, 2852, 2227, 1731, 1605, 1503, 1435, 1405, 1255, 1159, 1070, 989, 928, 842, 553 cm^{-1}. MS (EI): m/z (%) = 314(11), 313(44), 281(21), 254(80), 253(98), 238(9), 222(21), 221(15), 195(29), 194(100), 193(26), 180(20), 166(31), 153(14),

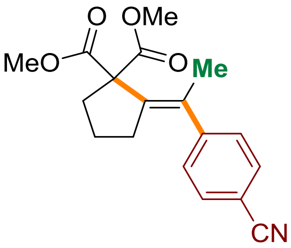

127(9), 116(20), 91(7), 77(6), 59(28). HRMS (EI): m/z calcd for $C_{18}H_{19}NO_4$ 313.1314; found 313.1320.

Dimethyl *(E)*-2-(1-(4-(trifluoromethyl)phenyl)ethylidene)cyclopentane-1,1-dicarboxylate (5).

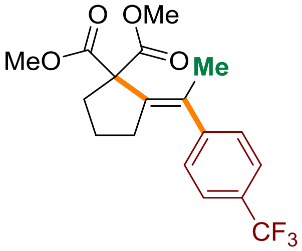

Prepared in reaction of dimethyl 2-(hex-4-yn-1-yl)malonate (84.9 mg, 0.400 mmol) and 4-bromobenzotrifluoride (113.0 mg, 0.502 mmol) under general procedure (130.1 mg, 0.365 mmol, yield 91%). Product was isolated as oil after column chromatography on silica gel (15g, hex/AcOEt 95:5→8:2). ^{1}H NMR (400 MHz, $CDCl_3$) δ 7.60 – 7.54 (m, 2H), 7.32 – 7.27 (m, 2H), 3.79 (s, 6H), 2.42 (t, *J* = 6.8 Hz, 2H), 2.24 – 2.18 (m, 2H), 1.95 (t, *J* = 2.0 Hz, 3H), 1.65 (p, *J* = 7.1 Hz, 3H).^{13}C NMR (101 MHz, $CDCl_3$) δ 171.8, 148.9, 137.1, 134.4, 128.8, 128.4, 127.8, 125.6, 125.2, 125.2, 125.2, 63.5, 52.6, 39.2, 33.6, 24.8, 22.1. IR(CH_2Cl_2): 2954, 1733, 1616, 1436, 1407, 1327, 1255, 1163, 1124, 1068, 1017, 990, 843, 785, 608 cm^{-1}. MS (EI): m/z (%) = 357(12), 356(52), 337(17), 298(29), 297(97), 296(100), 281(7), 265(19), 238(23), 237(90), 227(59), 209(24), 197(24), 183(14), 167(26), 159(23), 153(16), 128(7), 91(9), 77(4), 59(33), 41(9). HRMS (EI): m/z calcd for $C_{18}H_{19}O_4F$ 356.1235; found 356.1237.

Dimethyl *(E)*-2-(1-(4-nitrophenyl)ethylidene)cyclopentane-1,1-dicarboxylate (6). Prepared in

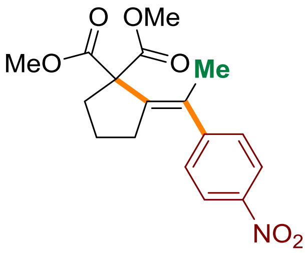

reaction of dimethyl 2-(hex-4-yn-1-yl)malonate (84.9 mg, 0.400 mmol) and 4-bromonitrobenzene (101.1 mg, 0.500 mmol) under general procedure (115.7 mg, 0.347 mmol, yield 86%). Product was isolated as yellow solid after column chromatography on silica gel (15g, hex/AcOEt 8:2). ^{1}H NMR (400 MHz, $CDCl_3$) δ 8.18 – 8.13 (m, 2H), 7.37 – 7.31 (m, 2H), 3.78 (s, 6H), 2.41 (t, *J* = 6.8 Hz, 2H), 2.20 (ddt, *J* = 7.1, 5.0, 2.0 Hz, 2H), 1.94 (t, *J* = 2.0 Hz, 3H), 1.65 (p, *J* = 7.1 Hz, 2H);^{13}C NMR (101 MHz, $CDCl_3$) δ 171.48, 151.97, 146.41, 137.87, 133.69, 128.39, 123.61, 77.32, 77.00, 76.68, 63.57, 52.69, 39.05, 33.60, 24.73, 21.82; IR (CH_2Cl_2): 2952, 2878, 2853, 1729, 1637, 1596, 1519, 1435, 1347, 1257, 1156, 927, 850, 754, 707, 511, 465 cm^{-1}; MS (EI): m/z (%) = 334(9), 333(36)[M^+], 301(24), 275(22), 274(78), 273(100), 256(34), 227(16), 226(21), 214(52), 213(17), 197(10), 169(22), 168(45), 167(39), 153(33), 152(29), 128(17), 115(18), 91(9), 77(7), 59(34), 41(5); HRMS (EI): m/z calcd for $C_{17}H_{19}NO_2$ 333.1212; found 333.1213.

Dimethyl *(E)*-2-(1-(4-formylphenyl)ethylidene)cyclopentane-1,1-dicarboxylate (7). Prepared in

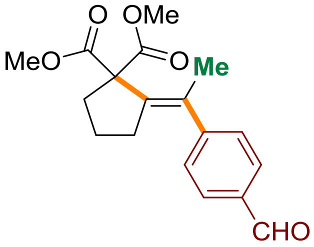

reaction of dimethyl 2-(hex-4-yn-1-yl)malonate (84.9 mg, 0.400 mmol) and 4-bromobenzaldehyde (93.0 mg, 0.503 mmol) under general procedure (40.2 mg, 0.127 mmol, yield 32%). Product was isolated as oil after column chromatography on silica gel (15g, hex/AcOEt 9:1). ^{1}H NMR (400 MHz, $CDCl_3$) δ 9.96 (s, 1H), 7.86 – 7.79 (m, 2H), 7.36 – 7.30 (m, 2H), 3.77 (s, 6H), 2.40 (t, *J* = 6.7 Hz, 2H), 2.25 – 2.16 (m, 2H), 1.94 (t, *J* = 2.0 Hz, 3H), 1.63 (p, *J* = 7.0 Hz, 2H); ^{13}C NMR (101 MHz, $CDCl_3$) δ 191.8, 171.7, 151.7, 137.2, 134.8, 134.5, 129.8, 128.2, 63.6, 52.7, 39.1, 33.6, 24.8, 21.9; IR (CH_2Cl_2): 2952, 2878, 2849, 2735, 1731, 1701, 1604, 1566, 1435, 1389, 1254, 1159, 1070, 989, 833, 557, 522 cm^{-1}; MS (EI): m/z (%) = 317(12), 316(44)[M^+], 285(8), 284(35), 258(29), 257(100), 256(95), 228(18), 227(54), 225(18), 197(44), 170(17), 169(64), 167(39), 153(32), 141(31), 115(25), 105(7), 91(30), 77(15), 59(30), 41(9); HRMS (EI): m/z calcd for $C_{18}H_{20}O_5$ 316.1311; found 316.1310.

Dimethyl *(E)*-2-(1-(4-acetylphenyl)ethylidene)cyclopentane-1,1-dicarboxylate (8). Prepared in reaction of dimethyl 2-(hex-4-yn-1-yl)malonate (84.9 mg, 0.400 mmol) and 4-bromoacetophenone (99.5 mg, 0.500 mmol) under general procedure (90.5 mg, 0.274 mmol, yield 68%). Product was isolated as white solid after column chromatography on silica gel (15g, hex/AcOEt 9:1→8:2). ^{1}H NMR (400 MHz, $CDCl_3$) δ 7.92 – 7.87 (m, 2H), 7.27 – 7.23 (m, 2H), 3.77 (s, 6H), 2.56 (s, 3H), 2.39 (t, *J* = 6.8 Hz, 2H), 2.20 (ddd, *J* = 7.2, 6.1, 2.0 Hz, 2H), 1.93 (t, *J* = 2.0 Hz, 3H), 1.66 – 1.58 (m, 2H); ^{13}C NMR (101 MHz, $CDCl_3$) δ 197.6, 171.7, 150.2, 136.8, 135.3, 134.6, 128.4, 127.6, 63.5, 52.6, 39.1, 33.5, 26.4, 24.7, 21.9; IR (CH_2Cl_2): 2953, 2879, 2851, 1731, 1683, 1604, 1559, 1434, 1359, 1254, 1158, 1069, 957, 838, 706, 605 cm^{-1}; MS (EI): m/z (%) = 331(14), 330(42)[M^{+}], 299(6), 298(29), 272(30), 271(100), 270(81), 239(16), 227(40), 211(27), 197(20), 169(40), 167(35), 153(23), 152(20), 128(16), 115(14), 105(6), 91(10), 77(6), 59(22), 43(70); HRMS (EI): m/z calcd for $C_{19}H_{22}O_5$ 330.1467; found 330.1476.

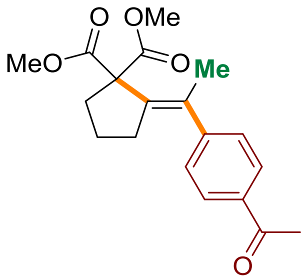

Dimethyl *(E)*-2-(1-(4-(methoxycarbonyl)phenyl)ethylidene) cyclopentane-1,1-dicarboxylate (9). Prepared in reaction of dimethyl 2-(hex-4-yn-1-yl)malonate (84.9 mg, 0.400 mmol) and methyl 4-bromobenzoate (108.5 mg, 0.506 mmol) under general procedure (121.3 mg, 0.353 mmol, yield 87%). Product was isolated as white solid after column chromatography on silica gel (15g, hex/AcOEt 8:2).^{1}H NMR (400 MHz, $CDCl_3$) δ 7.98 – 7.93 (m, 2H), 7.24 – 7.20 (m, 2H), 3.87 (s, 3H), 3.76 (s, 6H), 2.38 (t, *J* = 6.7 Hz, 2H), 2.23 – 2.16 (m, 2H), 1.92 (t, *J* = 2.0 Hz, 3H), 1.65 – 1.57 (m, 2H). ^{13}C NMR (101 MHz, $CDCl_3$) δ 171.7, 166.8, 149.9, 136.7, 134.6, 129.5, 128.2, 127.4, 63.4, 52.5, 51.9, 39.1, 33.5, 24.7, 21.9. IR (CH_2Cl_2): 3428, 2953, 2845, 1933, 1726, 1607, 1437, 1402, 1277, 1140, 1109, 1016, 860, 775, 710 cm^{-1}; HRMS (ESI): m/z calcd for $C_{19}H_{22}O_6Na$ 369.1314; found 369.1318.

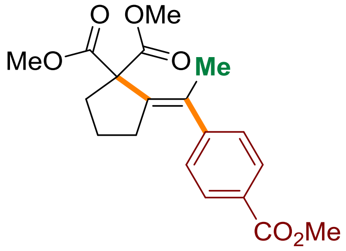

Dimethyl *(E)*-2-(1-(2-(methoxycarbonyl)phenyl)ethylidene)cyclopentane-1,1-dicarboxylate (10). Prepared in reaction of dimethyl 2-(hex-4-yn-1-yl)malonate (84.9 mg, 0.400 mmol) and methyl 2-bromobenzoate (106.9 mg, 0.497 mmol) under general procedure (114.2 mg, 0.330 mmol, yield 82%). Product was isolated as oil after column chromatography on silica gel (15g, hex/AcOEt 9:1). ^{1}H NMR (400 MHz, $CDCl_3$) δ 7.81 (dd, *J* = 7.8, 1.4 Hz, 1H), 7.45 (td, *J* = 7.5, 1.5 Hz, 1H), 7.28 (td, *J* = 7.6, 1.3 Hz, 1H), 7.12 (dd, *J* = 7.6, 1.3 Hz, 1H), 3.82 (s, 3H), 3.79 (s, 6H), 2.44 – 2.28 (m, 2H), 1.99 – 1.88 (m, 5H), 1.65 – 1.52 (m, 2H); ^{13}C NMR (101 MHz, $CDCl_3$) δ 168.0, 145.7, 135.6, 134.7, 132.0, 130.0, 129.1, 128.8, 126.5, 63.1, 52.4, 52.0, 39.2, 33.2, 24.5, 22.1; IR (CH_2Cl_2): 3464, 3062, 2952, 2878, 2849, 1730, 1598, 1434, 1292, 1254, 1157, 1081, 989, 864, 870, 762, 713, 595 cm^{-1}; MS (EI): m/z (%) = 347(2), 346(10)[M^{+}], 315(11), 314(31), 287(26), 286(32), 256(14), 255(53), 254(100), 227(29), 223(38), 222(26), 211(19), 196(30), 195(86), 194(42), 181(14), 167(41), 153(21), 152(33), 128(12), 115(13), 91(8), 77(7), 59(24); HRMS (EI): m/z calcd for $C_{19}H_{22}O_6$ 346.1416; found 346.1425.

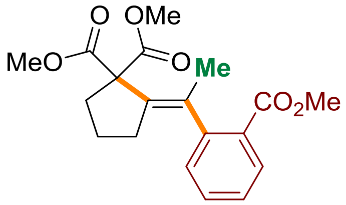

Dimethyl (E)-2-(1-(naphthalen-1-yl)ethylidene)cyclopentane-1,1-dicarboxylate (11). Prepared in reaction of dimethyl 2-(hex-4-yn-1-yl)malonate (84.9 mg, 0.400 mmol) and 1-bromonaphtalene (103.4 mg, 0.499 mmol) following general procedure (55.7 mg, 0.165 mmol, yield 41%). Product was isolated as oil after column chromatography on silica gel (15g, hex/AcOEt 9:1). ^{1}H NMR (400 MHz, $CDCl_3$) δ 8.00 – 7.96 (m, 1H), 7.88 – 7.84 (m, 1H), 7.75 (dt, *J* = 8.3, 1.1 Hz, 1H), 7.52 – 7.43 (m, 3H), 7.24 (dd, *J* = 7.0, 1.2 Hz, 1H), 3.91 (s, 3H), 3.86 (s, 3H), 2.58 – 2.38 (m, 2H), 2.15 – 2.00 (m, 4H), 1.96 – 1.84 (m, 1H), 1.69 – 1.52 (m, 2H); ^{13}C NMR (101 MHz, $CDCl_3$) δ 172.1, 172.0, 142.9, 137.5, 134.2, 133.9, 130.2, 128.3, 126.7, 126.1, 125.7, 125.7, 125.2, 124.1, 63.5, 52.7, 52.6, 39.4,

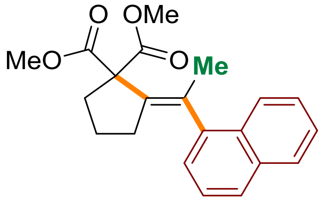

33.1, 24.6, 22.6; IR(CH_2Cl_2): 3054, 3040, 2998, 2952, 2877, 2844, 1936, 1730, 1590, 1505, 1435, 1394, 1376, 1314, 1262, 1142, 1110, 1088, 1020, 930, 867, 804, 782, 738, 617, 513, 471, 431 cm^{-1}; MS (EI): m/z (%) = 339 (11), 338 [M^+](47), 306(2), 279(14), 263(11), 247(48), 219(100), 203(28), 192(16), 179(28), 165(13), 152(6), 141(6), 128(3), 91(4), 71(1), 67(3), 59(6), 57(2); HRMS (EI): m/z calcd for $C_{21}H_{22}O_4$ 338.1518; found 338.1522.

dimethyl *(E)*-2-(1-(4-chlorophenyl)ethylidene)cyclopentane-1,1-dicarboxylate (12). Prepared in reaction of dimethyl 2-(hex-4-yn-1-yl)malonate (84.9 mg, 0.400 mmol) and 4-bromochlorobenzene (95.7 mg, 0.500 mmol) under general procedure (93.4 mg, 0.290 mmol, yield 72%). Product was isolated as oil after column chromatography on silica gel (15g, hex/AcOEt 9:1).^{1}H NMR (400 MHz, $CDCl_3$) δ 7.29 – 7.24 (m, 2H), 7.12 – 7.08 (m, 2H), 3.77 (s, 6H), 2.40 (t, *J* = 6.8 Hz, 2H), 2.22 (tq, *J* = 7.2, 2.0 Hz, 2H), 1.91 (t, *J* = 2.0 Hz, 3H), 1.68 – 1.58 (m, 2H); ^{13}C NMR (101 MHz, $CDCl_3$) δ 171.8, 143.5, 136.6, 134.4, 132.1, 128.8, 128.3, 63.4, 52.6, 39.2, 33.6, 24.7, 22.2; IR (CH_2Cl_2): 3453, 2952, 2877, 2842, 1731, 1593, 1488, 1434, 1394, 1314, 1266, 1251, 1189, 1171, 1140, 1090, 1014, 930, 911, 831, 792, 725, 665, 579, 560, 489 cm^{-1}; HRMS (ESI): m/z calcd for $C_{17}H_{19}O_4ClNa$ 345.0870; found 345.0871.

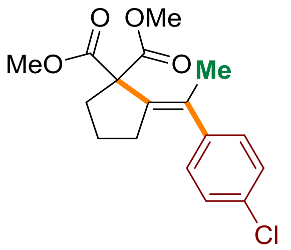

Dimethyl *(E)*-2-(1-(4-acetamidophenyl)ethylidene)cyclopentane-1,1-dicarboxylate (13). Prepared in reaction of dimethyl 2-(hex-4-yn-1-yl)malonate (84.9 mg, 0.400 mmol) and 4-bromoacetanilide (107.3 mg, 0.501 mmol) under general procedure (60.5 mg, 0.175 mmol, yield 44%). Product was isolated as yellow oil after column chromatography on silica gel (25g, hex/AcOEt 1:1).^{1}H NMR (400 MHz, $CDCl_3$) δ 7.79 (s, 1H), 7.45 (d, *J* = 8.2 Hz, 2H), 7.10 (d, *J* = 8.4 Hz, 2H), 3.77 (s, 6H), 2.39 (t, *J* = 6.8 Hz, 2H), 2.28 – 2.20 (m, 2H), 2.14 (s, 3H), 1.90 (t, *J* = 2.1 Hz, 3H), 1.62 (p, *J* = 6.9 Hz, 2H).^{13}C NMR (101 MHz, $CDCl_3$) δ 172.1, 168.5, 141.0, 136.3, 136.0, 134.8, 127.9, 119.7, 63.4, 52.6, 39.2, 33.6, 24.8, 24.4, 22.2; IR (CH_2Cl_2): 3361, 3305, 3182, 2953, 2925, 2853, 1731, 1668, 1597, 1529, 1435, 1401, 1371, 1315, 1261, 1179, 1140, 1019, 911, 839, 805, 735, 703, 659, 553, 506 cm^{-1}; HRMS (ESI): m/z calcd for $C_{19}H_{23}NO_5Na$ 368.1474; found 368.1480.

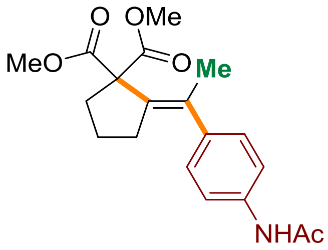

Dimethyl *(E)*-2-(1-(pyridin-3-yl)ethylidene)cyclopentane-1,1-dicarboxylate (14). Prepared in reaction of dimethyl 2-(hex-4-yn-1-yl)malonate (84.9 mg, 0.400 mmol) and 3-bromopyridine (79.0 mg, 0.500 mmol) under general procedure (98.1 mg, 0.339 mmol, yield 85%). Product was isolated as oil after column chromatography on silica gel (15g, hex/AcOEt 8:2→1:1). ^{1}H NMR (400 MHz, $CDCl_3$) δ 8.45 – 8.36 (m, 2H), 7.45 (dt, *J* = 7.9, 1.9 Hz, 1H), 7.18 (ddd, *J* = 7.8, 4.8, 0.9 Hz, 1H), 3.73 (s, 6H), 2.36 (t, *J* = 6.8 Hz, 2H), 2.23 – 2.15 (m, 2H), 1.90 (t, *J* = 2.0 Hz, 3H), 1.65 – 1.55 (m, 2H); ^{13}C NMR (101 MHz, $CDCl_3$) δ 171.6, 148.6, 147.6, 140.4, 137.9, 134.7, 131.9, 123.0, 63.4, 52.6, 39.0, 33.5, 24.7, 22.0.; IR (CH_2Cl_2): 3464, 3029, 2952, 2879, 2852, 1731, 1586, 1566, 1435, 1376, 1257, 1159, 1070, 952, 928, 869, 807, 715, 620 cm^{-1}; MS (EI): m/z (%) = 290(14), 289(43)[M^+], 257(16), 231(27), 230(100), 229(79), 228(16), 214(22), 171(25), 170(88), 154(22), 142(12), 130(11), 115(11), 92(15), 77(10), 59(22); HRMS (EI): m/z calcd for $C_{16}H_{19}NO_4$ 289.1314; found 289.1314.

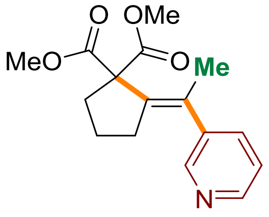

Dimethyl *(E)*-2-(1-(pyrimidin-5-yl)ethylidene)cyclopentane-1,1-dicarboxylate (15). Prepared in reaction of dimethyl 2-(hex-4-yn-1-yl)malonate (84.9 mg, 0.400 mmol) and 5-bromopyrimidine (79.6 mg, 0.501 mmol) under general procedure (112.0 mg, 0.386 mmol, yield 96%). Product was isolated as oil after column chromatography on silica gel (15g, hex/AcOEt 9:1→7:3). ^{1}H NMR (400 MHz, $CDCl_3$) δ 9.01 (s, 1H), 8.53 (s, 2H), 3.73 (s, 6H), 2.37 (t, *J* = 6.8 Hz, 2H), 2.21 (tq, *J* = 7.2, 2.1 Hz, 2H), 1.91 (t, *J* = 2.1 Hz, 3H), 1.67 – 1.59 (m, 2H). ^{13}C NMR (101 MHz, $CDCl_3$) δ 171.2, 156.8, 155.5, 139.9, 138.0,

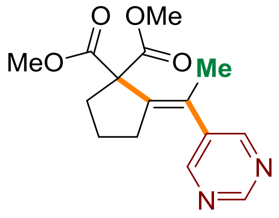

128.5, 63.6, 52.7, 38.9, 33.6, 24.7, 21.7. IR(CH_2Cl_2): 3458, 2957, 1731, 1635, 1550, 1436, 1246, 1120, 1066, 726, 632 cm^{-1}. MS (EI): m/z (%) = 291(7), 290(34)[M$^+$], 258(14), 232(20), 231(78), 230(100), 215(12), 199(13), 198(27), 172(19), 171(61), 157(10), 144(26), 117(20), 115(17), 103(9), 91(12), 77(10), 59(30). HRMS (EI): m/z calcd for $C_{15}H_{18}N_2O_4$ 290.1267; found 290.1272.

Dimethyl *(E)*-2-(1-(pyrazin-2-yl)ethylidene)cyclopentane-1,1-dicarboxylate (16). Prepared in reaction of dimethyl 2-(hex-4-yn-1-yl)malonate (84.9 mg, 0.400 mmol) and 2-bromopyrazine (80.0 mg, 0.506 mmol) under general procedure (66.8 mg, 0.230 mmol, yield 57%). Product was isolated as oil after column chromatography on silica gel (25g, hex/AcOEt 7:3→1:1).^{1}H NMR (400 MHz, $CDCl_3$) δ 8.57 – 8.50 (m, 2H), 8.39 (d, *J* = 2.5 Hz, 1H), 3.78 (s, 6H), 2.48 – 2.38 (m, 4H), 2.04 (t, *J* = 2.0 Hz, 3H), 1.70 (p, *J* = 7.0 Hz, 2H). ^{13}C NMR (101 MHz, $CDCl_3$) δ 171.4, 157.6, 144.4, 143.7, 142.2, 140.9, 131.5, 64.1, 52.8, 38.8, 33.6, 24.9, 20.1. IR (CH_2Cl_2): 3620, 3454, 2953, 2924, 2851, 1938, 1729, 1571, 1519, 1465, 1435, 1397, 1258, 1175, 1143, 1087, 1065, 1014, 931, 849, 800, 765, 659, 488 cm^{-1}; HRMS (ESI): m/z calcd for $C_{15}H_{18}N_2O_4Na$ 313.1164; found 313.1177.

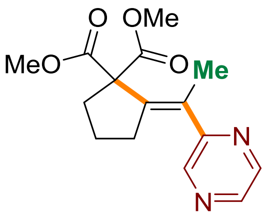

Dimethyl *(E)*-2-(1-(quinoxalin-6-yl)ethylidene)cyclopentane-1,1-dicarboxylate (17). Prepared in reaction of dimethyl 2-(hex-4-yn-1-yl)malonate (84.9 mg, 0.400 mmol) and 6-bromoquinoxaline (104.9 mg, 0.502 mmol) under general procedure (98 mg, 0.289 mmol, yield 72%). Product was isolated as oil after column chromatography on silica gel (15g, hex/AcOEt 9:1→8:2).^{1}H NMR (400 MHz, $CDCl_3$) δ 8.79 – 8.74 (m, 2H), 8.01 (d, *J* = 8.7 Hz, 1H), 7.86 (d, *J* = 1.9 Hz, 1H), 7.60 (dd, *J* = 8.6, 1.9 Hz, 1H), 3.77 (s, 6H), 2.40 (t, *J* = 6.8 Hz, 2H), 2.29 – 2.22 (m, 2H), 2.01 (t, *J* = 2.0 Hz, 3H), 1.62 (p, *J* = 7.0 Hz, 2H). ^{13}C NMR (101 MHz, $CDCl_3$) δ 171.6, 147.0, 145.0, 144.5, 143.0, 141.8, 137.5, 134.1, 130.4, 129.2, 127.3, 63.5, 52.6, 39.1, 33.6, 24.7, 22.0. IR(CH_2Cl_2): 3453, 2952, 2877, 2844, 1730, 1615, 1495, 1368, 1263, 1155, 1134, 1089, 1044, 1023, 956, 897, 835, 805, 768, 685, 609, 566 cm^{-1}; MS (EI): m/z (%) = 342(8), 341(45), 340(100), 308(2), 282(28), 281(84), 263(36), 250(33), 249(98), 247(98), 222(26), 221(75), 206(31), 195(21), 181(19), 167(10), 157(17), 143(17), 131(8), 113(5), 91(8), 77(3), 59(15), 41(4); HRMS (EI): m/z calcd for $C_{19}H_{20}N_2O_4$ 340.1423; found 340.1414.

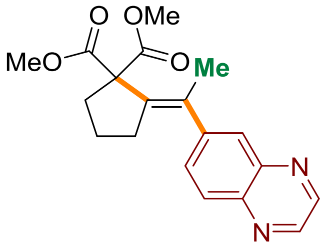

Dimethyl *(E)*-2-(1-(benzo[d]thiazol-5-yl)ethylidene)cyclopentane-1,1-dicarboxylate (18). Prepared in reaction of dimethyl 2-(hex-4-yn-1-yl)malonate (84.9 mg, 0.400 mmol) and 5-bromobenzothiazole (107.1 mg, 0.500 mmol) under general procedure (41.7 mg, 0.121 mmol, yield 30%). Product was isolated as oil after column chromatography on silica gel (15g, hex/AcOEt 8:2). ^{1}H NMR (400 MHz, $CDCl_3$) δ 8.98 (s, 1H), 7.95 (d, *J* = 1.6 Hz, 1H), 7.90 (d, *J* = 8.3 Hz, 1H), 7.29 (dd, *J* = 8.3, 1.6 Hz, 1H), 3.81 (s, 6H), 2.43 (t, *J* = 6.8 Hz, 2H), 2.30 – 2.24 (m, 2H), 2.02 (t, *J* = 2.0 Hz, 3H), 1.69 – 1.61 (m, 2H). ^{13}C NMR (101 MHz, $CDCl_3$) δ 171.9, 154.1, 153.50, 143.6, 136.8, 134.8, 131.7, 125.4, 122.2, 121.6, 63.5, 52.6, 39.2, 33.7, 24.8, 22.6. IR (CH_2Cl_2): 3075, 2951, 2878, 2849, 1729, 1538, 1437, 1260, 1155, 1067, 990, 887, 858, 815 cm^{-1}; MS (EI): m/z (%) = 346(11), 345(45)[M$^+$], 313(17), 287(32), 286(100), 285(49), 270(9), 254(20), 227(25), 226(87), 210(13), 198(21), 148(16), 115(6), 91(6), 59(17); HRMS (EI): m/z calcd for $C_{18}H_{19}NO_4S$ 345.1035; found 345.1038.

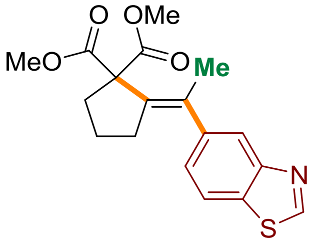

Dimethyl (*E*)-2-(1-(benzo[b]thiophen-5-yl)ethylidene)cyclopentane-1,1-dicarboxylate (19).

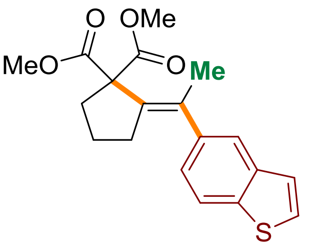

Prepared in reaction of dimethyl 2-(hex-4-yn-1-yl)malonate (84.9 mg, 0.400 mmol) and 5-bromobenzo[b]thiophene (106.9 mg, 0.502 mmol) under general procedure (104.8 mg, 0.301 mmol, yield 76%). Product was isolated as oil after column chromatography on silica gel (15g, hex/AcOEt 95:5→8:2). ^{1}H NMR (400 MHz, $CDCl_3$) δ 7.83 (d, *J* = 8.3 Hz, 1H), 7.65 (d, *J* = 1.7 Hz, 1H), 7.42 (d, *J* = 5.5 Hz, 1H), 7.29 (dd, *J* = 5.4, 0.8 Hz, 1H), 7.19 (dd, *J* = 8.2, 1.7 Hz, 1H), 3.82 (s, 6H), 2.44 (t, *J* = 6.8 Hz, 2H), 2.31 – 2.25 (m, 2H), 2.02 (t, *J* = 2.0 Hz, 3H), 1.66 (p, *J* = 7.0 Hz, 2H).^{13}C NMR (101 MHz, $CDCl_3$) δ 172.0, 141.5, 139.7, 137.8, 136.2, 135.5, 126.5, 124.1, 123.6, 122.2, 122.1, 63.4, 52.56, 39.3, 33.7, 24.7, 22.6. IR (CH_2Cl_2): 3464, 2951, 1731, 1542, 1434, 1258, 1154, 1070, 1053, 897, 704, 487 cm^{-1}. MS (EI): m/z (%) = 345(15), 344(44)[M^+], 286(32), 285(100), 284(29), 253(16), 147(14), 134(4), 115(6), 91(6), 77(2), 59(14), 45(2). HRMS (EI): m/z calcd for $C_{19}H_{20}O_4S$ 344.1078; found 344.1082.

Dimethyl (*E*)-2-(1-(2-methylbenzo[d]oxazol-5-yl)ethylidene) cyclopentane-1,1-dicarboxylate (20).

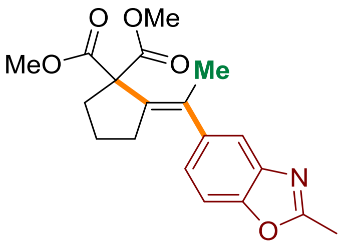

Prepared in reaction of dimethyl 2-(hex-4-yn-1-yl)malonate (84.9 mg, 0.400 mmol) and 5-bromo-2-methylbenzo[d]oxazole (105.8 mg, 0.499 mmol) under general procedure (125.3 mg, 0.365 mmol, yield 91%). Product was isolated as yellow solid after column chromatography on silica gel (15g, hex/AcOEt 8:2→7:3). ^{1}H NMR (400 MHz, $CDCl_3$) δ 7.44 – 7.37 (m, 2H), 7.10 (dd, *J* = 8.4, 1.7 Hz, 1H), 3.80 (s, 6H), 2.62 (s, 3H), 2.41 (t, *J* = 6.8 Hz, 2H), 2.25 – 2.20 (m, 2H), 1.97 (t, *J* = 2.0 Hz, 3H), 1.63 (p, *J* = 7.1 Hz, 2H).^{13}C NMR (101 MHz, $CDCl_3$) δ 172.01, 164.07, 149.60, 141.59, 136.41, 135.26, 124.00, 118.12, 109.77, 63.45, 52.62, 39.27, 33.67, 24.76, 22.77, 14.49. IR (CH_2Cl_2): 3458, 2954, 2935, 2851, 1730, 1622, 1578, 1433, 1379, 1263, 1156, 1115, 1068, 988, 918, 821, 674, 625, 568, 517, 431 cm^{-1}; MS (EI): m/z (%) = 344(8), 343(36)[M^+], 311(30), 285(20), 284(36), 283(20), 268(9), 252(21), 225(16), 224(66), 183(32), 184(8), 155(24), 128(8), 115(11), 91(7), 59(13), 43(12); HRMS (EI): m/z calcd for $C_{19}H_{21}NO_5$ 343.1420; found 343.1423.

dimethyl (*E*)-2-(1-(benzo[d][1,3]dioxol-5-yl)ethylidene)cyclopentane-1,1-dicarboxylate (21).

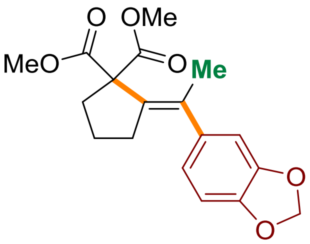

Prepared in reaction of dimethyl 2-(hex-4-yn-1-yl)malonate (84.9 mg, 0.400 mmol) and 1,2-(methylenedioxy)-4-bromobenzene (100.7 mg, 0.501 mmol) under general procedure (76.4 mg, 0.230 mmol, yield 57%). Product was isolated as yellow solid after column chromatography on silica gel (15g, hex/AcOEt 8:2).^{1}H NMR (400 MHz, $CDCl_3$) δ 6.75 (d, *J* = 7.9 Hz, 1H), 6.69 – 6.59 (m, 2H), 5.92 (s, 2H), 3.77 (s, 6H), 2.39 (t, *J* = 6.8 Hz, 2H), 2.27 (tq, *J* = 7.2, 2.0 Hz, 2H), 1.90 (t, *J* = 2.0 Hz, 3H), 1.64 (p, *J* = 7.1 Hz, 2H). ^{13}C NMR (101 MHz, $CDCl_3$) δ 172.0, 147.3, 145.8, 139.1, 136.0, 135.1, 120.5, 108.1, 108.0, 100.8, 63.4, 52.5, 39.2, 33.6, 24.7, 22.4. IR (CH_2Cl_2): 2952, 2883, 2844, 2778, 1730, 1605, 1503, 1488, 1433, 1246, 1227, 1146, 1103, 1087, 1038, 935, 885, 813, 653 cm^{-1}; HRMS (ESI): m/z calcd for $C_{18}H_{20}O_6Na$ 355.1158; found 355.1164.

Dimethyl (E)-2-(1-(thiophen-2-yl)ethylidene)cyclopentane-1,1-dicarboxylate (22). Prepared in reaction of dimethyl 2-(hex-4-yn-1-yl)malonate (84.9 mg, 0.400 mmol) and 2-bromothiophene (81.8 mg, 0.502 mmol) under general procedure (62.7 mg, 0.213 mmol, yield 53%). Product was isolated as oil after column chromatography on silica gel (15g, hex/AcOEt 8:2). ^{1}H NMR (400 MHz, $CDCl_3$) δ 7.25 (dd, *J* = 5.1, 1.2 Hz, 1H), 7.06 – 6.99 (m, 2H), 3.76 (s, 6H), 2.66 (td, *J* = 7.2, 2.0 Hz, 2H), 2.43 (t, *J* = 6.8 Hz, 2H), 2.09 (t, *J* = 2.0 Hz, 3H), 1.79 (p, *J* = 7.0 Hz, 2H); ^{13}C NMR (101 MHz, $CDCl_3$) δ 171.8, 146.1, 137.5, 127.7, 126.4, 125.3, 124.3, 64.8, 52.6, 39.2, 34.7, 25.1, 22.3; IR (CH_2Cl_2): 3107, 2952, 1731, 1434, 1373, 1249, 1159, 923, 827, 702 cm^{-1}; MS (EI): m/z (%) = 295(5), 294(30)[M^+], 236(26), 235(100),

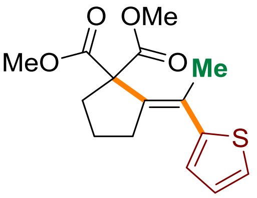

234(21), 219(7), 203(16), 175(48), 169(46), 161(37), 160(16), 147(13), 115(15), 91(13), 77(9), 59(13), 39(8); HRMS (EI): m/z calcd for $C_{15}H_{18}O_4S$ 294.0269; found 294.0925.

Dimethyl *(E)*-2-(1-(furan-2-yl)ethylidene)cyclopentane-1,1-dicarboxylate (23). Prepared in reaction of dimethyl 2-(hex-4-yn-1-yl)malonate (84.9 mg, 0.400 mmol) and 2-bromofuran (74.1 mg, 0.504 mmol) under general procedure (51.2 mg, 0.184 mmol, yield 46%). Product was isolated as oil after column chromatography on silica gel (15g, hex/AcOEt 8:2).^{1}H NMR (400 MHz, $CDCl_3$) δ 7.39 (dd, *J* = 1.9, 0.7 Hz, 1H), 6.41 (dd, *J* = 3.4, 1.8 Hz, 1H), 6.33 (dd, *J* = 3.4, 0.7 Hz, 1H), 3.75 (s, 6H), 2.77 (tt, *J* = 7.3, 2.0 Hz, 2H), 2.41 (t, *J* = 6.8 Hz, 2H), 2.00 (t, *J* = 2.0 Hz, 3H), 1.80 (p, *J* = 7.2 Hz, 2H). ^{13}C NMR (101 MHz, $CDCl_3$) δ 171.9, 155.7, 141.1, 136.8, 123.6, 111.0, 108.6, 65.2, 52.7, 39.0, 34.3, 25.1, 17.9. IR (CH_2Cl_2): 3455, 3148, 3118, 2953, 2879, 1731, 1633, 1434, 1386, 1314, 1264, 1174, 1138, 1092, 1065, 1022, 921, 885, 805, 740, 596 cm^{-1}; HRMS (ESI): m/z calcd for $C_{15}H_{18}O_5Na$ 301.1052; found 301.1055.

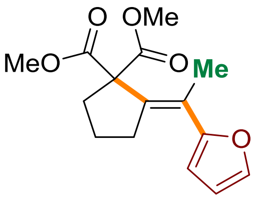

Dimethyl *(E)*-2-(1-(furan-3-yl)ethylidene)cyclopentane-1,1-dicarboxylate (24). Prepared in reaction of dimethyl 2-(hex-4-yn-1-yl)malonate (84.9 mg, 0.400 mmol) and 3-bromofuran (73.7 mg, 0.502 mmol) under general procedure (69.1 mg, 0.248 mmol, yield 62%). Product was isolated as oil after column chromatography on silica gel (15g, hex/AcOEt 8:2).^{1}H NMR (400 MHz, $CDCl_3$) δ 7.43 (t, *J* = 1.2 Hz, 1H), 7.36 (t, *J* = 1.7 Hz, 1H), 6.49 (dd, *J* = 1.9, 0.9 Hz, 1H), 3.75 (s, 6H), 2.59 – 2.50 (m, 2H), 2.40 (t, *J* = 6.8 Hz, 2H), 1.93 (t, *J* = 2.0 Hz, 3H), 1.76 (p, *J* = 7.1 Hz, 2H). ^{13}C NMR (101 MHz, $CDCl_3$) δ 171.9, 142.0, 140.3, 136.4, 127.9, 125.3, 110.3, 64.4, 52.6, 39.1, 34.3, 24.9, 20.7. IR(CH_2Cl_2): 3456, 3146, 2953, 2879, 2844, 1730, 1503, 1435, 1382, 1265, 1144, 1088, 1065, 1020, 874, 845, 733, 663, 599, 490 cm^1; MS (EI): m/z (%) = 278(21)[M$^+$], 260(4), 219(27), 201(38), 188(25), 187(100), 159(29), 145(7), 131(21), 117(10), 115(15), 105(8), 91(26), 81(12), 77(8), 59(13), 53(6), 43(6); HRMS (EI): m/z calcd for $C_{15}H_{18}O_5$ 278.1154; found 278.1162.

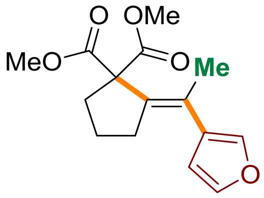

Dimethyl *(E)*-2-(pent-4-en-2-ylidene)cyclopentane-1,1-dicarboxylate (25). Prepared in reaction of dimethyl 2-(hex-4-yn-1-yl)malonate (84.9 mg, 0.400 mmol) and allyl bromide (60.9 mg, 0.503 mmol) under general procedure (80.4 mg, 0.319 mmol, yield 79%). Product was isolated as oil after column chromatography on silica gel (15g, hex/AcOEt 9:1).^{1}H NMR (400 MHz, $CDCl_3$) δ 5.69 – 5.56 (m, 1H), 5.12 – 5.03 (m, 2H), 3.69 (s, 6H), 2.62 (dt, *J* = 7.4, 1.2 Hz, 2H), 2.14 – 2.06 (m, 2H), 1.97 – 1.88 (m, 2H), 1.74 (t, *J* = 2.5 Hz, 3H), 1.42 – 1.30 (m, 2H). ^{13}C NMR (101 MHz, $CDCl_3$) δ 171.5, 132.3, 118.9, 78.3, 75.9, 57.4, 52.3, 37.1, 31.7, 23.8, 18.9, 3.3. IR (CH_2Cl_2): 3468, 3078, 2953, 1736, 1641, 1435, 1369, 1207, 1088, 997, 924, 881, 692, 656 cm^{-1}; MS(EI): m/z (%) = 252(1), 231(1), 221(12), 199(3), 193(22), 189(6), 179(31), 172(64), 161(24), 149(33), 139(24), 133(100), 121(7), 119(19), 117(23), 105(42), 91(37), 79(37), 71(11), 66(37), 59(28), 45(5), 43(10), 41(27); HRMS (ESI): m/z calcd for $C_{14}H_{20}O_4Na$ 275.1259; found 275.1251.

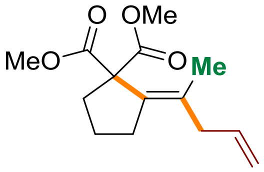

Methyl *(E)*-1-acetyl-2-(1-phenylethylidene)cyclopentane-1-carboxylate (26a). Prepared in reaction of methyl 2-acetyloct-6-ynoate (78.5 mg, 0.400 mmol) and bromobenzene (79.0 mg, 0.503 mmol) under general procedure (87.9 mg, 0.323 mmol, yield 81%). Product was isolated as oil after column chromatography on silica gel (15g, hex/AcOEt 8:2).^{1}H NMR (400 MHz, $CDCl_3$) δ 7.35 – 7.29 (m, 2H), 7.25 – 7.15 (m, 4H), 3.79 (s, 3H), 2.54 (dt, *J* = 12.9, 6.0 Hz, 1H), 2.34 – 2.24 (m, 5H), 2.16 – 2.07 (m, 1H), 1.92 (t, *J* = 2.0 Hz, 3H), 1.71 – 1.60 (m, 2H). ^{13}C NMR (101 MHz, $CDCl_3$) δ 205.1, 172.3, 144.9, 136.3, 135.6, 128.2, 127.1, 126.5, 70.5, 52.4, 37.6, 33.9, 26.3, 24.8, 22.7. IR(CH_2Cl_2): 3388, 3078, 3019, 2952, 2877, 1954, 1883, 1736, 1709, 1575, 1493, 1435, 1355, 1314, 1237, 1176, 1132, 1072, 908, 840, 767, 703, 481 cm^{-1}; MS(EI): m/z (%) = 272(1)[M$^+$], 254(8), 231(2), 230(87), 229(10), 1213(15), 199(26), 19(100), 197(70), 195(32), 183(21), 171(22), 170(42), 169(94), 155(26), 154(30), 141(28), 129(20), 115(11), 105(14), 91(28), 77(8), 73(48); HRMS (EI): m/z calcd for $C_{17}H_{20}O_3$ 272.1412; found 272.1416.

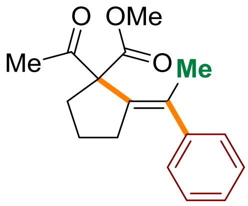

Methyl (*E*)-1-acetyl-2-(1-(4-methoxyphenyl)ethylidene)cyclopentane-1-carboxylate (26b).

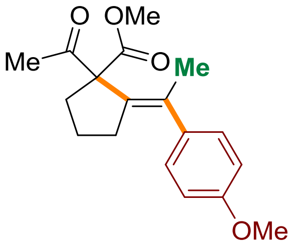

Prepared in reaction of methyl 2-acetyloct-6-ynoate (78.5 mg, 0.400 mmol) and 4-bromoanisol (94.1 mg, 0.503 mmol) under general procedure (72.3 mg, 0.239 mmol, yield 56%). Product was isolated as oil after column chromatography on silica gel (15g, hex/AcOEt 8:2).^{1}H NMR (400 MHz, $CDCl_3$) δ 7.14 – 7.07 (m, 2H), 6.90 – 6.80 (m, 2H), 3.79 (d, *J* = 6.2 Hz, 6H), 2.57 – 2.49 (m, 1H), 2.34 – 2.25 (m, 5H), 2.15 – 2.06 (m, 1H), 1.89 (t, *J* = 2.0 Hz, 3H), 1.69 – 1.59 (m, 2H). ^{13}C NMR (101 MHz, $CDCl_3$) δ 205.2, 172.4, 158.1, 137.3, 136.1, 135.1, 128.3, 113.5, 70.5, 55.2, 52.3, 37.6, 34.0, 26.4, 24.8, 22.8. IR(CH_2Cl_2): 2999, 2952, 2837, 2054, 1735, 1708, 1607, 1575, 1509, 1436, 1355, 1243, 1175, 1131, 1032, 905, 833, 741, 547, 506 cm^{-1}; MS (EI): m/z (%) = 303(3), 302(15)[M$^+$], 260(44), 259(100), 243(18), 229(19), 228(71), 227(96), 225(28), 213(15), 200(31), 199(94), 185(22), 172(14), 141(11), 121(15), 115(8) 91(16), 77(6), 43(35); HRMS (EI): m/z calcd for $C_{18}H_{22}O_4$ 302.1518; found 302.1508.

Methyl (*E*)-1-acetyl-2-(1-(4-cyanophenyl)ethylidene)cyclopentane-1-carboxylate (26c). Prepared

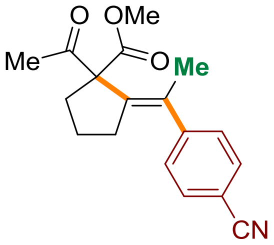

in reaction of methyl 2-acetyloct-6-ynoate (78.5 mg, 0.400 mmol) and 4-bromobenzonitrile (89.3 mg, 0.491 mmol) under general procedure (116.8 mg, 0.39 mmol, yield 98%). Product was isolated as oil after column chromatography on silica gel (15g, hex/AcOEt 7:3). ^{1}H NMR (400 MHz, $CDCl_3$) δ 7.63 – 7.58 (m, 2H), 7.31 – 7.26 (m, 2H), 2.53 (dt, *J* = 12.9, 6.0 Hz, 1H), 2.30 (s, 3H), 2.24 – 2.18 (m, 2H), 2.14 – 2.06 (m, 1H), 1.87 (t, *J* = 2.0 Hz, 3H), 1.69 – 1.61 (m, 2H); ^{13}C NMR (101 MHz, $CDCl_3$) δ 204.1, 172.0, 149.7, 137.9, 133.9, 132.2, 128.2, 118.8, 110.3, 70.4, 52.5, 37.6, 33.9, 26.6, 24.8, 22.1; IR(CH_2Cl_2): 3405, 2953, 2878, 2227, 1929, 1734, 1709, 1604, 1502, 1434, 1314, 1261, 1238, 1166, 1132, 1089, 1069, 905, 843, 739, 628, 575, 530 cm^{-1}; MS (EI): m/z (%) = 297(1)[M$^+$], 279(3), 256(30), 255(100), 238(16), 224(16), 223(58), 222(46), 208(13), 196(24), 195(27), 194(39), 181(4), 180(15), 166(19), 154(16), 140(8), 130(10), 128(5), 116(18), 91(5), 77(33), 59(5), 43(60); HRMS (EI): m/z calcd for $C_{18}H_{19}NO_3$ 297.1365; found 297.1368.

Isopropyl (*E*)-1-acetyl-2-(1-phenylethylidene)cyclopentane-1-carboxylate (27a). Prepared in

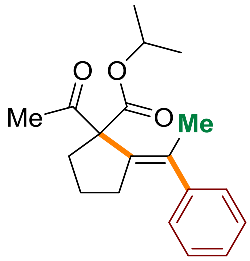

reaction of isopropyl 2-acetyloct-6-ynoate (89.7 mg, 0.400 mmol) and bromobenzene (78.2 mg, 0.498 mmol) under general procedure (74.9 mg, 0.250 mmol, yield 63%). Product was isolated as oil after column chromatography on silica gel (15g, hex/AcOEt 9:1). ^{1}H NMR (400 MHz, $CDCl_3$) δ 7.36 – 7.28 (m, 2H), 7.25 – 7.19 (m, 1H), 7.19 – 7.12 (m, 2H), 5.12 (p, *J* = 6.2 Hz, 1H), 2.57 – 2.49 (m, 1H), 2.32 (s, 3H), 2.29 – 2.22 (m, 2H), 2.12 – 2.02 (m, 1H), 1.70 – 1.58 (m, 2H), 1.30 (dd, *J* = 6.3, 2.0 Hz, 6H); ^{13}C NMR (101 MHz, $CDCl_3$) δ 205.1, 171.2, 145.2, 136.5, 135.3, 128.2, 127.1, 126.4, 70.5, 68.7, 37.6, 33.9, 26.6, 24.8, 23.1, 21.7, 21.6; IR (CH_2Cl_2): 3055, 3018, 2979, 2939, 2876, 2227, 1728, 1710, 1600, 1442, 1375, 1313, 1238, 1178, 1107, 952, 850, 830, 766, 703, 595 cm^{-1}; MS (EI): m/z (%) = 300(4)[M$^+$], 282(12), 259(27), 258(92), 216(65), 215(71), 213(39), 198(100), 197(65), 187(35), 170(38), 169(92), 155(24), 142(29), 141(44), 129(31), 115(27), 105(19), 91(33), 77(16), 43(96); HRMS (EI): m/z calcd for $C_{19}H_{24}O_3$ 300.1725; found 300.1724.

Isopropyl (*E*)-1-acetyl-2-(1-(4-methoxyphenyl)ethylidene)cyclopentane-1-carboxylate (27b).

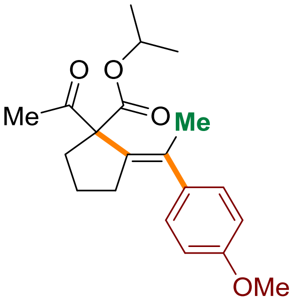

Prepared in reaction of isopropyl 2-acetyloct-6-ynoate (89.7 mg, 0.400 mmol) and 4-bromoanisole (94.0 mg, 0.500 mmol) under general procedure (78.6 mg, 0.238 mmol, yield 59%). Product was isolated as oil after column chromatography on silica gel (15g, hex/AcOEt 8:2). ^{1}H NMR (400 MHz, $CDCl_3$) δ 7.13 – 7.04 (m, 2H), 6.91 – 6.80 (m, 2H), 5.11 (hept, *J* = 6.3 Hz, 1H), 3.79 (s, 3H), 2.57 – 2.47 (m, 1H), 2.34 – 2.24 (m, 6H), 2.11 – 2.01 (m, 1H), 1.89 (t, *J* = 2.0 Hz, 3H), 1.69 – 1.57 (m, 2H), 1.28 (dd, *J* = 6.3, 1.7 Hz, 6H); ^{13}C NMR (101 MHz, $CDCl_3$) δ 205.2, 171.2,

158.1, 137.5, 136.3, 134.8, 128.3, 113.6, 70.5, 68.7, 55.1, 37.7, 34.0, 26.5, 24.8, 23.2, 21.7, 21.6; IR (CH_2Cl_2): 3031, 2979, 2876, 2837, 1727, 1709, 1607, 1509, 1455, 1374, 1243, 1176, 1134, 1107, 1033, 939, 833, 722, 508 cm^{-1}; MS (EI): m/z (%) = 330(12)[M^+], 288(26), 287(50), 246(31), 245(100), 228(52), 227(51), 225(26), 213(11), 199(49), 185(14), 172(12), 157(7), 128(10), 121(10), 91(9), 77(5), 43(50); HRMS (EI): m/z calcd for $C_{20}H_{26}O_4$ 330.1831; found 330.1828.

Isopropyl *(E)*-1-acetyl-2-(1-(4-cyanophenyl)ethylidene)cyclopentane-1-carboxylate (27c).

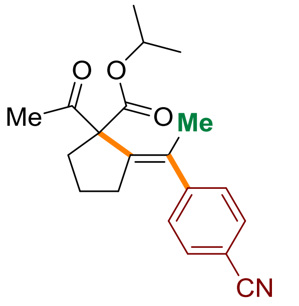

Prepared in reaction of isopropyl 2-acetyloct-6-ynoate (89.7 mg, 0.400 mmol) and 4-bromobenzonitrile (91.7 mg, 0.506 mmol) under general procedure (94.7 mg, 0.291 mmol, yield 73%). Product was isolated as oil after column chromatography on silica gel (15g, hex/AcOEt 9:1→8:2). ^{1}H NMR (400 MHz, $CDCl_3$) δ 7.61 – 7.55 (m, 2H), 7.28 – 7.22 (m, 2H), 5.08 (hept, J = 6.2 Hz, 1H), 2.54 – 2.45 (m, 1H), 2.28 (s, 3H), 2.22 – 2.13 (m, 2H), 2.09 – 2.00 (m, 1H), 1.85 (t, J = 2.0 Hz, 3H), 1.69 – 1.57 (m, 2H), 1.25 (d, J = 6.2 Hz, 6H); ^{13}C NMR (101 MHz, $CDCl_3$) δ 204.1, 170.8, 149.8, 138.0, 133.5, 132.7, 132.1, 128.1, 127.8, 118.7, 110.2, 70.3, 68.9, 37.6, 33.9, 26.8, 24.7, 22.4, 21.6, 21.5; IR (CH_2Cl_2): 2980, 2877, 2227, 1727, 1709, 1604, 1503, 1452, 1356, 1314, 1239, 1181, 1106, 1018, 904, 844, 740, 578, 503 cm^{-1}; HRMS (ESI): m/z calcd for $C_{20}H_{23}NO_3Na$ 348.1576; found 348.1575.

Methyl *(E)*-1-isobutyryl-2-(1-phenylethylidene)cyclopentane-1-carboxylate (28a).

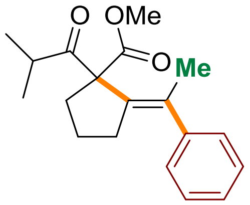

Prepared in reaction of methyl 2-isobutyryloct-6-ynoate (89.7 mg, 0.400 mmol) and bromobenzene (78.4 mg, 0.499 mmol) under general procedure (88.2 mg, 0.294 mmol, yield 73%). Product was isolated as oil after column chromatography on silica gel (15g, hex/AcOEt 9:1). ^{1}H NMR (400 MHz, $CDCl_3$) δ 7.36 – 7.27 (m, 2H), 7.26 – 7.14 (m, 3H), 3.79 (s, 3H), 3.08 (hept, J = 6.6 Hz, 1H), 2.54 – 2.43 (m, 1H), 2.32 – 2.23 (m, 3H), 1.87 (t, J = 2.0 Hz, 3H), 1.65 – 1.55 (m, 2H), 1.18 (dd, J = 6.7, 4.1 Hz, 6H); ^{13}C NMR (101 MHz, $CDCl_3$) δ 211.2, 172.7, 145.3, 135.7, 135.6, 128.2, 127.2, 126.4, 70.5, 52.2, 37.7, 37.5, 34.2, 24.7, 22.8, 20.9, 20.7; IR (CH_2Cl_2): 3417, 3055, 2973, 2874, 1706, 1599, 1441, 1380, 1253, 1161, 1095, 1028, 916, 845, 766, 703, 525 cm^{-1}; MS (EI): m/z (%) = 300(2)[M^+], 241(9), 231(12), 230(62), 229(33), 228(15), 199(13), 198(62), 197(71), 183(13), 170(33), 169(100), 154(21), 141(33), 128(19), 105(19), 91(23), 77(16), 71(54), 43(78). HRMS (EI): m/z calcd for $C_{17}H_{24}O_3$ 300.1725; found 300.1725.

Methyl *(E)*-1-isobutyryl-2-(1-(4-methoxyphenyl)ethylidene)cyclopentane-1-carboxylate (28b).

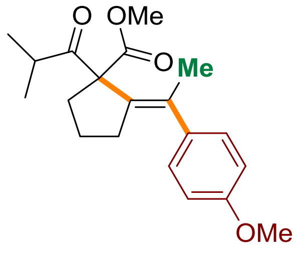

Prepared in reaction of methyl 2-isobutyryloct-6-ynoate (89.7 mg, 0.400 mmol) and 4-bromoanisole (93.8 mg, 0.502 mmol) under general procedure (68.8 mg, 0.208 mmol, yield 52%). Product was isolated as oil after column chromatography on silica gel (15g, hex/AcOEt 8:2) ^{1}H NMR (400 MHz, $CDCl_3$) δ 7.13 – 7.08 (m, 2H), 6.87 – 6.83 (m, 2H), 3.79 (s, 3H), 3.78 (s, 3H), 3.10 – 3.02 (m, 1H), 2.51 – 2.42 (m, 1H), 2.34 – 2.25 (m, 3H), 1.84 (t, J = 2.0 Hz, 3H), 1.64 – 1.55 (m, 2H), 1.17 (dd, J = 6.7, 5.4 Hz, 6H); ^{13}C NMR (101 MHz, $CDCl_3$) δ 211.3, 172.8, 158.1, 137.6, 135.4, 135.2, 128.4, 113.5, 70.6, 55.1, 52.2, 37.6, 37.6, 34.3, 24.7, 22.9, 20.9, 20.7; IR (CH_2Cl_2): 2953, 2874, 2837, 1710, 1607, 1510, 1462, 1380, 1243, 1175, 1091, 1034, 930, 909, 833, 670, 565, 431 cm^{-1}; MS (EI): m/z (%) = 330(2)[M^+], 271(7), 260(37), 259(100), 228(48), 227(83), 200(25), 199(90), 184(15), 167(26), 158(6), 141(9), 128(8), 115(7), 91(9), 71(19), 43(46); HRMS (EI): m/z calcd for $C_{20}H_{26}O_4$ 330.1831; found 330.1845.

Methyl*(E)*-2-(1-(4-cyanophenyl)ethylidene)-1-isobutyrylcyclopentane-1-carboxylate (28c).

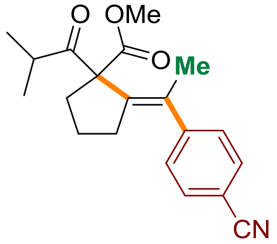

Prepared in reaction of methyl 2-isobutyryloct-6-ynoate (89.7 mg, 0.400 mmol) and 4-bromobenzonitrile (92.1 mg, 0.506 mmol) under modified procedure (run at 100°C)(113.2 mg, 0.348 mmol, yield 87%). Product was isolated as oil after column chromatography on silica gel (15g, hex/AcOEt 8:2). ^{1}H NMR (400 MHz, $CDCl_3$) δ 7.62 – 7.56 (m, 2H), 7.30 – 7.24 (m, 2H), 3.77 (s, 3H), 3.02 (hept, *J* = 6.6 Hz, 1H), 2.53 – 2.45 (m, 1H), 2.29 – 2.14 (m, 3H), 1.81 (t, *J* = 2.0 Hz, 3H), 1.68 – 1.54 (m, 2H), 1.15 (dd, *J* = 11.1, 6.7 Hz, 6H).^{13}C NMR (101 MHz, $CDCl_3$) δ 210.5, 172.4, 150.0, 137.4, 134.0, 132.2, 128.2, 118.8, 110.3, 70.5, 52.4, 37.9, 37.5, 34.2, 26.8, 24.7, 22.2, 20.9, 20.7. HRMS (ESI): m/z calcd for $C_{20}H_{23}NO_3Na$ 348.1576; found 348.1577.

Methyl *(E)*-1-cyano-2-(1-phenylethylidene)cyclopentane-1-carboxylate (29a). Prepared in reaction

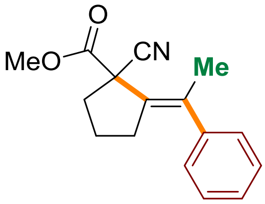

of methyl 2-cyanooct-6-ynoate (71.7 mg, 0.400 mmol) and bromobenzene (78.5 mg, 0.500 mmol) under general procedure (75.1 mg, 0.294 mmol, yield 73%). Product was isolated as oil after column chromatography on silica gel (15g, hex/AcOEt 9:1→8:2). ^{1}H NMR (400 MHz, $CDCl_3$) δ 7.35 – 7.30 (m, 2H), 7.27 – 7.17 (m, 3H), 3.86 (s, 3H), 2.58 – 2.50 (m, 1H), 2.43 – 2.35 (m, 3H), 2.08 (t, *J* = 2.0 Hz, 3H), 1.94 – 1.84 (m, 1H), 1.81 – 1.72 (m, 1H); ^{13}C NMR (101 MHz, $CDCl_3$) δ 169.9, 143.1, 136.0, 135.4, 128.2, 127.2, 127.0, 119.1, 53.5, 50.1, 41.0, 33.3, 25.3, 21.3; IR (CH_2Cl_2): 3055, 3022, 2955, 2875, 2240, 1743, 1600, 1575, 1493, 1437, 1381, 1312, 1244, 1160, 1072, 971, 907, 845, 767, 703, 593, 568, 515 cm^{-1}; MS (EI): m/z (%) = 256(4), 255(22)[M^{+}], 197(29), 196(100), 181(13), 180(17), 169(28), 168(18), 155(23), 154(28), 129(16), 115(15), 77(15), 59(10), 39(7); HRMS (EI): m/z calcd for $C_{16}H_{17}NO_2$ 255.1259; found 255.1254.

Methyl *(E)*-1-cyano-2-(1-(4-methoxyphenyl)ethylidene)cyclopentane-1-carboxylate (29a).

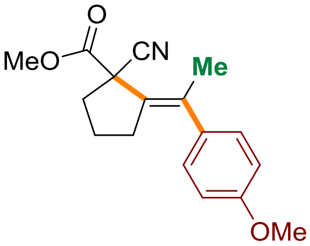

Prepared in reaction of methyl 2-cyanooct-6-ynoate (71.7 mg, 0.400 mmol) and 4-bromoanisole (93.5 mg, 0.500 mmol) under general conditions (102.7 mg, 0.360 mmol, yield 90%). Product was isolated as oil after column chromatography on silica gel (15g, hex/AcOEt 9:1→8:2). ^{1}H NMR (400 MHz, $CDCl_3$) δ 7.17 – 7.10 (m, 2H), 6.89 – 6.82 (m, 2H), 3.86 (s, 3H), 3.80 (s, 3H), 2.58 – 2.50 (m, 1H), 2.46 – 2.33 (m, 3H), 2.06 (t, *J* = 2.0 Hz, 3H), 1.94 – 1.84 (m, 1H), 1.83 – 1.71 (m, 1H); ^{13}C NMR (101 MHz, $CDCl_3$) δ 170.0, 158.5, 135.5, 135.5, 134.9, 128.4, 119.2, 113.5, 55.2, 53.4, 50.2, 41.0, 33.5, 25.4, 21.3; IR (CH_2Cl_2): 2956, 2839, 2239, 2054, 1742, 1607, 1511, 1439, 1246, 1178, 1031, 970, 835, 787, 742 cm^{-1}; MS (EI): m/z (%) = 286(10), 285(37)[M^{+}], 270(4), 227(30), 226(100), 210(6), 199(7), 185(16), 183(13), 170(5), 159(6), 141(6), 121(7), 115(7), 108(2), 91(10), 77(4), 59(4); HRMS (EI): m/z calcd for $C_{17}H_{19}NO_3$ 285.1365; found 285.1376.

Methyl *(E)*-1-cyano-2-(1-(4-cyanophenyl)ethylidene)cyclopentane-1-carboxylate (29a). Prepared

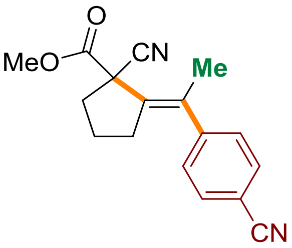

in reaction of methyl 2-cyanooct-6-ynoate (71.7 mg, 0.400 mmol) and 4-bromobenzonitrile (92.5 mg, 0.508 mmol) under general procedure (98.1 mg, 0.350 mmol, yield 87%). Product was isolated as oil after column chromatography on silica gel (15g, hex/AcOEt 9:1→8:2).^{1}H NMR (400 MHz, $CDCl_3$) δ 7.65 – 7.59 (m, 2H), 7.33 – 7.27 (m, 2H), 3.85 (s, 3H), 2.58 – 2.50 (m, 1H), 2.42 – 2.30 (m, 3H), 2.05 (t, *J* = 2.1 Hz, 3H), 1.96 – 1.86 (m, 1H), 1.83 – 1.71 (m, 1H); ^{13}C NMR (101 MHz, $CDCl_3$) δ 169.4, 147.7, 137.3, 134.4, 132.1, 128.1, 118.6, 118.5, 110.9, 53.6, 50.1, 40.8, 33.3, 25.3, 20.8; IR (CH_2Cl_2): 2956, 2876, 2228, 1742, 1605, 1503, 1435, 1401, 1263, 1246, 1163, 1081, 1017, 841, 788, 740, 580 cm^{-1}; MS (EI): m/z (%) = 281(7), 280(25)[M^{+}], 236(19), 235(26), 222(29), 221(100), 206(13), 205(17), 194(20), 180(19), 179(19), 167(18), 154(17), 140(15), 130(18),

116(23), 91(9), 77(7), 59(19), 41(12); HRMS (EI): m/z calcd for $C_{17}H_{16}N_2O_2$ 280.1212; found 280.1213.

***(E)*-1-(2-(1-phenylethylidene)-1-(phenylsulfonyl)cyclopentyl)ethan-1-one (30a).** Prepared in reaction of 3-(phenylsulfonyl)non-7-yn-2-one (111.4 mg, 0.400 mmol) and bromobenzene (78.3 mg, 0.499 mmol) under general procedure (77.8 mg, 0.220 mmol, yield 55%). Product was isolated as oil after column chromatography on silica gel (15g, hex/AcOEt 9:1). ^{1}H NMR (400 MHz, $CDCl_3$) δ 8.16 – 8.10 (m, 2H), 7.66 – 7.59 (m, 1H), 7.56 – 7.49 (m, 2H), 7.35 – 7.29 (m, 2H), 7.28 – 7.21 (m, 1H), 7.12 – 7.07 (m, 2H), 2.81 – 2.71 (m, 1H), 2.33 (s, 2H), 2.29 – 2.18 (m, 2H), 2.18 – 2.05 (m, 1H), 1.91 – 1.81 (m, 1H), 1.77 (dd, J = 2.5, 1.2 Hz, 3H), 1.63 – 1.51 (m, 1H); ^{13}C NMR (101 MHz, $CDCl_3$) δ 201.8, 144.0, 139.3, 138.0, 134.3, 133.7, 131.2, 128.5, 128.3, 127.0, 126.8, 85.8, 35.5, 34.7, 26.9, 24.2, 22.8; IR (CH_2Cl_2): 3407, 3059, 3020, 2963, 2879, 1712, 1599, 1583, 1445, 1356, 1303, 1206, 1146, 1083, 1025, 970, 881, 849, 762, 703, 579, 461 cm^{-1}; HRMS (ESI): m/z calcd for $C_{21}H_{22}O_3NaS$ 377.1187; found 377.1177.

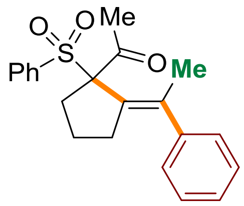

***(E)*-1-(2-(1-(4-methoxyphenyl)ethylidene)-1-(phenylsulfonyl) cyclopentyl)ethan-1-one (30b).** Prepared in reaction of 3-(phenylsulfonyl)non-7-yn-2-one (111.4 mg, 0.400 mmol) and 4-bromoanisole (93.8 mg, 0.502 mmol) under modified procedure (run at 100°C, 5 mol% cat.) (47.6 mg, 0.124 mmol, yield 31%). Product was isolated as oil after column chromatography on silica gel (25g, hex/AcOEt 8:2→7:3). ^{1}H NMR (400 MHz, $CDCl_3$) δ 8.15 – 8.09 (m, 2H), 7.66 – 7.60 (m, 1H), 7.56 – 7.50 (m, 2H), 7.06 – 7.01 (m, 2H), 6.88 – 6.83 (m, 2H), 3.80 (s, 3H), 2.82 – 2.70 (m, 1H), 2.34 (s, 3H), 2.28 – 2.10 (m, 3H), 1.91 – 1.81 (m, 1H), 1.75 (dd, J = 2.4, 1.1 Hz, 3H), 1.61 – 1.51 (m, 1H); ^{13}C NMR (101 MHz, $CDCl_3$) δ 201.9, 158.6, 138.8, 138.0, 136.3, 133.9, 133.7, 131.3, 128.5, 128.2, 113.6, 85.9, 55.2, 35.5, 34.9, 27.0, 24.3, 22.8; IR (CH_2Cl_2): 3062, 2957, 2957, 2838, 1712, 1607, 1510, 1446, 1302, 1246, 1206, 1146, 1080, 1031, 967, 935, 881, 835, 785, 690, 458 cm^{-1}; HRMS (ESI): m/z calcd for $C_{22}H_{24}O_4NaS$ 407.1293; found 407.1288.

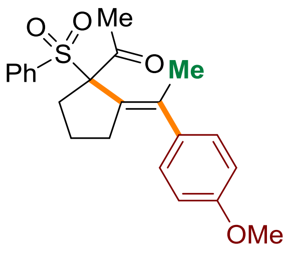

***(E)*-4-(1-(2-acetyl-2-(phenylsulfonyl)cyclopentylidene)ethyl)benzonitrile (30c).** Prepared in reaction of 3-(phenylsulfonyl)non-7-yn-2-one (111.4 mg, 0.400 mmol) and 4-bromobenzonitrile (91.0 mg, 0.500 mmol) under modified procedure (run at 100°C, 5 mol% cat.)(68.3 mg, 0.180 mmol, yield 45%, E/Z 85:15). Product was isolated as oil after column chromatography on silica gel (25g, hex/AcOEt 8:2→7:3). ^{1}H NMR (400 MHz, $CDCl_3$) δ 8.14 – 8.04 (m, 2H), 7.70 – 7.58 (m, 3H), 7.59 – 7.50 (m, 2H), 7.24 – 7.14 (m, 2H), 2.77 – 2.64 (m, 1H), 2.41 – 2.32 (m, 3H), 2.25 – 2.03 (m, 3H), 1.93 – 1.78 (m, 4H), 1.64 – 1.48 (m, 1H); ^{13}C NMR (101 MHz, $CDCl_3$) δ 201.1, 148.8, 137.9, 137.9, 137.7, 135.9, 134.0, 132.3, 131.1, 131.1, 128.6, 127.9, 118.6, 111.0, 85.9, 35.8, 34.9, 27.2, 27.1, 24.1, 22.8, 22.8; Indicative signals of minor isomer ^{1}H NMR (400 MHz, $CDCl_3$) δ 4.15 – 4.04 (m, 1H), 2.04 – 1.99 (m, 3H), 1.27 – 1.18 (m, 3H); ^{13}C NMR (101 MHz, $CDCl_3$) δ 201.1, 21.0, 14.2; IR (CH_2Cl_2) : 3626, 3546, 3407, 3064, 2961, 2228, 1713, 1604, 1502, 1446, 1358, 1304, 1209, 1144, 1080, 1019, 915, 845, 735, 571, 452 cm^{-1}; MS (EI): m/z (%) = 337(1), 240(2), 239(21), 238(100), 210(7), 196(46), 167(11), 166(15), 154(7), 130(6), 116(6), 91(3), 77(18), 51(7), 43(63); HRMS (ESI): m/z calcd for $C_{22}H_{21}NO_3NaS$ 402.1140; found 402.1133.

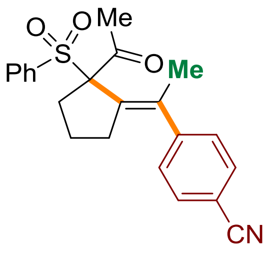

Ethyl *(E)*-1-(methylsulfonyl)-2-(1-phenylethylidene)cyclopentane-1-carboxylate (31a). Prepared in reaction of ethyl 2-(methylsulfonyl)oct-6-ynoate (98.5 mg, 0.400 mmol) and bromobenzene (78.6 mg, 0.501 mmol) under general procedure (86.8 mg, 0.270 mmol, yield 68%). Product was isolated as oil after column chromatography on silica gel (15g, hex/AcOEt 8:2). ^{1}H NMR (400 MHz, $CDCl_3$) δ 7.34 – 7.28 (m, 2H), 7.26 – 7.16 (m, 3H), 4.38 – 4.23 (m, 2H), 3.19 (s, 3H), 2.70 – 2.60 (m, 1H), 2.48 – 2.27 (m, 3H), 2.06 (dd, J = 2.5, 1.1 Hz,

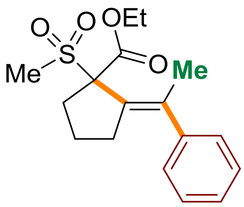

3H), 1.94 – 1.84 (m, 1H), 1.72 – 1.59 (m, 1H), 1.34 (t, J = 7.1 Hz, 3H). ^{13}C NMR (101 MHz, $CDCl_3$) δ 170.4, 144.4, 139.4, 132.3, 128.1, 127.2, 126.9, 77.92, 62.2, 39.5, 38.0, 35.0, 24.6, 23.2, 14.0; IR (CH_2Cl_2): 3628, 2976, 1728, 1599, 1493, 1443, 1367, 1306, 1237, 1131, 1072, 1024, 953, 921, 845, 766, 704, 539, 504 cm^{-1}; HRMS (ESI): m/z calcd for $C_{17}H_{22}O_4NaS$ 345.1136; found 345.1140.

Ethyl *(E)*-2-(1-(4-methoxyphenyl)ethylidene)-1-(methylsulfonyl)cyclopentane-1-carboxylate (31b). Prepared in reaction of ethyl 2-(methylsulfonyl)oct-6-ynoate (98.5 mg, 0.400 mmol) and 4-bromoanisole (93.7 mg, 0.501 mmol) under general procedure (87.3 mg, 0.248 mmol, yield 62%). Product was isolated as oil after column chromatography on silica gel (15g, hex/AcOEt 8:2→7:3). ^{1}H NMR (400 MHz, $CDCl_3$) δ 7.15 – 7.10 (m, 2H), 6.87 – 6.82 (m, 2H), 4.37 – 4.22 (m, 2H), 3.79 (s, 3H), 3.18 (s, 3H), 2.69 – 2.60 (m, 1H), 2.51 – 2.41 (m, 1H), 2.38 – 2.30 (m, 2H), 2.04 (dd, J = 2.5, 1.0 Hz, 3H), 1.94 – 1.83 (m, 1H), 1.72 – 1.60 (m, 1H), 1.34 (t, J = 7.1 Hz, 3H); ^{13}C NMR (101 MHz, $CDCl_3$) δ 170.5, 158.4, 138.8, 136.7, 132.0, 128.5, 113.4, 77.95, 62.1, 55.2, 39.5, 37.9, 35.1, 24.7, 23.1, 14.0; IR (CH_2Cl_2): 2962, 2910, 2839, 1727, 1606, 1574, 1511, 1445, 1367, 1304, 1243, 1176, 1131, 1088, 953, 836, 742, 540 cm^{-1}; HRMS (ESI): m/z calcd for $C_{18}H_{24}O_5NaS$ 375.1242; found 375.1238.

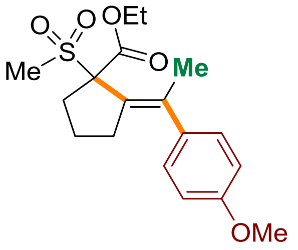

Ethyl *(E)*-2-(1-(4-cyanophenyl)ethylidene)-1-(methylsulfonyl)cyclopentane-1-carboxylate (31c). Prepared in reaction of ethyl 2-(methylsulfonyl)oct-6-ynoate (98.5 mg, 0.400 mmol) and 4-brombenzonitrile (92.1 mg, 0.506 mmol) under general procedure (101.4 mg, 0.292 mmol, yield 73%, E/Z 75:25). Product was isolated as oil after column chromatography on silica gel (15g, hex/AcOEt 8:2→1:1). ^{1}H NMR (400 MHz, $CDCl_3$) δ 7.6 – 7.6 (m, 2H), 7.3 – 7.3 (m, 2H), 4.4 – 4.2 (m, 2H), 3.2 (s, 3H), 2.7 – 2.6 (m, 1H), 2.4 – 2.3 (m, 2H), 2.2 – 2.1 (m, 1H), 2.0 (dd, J = 2.5, 1.1 Hz, 3H), 2.0 – 1.9 (m, 1H), 1.7 – 1.6 (m, 1H), 1.3 (t, J = 7.1 Hz, 3H); ^{13}C NMR (101 MHz, $CDCl_3$) δ 169.8, 149.1, 137.8, 133.8, 132.1, 128.2, 118.6, 110.7, 77.8, 62.3, 39.6, 38.1, 34.9, 24.6, 22.9, 14.0.Indicative signals of minor isomer: ^{1}H NMR (400 MHz, $CDCl_3$) δ 4.08 (q, J = 7.2 Hz, 2H), 2.00 (s, 3H), 1.22 (t, J = 7.1 Hz, 3H); ^{13}C NMR (101 MHz, $CDCl_3$) δ 170.9, 60.2, 20.9, 14.1; IR (CH_2Cl_2): 3042, 2978, 2940, 2876, 2227, 1728, 1604, 1503, 1446, 1401, 1366, 1305, 1238, 1132, 1020, 844, 743, 542, 491 cm^{-1}; HRMS (ESI): m/z calcd for $C_{18}H_{21}NO_4NaS$ 370.1089; found 370.1086.

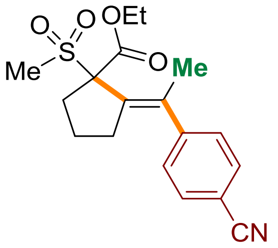

***(E)*-1,1'-(2-(1-phenylethylidene)cyclopentane-1,1-diyl)bis(ethan-1-one) (32a).** Prepared in reaction of 3-(hex-4-yn-1-yl)pentane-2,4-dione (72.1 mg, 0.400 mmol) and bromobenzene (78.9 mg, 0.503 mmol) under modified procedure (5 mol% cat.) (26.4 mg, 0.103 mmol, yield 27%). Product was isolated as oil after column chromatography on silica gel (15g, hex/AcOEt 9:1→8:2). ^{1}H NMR (400 MHz, $CDCl_3$) δ 7.36 – 7.30 (m, 2H), 7.26 – 7.21 (m, 1H), 7.19 – 7.16 (m, 2H), 2.34 – 2.25 (m, 10H), 1.85 (t, J = 2.0 Hz, 3H), 1.68 – 1.59 (m, 2H); ^{13}C NMR (101 MHz, $CDCl_3$) δ 206.6, 144.8, 136.6, 135.4, 128.3, 127.2, 126.6, 76.4, 36.3, 34.0, 27.3, 24.7, 22.7; IR (CH_2Cl_2): 3376, 3056, 2951, 1698, 1600, 1493, 1442, 1366, 1224, 1147, 1074, 1027, 921, 832, 767, 703, 628, 560 cm^{-1}; HRMS (ESI): m/z calcd for $C_{17}H_{20}O_2Na$ 279.1361; found 279.1356.

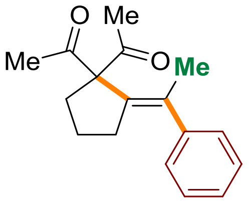

***(E)*-1,1'-(2-(1-(4-methoxyphenyl)ethylidene)cyclopentane-1,1-diyl)bis(ethan-1-one) (32b).** Prepared in reaction of 3-(hex-4-yn-1-yl)pentane-2,4-dione (72.1 mg, 0.400 mmol) and 4-bromoanisole (94.9 mg, 0.507 mmol) under modified procedure (5 mol% cat.) (32.4 mg, 0.113 mmol, yield 24%). Product was isolated as oil after column chromatography on silica gel (15g, hex/AcOEt 9:1→8:2). ^{1}H NMR (400 MHz, $CDCl_3$) δ 7.14 – 7.09 (m, 2H), 6.90 – 6.85 (m, 2H), 3.81 (s, 3H), 2.36 – 2.26 (m, 10H), 1.83 (t, J = 2.0 Hz, 3H), 1.64 (p, J = 7.0 Hz, 2H); ^{13}C NMR (101 MHz, $CDCl_3$) δ 206.7, 158.2, 137.1,

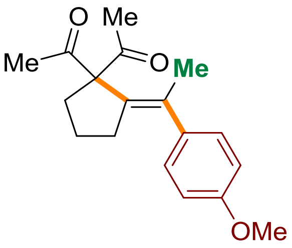

136.4, 134.9, 128.4, 113.6, 76.4, 55.2, 36.4, 34.1, 27.4, 24.7, 22.7; IR (CH_2Cl_2): 3374, 2999, 2955, 2928, 2838, 1696, 1607, 1574, 1510, 1442, 1354, 1291, 1244, 1175, 1128, 1032, 904, 834, 723, 573, 505 cm^{-1}; MS (EI): m/z (%) = 287(2), 286(7)[M^+], 245(10), 244(62), 243(87), 230(8), 229(51), 228(58), 227(87), 226(61), 225(20), 201(83), 186(25), 185(29), 173(16), 172(31), 135(16), 121(28), 115(16), 91(15), 77(12), 43(100); HRMS (EI): m/z calcd for $C_{18}H_{22}O_3$ 286.1569; found 286.1576.

_(E)_-4-(1-(2,2-diacetylcyclopentylidene)ethyl)benzonitrile (32c). Prepared in reaction of 3-(hex-4-yn-1-yl)pentane-2,4-dione (72.1 mg, 0.400 mmol) and 4-bromobenzonitrile (91.9 mg, 0.505 mmol) under modified procedure (run at 100°C, 5 mol% cat.) (60.9 mg, 0.220 mmol, yield 54%). Product was isolated as yellow solid after column chromatography on silica gel (15g, hex/AcOEt 9:1→7:3).^{1}H NMR (400 MHz, $CDCl_3$) δ 7.64 – 7.59 (m, 2H), 7.32 – 7.27 (m, 2H), 2.32 – 2.21 (m, 10H), 1.78 (t, J = 2.0 Hz, 3H), 1.65 (p, J = 7.0 Hz, 2H). ^{13}C NMR (101 MHz, $CDCl_3$) δ 205.8, 149.5, 137.9, 134.0, 132.2, 128.2, 118.7, 110.4, 76.4, 36.3, 34.1, 27.4, 24.7, 21.8. IR (CH_2Cl_2): 3625, 3405, 3374, 2959, 2877, 2227, 1929, 1713, 1697, 1604, 1503, 1433, 1355, 1177, 1153, 1129, 1087, 1018, 957, 905, 845, 739, 576 cm^{-1}; HRMS (ESI): m/z calcd for $C_{18}H_{19}NO_2Na$ 304.1313; found 304.1314.

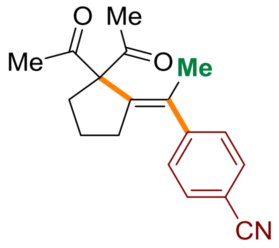

Dimethyl _(E)_-2-(1-phenylpropylidene)cyclopentane-1,1-dicarboxylate (33a). Prepared in reaction of dimethyl 2-(hept-4-yn-1-yl)malonate (90.5 mg, 0.400 mmol) and bromobenzene (78.5 mg, 0.500 mmol) under general procedure (70.3 mg, 0.233 mmol, yield 58%). Product was isolated as oil after column chromatography on silica gel (15g, hex/AcOEt 9:1). ^{1}H NMR (400 MHz, $CDCl_3$) δ 7.34 – 7.29 (m, 2H), 7.24 – 7.19 (m, 1H), 7.15 – 7.10 (m, 2H), 3.79 (s, 6H), 2.43 – 2.31 (m, 4H), 2.17 (tt, J = 7.3, 1.4 Hz, 2H), 1.61 (p, J = 7.0 Hz, 2H), 0.75 (t, J = 7.4 Hz, 3H). ^{13}C NMR (101 MHz, $CDCl_3$) δ 172.2, 143.0, 141.5, 135.0, 128.0, 128.0, 126.3, 63.2, 52.7, 39.4, 33.4, 28.7, 24.6, 11.6. IR (CH_2Cl_2): 3429, 3056, 2954, 2876, 1955, 1833, 1730, 1599, 1492, 1437, 1262, 1092, 1016, 918, 887, 803, 768, 737, 705, 524 cm^{-1}; MS (EI): m/z = 302(20)[M^+], 301(6), 284(10), 270(13), 243(28), 242(66), 228(13), 227(65), 225(20), 212(26), 211(100), 210(54), 209(30), 197(14), 183(65), 181(32), 165(23), 155(34), 141(25), 129(17), 105(56), 91(35), 77(26), 59(25), 57(19), 43(17); HRMS (EI): m/z calcd for $C_{18}H_{22}O_4$ 302.1518; found 302.1516.

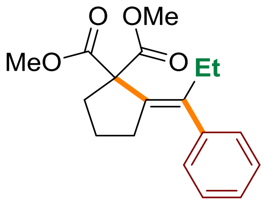

Dimethyl_(E)_-2-(1-(4-methoxyphenyl)propylidene)cyclopentane-1,1-dicarboxylate (33b). Prepared in reaction of dimethyl 2-(hept-4-yn-1-yl)malonate (90.5 mg, 0.400 mmol) and 4-bromoanisole (94.3 mg, 0.504 mmol) under general procedure (36.8 mg, 0.111 mmol, yield 28%). Product was isolated as oil after column chromatography on silica gel (15g, hex/AcOEt 8:2).^{1}H NMR (400 MHz, $CDCl_3$) δ 7.09 – 7.01 (m, 2H), 6.90 – 6.81 (m, 2H), 3.80 (s, 3H), 3.78 (s, 6H), 2.40 (t, J = 6.8 Hz, 2H), 2.33 (qt, J = 7.5, 1.4 Hz, 2H), 2.20 (tt, J = 7.2, 1.3 Hz, 2H), 1.61 (p, J = 7.1 Hz, 2H), 0.74 (t, J = 7.4 Hz, 3H). ^{13}C NMR (101 MHz, $CDCl_3$) δ 172.3, 158.0, 141.1, 135.4, 135.0, 129.1, 113.4, 63.2, 55.2, 52.6, 39.4, 33.5, 28.7, 24.6, 11.7. IR(CH_2Cl_2): 2953, 2874, 2838, 1730, 1607, 1574, 1509, 1492, 1434, 1371, 1244, 1174, 1139, 1087, 1034 cm^{-1}; MS (EI): m/z = 333(19), 332(61), 304(15), 303(57), 273(43), 272(44), 257(25), 243(53), 241(100), 240(35), 227(10), 213(58), 212(18), 198(14), 185(23), 171(14), 147(27), 121(23), 105(10), 91(10), 77(8), 59(17), 41(4); HRMS (EI): m/z calcd for $C_{19}H_{24}O_5$ 332.1624; found 332.1624.

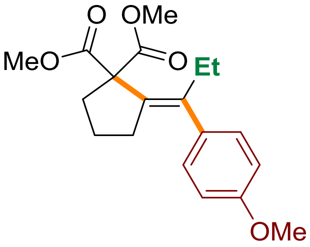

Dimethyl *(E)*-2-(1-(4-cyanophenyl)propylidene)cyclopentane-1,1-dicarboxylate (33c). Prepared in

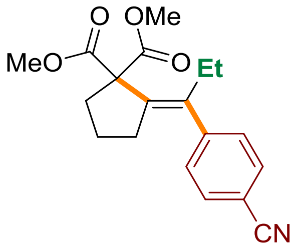

reaction of dimethyl 2-(hept-4-yn-1-yl)malonate (90.5 mg, 0.400 mmol) and 4-bromobenzonitrile (91.5 mg, 0.503 mmol) under general procedure (97.7 mg, 0.299 mmol, yield 75%). Product was isolated as oil after column chromatography on silica gel (15g, hex/AcOEt 7:3). ^{1}H NMR (400 MHz, $CDCl_3$) δ 7.62 – 7.58 (m, 2H), 7.25 – 7.21 (m, 2H), 3.77 (s, 6H), 2.40 – 2.31 (m, 4H), 2.13 – 2.07 (m, 2H), 1.61 (p, *J* = 7.0 Hz, 2H), 0.70 (t, *J* = 7.4 Hz, 3H). ^{13}C NMR (101 MHz, $CDCl_3$) δ 171.7, 148.0, 139.9, 136.4, 132.0, 128.9, 118.8, 110.3, 63.3, 52.7, 39.2, 33.4, 28.3, 24.6, 11.5. IR(CH_2Cl_2): 2954, 2876, 2842, 2227, 1933, 1731, 1604, 1502, 1434, 1400, 1374, 1265, 1188, 1186, 1140, 926, 842, 710, 570 cm^{-1}; MS (EI): m/z (%) = 328(8), 327(34)[M^+], 309(2), 295(19), 268(42), 267(77), 253(24), 252(79), 237(31), 236(100), 235(56), 222(10), 208(43), 207(19), 180(31), 166(22), 154(13), 130(12), 116(28), 113(17), 105(3), 91(5), 79(7), 59(26), 41(12); HRMS (EI): m/z calcd for $C_{19}H_{21}NO_4$ 327.1471; found 327.1465.

Dimethyl 2-(diphenylmethylene)cyclopentane-1,1-dicarboxylate (34a). Prepared in reaction of

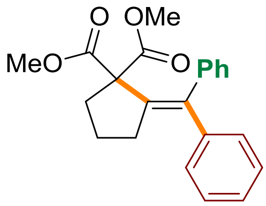

dimethyl 2-(5-phenylpent-4-yn-1-yl)malonate (109.7 mg, 0.400 mmol) and bromobenzene (79.1 mg, 0.504 mmol) under general procedure (92 mg, 0.260 mmol, yield 66%). Product was isolated as oil after column chromatography on silica gel (15g, hex/AcOEt 9:1) ^{1}H NMR (400 MHz, $CDCl_3$) δ 7.33 – 7.12 (m, 10H), 3.45 (s, 6H), 2.51 – 2.44 (m, 4H), 1.71 – 1.61 (m, 2H). ^{13}C NMR (101 MHz, $CDCl_3$) δ 171.3, 143.9, 140.9, 139.1, 138.9, 129.1, 128.3, 128.0, 127.6, 126.6, 126.4, 64.9, 52.3, 40.1, 32.6, 23.2. IR (CH_2Cl_2): 3455, 3054, 3020, 2952, 2875, 2841, 1954, 1890, 1730, 1598, 1577, 1492, 1433, 1310, 1265, 1192, 1167, 1133, 1065, 1033, 930, 892, 872, 799, 705, 522 cm^{-1}; HRMS (EI): m/z calcd for $C_{22}H_{22}O_4$ 350.1518; found 350.1506.

Dimethyl *(E)*-2-((4-methoxyphenyl)(phenyl)methylene)cyclopentane-1,1-dicarboxylate (34b).

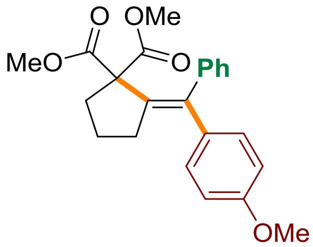

Prepared in reaction of dimethyl 2-(5-phenylpent-4-yn-1-yl)malonate (109.7 mg, 0.400 mmol) and 4-bromoanisole (94.9 mg, 0.507 mmol) under general procedure (49.2 mg, 0.129 mmol, yield 32%). Product was isolated as oil after column chromatography on silica gel (25g, hex/AcOEt 9:1→8:2) ^{1}H NMR (400 MHz, $CDCl_3$) δ 7.28 – 7.11 (m, 7H), 6.85 – 6.79 (m, 2H), 3.76 (s, 3H), 3.44 (s, 6H), 2.48 (dt, *J* = 18.6, 7.1 Hz, 4H), 1.70 – 1.61 (m, 2H). ^{13}C NMR (101 MHz, $CDCl_3$) δ 171.4, 158.1, 141.3, 138.7, 138.6, 136.4, 129.3, 129.1, 127.6, 126.6, 113.6, 64.9, 55.1, 52.3, 40.2, 32.8, 26.88, 23.3. IR (CH_2Cl_2): 3453, 2953, 2058, 1958, 1896, 1728, 1605, 1508, 1435, 1246, 1175, 1134, 1065, 1034, 930, 897, 833, 762, 704, 640, 584, 536, 500 cm^{-1}; HRMS (ESI): m/z calcd for $C_{23}H_{24}O_5Na$ 403.1521; found 403.1528

Dimethyl*(E)*-2-((4-cyanophenyl)(phenyl)methylene)cyclopentane-1,1-dicarboxylate (34c).

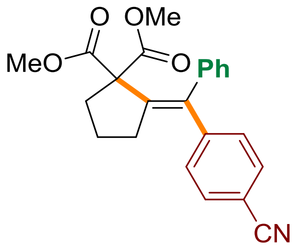

Prepared in reaction of dimethyl 2-(5-phenylpent-4-yn-1-yl)malonate (109.7 mg, 0.400 mmol) and 4-bromobenzonitrile (91.8 mg, 0.504 mmol) under general procedure (138.4 mg, 0.369 mmol, yield 92%). Product was isolated as white solid after column chromatography on silica gel (15g, hex/AcOEt 8:2). ^{1}H NMR (400 MHz, $CDCl_3$) δ 7.77 – 7.53 (m, 2H), 7.42 – 7.32 (m, 2H), 7.28 – 7.10 (m, 5H), 3.49 – 3.33 (m, 6H), 2.55 – 2.35 (m, 4H), 1.75 – 1.59 (m, 2H). ^{13}C NMR (101 MHz, $CDCl_3$) δ 170.9, 148.4, 140.5, 139.5, 137.5, 132.8, 132.2, 129.1, 129.0, 127.8, 127.2, 118.7, 110.3, 65.0, 52.4, 39.9, 32.6, 23.1. IR (CH_2Cl_2): 3055, 2952, 2877, 2842, 2227, 1954, 1730, 1604, 1492, 1434, 1400, 1312, 1264, 1193, 1168, 1135, 1108, 1065, 1030, 900, 822, 777, 762, 736, 703, 638, 556, 509, 459 cm^{-1};MS (EI): m/z (%) = 376(6), 375(22)[M^+], 343(14), 315(14), 298(12), 285(39), 284(100), 283(44), 257(31), 56(86), 255(17), 240(23), 229(15), 216(9), 190(5), 178(5), 153(8),

128(7), 113(4), 91(6), 77(2), 59(11), 41(1); HRMS (EI): m/z calcd for $C_{23}H_{21}NO_4$ 375.1471; found 375.1488.

Kinetic profiles for the reaction of 1 with electronically varied bromoarenes.

Table S7.

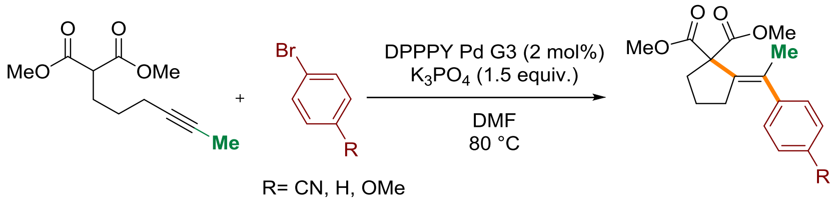

Entry	Time	Yield		
		R= CN	R= H	R= OMe
1	15 minutes	25 %	19 %	15 %
2	30 minutes	60 %	54 %	32 %
3	1 h	80 %	65 %	38 %
4	2 h	84 %	69 %	43 %
5	3 h	86 %	73 %	45 %
6	4 h	86 %	73 %	48 %
7	6 h	87 %	73 %	50 %

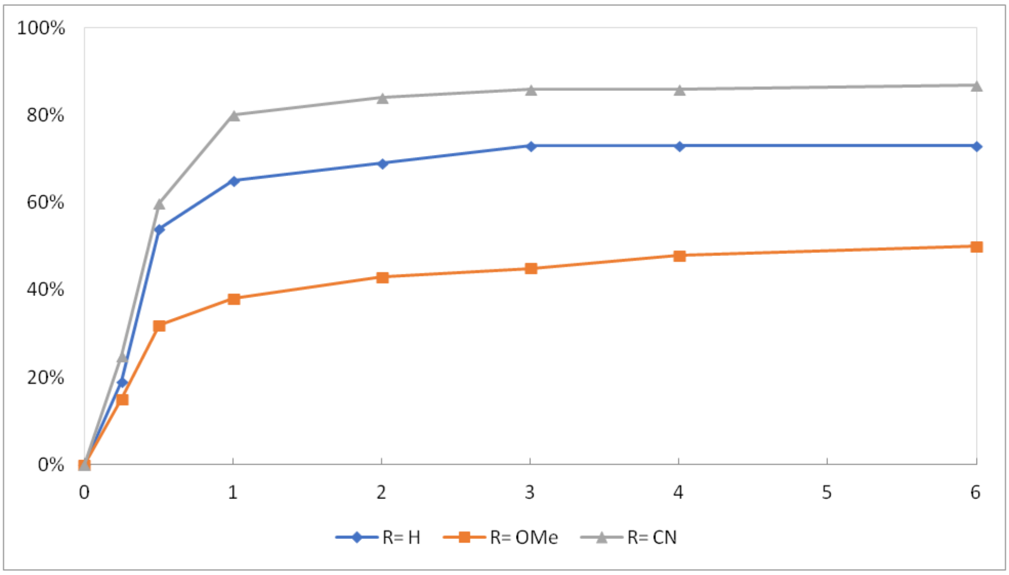

X-Ray Diffraction Data of Compound 4

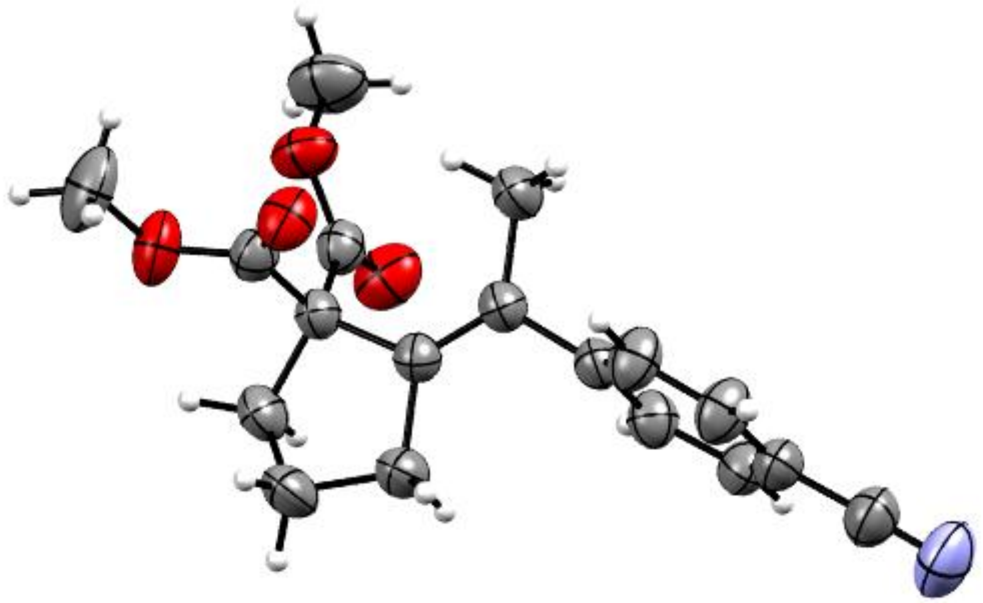

Crystal colour	colourless
Crystal description	prism
Crystal dimensions [mm]	0.508 x 0.368 x 0.198
Formula	$C_{18}H_{19}NO_4$
Temperature [K]	296(2)
Radiation type	CuK\α
Crystal system	monoclinic
Space group	C2/c
Unit cell parameters	
a [Å]	12.581(4)
b [Å]	10.168(4)
c [Å]	26.637(9)
α [°]	90
β [°]	100.811(18)
γ [°]	90
V [Å3]	3347(2)
F(000)	1328
Dx [g cm-3]	1.244
Reflections used in refinement	3079
Parameters refined	251
Restraints	0
Goodness of fit	0.926

NMR signal assignment for compound 2

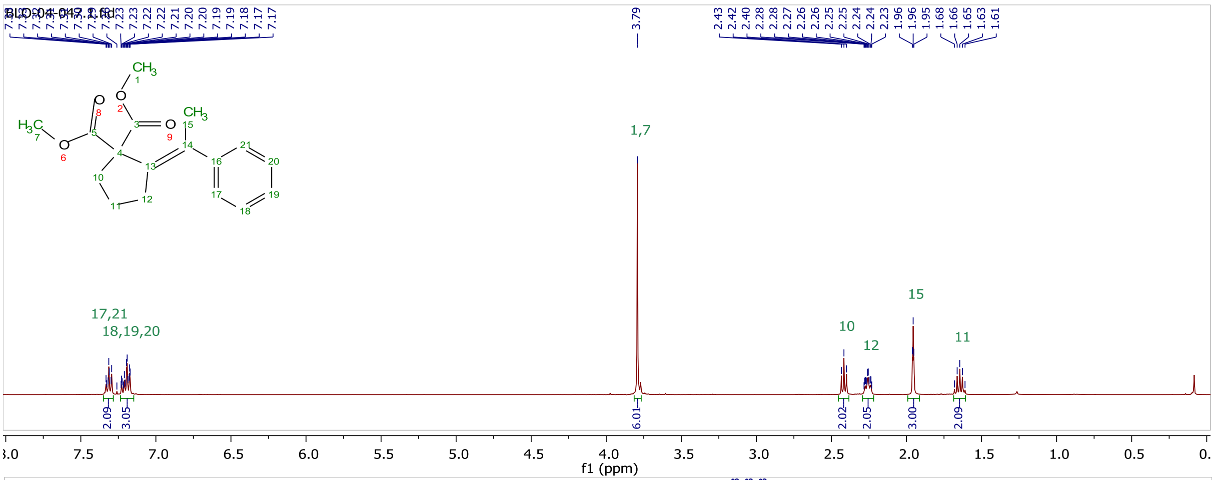

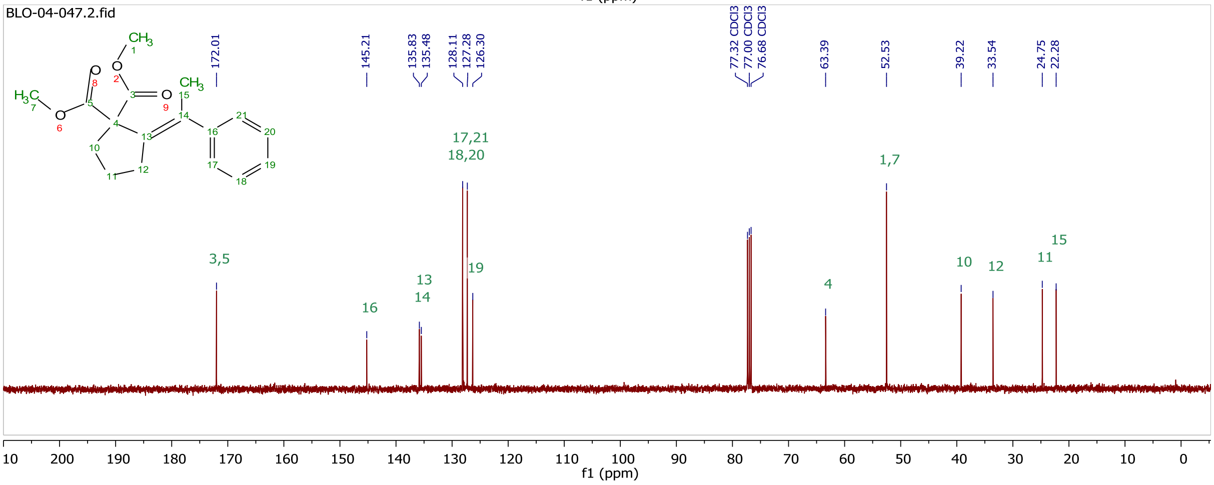

Computational methods

All calculations were performed using Gaussian 09 package. Structures of minima and transition states were optimized employing B3LYP functional with the D3 version of Grimme's empirical dispersion correction and solvation (DMF) with SMD model. LANL2DZ basis set was used for Pd, and the 6-31G(d) basis set for the other atoms (later on denoted as BS1). Frequency calculations were then performed at the same level of theory to confirm the nature of stationary points and provide corrections to thermodynamic functions. Single point energies were calculated at M06 level of theory using SDD basis set for Pd and I, and the 6-311++g(d,p) basis set for the other atoms (later on denoted as BS2) and SMD solvation model (DMF). Various conformers of intermediates and transition states were investigated and only the lowest energy conformers are shown in the work. Structures were visualized using: CYLview, 1.0b; Legault, C. Y., Université de Sherbrooke, 2009 (http://www.cylview.org)

Optimized geometries, energies and corrections to thermodynamic functions.

PhBr

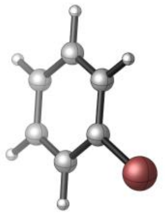

E (SMD(DMF)/B3LYP-D3/BS1) = -2803.370091
E (SMD (DMF)/M06/BS2//SMD(DMF)/B3LYP-D3/BS1) = -2805.541823

Zero-point correction=	0.090810
Thermal correction to Energy=	0.096509
Thermal correction to Enthalpy=	0.097453
Thermal correction to Gibbs Free Energy=	0.059928

Charge = 0 Multiplicity = 1

C	-2.18408200	-1.20913000	0.00000100
C	-0.78686200	-1.21822400	0.00001600
C	-0.10898700	-0.00001500	-0.00001800
C	-0.78685600	1.21821900	-0.00000300
C	-2.18405400	1.20914700	0.00001900
C	-2.88394900	0.00000300	-0.00001400
H	-2.72110100	-2.15366700	-0.00000300
H	-0.23918100	-2.15463000	0.00001400
H	-0.23913200	2.15460100	-0.00000600
H	-2.72109400	2.15367100	0.00001900
H	-3.97021500	0.00002800	-0.00002500
Br	1.81427000	0.00000000	0.00000000

DPPPyPd(0)

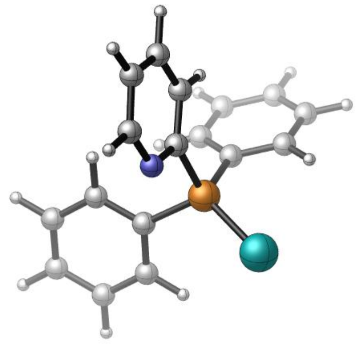

E (SMD(DMF)/B3LYP-D3/BS1) = -1179.148548
E (SMD(DMF)/M06/BS2//SMD(DMF)/B3LYP-D3/BS1) = -1179.903588

Zero-point correction=	0.263685
Thermal correction to Energy=	0.281437
Thermal correction to Enthalpy=	0.282382
Thermal correction to Gibbs Free Energy=	0.214231

Charge = 0 Multiplicity = 1

Pd	-0.00937400	0.17025900	-2.58148300
P	-0.02247200	-0.00663500	-0.38320300
C	0.24629200	1.60380200	0.50510300
C	-0.62261600	2.12646900	1.46930900
C	1.65505400	3.41672300	0.68643900
C	-0.31542200	3.36051200	2.04760400
H	-1.51328300	1.58446900	1.76656500
C	0.84619100	4.02127900	1.65317200
H	2.57102800	3.90129500	0.35332000
H	-0.97320200	3.79256200	2.79688500
H	1.12379100	4.98162000	2.07642300
N	1.36689800	2.24208600	0.11479400
C	1.30064500	-1.05871400	0.36700600
C	1.72273100	-2.19687500	-0.33868900
C	1.88287200	-0.77738400	1.61412300
C	2.69577900	-3.04397600	0.19492200
H	1.28998400	-2.41395100	-1.31271600
C	2.86357700	-1.62049000	2.14227800
H	1.57345700	0.09687300	2.17913100
C	3.27040600	-2.75570700	1.43607300
H	3.01053500	-3.92252800	-0.36213200
H	3.30857200	-1.38932200	3.10662100
H	4.03447100	-3.40934000	1.84851100
C	-1.57192200	-0.64781500	0.39995700
C	-2.78983200	-0.41561000	-0.25991500
C	-1.58515300	-1.32164300	1.63222500
C	-3.99609500	-0.83784100	0.30251100
H	-2.78851700	0.09625800	-1.21979900
C	-2.79145500	-1.75242700	2.18907500
H	-0.65698800	-1.51414300	2.16141700
C	-3.99880600	-1.50989400	1.52782500

H	-4.93048200	-0.64848800	-0.21933700
H	-2.78699200	-2.27752000	3.14073600
H	-4.93581500	-1.84724600	1.96297000

DPPPyPd(0)·PhBr - I

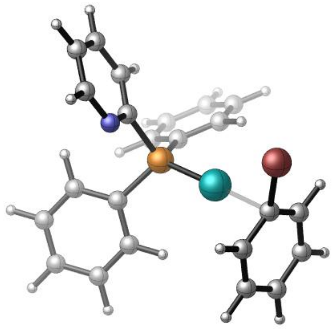

E (SMD(DMF)/B3LYP-D3/BS1) = -3982.241344
E (SMD(DMF)/M06/BS2//SMD(DMF)/B3LYP-D3/BS1) = -3985.477281

Zero-point correction=	0.354382
Thermal correction to Energy=	0.379267
Thermal correction to Enthalpy=	0.380211
Thermal correction to Gibbs Free Energy=	0.292169

Charge = 0 Multiplicity = 1

Pd	1.13395100	0.07167000	0.03682200
P	-1.13910500	-0.00227700	0.03260600
C	-1.83929100	-1.62225800	-0.53756200
C	-2.76030200	-2.38480100	0.18785600
C	-1.78046300	-3.17259300	-2.23853500
C	-3.18971900	-3.60128100	-0.34858300
H	-3.13786300	-2.03728400	1.14309700
C	-2.69395100	-4.00590500	-1.58623900
H	-1.37206900	-3.45414800	-3.20725000
H	-3.90347900	-4.21659900	0.19231500
H	-3.00058600	-4.94286800	-2.04064800
C	3.35798400	0.17365400	0.01564600
C	3.30546600	0.93285600	-1.17794200
C	3.35549600	0.81511900	1.27751800
C	3.29422100	2.33247500	-1.09206000
H	3.35489900	0.43861500	-2.14193800
C	3.34728600	2.21598500	1.32751100
H	3.43782200	0.22965000	2.18679000
C	3.33760500	2.97151900	0.15084200
H	3.27223400	2.91412500	-2.00922900
H	3.36683800	2.70704600	2.29631800
H	3.35310600	4.05619200	0.20259600
N	-1.35157700	-2.01100400	-1.73171400
C	-1.97126300	1.18668300	-1.10716100
C	-1.33279200	2.40960100	-1.36830500
C	-3.21011800	0.92021800	-1.71353000

C	-1.92665100	3.35393800	-2.20812900
H	-0.36413500	2.61683600	-0.91880300
C	-3.79860400	1.86291700	-2.55954200
H	-3.71893900	-0.02156900	-1.52845200
C	-3.15990300	3.08140500	-2.80667200
H	-1.42156900	4.29667000	-2.40164000
H	-4.75561900	1.64414400	-3.02600000
H	-3.61866500	3.81245900	-3.46712600
C	-1.97280800	0.27123800	1.65674900
C	-1.26382500	-0.07306800	2.81939500
C	-3.27772300	0.77533400	1.77908800
C	-1.85070200	0.07071300	4.07821100
H	-0.24716900	-0.45114900	2.73456300
C	-3.86020700	0.92699900	3.03935500
H	-3.84240700	1.05273600	0.89410400
C	-3.15005200	0.57290300	4.19027100
H	-1.29101400	-0.20154800	4.96921400
H	-4.86956200	1.32170600	3.12130600
H	-3.60562700	0.69288500	5.16967600
Br	3.85690200	-1.70644100	-0.08282600

DPPPyPd(PhBr) – TS1

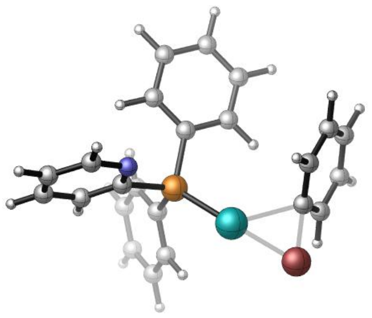

E (SMD(DMF)/B3LYP-D3/BS1) = -3982.230928
E (SMD(DMF)/M06/BS2//SMD(DMF)/B3LYP-D3/BS1) = -3985.465993

Zero-point correction=	0.353894
Thermal correction to Energy=	0.378071
Thermal correction to Enthalpy=	0.379015
Thermal correction to Gibbs Free Energy=	0.295525

Charge = 0 Multiplicity = 1

Pd	-1.04413900	-1.00602700	0.00269500
P	1.08410400	-0.11022600	0.03129800
C	2.27865000	-0.95996700	-1.10049500
C	3.52990000	-1.44773000	-0.71016800
C	2.58098100	-1.69083500	-3.26244300
C	4.32193300	-2.09066300	-1.66491500
H	3.88143200	-1.32497500	0.30813700
C	3.84325700	-2.21462900	-2.96752000
H	2.17323700	-1.76831100	-4.26848100
H	5.29781200	-2.48263400	-1.39185600

H	4.42530600	-2.70633700	-3.74067400
C	-2.98337000	0.10809200	0.03972000
C	-3.11663400	0.83321100	-1.15340400
C	-3.15636000	0.71112800	1.29417000
C	-3.33242700	2.21052200	-1.07339800
H	-3.03576300	0.33705100	-2.11390900
C	-3.37268900	2.09044200	1.34406400
H	-3.10719700	0.12177200	2.20291800
C	-3.46143200	2.84267800	0.16777600
H	-3.40418800	2.78722500	-1.99164100
H	-3.48001300	2.57267800	2.31211300
H	-3.64032000	3.91271600	0.21786200
N	1.80628500	-1.08433600	-2.35614000
C	1.95489700	-0.07175300	1.65406800
C	1.56440700	-0.99323500	2.63839000
C	2.99878300	0.82630700	1.93279800
C	2.21136400	-1.02491700	3.87592800
H	0.74954200	-1.68390000	2.43270700
C	3.64090700	0.79620900	3.17188200
H	3.31151200	1.54939700	1.18518700
C	3.24949800	-0.12931800	4.14453600
H	1.89975300	-1.74297400	4.62976300
H	4.44603000	1.49642100	3.37835700
H	3.74945800	-0.14846800	5.10927600
C	1.16690700	1.63566800	-0.55239700
C	2.23242300	2.14006900	-1.31501400
C	0.11184600	2.49148200	-0.19849800
C	2.23914100	3.47920600	-1.71326700
H	3.05777500	1.49473100	-1.60036500
C	0.12529600	3.83060900	-0.59151900
H	-0.72429400	2.10886100	0.37886300
C	1.18785000	4.32657700	-1.35226700
H	3.06697100	3.85900500	-2.30633100
H	-0.70029100	4.47983300	-0.31291500
H	1.19492500	5.36688100	-1.66654200
Br	-3.39793100	-1.96865500	-0.07174500

DPPPyPdPhBr - II

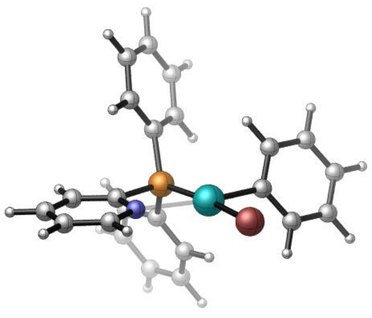

E (SMD(DMF)/B3LYP-D3/BS1) = -3982.281347
E (SMD(DMF)/M06/BS2//SMD(DMF)/B3LYP-D3/BS1) = -3985.506557

Zero-point correction=	0.355597
Thermal correction to Energy=	0.380925
Thermal correction to Enthalpy=	0.381870
Thermal correction to Gibbs Free Energy=	0.294452

Charge = 0 Multiplicity = 1

Pd	1.25474100	0.39681300	-0.04094300
P	-1.03797300	0.18959300	0.05518200
C	-1.11766700	2.02565100	0.14640100
C	-2.19742400	2.89281900	0.26062700
C	0.42219600	3.76427500	0.15341600
C	-1.92780700	4.26413400	0.32000300
H	-3.21138700	2.50878300	0.30060000
C	-0.60489200	4.70461800	0.26551600
H	1.46728700	4.05497400	0.10924900
H	-2.74162200	4.97780500	0.40792300
H	-0.36484400	5.76191500	0.30971100
C	1.64113200	-1.56202100	-0.12366200
C	1.15543100	-2.34083300	-1.18518500
C	2.37451300	-2.18703200	0.89653200
C	1.40455800	-3.71894400	-1.22821800
H	0.56805200	-1.88575600	-1.97878200
C	2.61193900	-3.56615200	0.85587200
H	2.77220100	-1.60158400	1.72044400
C	2.12982500	-4.33708100	-0.20660900
H	1.02001000	-4.30705300	-2.05891400
H	3.17994000	-4.03628700	1.65604100
H	2.31745500	-5.40753900	-0.23672300
N	0.16790900	2.45301700	0.09492800
C	-1.87961400	-0.50182000	1.51265100
C	-1.10177800	-1.01240700	2.56229800
C	-3.28155400	-0.49892100	1.61304800
C	-1.72293500	-1.51121600	3.70958000
H	-0.01887000	-1.02586600	2.47621700
C	-3.89413600	-0.99641000	2.76265200
H	-3.88797100	-0.11815000	0.79623300
C	-3.11610100	-1.50165500	3.81038700
H	-1.11859500	-1.90987300	4.51959100
H	-4.97777400	-0.99247700	2.84078100
H	-3.59808400	-1.89171200	4.70274500
C	-1.98874800	-0.34493400	-1.40254200
C	-2.20152500	0.52628700	-2.48279100
C	-2.41070700	-1.68221000	-1.49299500
C	-2.84700300	0.06674800	-3.63170800
H	-1.87236900	1.55997400	-2.42957300
C	-3.05303900	-2.13427200	-2.64648700
H	-2.24415500	-2.36714100	-0.66671000
C	-3.27357500	-1.26173300	-3.71584300
H	-3.01767100	0.74881000	-4.46003600
H	-3.38083400	-3.16826600	-2.70794900
H	-3.77523600	-1.61604700	-4.61214000
Br	3.68097300	1.08359300	-0.13757700

Compound 38

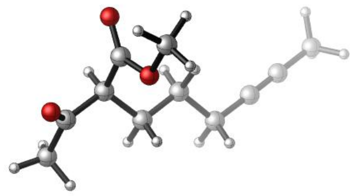

E (SMD(DMF)/B3LYP-D3/BS1) = -654.472571
E (SMD(DMF)/M06/BS2//SMD(DMF)/B3LYP-D3/BS1) = -654.199144

Zero-point correction=	0.252125
Thermal correction to Energy=	0.269089
Thermal correction to Enthalpy=	0.270033
Thermal correction to Gibbs Free Energy=	0.205080

Charge = 0 Multiplicity = 1

C	2.26733800	-1.10422000	0.69527900
H	2.25646900	-2.19662100	0.57449100
H	2.09547100	-0.91328700	1.76400100
C	3.57975200	-0.57932700	0.30706100
C	4.65226000	-0.12917000	-0.03283400
C	-1.41825800	-0.49614800	-0.52120900
C	-0.24581500	-1.05470400	0.31570600
H	-0.41870000	-0.83157300	1.37486900
H	-0.23060000	-2.14520800	0.21219700
C	1.11057000	-0.49155500	-0.12062100
H	1.12617800	0.59815900	0.00411900
H	1.27045000	-0.69402700	-1.18742800
C	-2.78110600	-1.07285200	-0.10802200
C	-1.49531100	1.02325300	-0.50482900
O	-3.73483500	-0.34511000	0.11181500
O	-1.63878400	1.71811100	-1.49021900
C	-2.88396900	-2.57709900	-0.01035100
H	-3.93585000	-2.87265600	0.00636100
H	-2.36402300	-3.06794600	-0.84038400
H	-2.40746400	-2.91582100	0.91871600
O	-1.38523700	1.50900700	0.74390600
C	-1.47883300	2.94174500	0.86899100
H	-1.36252000	3.15232300	1.93292900
H	-0.68563000	3.43135900	0.29674300
H	-2.45253900	3.29383800	0.51649300
C	5.95011200	0.41024100	-0.43687700
H	6.45985100	0.89491000	0.40530400
H	6.61042100	-0.38050200	-0.81470200
H	5.83909700	1.15797300	-1.23226300
H	-1.27019300	-0.78797800	-1.56923700

Dimethyl 2-(pent-4-yn-1-yl)malonate

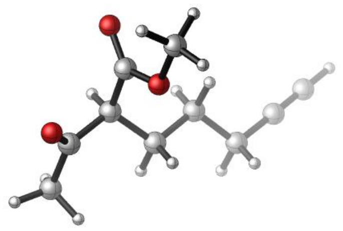

E (SMD(DMF)/B3LYP-D3/BS1) = -615.144488
E (SMD(DMF)/M06/BS2//SMD(DMF)/B3LYP-D3/BS1) = -614.897401

Zero-point correction=	0.223474
Thermal correction to Energy=	0.238599
Thermal correction to Enthalpy=	0.239543
Thermal correction to Gibbs Free Energy=	0.179781

Charge = 0 Multiplicity = 1

C	-2.86386600	-0.83470900	-0.54814000
H	-2.92976000	-1.92838600	-0.46728900
H	-2.73467800	-0.61088200	-1.61595500
C	-4.11462700	-0.23865100	-0.07577500
C	-5.13682900	0.26360100	0.33222700
C	0.90541400	-0.53177600	0.50637000
C	-0.34290500	-0.98249900	-0.28398200
H	-0.20514400	-0.74446700	-1.34497500
H	-0.43378600	-2.07123000	-0.20440900
C	-1.63133500	-0.33480200	0.23288800
H	-1.57087300	0.75645500	0.14168200
H	-1.75895200	-0.56174300	1.29888100
C	2.19838500	-1.20186900	0.01568900
C	1.10139000	0.97703900	0.51387600
O	3.18893800	-0.54333600	-0.25357600
O	1.36588300	1.63319500	1.50068700
C	2.18499400	-2.70856800	-0.09615100
H	3.20993700	-3.08011200	-0.17070200
H	1.67461600	-3.16829400	0.75730900
H	1.63675500	-3.00230300	-1.00063700
O	0.94907300	1.50124900	-0.71465100
C	1.15462800	2.92419300	-0.81754500
H	0.98353400	3.17095600	-1.86615000
H	0.44732000	3.46310100	-0.18069400
H	2.17674800	3.18478200	-0.52808800
H	0.78433500	-0.83444500	1.55472500
H	-6.04269700	0.70595400	0.68987000

Compound 1

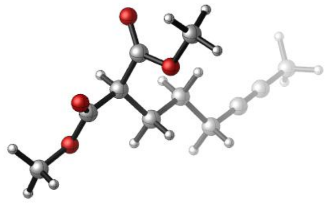

E (SMD(DMF)/B3LYP-D3/BS1) = -729.70421
E (SMD(DMF)/M06/BS2//SMD(DMF)/B3LYP-D3/BS1) = -729.422096

Zero-point correction=	0.257885
Thermal correction to Energy=	0.275882
Thermal correction to Enthalpy=	0.276826
Thermal correction to Gibbs Free Energy=	0.208356

Charge = 0 Multiplicity = 1

C	-2.42267000	-1.18448300	-0.69638600
H	-2.27907600	-2.26899100	-0.59030900
H	-2.26373200	-0.95794600	-1.76012600
C	-3.79367300	-0.83009900	-0.31787700
C	-4.91687000	-0.51972900	0.01451300
C	1.14511400	-0.13891400	0.56403500
C	0.05938300	-0.84056600	-0.28096900
H	0.21561800	-0.59876700	-1.33845900
H	0.19067200	-1.92181200	-0.17190200
C	-1.35866600	-0.44727700	0.14219000
H	-1.50305200	0.63434700	0.02797700
H	-1.50708100	-0.67924600	1.20460100
C	2.55582100	-0.56097200	0.16206100
C	1.06427000	1.38231400	0.51048000
O	3.45712400	0.20100500	-0.12681200
O	1.13488100	2.10679300	1.48165200
O	0.89934600	1.82365700	-0.74658600
C	0.83876700	3.25596500	-0.90527300
H	0.69207200	3.42675200	-1.97242000
H	0.00240900	3.67070800	-0.33564200
H	1.77263400	3.71685700	-0.57108500
C	-6.27590100	-0.15074100	0.40884700
H	-6.80110600	0.36443800	-0.40524500
H	-6.86659400	-1.03537300	0.67820600
H	-6.26922200	0.52063100	1.27675200
H	1.01664700	-0.41807200	1.61614500
O	2.68341200	-1.89655000	0.19061800
C	3.98783100	-2.41156800	-0.15687300
H	3.90295700	-3.49590300	-0.07798500
H	4.25352900	-2.12334800	-1.17768800
H	4.74478200	-2.03709800	0.53770800

IIIa

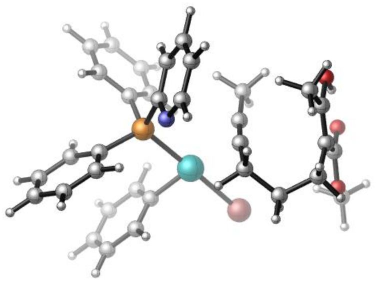

E (SMD(DMF)/B3LYP-D3/BS1) = -4636.768315
E (SMD(DMF)/M06/BS2//SMD(DMF)/B3LYP-D3/BS1) = -4639.735313

Zero-point correction=	0.611152
Thermal correction to Energy=	0.653717
Thermal correction to Enthalpy=	0.654661
Thermal correction to Gibbs Free Energy=	0.530324

Charge = 0 Multiplicity = 1

C	2.13217700	-0.32291000	-1.97463600
H	2.38276800	-1.30373900	-2.40006900
H	1.22435000	0.00833700	-2.49143600
C	1.82458900	-0.50065600	-0.54724500
C	1.67738000	-0.69987500	0.64777500
Pd	-0.23519600	0.88093400	0.07054000
P	-1.71754300	-0.91229200	-0.01149500
C	-0.84210400	-2.44799700	-0.56178300
C	-0.53530700	-3.49349800	0.31615900
C	0.34355300	-3.42723900	-2.27280500
C	0.26517000	-4.53790800	-0.15133100
H	-0.89771000	-3.49229000	1.33720200
C	0.71586800	-4.50856900	-1.47001900
H	0.67220000	-3.36665800	-3.30798000
H	0.53071000	-5.35908400	0.50828400
H	1.34181300	-5.29867500	-1.87251600
C	-1.86571900	2.05962400	-0.01629700
C	-2.29284700	2.52921400	-1.26659900
C	-2.59856600	2.39948500	1.12732300
C	-3.44214600	3.31988600	-1.37092200
H	-1.74680500	2.26602100	-2.16889600
C	-3.75050500	3.19058000	1.01954600
H	-2.29279100	2.04094200	2.10586100
C	-4.17779800	3.65078300	-0.22820900
H	-3.76577900	3.66984000	-2.34881400
H	-4.31459400	3.43984900	1.91594500
H	-5.07589800	4.25775700	-0.31100700
C	4.88367200	-0.34113000	-0.52114600
C	4.68548000	0.12052500	-1.95081100
H	5.41605600	0.90365400	-2.18424400
H	4.88685200	-0.70206300	-2.64326900

C	3.27604700	0.67970000	-2.24191800
H	3.23251300	0.96346900	-3.30149000
H	3.09671900	1.58168300	-1.64940900
C	4.82363100	-1.65558800	-0.12658000
C	4.96613300	0.67471100	0.52488800
O	4.90028500	-2.03136300	1.16114400
H	4.96281500	-1.18572200	1.69428200
O	5.00088300	0.44521700	1.74870600
C	4.61627700	-2.84107500	-1.01970200
H	3.58618600	-3.20233300	-0.90005000
H	4.78403400	-2.62635300	-2.07454700
H	5.28394000	-3.65218700	-0.70864900
O	5.00445600	1.93012100	0.05607600
C	4.95416900	2.98251700	1.03680000
H	5.01768500	3.91038200	0.46663800
H	4.00701700	2.94256900	1.57938100
H	5.79468000	2.90641000	1.73256000
N	-0.40466200	-2.40999600	-1.83152900
C	-3.16985200	-0.69798200	-1.11659500
C	-4.38322500	-0.23064100	-0.58540000
C	-3.03679200	-0.83406800	-2.50795800
C	-5.44760000	0.08275300	-1.43200700
H	-4.49874500	-0.09181400	0.48388500
C	-4.10923800	-0.52811600	-3.34790900
H	-2.09769500	-1.17428800	-2.92965600
C	-5.31549800	-0.06684200	-2.81469000
H	-6.37828600	0.44893700	-1.00725200
H	-3.99626200	-0.64427600	-4.42259800
H	-6.14480700	0.17993300	-3.47211900
C	-2.40812500	-1.33988800	1.62858400
C	-1.89543300	-0.71705700	2.77594800
C	-3.40920400	-2.31803900	1.76220700
C	-2.37527500	-1.06626800	4.04063900
H	-1.12008400	0.03714600	2.67792600
C	-3.88699700	-2.66269800	3.02625300
H	-3.81442600	-2.80712000	0.88093800
C	-3.37050800	-2.03761300	4.16648200
H	-1.97175300	-0.57774700	4.92317900
H	-4.66110900	-3.41911200	3.12172000
H	-3.74505200	-2.30837800	5.14996100
Br	1.26545500	2.92008200	0.30336900
C	1.67310200	-1.05215600	2.06779500
H	0.76590400	-1.60346300	2.33777400
H	2.54151800	-1.68151400	2.29163800
H	1.72585700	-0.15516900	2.69476800

IIIb

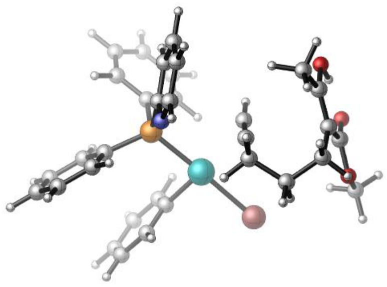

E (SMD(DMF)/B3LYP-D3/BS1) = -4597.436349
E (SMD(DMF)/M06/BS2//SMD(DMF)/B3LYP-D3/BS1) = -4600.429489

Zero-point correction=	0.581617
Thermal correction to Energy=	0.622813
Thermal correction to Enthalpy=	0.623757
Thermal correction to Gibbs Free Energy=	0.500640

Charge = 0 Multiplicity = 1

C	-2.34016300	0.38637600	-1.93095100
H	-2.58418600	1.39008300	-2.30261500
H	-1.47145100	0.04903200	-2.50751300
C	-1.93908200	0.50231300	-0.52437500
C	-1.63787100	0.62289100	0.65077000
Pd	0.21367800	-0.90183000	-0.00597500
P	1.65552800	0.92629500	0.04204600
C	0.80488600	2.41158200	-0.66021200
C	0.47671000	3.53755900	0.10102600
C	-0.29063000	3.23722900	-2.50715100
C	-0.28334800	4.54419800	-0.49875900
H	0.79238400	3.62669000	1.13379100
C	-0.67650100	4.39566800	-1.82772700
H	-0.57739100	3.08209400	-3.54487700
H	-0.56307700	5.42777100	0.06788300
H	-1.27039100	5.15229800	-2.33055400
C	1.86914900	-2.04108900	-0.05247300
C	2.39948800	-2.46128400	-1.27956200
C	2.52469000	-2.39192500	1.13432500
C	3.57538700	-3.21854400	-1.31752600
H	1.91346500	-2.18658500	-2.21207500
C	3.70217500	-3.15041300	1.09260400
H	2.13922100	-2.06200200	2.09545400
C	4.23238300	-3.56386900	-0.13204500
H	3.98068800	-3.53229800	-2.27709000
H	4.20630400	-3.40930000	2.02136800
H	5.15048400	-4.14517600	-0.16384100
C	-4.92907900	0.41927200	-0.27276100
C	-4.89150000	0.00256000	-1.72872800
H	-5.65846600	-0.75757100	-1.91610100
H	-5.13640400	0.85281400	-2.37166300

C	-3.52664800	-0.57400100	-2.16483400
H	-3.56891600	-0.79688400	-3.23849300
H	-3.32463500	-1.51146800	-1.63870200
C	-4.79372800	1.71932200	0.15378700
C	-4.90282100	-0.63026500	0.74220400
O	-4.68837000	2.04970700	1.44864000
H	-4.68846400	1.18717600	1.95737500
O	-4.77651700	-0.44313600	1.96683200
C	-4.68419600	2.93132900	-0.72150000
H	-3.63876100	3.26721500	-0.73887600
H	-5.00706900	2.75861300	-1.74767900
H	-5.27976300	3.74393200	-0.29105600
O	-5.02872600	-1.86783900	0.24117400
C	-4.87098900	-2.95574200	1.17084400
H	-5.04468700	-3.86074100	0.58688600
H	-3.85417200	-2.95995200	1.57044400
H	-5.59953500	-2.88290400	1.98321800
N	0.42021700	2.25618400	-1.93906400
C	3.24004600	0.76056800	-0.87335100
C	4.38208700	0.29382800	-0.20178500
C	3.28989300	0.94866700	-2.26364400
C	5.55455900	0.02739100	-0.90999000
H	4.35793400	0.12041900	0.86843900
C	4.46912900	0.68840300	-2.96450400
H	2.40852100	1.28757000	-2.79702200
C	5.60273700	0.22510600	-2.29226900
H	6.42847500	-0.33933800	-0.37850900
H	4.49699500	0.84324300	-4.03984000
H	6.51616600	0.01478500	-2.84210800
C	2.12407800	1.39581200	1.74644000
C	1.48536100	0.76952000	2.82733700
C	3.08696100	2.39025500	1.99313500
C	1.80303000	1.13278000	4.13821700
H	0.74381200	-0.00251300	2.63998600
C	3.40081900	2.75000000	3.30355600
H	3.59000500	2.87944500	1.16395000
C	2.75984600	2.12169400	4.37682200
H	1.30521800	0.64085000	4.96921600
H	4.14573600	3.51931300	3.48705500
H	3.00860600	2.40266900	5.39661600
Br	-1.26116900	-2.97452500	-0.00498700
H	-1.53163800	0.83257500	1.69442800

IIIc

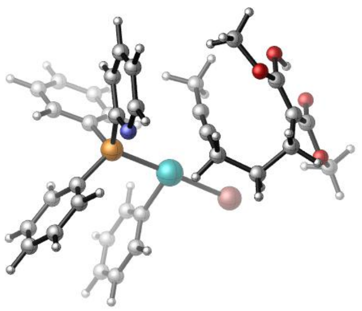

E (SMD(DMF)/B3LYP-D3/BS1) = -4711.984298
E (SMD(DMF)/M06/BS2//SMD(DMF)/B3LYP-D3/BS1) = -4714.944193

Zero-point correction=	0.615876
Thermal correction to Energy=	0.659297
Thermal correction to Enthalpy=	0.660241
Thermal correction to Gibbs Free Energy=	0.535071

Charge = 0 Multiplicity = 1

C	-2.02986300	0.36951200	-1.94627300
H	-2.34569800	1.34374300	-2.34130100
H	-1.09364100	0.12219800	-2.45955100
C	-1.74001800	0.53224700	-0.51317600
C	-1.57197000	0.73735800	0.67849900
Pd	0.30179200	-0.88735300	0.09648800
P	1.80988100	0.88480700	-0.01626000
C	0.95850100	2.43690400	-0.55964800
C	0.68404300	3.49076800	0.31945400
C	-0.21457100	3.44462200	-2.26304600
C	-0.09037200	4.55619900	-0.14447000
H	1.05311600	3.48119100	1.33802900
C	-0.55054700	4.53716200	-1.46006900
H	-0.55317600	3.39049000	-3.29535300
H	-0.32923600	5.38518700	0.51557700
H	-1.15735800	5.34349300	-1.85942800
C	1.91430000	-2.08897800	-0.00118100
C	2.32383000	-2.56764700	-1.25383300
C	2.65167900	-2.43617700	1.13716400
C	3.46089000	-3.37486600	-1.36575600
H	1.77455700	-2.29809000	-2.15223700
C	3.79150600	-3.24360100	1.02161200
H	2.35878100	-2.07177000	2.11739400
C	4.20161800	-3.71297100	-0.22847300
H	3.77125900	-3.73162100	-2.34546600
H	4.35957800	-3.49851200	1.91388600
H	5.09035800	-4.33273400	-0.31726200
C	-4.81716400	0.04515200	-0.52798500
C	-4.54655400	-0.25779700	-1.98454300
H	-5.22166100	-1.05241100	-2.32135300
H	-4.77715700	0.62678000	-2.58803000

C	-3.09679800	-0.70097300	-2.26959000
H	-3.01105000	-0.93869200	-3.33820900
H	-2.86750200	-1.61135100	-1.70640900
C	-4.68379200	1.32197100	-0.01857200
C	-4.95518100	-1.01169700	0.44010000
O	-4.77121500	1.62248600	1.26877000
H	-4.89882700	0.72083200	1.72941500
O	-5.03951800	-0.84440500	1.68032400
O	-4.98359300	-2.24329600	-0.09274200
C	-4.95831300	-3.34114500	0.83609600
H	-4.98694400	-4.24110200	0.21966000
H	-4.03526300	-3.31777900	1.42029500
H	-5.82662500	-3.31157400	1.50087400
N	0.51087600	2.40909700	-1.82582700
C	3.24060700	0.63848800	-1.14266000
C	4.45752900	0.16323200	-0.62719300
C	3.08321600	0.75579000	-2.53331600
C	5.50199200	-0.17588000	-1.48870500
H	4.59122400	0.03772500	0.44162500
C	4.13589600	0.42425200	-3.38830600
H	2.14061800	1.10146100	-2.94255500
C	5.34612500	-0.04447300	-2.87069600
H	6.43572300	-0.54789900	-1.07586900
H	4.00420000	0.52585800	-4.46233500
H	6.15991100	-0.31124500	-3.53960300
C	2.53213100	1.30651000	1.61162100
C	2.02597100	0.69650100	2.76862100
C	3.55110200	2.26838200	1.72695200
C	2.53025300	1.04163100	4.02487200
H	1.23630000	-0.04424900	2.68425500
C	4.05351600	2.60886100	2.98255300
H	3.95101300	2.74795600	0.83808700
C	3.54369500	1.99619300	4.13249400
H	2.13153500	0.56301800	4.91499100
H	4.84162300	3.35236800	3.06389800
H	3.93751800	2.26363700	5.10932900
Br	-1.21900100	-2.90785800	0.36675400
C	-1.53006200	1.08881200	2.09869100
H	-0.62580100	1.65842300	2.33907500
H	-2.40401100	1.69786000	2.35495600
H	-1.54123700	0.19014900	2.72565500
O	-4.44373900	2.34390200	-0.83955400
C	-3.97512700	3.58078900	-0.26359600
H	-3.03678100	3.41870400	0.27415400
H	-3.80809600	4.24304900	-1.11346400
H	-4.72288000	4.01210600	0.40687400

TS2a

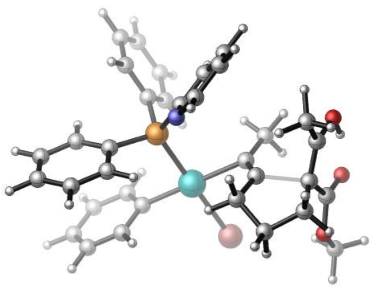

E (SMD(DMF)/B3LYP-D3/BS1) = -4636.730023
E (SMD(DMF)/M06/BS2//SMD(DMF)/B3LYP-D3/BS1) = -4639.696772

Zero-point correction=	0.610179
Thermal correction to Energy=	0.651467
Thermal correction to Enthalpy=	0.652411
Thermal correction to Gibbs Free Energy=	0.531541

Charge = 0 Multiplicity = 1

C	-2.02874700	0.07825700	-1.99246200
H	-1.90628600	1.07597000	-2.43582000
H	-1.08336400	-0.45584500	-2.13008600
C	-2.28697900	0.20365700	-0.51656300
C	-1.59578400	0.08789300	0.58018400
Pd	0.26335300	-0.98464300	0.23788200
P	1.47655600	0.94892400	0.01971700
C	0.47304900	2.46624700	-0.36359200
C	-0.06216100	3.23986600	0.67673300
C	-0.54312800	3.77580700	-1.95761100
C	-0.86693900	4.32981200	0.34952800
H	0.14389200	2.99734300	1.71319500
C	-1.10792500	4.61297600	-0.99557100
H	-0.71621100	3.95389700	-3.01707300
H	-1.29612800	4.94773600	1.13321900
H	-1.72599500	5.45330900	-1.29526000
C	2.02921300	-2.02785200	-0.02864800
C	2.36778800	-2.47886200	-1.31739300
C	2.94267600	-2.28777400	1.00636500
C	3.56961100	-3.15105900	-1.56643700
H	1.69759600	-2.28533000	-2.15320300
C	4.14943100	-2.95981800	0.76585600
H	2.72914800	-1.95310600	2.01913000
C	4.47063400	-3.39217200	-0.52332900
H	3.80661800	-3.47903700	-2.57713900
H	4.84064600	-3.13878600	1.58792600
H	5.40968700	-3.90644100	-0.71462300
C	-4.20467400	0.36944600	-0.57815500
C	-4.44970100	0.07434200	-2.06302200
H	-5.36106500	-0.52006700	-2.17420900
H	-4.60903600	1.01102700	-2.60185400

C	-3.21368100	-0.63939200	-2.63016900
H	-3.19063000	-0.58839700	-3.72360800
H	-3.21323200	-1.69184000	-2.33008100
C	-4.32324400	1.70937500	-0.10754300
C	-4.58903500	-0.70583300	0.38284600
O	-4.55289300	1.97763600	1.15449300
H	-4.70291100	1.09212000	1.62507000
O	-4.79798100	-0.50796700	1.58802400
C	-4.18343500	2.91415400	-0.97223700
H	-3.91495100	3.77691800	-0.35972300
H	-3.43629700	2.77001000	-1.75389000
H	-5.14782700	3.11852700	-1.45761500
O	-4.66322100	-1.90470200	-0.16882900
C	-4.85136000	-3.02671400	0.72751700
H	-4.95703000	-3.89402400	0.07610200
H	-3.96616000	-3.13266300	1.35794900
H	-5.75056300	-2.88767200	1.33216500
N	0.22526700	2.71949900	-1.65577900
C	2.78551000	0.86579100	-1.26667700
C	4.13495200	0.70708100	-0.91572600
C	2.41416200	0.76412800	-2.61894100
C	5.09737200	0.47243400	-1.90075400
H	4.44199600	0.74607300	0.12362000
C	3.38184900	0.54293600	-3.59938500
H	1.37249900	0.85406200	-2.90283300
C	4.72574700	0.39500700	-3.24440000
H	6.13754700	0.34626400	-1.61245200
H	3.08184500	0.47283100	-4.64169200
H	5.47574600	0.21105000	-4.00902600
C	2.31413100	1.44095000	1.57604500
C	2.06244200	0.72597100	2.75547300
C	3.14981100	2.57079700	1.61968700
C	2.64411400	1.12749400	3.96119300
H	1.40660100	-0.13931200	2.72858900
C	3.73390700	2.96543600	2.82281000
H	3.34393700	3.13826200	0.71367000
C	3.48167600	2.24403700	3.99513200
H	2.44126400	0.56704100	4.86970000
H	4.38216000	3.83714500	2.84707200
H	3.93583600	2.55546200	4.93199000
Br	-1.02936600	-3.16333800	0.65148400
C	-1.87745700	0.34673700	2.02881800
H	-0.94449100	0.47914600	2.58661300
H	-2.49114900	1.23553800	2.20020700
H	-2.39547600	-0.51380000	2.46720500

TS2b

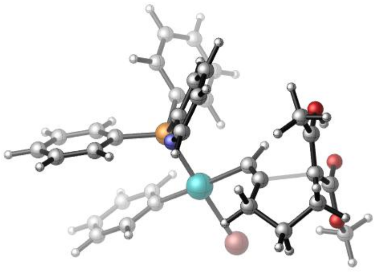

E (SMD(DMF)/B3LYP-D3/BS1) = -4597.413743
E (SMD(DMF)/M06/BS2//SMD(DMF)/B3LYP-D3/BS1) = -4600.403436

Zero-point correction=	0.581412
Thermal correction to Energy=	0.621243
Thermal correction to Enthalpy=	0.622187
Thermal correction to Gibbs Free Energy=	0.504033

Charge = 0 Multiplicity = 1

C	-2.61575900	-0.04910500	-2.12052300
H	-2.51274800	0.91714400	-2.63086700
H	-1.80503100	-0.69779900	-2.46927300
C	-2.44103300	0.12963200	-0.65292900
C	-1.59124500	0.00758400	0.30971200
Pd	0.26367800	-1.03496500	0.00038100
P	1.42908600	0.94609600	0.04533300
C	0.48022100	2.31325600	-0.76849900
C	0.04474100	3.45983900	-0.09609200
C	-0.54473100	2.97376500	-2.72123700
C	-0.72387300	4.39055100	-0.79845700
H	0.29107600	3.62532100	0.94595000
C	-1.02218000	4.14979300	-2.13822200
H	-0.76095800	2.74434800	-3.76239900
H	-1.08202500	5.28799800	-0.30207000
H	-1.61772600	4.84629400	-2.71979600
C	2.05974500	-2.03038900	-0.10569000
C	2.65619900	-2.35181100	-1.33619200
C	2.75399700	-2.37479500	1.06614800
C	3.90067300	-2.99028400	-1.39689900
H	2.15848500	-2.08620200	-2.26737900
C	3.99994500	-3.01371200	1.01331500
H	2.33327100	-2.12917800	2.04004600
C	4.58084900	-3.32187600	-0.22030000
H	4.34325300	-3.22138600	-2.36430200
H	4.51981800	-3.26237700	1.93703200
H	5.55148500	-3.81032000	-0.26515600
C	-4.36200200	0.45900400	-0.18588700
C	-4.98714000	0.19079000	-1.54862700
H	-5.94529000	-0.32254200	-1.42649600
H	-5.18934800	1.13791200	-2.05428800

C	-3.99957900	-0.64413600	-2.38734500
H	-4.25711700	-0.61337900	-3.45079000
H	-4.01787000	-1.68691200	-2.05681900
C	-4.11238700	1.78016300	0.26406300
C	-4.47640600	-0.59275300	0.85545700
O	-3.79120900	2.02524600	1.51058200
H	-3.87454300	1.15119300	2.01800100
O	-4.19105000	-0.40914100	2.04868400
C	-4.13868700	2.98201500	-0.61808300
H	-3.63468000	3.81369500	-0.12269700
H	-3.65968600	2.78316300	-1.57919000
H	-5.18153400	3.26414800	-0.81370600
O	-4.86984800	-1.76616300	0.38537400
C	-4.77077700	-2.89617200	1.28685800
H	-5.21985400	-3.72983000	0.74685400
H	-3.71495100	-3.09638400	1.48763500
H	-5.31582500	-2.70052700	2.21287600
N	0.17783700	2.06461000	-2.05537200
C	3.08779400	0.97400200	-0.75464800
C	4.21908000	0.61610700	-0.00183500
C	3.22789400	1.19852000	-2.13281500
C	5.46511600	0.49111500	-0.61685600
H	4.13091100	0.41986400	1.06130200
C	4.47954400	1.08050600	-2.74147700
H	2.36105400	1.45355000	-2.73243700
C	5.60021200	0.72399700	-1.98821300
H	6.32924900	0.20752200	-0.02211000
H	4.57415500	1.26257000	-3.80881400
H	6.57117900	0.62433800	-2.46596300
C	1.73437000	1.52909600	1.75597900
C	1.11298100	0.86732200	2.82558100
C	2.56292400	2.63489800	2.01680800
C	1.31173500	1.30655500	4.13691800
H	0.47904700	0.00698800	2.62897900
C	2.75826300	3.07109000	3.32704900
H	3.05503900	3.15134400	1.19766600
C	2.13268500	2.40781100	4.38838500
H	0.82748200	0.78592000	4.95851200
H	3.39943900	3.92673200	3.52039400
H	2.28867900	2.74842700	5.40847500
Br	-1.02619900	-3.24951700	0.01272200
H	-1.79995700	0.26232000	1.34582900

TS2c

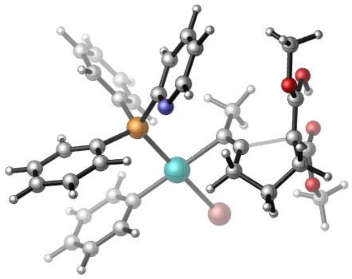

E (SMD(DMF)/B3LYP-D3/BS1) = -4711.950059
E (SMD(DMF)/M06/BS2//SMD(DMF)/B3LYP-D3/BS1) = -4714.907117

Zero-point correction=	0.614888 (Hartree/Particle)
Thermal correction to Energy=	0.657306
Thermal correction to Enthalpy=	0.658250
Thermal correction to Gibbs Free Energy=	0.535371

Charge = 0 Multiplicity = 1

C	-2.00326000	-0.30893900	-2.01356000
H	-1.95714000	0.68005700	-2.48916400
H	-1.03803800	-0.79639600	-2.18489900
C	-2.18803700	-0.14475000	-0.53511600
C	-1.47484800	-0.14664900	0.54367700
Pd	0.48855900	-1.03626200	0.17592100
P	1.51051900	1.01169900	0.03314600
C	0.34343100	2.40289900	-0.35334600
C	-0.15790900	3.24222800	0.65050100
C	-0.96222900	3.41523000	-1.95511300
C	-1.10377400	4.20630700	0.30046700
H	0.17746400	3.14798100	1.67657400
C	-1.51073200	4.30444900	-1.02965600
H	-1.26174300	3.44946700	-3.00056200
H	-1.51197700	4.87102400	1.05666000
H	-2.23992300	5.04296300	-1.34559000
C	2.34899000	-1.88909000	-0.09962300
C	2.75373200	-2.25321300	-1.39670200
C	3.26609600	-2.09100700	0.94499500
C	4.02296200	-2.78829900	-1.64392000
H	2.08098900	-2.09797200	-2.23859300
C	4.53991200	-2.62534000	0.70572500
H	3.00112300	-1.81603800	1.96366600
C	4.92596300	-2.97380700	-0.59128800
H	4.31012900	-3.05223800	-2.66032700
H	5.23238900	-2.76219000	1.53476000
H	5.91649800	-3.38087000	-0.78092300
C	-4.17988600	-0.10861700	-0.53242100
C	-4.42936800	-0.44280000	-2.00430000
H	-5.31254300	-1.08243900	-2.09909000
H	-4.63363300	0.48347200	-2.54724500

C	-3.17118800	-1.10720400	-2.58199400
H	-3.18325100	-1.09253500	-3.67691700
H	-3.10510100	-2.14720300	-2.24884800
C	-4.49323000	1.20942400	-0.06818700
C	-4.42409100	-1.17597500	0.46412400
O	-4.74641100	1.48657600	1.18067900
H	-4.75003600	0.57865400	1.66635900
O	-4.61887200	-0.96221000	1.67425600
O	-4.38536500	-2.39522900	-0.04341900
C	-4.41178900	-3.49766300	0.89515100
H	-4.43845600	-4.39441500	0.27649200
H	-3.49941800	-3.48021600	1.49456100
H	-5.29992000	-3.44090700	1.52882500
N	-0.06406500	2.47760900	-1.62983500
C	2.86800300	1.12065300	-1.20548600
C	4.20712200	0.99953300	-0.80023000
C	2.56901500	1.13577400	-2.57857400
C	5.22710500	0.91174000	-1.74979000
H	4.46153100	0.95373700	0.25290300
C	3.59436100	1.05958000	-3.52270000
H	1.53819900	1.20764800	-2.90476700
C	4.92546600	0.94494800	-3.11306000
H	6.25761300	0.81235600	-1.41941200
H	3.34903100	1.07922200	-4.58140300
H	5.72051800	0.87391500	-3.85057400
C	2.25845200	1.53918600	1.62270700
C	2.04431800	0.77410500	2.77799700
C	2.99922200	2.73081600	1.71299100
C	2.56498500	1.19047300	4.00627100
H	1.46741600	-0.14387900	2.71379600
C	3.52061400	3.14216700	2.93915000
H	3.16861700	3.33373300	0.82520300
C	3.30369600	2.37246400	4.08751100
H	2.39270100	0.59011300	4.89536200
H	4.09403600	4.06313400	2.99944400
H	3.70995400	2.69591600	5.04211100
Br	-0.59255300	-3.33532200	0.54202700
C	-1.74038900	0.13204800	1.98912400
H	-0.86159900	0.58640700	2.46030400
H	-2.58538400	0.80319600	2.15937400
H	-1.93702100	-0.80845400	2.51737600
O	-4.50378400	2.20075000	-0.91782700
C	-4.79603900	3.53855400	-0.42438000
H	-4.06567300	3.82703300	0.33273200
H	-4.71780100	4.17906500	-1.30092300
H	-5.80781000	3.56778200	-0.01410500

TS3a

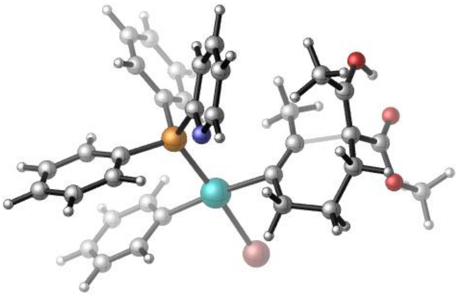

E (SMD(DMF)/B3LYP-D3/BS1) = -4636.728277
E (SMD(DMF)/M06/BS2//SMD(DMF)/B3LYP-D3/BS1) = -4639.691796

Zero-point correction=	0.610795
Thermal correction to Energy=	0.651913
Thermal correction to Enthalpy=	0.652857
Thermal correction to Gibbs Free Energy=	0.533696

Charge = 0 Multiplicity = 1

C	-1.94147500	-0.73171200	-2.06538400
H	-1.61218300	0.13083200	-2.66234400
H	-1.35749900	-1.59182000	-2.40753000
C	-1.61790400	-0.45247500	-0.62488600
C	-2.35637600	0.05258100	0.31592300
Pd	0.38954400	-1.15534200	-0.09910800
P	1.35560000	0.93846400	0.00185500
C	0.39217300	2.32333200	-0.78127700
C	0.00264900	3.48715200	-0.10809800
C	-0.49648800	3.09001600	-2.76518200
C	-0.63768900	4.49485500	-0.83285600
H	0.20209200	3.61737100	0.94839900
C	-0.88008800	4.30422400	-2.19137300
H	-0.69310500	2.88647700	-3.81560600
H	-0.94107200	5.41267600	-0.33730400
H	-1.37106300	5.06326000	-2.79207000
C	2.29250300	-1.91219400	0.15764700
C	3.03567200	-2.26923300	-0.98077500
C	2.89783100	-2.08341800	1.41196800
C	4.33976100	-2.76331600	-0.87358500
H	2.60578800	-2.14048800	-1.97223400
C	4.20451300	-2.57948000	1.52727100
H	2.36198800	-1.81688600	2.31984200
C	4.93326900	-2.91785300	0.38446900
H	4.89588800	-3.02136100	-1.77308000
H	4.65253300	-2.69400800	2.51292500
H	5.95000600	-3.29409000	0.47070100
C	-4.22758700	0.42250500	-0.26261500
C	-4.30772700	0.14028400	-1.77189400

H	-5.35866500	-0.06597400	-2.00547000
H	-4.04419300	1.04819900	-2.32006300
C	-3.43005300	-0.99999400	-2.28983000
H	-3.62228700	-1.11549200	-3.36387200
H	-3.70472200	-1.94101800	-1.80458000
C	-4.23152100	1.79060100	0.14579000
C	-5.00458900	-0.49474800	0.61806800
O	-4.64037700	2.16371300	1.33147000
H	-5.01734400	1.34250100	1.79146300
O	-5.36707000	-0.20768300	1.77115100
C	-3.70624700	2.90100700	-0.69354700
H	-3.41595400	3.73849000	-0.05627500
H	-2.85331700	2.57550500	-1.28871000
H	-4.49116200	3.24378900	-1.38119900
O	-5.27669100	-1.67119000	0.07217600
C	-5.99931800	-2.61925800	0.89529400
H	-6.11397900	-3.50602800	0.27243800
H	-5.42402900	-2.85264900	1.79454800
H	-6.97552300	-2.21385200	1.17204600
N	0.11752400	2.11611400	-2.08169700
C	2.99514800	1.04346400	-0.84789000
C	4.18340600	0.90571500	-0.11216400
C	3.06571500	1.10157100	-2.24891400
C	5.41558700	0.83902800	-0.76473000
H	4.15537000	0.82634700	0.96870100
C	4.30144700	1.04299800	-2.89646100
H	2.15605200	1.18561900	-2.83310400
C	5.48003000	0.90999300	-2.15828200
H	6.32430200	0.72520500	-0.17970700
H	4.33934200	1.09419700	-3.98152600
H	6.43985900	0.85556000	-2.66498800
C	1.70784900	1.51981200	1.70710200
C	1.27276600	0.75943400	2.80133100
C	2.41963800	2.71068500	1.94154700
C	1.52757100	1.18549900	4.10730300
H	0.74287300	-0.17164000	2.63018900
C	2.67175600	3.13566200	3.24555500
H	2.77893300	3.30300600	1.10501300
C	2.22430500	2.37436000	4.33078400
H	1.18452100	0.58522900	4.94545800
H	3.21991400	4.05844400	3.41469400
H	2.42364900	2.70638300	5.34613200
Br	-0.50876800	-3.55346800	-0.14712500
C	-2.19743300	0.42795300	1.74826000
H	-1.33596400	-0.11303400	2.14349000
H	-2.01244000	1.50095700	1.88343200
H	-3.05886600	0.15264100	2.36281600

IVa

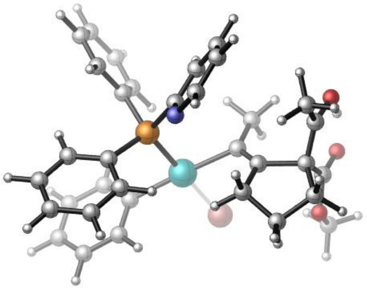

E (SMD(DMF)/B3LYP-D3/BS1) = -4636.734447
E (SMD(DMF)/M06/BS2//SMD(DMF)/B3LYP-D3/BS1) = -4639.701422

Zero-point correction=	0.610179
Thermal correction to Energy=	0.651467
Thermal correction to Enthalpy=	0.652411
Thermal correction to Gibbs Free Energy=	0.531541

Charge = 0 Multiplicity = 1

C	-2.02874700	0.07825700	-1.99246200
H	-1.90628600	1.07597000	-2.43582000
H	-1.08336400	-0.45584500	-2.13008600
C	-2.28697900	0.20365700	-0.51656300
C	-1.59578400	0.08789300	0.58018400
Pd	0.26335300	-0.98464300	0.23788200
P	1.47655600	0.94892400	0.01971700
C	0.47304900	2.46624700	-0.36359200
C	-0.06216100	3.23986600	0.67673300
C	-0.54312800	3.77580700	-1.95761100
C	-0.86693900	4.32981200	0.34952800
H	0.14389200	2.99734300	1.71319500
C	-1.10792500	4.61297600	-0.99557100
H	-0.71621100	3.95389700	-3.01707300
H	-1.29612800	4.94773600	1.13321900
H	-1.72599500	5.45330900	-1.29526000
C	2.02921300	-2.02785200	-0.02864800
C	2.36778800	-2.47886200	-1.31739300
C	2.94267600	-2.28777400	1.00636500
C	3.56961100	-3.15105900	-1.56643700
H	1.69759600	-2.28533000	-2.15320300
C	4.14943100	-2.95981800	0.76585600
H	2.72914800	-1.95310600	2.01913000
C	4.47063400	-3.39217200	-0.52332900
H	3.80661800	-3.47903700	-2.57713900
H	4.84064600	-3.13878600	1.58792600
H	5.40968700	-3.90644100	-0.71462300
C	-4.20467400	0.36944600	-0.57815500
C	-4.44970100	0.07434200	-2.06302200
H	-5.36106500	-0.52006700	-2.17420900
H	-4.60903600	1.01102700	-2.60185400

C	-3.21368100	-0.63939200	-2.63016900
H	-3.19063000	-0.58839700	-3.72360800
H	-3.21323200	-1.69184000	-2.33008100
C	-4.32324400	1.70937500	-0.10754300
C	-4.58903500	-0.70583300	0.38284600
O	-4.55289300	1.97763600	1.15449300
H	-4.70291100	1.09212000	1.62507000
O	-4.79798100	-0.50796700	1.58802400
C	-4.18343500	2.91415400	-0.97223700
H	-3.91495100	3.77691800	-0.35972300
H	-3.43629700	2.77001000	-1.75389000
H	-5.14782700	3.11852700	-1.45761500
O	-4.66322100	-1.90470200	-0.16882900
C	-4.85136000	-3.02671400	0.72751700
H	-4.95703000	-3.89402400	0.07610200
H	-3.96616000	-3.13266300	1.35794900
H	-5.75056300	-2.88767200	1.33216500
N	0.22526700	2.71949900	-1.65577900
C	2.78551000	0.86579100	-1.26667700
C	4.13495200	0.70708100	-0.91572600
C	2.41416200	0.76412800	-2.61894100
C	5.09737200	0.47243400	-1.90075400
H	4.44199600	0.74607300	0.12362000
C	3.38184900	0.54293600	-3.59938500
H	1.37249900	0.85406200	-2.90283300
C	4.72574700	0.39500700	-3.24440000
H	6.13754700	0.34626400	-1.61245200
H	3.08184500	0.47283100	-4.64169200
H	5.47574600	0.21105000	-4.00902600
C	2.31413100	1.44095000	1.57604500
C	2.06244200	0.72597100	2.75547300
C	3.14981100	2.57079700	1.61968700
C	2.64411400	1.12749400	3.96119300
H	1.40660100	-0.13931200	2.72858900
C	3.73390700	2.96543600	2.82281000
H	3.34393700	3.13826200	0.71367000
C	3.48167600	2.24403700	3.99513200
H	2.44126400	0.56704100	4.86970000
H	4.38216000	3.83714500	2.84707200
H	3.93583600	2.55546200	4.93199000
Br	-1.02936600	-3.16333800	0.65148400
C	-1.87745700	0.34673700	2.02881800
H	-0.94449100	0.47914600	2.58661300
H	-2.49114900	1.23553800	2.20020700
H	-2.39547600	-0.51380000	2.46720500

IVb

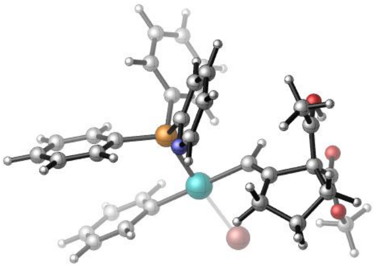

E (SMD(DMF)/B3LYP-D3/BS1) = -4597.419513
E (SMD(DMF)/M06/BS2//SMD(DMF)/B3LYP-D3/BS1) = -4600.410089

Zero-point correction=	0.583187
Thermal correction to Energy=	0.622800
Thermal correction to Enthalpy=	0.623744
Thermal correction to Gibbs Free Energy=	0.506670

Charge = 0 Multiplicity = 1

C	-2.52434300	0.00179100	-2.15653600
H	-2.29958800	0.96022800	-2.64339000
H	-1.74152300	-0.70674600	-2.44216800
C	-2.53652600	0.16599700	-0.65206200
C	-1.58248200	-0.04637600	0.25585800
Pd	0.23395300	-1.04912200	-0.01711300
P	1.38440200	0.92963100	0.07345100
C	0.53666200	2.30414200	-0.83623300
C	0.13549100	3.50531300	-0.24160800
C	-0.31606800	2.94278300	-2.87834100
C	-0.51819000	4.45348600	-1.03146200
H	0.32479100	3.70127900	0.80699500
C	-0.74585500	4.17363700	-2.37758000
H	-0.48170300	2.68055800	-3.92105900
H	-0.84370300	5.39434500	-0.59683900
H	-1.25025100	4.88267100	-3.02640000
C	2.02620700	-2.09239200	-0.18064100
C	2.67765200	-2.27241400	-1.41520200
C	2.66509500	-2.62022800	0.95695700
C	3.90372100	-2.94167700	-1.51424400
H	2.23718800	-1.86171500	-2.32361500
C	3.89413400	-3.28746800	0.87204000
H	2.20453000	-2.50356100	1.93756200
C	4.52138100	-3.45100300	-0.36707100
H	4.38209500	-3.05723700	-2.48565700
H	4.36438600	-3.67598300	1.77431400
H	5.47820200	-3.96355900	-0.43764900
C	-4.08372000	0.48082900	-0.25635100
C	-4.84618800	0.37766700	-1.60026100
H	-5.83524300	-0.06049300	-1.44853100
H	-4.99129200	1.37626600	-2.01845900

C	-3.94030200	-0.45471300	-2.52567500
H	-4.18278900	-0.29207200	-3.58045300
H	-4.05902500	-1.52000300	-2.30412200
C	-3.87460200	1.82086100	0.30872000
C	-4.43449000	-0.58205900	0.76039100
O	-3.71899200	1.97847800	1.58086900
H	-3.90477900	1.07309000	2.03047600
O	-4.29034100	-0.42483600	1.98048800
C	-3.76270800	3.05078500	-0.51364700
H	-3.19295700	3.80905700	0.02629200
H	-3.30500900	2.85294800	-1.48419100
H	-4.77859800	3.43334400	-0.68911100
O	-4.83107000	-1.71134200	0.21793900
C	-4.90575400	-2.87257600	1.08821900
H	-5.34244700	-3.65816400	0.47242100
H	-3.89152400	-3.14191500	1.39298400
H	-5.53765400	-2.66267000	1.95334700
N	0.30062300	2.01941500	-2.12987200
C	3.10825800	0.94745400	-0.57879700
C	4.14834200	0.50113700	0.25484800
C	3.39170500	1.25342100	-1.91780900
C	5.44401000	0.36637000	-0.24280400
H	3.94769500	0.24382300	1.28969600
C	4.69321400	1.12332000	-2.40995500
H	2.59937800	1.57797000	-2.58316900
C	5.72144600	0.67768900	-1.57736900
H	6.23566100	0.01331000	0.41257000
H	4.89837200	1.36692400	-3.44912500
H	6.73105400	0.56962700	-1.96464700
C	1.56632500	1.54020600	1.79531300
C	0.86407100	0.90587100	2.83068700
C	2.39466400	2.63603600	2.09652500
C	0.97948400	1.36454100	4.14552000
H	0.23603200	0.04855800	2.60681300
C	2.50603500	3.09275100	3.40958800
H	2.95257700	3.12952400	1.30590700
C	1.79783900	2.45806500	4.43580300
H	0.43311400	0.86382100	4.94016500
H	3.14744000	3.94101900	3.63262400
H	1.88897300	2.81365600	5.45864500
Br	-1.11222800	-3.24284400	0.07995100
H	-1.83145100	0.17297200	1.29875000

IVc

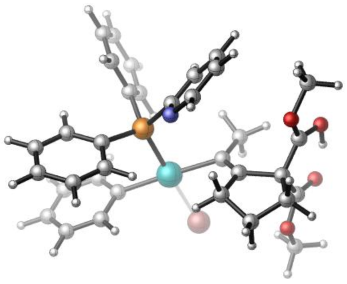

E (SMD(DMF)/B3LYP-D3/BS1) = -4711.958772
E (SMD(DMF)/M06/BS2//SMD(DMF)/B3LYP-D3/BS1) = -4714.915877

Zero-point correction=	0.615876
Thermal correction to Energy=	0.658140
Thermal correction to Enthalpy=	0.659084
Thermal correction to Gibbs Free Energy=	0.536889

Charge = 0 Multiplicity = 1

C	1.84283400	-0.21424500	2.02988200
H	1.58727700	0.78699600	2.40514600
H	0.94921700	-0.83674300	2.13626000
C	2.26478500	-0.12506800	0.56861300
C	1.47522300	-0.26891700	-0.50336600
Pd	-0.47154900	-1.04707000	-0.28315200
P	-1.42622400	1.00325500	-0.01328000
C	-0.29132800	2.42432100	0.38187200
C	0.37245800	3.09504600	-0.65666900
C	0.73532100	3.72816300	1.97431900
C	1.23898900	4.13580600	-0.33096100
H	0.20797600	2.81615100	-1.69183500
C	1.42258000	4.46754800	1.01295600
H	0.85871100	3.94623700	3.03333300
H	1.76084200	4.67904600	-1.11347200
H	2.08328100	5.27531800	1.31143700
C	-2.38437200	-1.88637100	-0.03201900
C	-2.77641600	-2.32289400	1.24976900
C	-3.34287400	-2.01694200	-1.05485900
C	-4.04733200	-2.85123200	1.50301400
H	-2.08343000	-2.22811800	2.08559200
C	-4.62041800	-2.54401600	-0.81692100
H	-3.10425800	-1.68889600	-2.06553200
C	-4.98135800	-2.96226000	0.46660600
H	-4.31318400	-3.16856800	2.51042300
H	-5.33587100	-2.62114000	-1.63472700
H	-5.97385700	-3.36420200	0.65846500
C	3.85922800	-0.05936600	0.56549300
C	4.24762900	-0.10606600	2.08209200
H	5.19757500	-0.62740400	2.22804900
H	4.36843100	0.91965200	2.43949600

C	3.05358400	-0.76241000	2.78847700
H	3.04285000	-0.51487600	3.85501700
H	3.10666400	-1.85011400	2.68862300
C	4.36048200	1.18912500	-0.08721200
C	4.33779400	-1.29443300	-0.18736800
O	5.00980200	1.19409800	-1.19519600
H	5.08279800	0.18342200	-1.48647500
O	4.86623900	-1.25370700	-1.31160300
O	4.10585800	-2.41231400	0.45024900
C	4.32806600	-3.65659000	-0.26762300
H	4.17651200	-4.43819800	0.47540700
H	3.58378400	-3.73751200	-1.06183300
H	5.34421800	-3.68926700	-0.66548200
N	-0.10407900	2.72629200	1.67341000
C	-2.71550400	1.04065200	1.29524200
C	-4.08178800	1.10466100	0.98441700
C	-2.32802000	0.81242100	2.62734800
C	-5.04188300	0.96523400	1.99034700
H	-4.40734700	1.24424200	-0.04063500
C	-3.28976300	0.68697900	3.62967500
H	-1.27768900	0.72226200	2.87862600
C	-4.64999800	0.76212700	3.31486300
H	-6.09672700	1.01132300	1.73285200
H	-2.97569500	0.51597300	4.65607800
H	-5.39818600	0.65208000	4.09539800
C	-2.22311900	1.61388000	-1.55146800
C	-2.07541600	0.88976800	-2.74277900
C	-2.91679000	2.83654600	-1.57077200
C	-2.62277400	1.37242200	-3.93477400
H	-1.52436600	-0.04633200	-2.73447900
C	-3.46789200	3.31292700	-2.75998800
H	-3.02640100	3.41386700	-0.65687800
C	-3.32190700	2.58098500	-3.94367300
H	-2.50094900	0.80349700	-4.85248900
H	-4.00721300	4.25624100	-2.76461400
H	-3.74947000	2.95597200	-4.86971500
Br	0.57568700	-3.35594200	-0.79120000
C	1.90458200	-0.12749800	-1.93933000
H	1.15518200	0.44297100	-2.50387300
H	2.86639300	0.37179500	-2.10303300
H	1.95430900	-1.12092400	-2.40373900
O	4.11520200	2.30928800	0.49855100
C	4.60369400	3.54883400	-0.11239000
H	4.13998800	3.67722800	-1.09083000
H	4.29549300	4.33183700	0.57539800
H	5.69070700	3.50275500	-0.19649800

Va

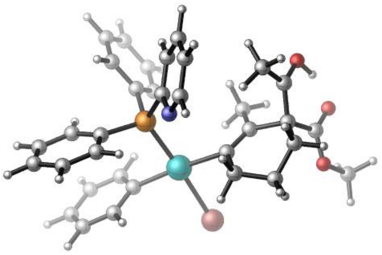

E (SMD(DMF)/B3LYP-D3/BS1) = -4636.735242
E (SMD(DMF)/M06/BS2//SMD(DMF)/B3LYP-D3/BS1) = -4639.700663

Zero-point correction=	0.611824
Thermal correction to Energy=	0.652970
Thermal correction to Enthalpy=	0.653914
Thermal correction to Gibbs Free Energy=	0.534330

Charge = 0 Multiplicity = 1

C	-1.90816100	-0.51884600	-2.10758600
H	-1.57701900	0.39537500	-2.62168000
H	-1.31265500	-1.33839900	-2.52480700
C	-1.60294400	-0.38663400	-0.63462900
C	-2.51492100	0.03140300	0.26483600
Pd	0.31780500	-1.12545500	-0.13181800
P	1.35733900	0.92463800	-0.01027200
C	0.49761100	2.38145500	-0.78948600
C	0.19106900	3.56758900	-0.11188500
C	-0.34673900	3.21594300	-2.76682700
C	-0.38719900	4.61793600	-0.82829500
H	0.40410600	3.68116100	0.94374000
C	-0.65491000	4.44768200	-2.18471400
H	-0.55850100	3.03105300	-3.81784100
H	-0.62612200	5.55175300	-0.32718400
H	-1.10187200	5.23839200	-2.77891800
C	2.21001700	-1.96095100	0.20488700
C	2.96865800	-2.39926500	-0.89811600
C	2.79456800	-2.11408900	1.47457100
C	4.24674300	-2.94733300	-0.74779900
H	2.56674500	-2.29258800	-1.90498500
C	4.07392500	-2.66481100	1.63974400
H	2.25833700	-1.78397800	2.36267600
C	4.80983900	-3.08036900	0.52711800
H	4.80826500	-3.26481900	-1.62522900
H	4.49738500	-2.76067200	2.63864500
H	5.80648200	-3.49884400	0.64893600
C	-4.00398100	0.42578000	-0.19361600
C	-4.23862200	0.31329200	-1.72984500
H	-5.30776600	0.14717100	-1.89494500
H	-3.99810600	1.27035700	-2.19994800

C	-3.39257700	-0.77144200	-2.38334900
H	-3.58974600	-0.76998100	-3.46255000
H	-3.67075700	-1.75571900	-1.99432000
C	-4.01635600	1.83781500	0.27176800
C	-4.97591200	-0.45101600	0.58526900
O	-4.55101900	2.16855900	1.39372300
H	-5.05113100	1.34070100	1.75472900
O	-5.51769800	-0.10671100	1.64646000
C	-3.32273600	2.92984100	-0.44968700
H	-2.87226700	3.61516300	0.27209400
H	-2.56828800	2.55089800	-1.13524500
H	-4.07400000	3.49195500	-1.02298500
O	-5.16079200	-1.63657900	0.05017700
C	-6.02360800	-2.55898700	0.76959100
H	-6.02942500	-3.46620200	0.16703300
H	-5.61191600	-2.75492700	1.76220200
H	-7.02969100	-2.14209900	0.85327500
N	0.20897900	2.20247600	-2.09133500
C	3.00906700	0.94752800	-0.84629200
C	4.18767300	0.76027500	-0.10633500
C	3.08737100	0.99073000	-2.24752100
C	5.41730900	0.63020300	-0.75426200
H	4.15288500	0.68923600	0.97472900
C	4.32062000	0.86861200	-2.89102200
H	2.18449500	1.11323700	-2.83570100
C	5.48960200	0.68641100	-2.14810100
H	6.31775100	0.47769600	-0.16515600
H	4.36374200	0.90864000	-3.97641500
H	6.44731600	0.58206800	-2.65106200
C	1.71685800	1.48329100	1.70281900
C	1.17230000	0.77793200	2.78492200
C	2.52266800	2.60833800	1.95683300
C	1.41694800	1.19153900	4.09695800
H	0.56316200	-0.09951000	2.59900300
C	2.76643400	3.01994800	3.26691000
H	2.96117300	3.16086300	1.13101200
C	2.21222800	2.31298600	4.33945800
H	0.98850600	0.63398400	4.92532800
H	3.38970300	3.89085800	3.45051900
H	2.40442800	2.63464400	5.35954300
Br	-0.69030100	-3.48332600	-0.23210400
C	-2.24173300	0.14553700	1.75212100
H	-1.48978100	-0.59539100	2.03028000
H	-1.85424400	1.13352000	2.03883900
H	-3.12321600	-0.04725500	2.37356700

VIa

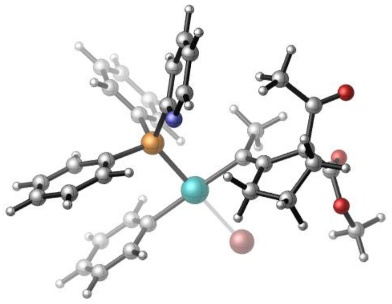

E (SMD(DMF)/B3LYP-D3/BS1) = -4636.28729
E (SMD(DMF)/M06/BS2//SMD(DMF)/B3LYP-D3/BS1) = -4639.264879

Zero-point correction=	0.598604
Thermal correction to Energy=	0.639981
Thermal correction to Enthalpy=	0.640925
Thermal correction to Gibbs Free Energy=	0.520299

Charge = -1 Multiplicity = 1

C	2.25712400	-0.26471700	1.94439100
H	1.64383400	0.50314200	2.43832200
H	1.69675400	-1.20465500	1.99741300
C	2.50947000	0.13253700	0.49066000
C	1.59662600	0.02708500	-0.48733000
Pd	-0.22230000	-0.99268000	-0.18796600
P	-1.44068700	0.92580200	-0.02039700
C	-0.56128700	2.47804900	0.51314700
C	-0.37099500	3.58304800	-0.32636400
C	0.51469800	3.53568600	2.25628700
C	0.27845500	4.70895600	0.18452300
H	-0.72607900	3.57546400	-1.34963800
C	0.71903200	4.69594700	1.50591100
H	0.86508500	3.47217500	3.28446500
H	0.43352800	5.58088100	-0.44472200
H	1.22309700	5.55104200	1.94505900
C	-2.02313400	-2.08297000	0.03889500
C	-2.42938800	-2.55615500	1.30356500
C	-2.89947200	-2.35325100	-1.03126200
C	-3.63179500	-3.24659900	1.49623400
H	-1.80237600	-2.36468000	2.17516800
C	-4.10720100	-3.04398800	-0.85683500
H	-2.65103400	-2.00163700	-2.03291200
C	-4.48264500	-3.49344500	0.41240300
H	-3.91070300	-3.58637500	2.49305900
H	-4.75874200	-3.22419200	-1.71134800
H	-5.42209400	-4.02285600	0.55672400
C	4.01134400	0.51694400	0.33554700
C	4.52774200	0.58550300	1.80812300
H	5.59599800	0.35804600	1.87116500
H	4.38835600	1.61007300	2.17615200

C	3.63651400	-0.37055800	2.61654600
H	3.61579600	-0.10437800	3.67986900
H	4.02229700	-1.38951900	2.53079600
C	4.34272300	1.87518600	-0.29110700
C	4.62860900	-0.62142400	-0.47781300
O	5.41037200	2.07063300	-0.85083500
O	4.72208200	-0.65535900	-1.68914300
C	3.34018000	2.98967200	-0.08831400
H	3.81778200	3.95505400	-0.27646900
H	2.50119500	2.86536300	-0.78011100
H	2.91813300	2.96877700	0.91972100
O	4.98526500	-1.66345100	0.30535200
C	5.25272700	-2.89596400	-0.38571900
H	5.56076600	-3.60433800	0.38578800
H	4.33820800	-3.24515300	-0.87556000
H	6.05080100	-2.77174200	-1.12327600
N	-0.09911200	2.44824100	1.77591400
C	-2.86388700	0.81605500	1.15655100
C	-4.15836600	0.53166000	0.69226900
C	-2.63176300	0.83792000	2.54211900
C	-5.19802600	0.29032300	1.59289400
H	-4.36139600	0.47506200	-0.37120800
C	-3.67630500	0.60525800	3.43879700
H	-1.63412000	1.03414600	2.91766900
C	-4.96270500	0.32906900	2.96896200
H	-6.19151000	0.06575000	1.21362900
H	-3.47907300	0.63125100	4.50748700
H	-5.77240500	0.13848900	3.66840200
C	-2.20362700	1.40167200	-1.62424700
C	-1.83938700	0.70720600	-2.78717200
C	-3.12920400	2.45634400	-1.71838800
C	-2.38931600	1.05817300	-4.02301200
H	-1.12491800	-0.10828100	-2.72064800
C	-3.67779100	2.80481200	-2.95247600
H	-3.42121400	3.00337100	-0.82632400
C	-3.30885100	2.10549300	-4.10683400
H	-2.09952100	0.51114600	-4.91608700
H	-4.39290400	3.62075800	-3.01354000
H	-3.73865400	2.37760000	-5.06722000
Br	1.12396100	-3.17511100	-0.49275000
C	1.83541800	0.43712800	-1.91868500
H	1.08884900	1.18052200	-2.23689600
H	2.83218800	0.84785700	-2.12069100
H	1.71050900	-0.42924300	-2.58357700

VIc

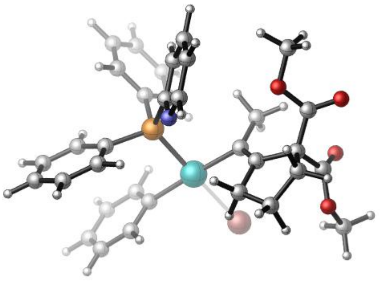

E (SMD(DMF)/B3LYP-D3/BS1) = -4711.516831
E (SMD(DMF)/M06/BS2//SMD(DMF)/B3LYP-D3/BS1) = -4714.48621

Zero-point correction=	0.603920
Thermal correction to Energy=	0.646476
Thermal correction to Enthalpy=	0.647420
Thermal correction to Gibbs Free Energy=	0.523738

Charge = -1 Multiplicity = 1

C	-2.10881400	-0.66233300	-1.94701500
H	-1.48408200	0.07684100	-2.46854200
H	-1.54034200	-1.59866800	-1.91738300
C	-2.40940900	-0.17326400	-0.52983200
C	-1.50638100	-0.13549900	0.46095300
Pd	0.39033800	-1.02497600	0.22636200
P	1.47756000	0.96569600	-0.01861600
C	0.47432000	2.42151200	-0.59731300
C	0.21349200	3.54365000	0.19839100
C	-0.78242600	3.27486900	-2.33081700
C	-0.57243200	4.56956500	-0.32993000
H	0.61432100	3.62392100	1.20143800
C	-1.08013100	4.43944900	-1.62086800
H	-1.16812100	3.12560500	-3.33733700
H	-0.78377100	5.45478700	0.26349700
H	-1.69898600	5.21123000	-2.06762100
C	2.25915300	-1.99706000	0.02786800
C	2.65964000	-2.51765800	-1.22017700
C	3.18058100	-2.14550900	1.08389200
C	3.89905400	-3.13996500	-1.41062800
H	1.99517200	-2.42218600	-2.07975800
C	4.42544900	-2.76742100	0.91129400
H	2.93754000	-1.74989000	2.07045400
C	4.79414000	-3.26589800	-0.34163000
H	4.17186000	-3.52115000	-2.39408800
H	5.11129500	-2.85452700	1.75343600
H	5.76181600	-3.74215300	-0.48464800
C	-3.93722600	0.09567100	-0.41993700
C	-4.42158500	0.09555200	-1.90248500
H	-5.47511700	-0.18740900	-1.98909900
H	-4.31836700	1.11946200	-2.28346000

C	-3.46176400	-0.83212400	-2.66341300
H	-3.41440600	-0.57487100	-3.72792200
H	-3.80864200	-1.86497700	-2.58323800
C	-4.42621800	1.40293200	0.18861400
C	-4.46360400	-1.07253400	0.41738900
O	-5.53921800	1.55829400	0.65558900
O	-4.59461900	-1.06764100	1.62590000
O	-4.67628200	-2.17161900	-0.33517100
C	-4.82680900	-3.40316500	0.39379800
H	-5.02583900	-4.16715200	-0.36014300
H	-3.89530000	-3.62768000	0.92258100
H	-5.65971000	-3.34494400	1.10030900
N	-0.02812400	2.28700500	-1.83721300
C	2.90748600	0.92858000	-1.19183500
C	4.21362000	0.71854700	-0.71906500
C	2.68336100	0.94113300	-2.57844500
C	5.27112600	0.53833300	-1.61271200
H	4.41164900	0.67334100	0.34584400
C	3.74541800	0.76790600	-3.46844300
H	1.67851900	1.08284900	-2.96000000
C	5.04230800	0.56464100	-2.99046700
H	6.27352500	0.37119500	-1.22733200
H	3.55425400	0.78495000	-4.53842100
H	5.86588800	0.42135200	-3.68498600
C	2.20299000	1.55120700	1.56630300
C	1.86764000	0.88968300	2.75671800
C	3.06576100	2.66051800	1.61917400
C	2.38408400	1.32716600	3.97937300
H	1.20208900	0.03173300	2.72179300
C	3.58115700	3.09525200	2.84016200
H	3.33449800	3.18284300	0.70536200
C	3.24128500	2.42849300	4.02236300
H	2.11733400	0.80500700	4.89427100
H	4.24781200	3.95294100	2.86918800
H	3.64490800	2.76804400	4.97255800
Br	-0.79158500	-3.28367900	0.65195000
C	-1.79524800	0.36270100	1.85345800
H	-1.13847900	1.21032000	2.10135000
H	-2.83115700	0.67926000	2.02439500
H	-1.57601300	-0.42331800	2.59014600
O	-3.54508300	2.41106600	0.04751600
C	-4.01022500	3.69410100	0.50304800
H	-4.89723000	4.00676900	-0.05653600
H	-4.25249100	3.65907200	1.56946400
H	-3.18819000	4.38563700	0.32590300

VIIa

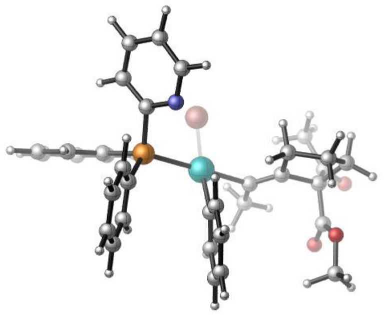

E (SMD(DMF)/B3LYP-D3/BS1) = -4636.298533
E (SMD(DMF)/M06/BS2//SMD(DMF)/B3LYP-D3/BS1) = -4639.273997

Zero-point correction=	0.598623
Thermal correction to Energy=	0.640317
Thermal correction to Enthalpy=	0.641261
Thermal correction to Gibbs Free Energy=	0.517588

Charge = -1 Multiplicity = 1

C	-2.64214700	-0.14497100	1.88399200
H	-2.25936000	-1.06299500	2.35703400
H	-1.85646600	0.60959200	1.98492200
C	-2.98234300	-0.42895100	0.42553500
C	-2.09898400	-0.52174200	-0.58110900
Pd	-0.07392700	-0.32102100	-0.32303600
P	2.31625800	-0.03135500	-0.00721600
C	2.80299200	-0.78808300	1.60534900
C	3.79974600	-1.75620800	1.75925200
C	2.27299600	-0.86900600	3.84573600
C	4.01654400	-2.29546400	3.02910100
H	4.39142100	-2.08189800	0.91102000
C	3.24082300	-1.84552100	4.09612700
H	1.64957300	-0.48942800	4.65299500
H	4.78070200	-3.05329100	3.17888400
H	3.37474700	-2.23814500	5.09933500
C	-0.44699700	1.68459600	-0.31946700
C	-0.21246000	2.45092600	0.84129800
C	-0.89814200	2.38305100	-1.45585500
C	-0.41026500	3.83596800	0.86593500
H	0.16292800	1.96578600	1.74001900
C	-1.08892200	3.77138200	-1.44399900
H	-1.10288800	1.84355000	-2.37839300
C	-0.84732500	4.50742400	-0.28032100
H	-0.21108700	4.39241800	1.78030800
H	-1.42803300	4.27665200	-2.34699400
H	-0.99540600	5.58480100	-0.26717200
C	-4.52458100	-0.61182000	0.30958800
C	-5.02310100	-0.53551200	1.79170900
H	-6.03229100	-0.11969100	1.85510400
H	-5.06081300	-1.55135700	2.20325000

C	-3.96077100	0.27596800	2.54809500
H	-3.98206600	0.07609700	3.62551100
H	-4.13493700	1.34528100	2.39685600
C	-5.00162400	-1.96393700	-0.24421000
C	-5.03482300	0.54961600	-0.54584100
O	-6.08719700	-2.07539400	-0.79049600
O	-5.18769300	0.53952000	-1.75139200
C	-4.11911700	-3.16734800	0.02315700
H	-3.15673600	-3.07688200	-0.49069500
H	-3.88490400	-3.23831800	1.09230100
H	-4.63736900	-4.07490800	-0.29739300
O	-5.21566800	1.66919500	0.19206100
C	-5.47794500	2.87165000	-0.55277800
H	-5.61447600	3.65534700	0.19414500
H	-4.62704500	3.11195000	-1.19856200
H	-6.37975400	2.76782300	-1.16274200
N	2.04635000	-0.35194300	2.63229800
C	2.91576800	1.70714400	0.13240900
C	2.69108800	2.54095900	-0.97725200
C	3.51490500	2.24849900	1.27723200
C	3.05107700	3.88654300	-0.94005600
H	2.21305700	2.13949900	-1.86655600
C	3.87148100	3.60108100	1.31486500
H	3.69976700	1.62894900	2.14825900
C	3.63839000	4.42306700	0.21137200
H	2.86176500	4.51906900	-1.80329800
H	4.33151600	4.00878300	2.21141200
H	3.90995200	5.47483600	0.24625900
C	3.49647600	-0.72935000	-1.23754300
C	3.03168800	-1.64647700	-2.19226300
C	4.85027000	-0.34904600	-1.25310200
C	3.90795300	-2.17577400	-3.14423700
H	1.99345000	-1.96290800	-2.16829300
C	5.72253100	-0.88188100	-2.20289400
H	5.22240800	0.36548600	-0.52400900
C	5.25177200	-1.79521600	-3.15223600
H	3.53774200	-2.88526600	-3.88000300
H	6.76719200	-0.58179300	-2.20483600
H	5.93091500	-2.20608400	-3.89485900
Br	0.14938700	-2.95905600	-0.32340300
C	-2.45132700	-0.89345600	-2.00042400
H	-1.86316300	-1.77102600	-2.29866500
H	-3.51279100	-1.11480000	-2.16001200
H	-2.19310100	-0.08450900	-2.69856000

VIIc

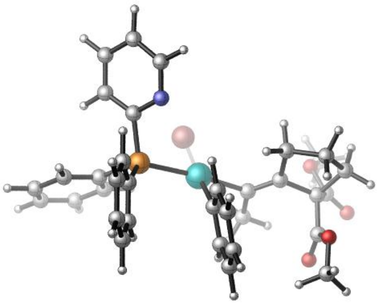

E (SMD(DMF)/B3LYP-D3/BS1) = -4711.527861
E (SMD(DMF)/M06/BS2//SMD(DMF)/B3LYP-D3/BS1) = -4714.495098

Zero-point correction=	0.604410
Thermal correction to Energy=	0.646891
Thermal correction to Enthalpy=	0.647835
Thermal correction to Gibbs Free Energy=	0.524216

Charge = -1 Multiplicity = 1

C	-2.63903600	0.07559900	1.98636300
H	-2.45123100	-0.84741100	2.55778800
H	-1.74249000	0.69417700	2.08410900
C	-2.93295900	-0.28091800	0.53321500
C	-2.01625200	-0.57026400	-0.40467200
Pd	0.02424200	-0.40008400	-0.16152300
P	2.42780300	-0.06327100	-0.01932600
C	3.08206000	-0.80503600	1.53898500
C	4.12251700	-1.73653200	1.60220400
C	2.75906300	-0.89804800	3.81793200
C	4.47271500	-2.26288200	2.84727400
H	4.64621300	-2.04385400	0.70400800
C	3.78094900	-1.83721100	3.98004000
H	2.19743000	-0.53879100	4.67809100
H	5.27432200	-2.99207400	2.92778000
H	4.01963400	-2.22088900	4.96713900
C	-0.35815900	1.60314100	-0.16105400
C	-0.03540000	2.38870000	0.96489400
C	-0.91254500	2.27872500	-1.26439600
C	-0.24694000	3.77188100	0.98677700
H	0.41726500	1.92015800	1.83642200
C	-1.10938500	3.66618500	-1.25908400
H	-1.20000700	1.72195200	-2.15376300
C	-0.78178300	4.42215500	-0.12976200
H	0.01925700	4.34337100	1.87434800
H	-1.52472700	4.15466100	-2.13896900
H	-0.93766600	5.49845200	-0.12090200
C	-4.47476100	-0.18964400	0.31896000
C	-5.03874000	0.07968800	1.75249500
H	-5.96939400	0.65300800	1.72283800
H	-5.25641600	-0.88952000	2.21753900

C	-3.90111400	0.76493000	2.52212900
H	-4.02061000	0.66049600	3.60646200
H	-3.87964700	1.83194500	2.28302800
C	-5.20048600	-1.42417700	-0.20335100
C	-4.69732300	0.97931800	-0.64687500
O	-6.26536100	-1.40224000	-0.79028200
O	-4.77432000	0.89597900	-1.85658100
O	-4.71791700	2.16391800	-0.00006700
C	-4.77684800	3.33167800	-0.83941600
H	-4.69895500	4.18307400	-0.16160300
H	-3.94709200	3.33772400	-1.55010700
H	-5.72488200	3.36677700	-1.38486500
N	2.40482300	-0.39392700	2.62963200
C	2.99918900	1.68939500	0.05730000
C	2.71810700	2.49033400	-1.06403500
C	3.61182000	2.27548300	1.17224000
C	3.03501900	3.84700600	-1.06625600
H	2.23138900	2.05362600	-1.93189900
C	3.92647400	3.63906300	1.16967600
H	3.83641400	1.68319600	2.05265800
C	3.63672800	4.42819100	0.05585200
H	2.80230700	4.45326100	-1.93774400
H	4.39774300	4.08115600	2.04381800
H	3.87532900	5.48847800	0.05968400
C	3.49796800	-0.72707000	-1.36422700
C	2.95192100	-1.60568200	-2.31178000
C	4.84619600	-0.34379700	-1.48113500
C	3.74259700	-2.09493800	-3.35574200
H	1.91915600	-1.92392900	-2.21226200
C	5.63382300	-0.83814900	-2.52125600
H	5.27987200	0.34451700	-0.76081900
C	5.08182800	-1.71345900	-3.46263800
H	3.30926200	-2.77419300	-4.08554100
H	6.67508700	-0.53662900	-2.60031900
H	5.69427700	-2.09355500	-4.27626500
Br	0.39424800	-3.02620400	-0.13245300
C	-2.35071700	-1.04705100	-1.79674900
H	-1.86312100	-2.01634500	-1.96338600
H	-3.42347000	-1.15733100	-1.99257800
H	-1.96018900	-0.36171000	-2.56311900
O	-4.58849100	-2.56323700	0.16857400
C	-5.27526600	-3.77921700	-0.17890200
H	-5.38417400	-3.86369900	-1.26431000
H	-4.64959900	-4.58827900	0.20088900
H	-6.26479500	-3.81405800	0.28707500

TS4a

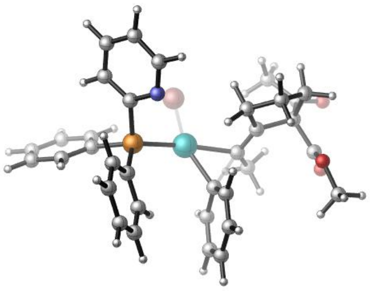

E (SMD(DMF)/B3LYP-D3/BS1) = -4636.274043
E (SMD(DMF)/M06/BS2//SMD(DMF)/B3LYP-D3/BS1) = -4639.25447

Zero-point correction=	0.597555
Thermal correction to Energy=	0.638734
Thermal correction to Enthalpy=	0.639678
Thermal correction to Gibbs Free Energy=	0.518266

Charge = -1 Multiplicity = 1

C	-2.06787000	-0.35783900	1.77887300
H	-1.65781100	-1.36197300	1.97134800
H	-1.22485700	0.33018900	1.88413000
C	-2.69287700	-0.35049000	0.39485600
C	-2.07270400	-0.04982900	-0.77577600
Pd	0.03754300	-0.30693300	-0.64118600
P	2.25691200	0.07040200	0.09145600
C	2.47771800	-0.85215500	1.68252900
C	3.41392600	-1.87373100	1.87780800
C	1.60915900	-1.15008600	3.79663500
C	3.41917000	-2.55423500	3.09714400
H	4.12157100	-2.13072400	1.09715700
C	2.49998800	-2.18896900	4.07974900
H	0.87800100	-0.83261300	4.53798600
H	4.13323800	-3.35392600	3.27482000
H	2.46760000	-2.69208300	5.04117900
C	-0.96145700	1.53063200	-0.88023300
C	-1.15000300	2.49139600	0.14618800
C	-0.81463500	2.04729100	-2.19453800
C	-1.16628800	3.85922200	-0.11623100
H	-1.29723900	2.16246500	1.16905200
C	-0.81433600	3.42080700	-2.45582800
H	-0.69553600	1.36254400	-3.02964900
C	-0.99053000	4.34239800	-1.42027900
H	-1.30905200	4.55711100	0.70675900
H	-0.67955000	3.76884200	-3.47835600
H	-0.99934900	5.41046400	-1.62197200
C	-4.19206100	-0.71346600	0.55072900
C	-4.39186400	-0.85740500	2.10187100
H	-5.38129100	-0.50923600	2.40865500
H	-4.31861200	-1.91662900	2.37449800

C	-3.22732700	-0.09000400	2.74513500
H	-3.02354800	-0.42993600	3.76679300
H	-3.45486400	0.98001900	2.77666200
C	-4.63468400	-2.05211300	-0.06953900
C	-4.98410800	0.46632100	-0.02078000
O	-5.79217100	-2.24127100	-0.40425600
O	-5.46266600	0.54237000	-1.13389300
C	-3.59519600	-3.15014700	-0.12054300
H	-2.72841500	-2.87178900	-0.73050700
H	-3.19833200	-3.33524600	0.88551400
H	-4.05318300	-4.06718500	-0.50118800
O	-5.01458900	1.49976100	0.85313300
C	-5.56611800	2.72851900	0.34788600
H	-5.51940500	3.43410500	1.17891500
H	-4.97068300	3.09736100	-0.49320700
H	-6.60243000	2.59038000	0.02622100
N	1.58611200	-0.49598400	2.62960900
C	2.71158600	1.79055300	0.58171900
C	2.28294100	2.83479100	-0.25372200
C	3.46052000	2.10356700	1.72845700
C	2.60275500	4.16119500	0.04390600
H	1.68243100	2.61220100	-1.13074300
C	3.77231000	3.43147900	2.02999000
H	3.80635700	1.31317900	2.38797200
C	3.34557900	4.46310800	1.18823200
H	2.25645300	4.95651800	-0.61073000
H	4.35086700	3.65928100	2.92173900
H	3.58686200	5.49584300	1.42633000
C	3.71746700	-0.46023400	-0.91107100
C	3.53812200	-1.52445400	-1.81151100
C	4.98331600	0.14019200	-0.80754400
C	4.60791400	-1.98063600	-2.58608600
H	2.56310500	-2.00217200	-1.88600600
C	6.04771700	-0.31335800	-1.58989500
H	5.14279500	0.96345800	-0.11774100
C	5.86304600	-1.37503200	-2.48050600
H	4.45816500	-2.80708100	-3.27670600
H	7.02125600	0.16256100	-1.50259300
H	6.69238200	-1.72612900	-3.08931700
Br	0.12709000	-2.97014000	-1.17320900
C	-2.74327500	-0.23204000	-2.12178300
H	-2.01206300	-0.55631000	-2.86968100
H	-3.53510500	-0.98566900	-2.08924300
H	-3.20332600	0.69350500	-2.48522300

TS4c

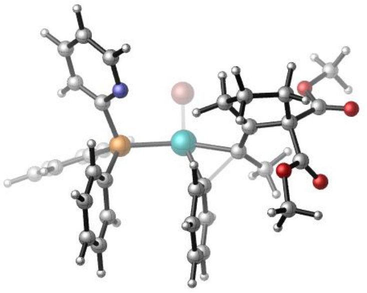

E (SMD(DMF)/B3LYP-D3/BS1) = -4711.502211
E (SMD(DMF)/M06/BS2//SMD(DMF)/B3LYP-D3/BS1) = -4714.475384

Zero-point correction=	0.603617
Thermal correction to Energy=	0.645417
Thermal correction to Enthalpy=	0.646361
Thermal correction to Gibbs Free Energy=	0.524618

Charge = -1 Multiplicity = 1

C	-2.06830200	-0.45697000	1.76695700
H	-1.90025000	-1.52331700	1.99023400
H	-1.08426100	0.01579000	1.84510400
C	-2.67609300	-0.35458700	0.38054100
C	-1.98877600	-0.14501500	-0.77874400
Pd	0.14056800	-0.53845300	-0.60132300
P	2.33826300	0.00947000	0.13503200
C	2.66892400	-0.77230600	1.78050600
C	3.78058100	-1.57651800	2.05981100
C	1.78796500	-1.15022200	3.87903200
C	3.86803100	-2.19082000	3.31041100
H	4.55925300	-1.72086200	1.31929400
C	2.85443900	-1.97564900	4.24324400
H	0.97711600	-0.95397300	4.57813000
H	4.71755200	-2.82464900	3.55027300
H	2.88147800	-2.43451400	5.22672100
C	-0.80771600	1.31967900	-0.84732400
C	-0.92962400	2.27267300	0.20145000
C	-0.64105100	1.85911500	-2.15118700
C	-0.89576600	3.64240300	-0.03542100
H	-1.07105500	1.93191800	1.22037400
C	-0.60639900	3.23707100	-2.38827400
H	-0.55132000	1.18835700	-3.00076100
C	-0.74057500	4.14473400	-1.33634100
H	-0.99014600	4.32863200	0.80386000
H	-0.47237500	3.59771900	-3.40652700
H	-0.71697200	5.21603100	-1.51820600
C	-4.21822600	-0.31463800	0.54555000
C	-4.43934100	-0.38412400	2.09806200
H	-5.32099000	0.18572600	2.40296800
H	-4.60893100	-1.43197200	2.37409700

C	-3.12497100	0.09629200	2.72827200
H	-3.00642900	-0.26738300	3.75499100
H	-3.09025700	1.18934100	2.74271300
C	-5.07439400	-1.43038200	-0.04766500
C	-4.66036000	1.03040700	-0.04502000
O	-6.27925400	-1.35913500	-0.18556800
O	-5.16591900	1.19444900	-1.13741300
O	-4.33133400	2.05275200	0.77087600
C	-4.52044600	3.37205200	0.22716300
H	-4.17458300	4.05911500	1.00039100
H	-3.92863700	3.50084400	-0.68293100
H	-5.57667900	3.55145800	0.00561900
N	1.68277500	-0.56802500	2.67835200
C	2.56013600	1.81638800	0.45401900
C	2.65390400	2.65325700	-0.67369600
C	2.53031800	2.40411400	1.72702300
C	2.73667800	4.03734100	-0.52971600
H	2.66173400	2.22009200	-1.67063400
C	2.60540800	3.79361600	1.86844600
H	2.43944300	1.78607000	2.61353600
C	2.71045500	4.61447600	0.74426200
H	2.80773800	4.66645700	-1.41309000
H	2.58266600	4.23143200	2.86335500
H	2.76668000	5.69394500	0.85721400
C	3.85593100	-0.38052300	-0.84122500
C	3.77090900	-1.36812000	-1.83489500
C	5.08356600	0.27057200	-0.62577700
C	4.89482200	-1.69762700	-2.59872700
H	2.82979300	-1.89181700	-1.98532100
C	6.20276700	-0.05929800	-1.39141600
H	5.16444400	1.03815600	0.13886500
C	6.10969800	-1.04339500	-2.38162000
H	4.81844400	-2.46445100	-3.36582600
H	7.14631400	0.45158000	-1.21669900
H	6.98132000	-1.29717500	-2.97962700
Br	0.64561500	-3.23298300	-0.98226300
C	-2.67316200	-0.26755400	-2.12893700
H	-3.46163400	-1.02546400	-2.09980900
H	-3.12699600	0.67147000	-2.46227500
H	-1.95656200	-0.57639100	-2.89751100
O	-4.36758200	-2.54833100	-0.28667800
C	-5.13243800	-3.68738200	-0.72845300
H	-5.64481300	-3.46522300	-1.66888800
H	-4.40633100	-4.48836700	-0.87294800
H	-5.86940900	-3.97346500	0.02782100

VIIIa

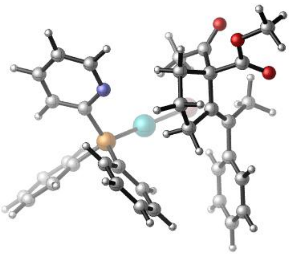

E (SMD(DMF)/B3LYP-D3/BS1) = -4636.350897
E (SMD(DMF)/M06/BS2//SMD(DMF)/B3LYP-D3/BS1) = -4639.328461

Zero-point correction=	0.601836
Thermal correction to Energy=	0.643235
Thermal correction to Enthalpy=	0.644179
Thermal correction to Gibbs Free Energy=	0.522074

Charge = -1 Multiplicity = 1

C	2.00523400	0.58374300	1.80988600
H	0.93115200	0.61760100	1.59268800
H	2.12828700	-0.03389100	2.70114500
C	2.73933800	0.03974000	0.58689300
C	2.76951200	-1.24930800	0.20103200
Pd	-1.27309300	-0.88526700	-1.48650800
P	-2.32479800	0.37220500	0.01621200
C	-2.18362900	2.20073300	-0.30298400
C	-3.27431500	3.02700600	-0.60075100
C	-0.70704300	3.95251500	-0.56179100
C	-3.04002000	4.37171400	-0.89522000
H	-4.28390100	2.63168300	-0.60154200
C	-1.73117900	4.85010000	-0.87419300
H	0.32858100	4.28622600	-0.53534300
H	-3.86949300	5.03254200	-1.13236400
H	-1.50203700	5.88793100	-1.09543200
C	2.12563800	-2.31871000	1.02829000
C	2.58442900	-2.59895300	2.32644100
C	1.09106800	-3.10893000	0.49981900
C	2.00794100	-3.61761800	3.08812500
H	3.40513900	-2.01591300	2.73612100
C	0.50920800	-4.12404900	1.26409800
H	0.73276600	-2.91999600	-0.50841400
C	0.96132700	-4.38128100	2.56161300
H	2.37840300	-3.81666300	4.09078200
H	-0.30103600	-4.71437300	0.84220600
H	0.50942100	-5.17318700	3.15354800
C	3.41986400	1.22138800	-0.13046900
C	2.76917900	2.46841400	0.54764300
H	3.38470100	3.36219800	0.44387800
H	1.79973600	2.65962600	0.07886700

C	2.53460100	2.01221600	1.99057100
H	1.83296200	2.65890000	2.52790300
H	3.48381900	2.00680400	2.54318800
C	3.18676700	1.31694900	-1.65289900
C	4.94512800	1.16735500	0.08955300
O	4.10833200	1.58492700	-2.40909800
O	5.59907200	0.14549600	0.13945100
C	1.78450900	1.09198000	-2.16415400
H	1.66518100	0.03767200	-2.44543000
H	1.01026100	1.31087900	-1.42556900
H	1.62859700	1.69741900	-3.06284400
O	5.49235300	2.39003800	0.19738200
C	6.92657900	2.42071000	0.32683500
H	7.19176200	3.47605000	0.40478700
H	7.24546300	1.88175100	1.22367100
H	7.39948600	1.97374000	-0.55246000
N	-0.91591800	2.65973600	-0.28833600
C	-1.70741700	0.25259900	1.75822300
C	-1.32691200	-1.01702100	2.22215200
C	-1.58833100	1.35295500	2.62290700
C	-0.84141200	-1.18679600	3.51942500
H	-1.38940900	-1.87219200	1.55532600
C	-1.08713400	1.18517200	3.91674300
H	-1.87433500	2.34588700	2.29070700
C	-0.71219800	-0.08343500	4.36788100
H	-0.54268000	-2.17554300	3.85503500
H	-0.99043100	2.04755300	4.57147300
H	-0.31757900	-0.21029100	5.37263800
C	-4.15392400	0.16336200	0.23792700
C	-4.88976900	-0.42061700	-0.80462500
C	-4.83409900	0.58866400	1.39156700
C	-6.27526000	-0.57091000	-0.70301400
H	-4.36904700	-0.76019900	-1.69721300
C	-6.21740200	0.43221000	1.49664900
H	-4.28536400	1.04199700	2.21208400
C	-6.94140100	-0.14647600	0.44924200
H	-6.83057500	-1.02466500	-1.51987200
H	-6.73039100	0.76194100	2.39658300
H	-8.01806600	-0.26922500	0.53386700
Br	0.00373100	-2.28778800	-3.17052000
C	3.41946800	-1.76601300	-1.06202900
H	2.65378900	-2.14964200	-1.75090600
H	4.00835100	-1.00931300	-1.58072700
H	4.09341000	-2.59977300	-0.82581900

Copies of ^{1}H and $^{13}C\{^{1}H\}$ NMR spectra of isolated compounds

Methyl 2-cyanooct-6-ynoate (39)

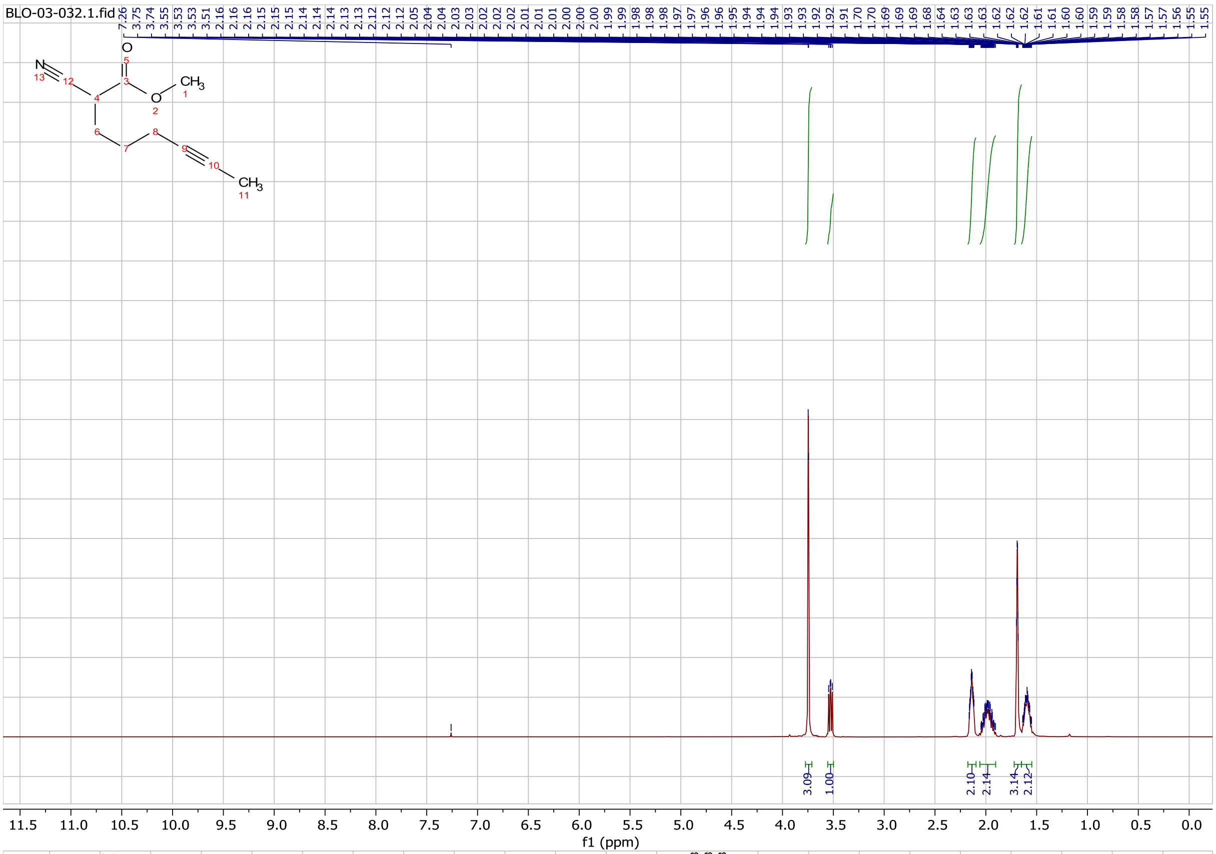

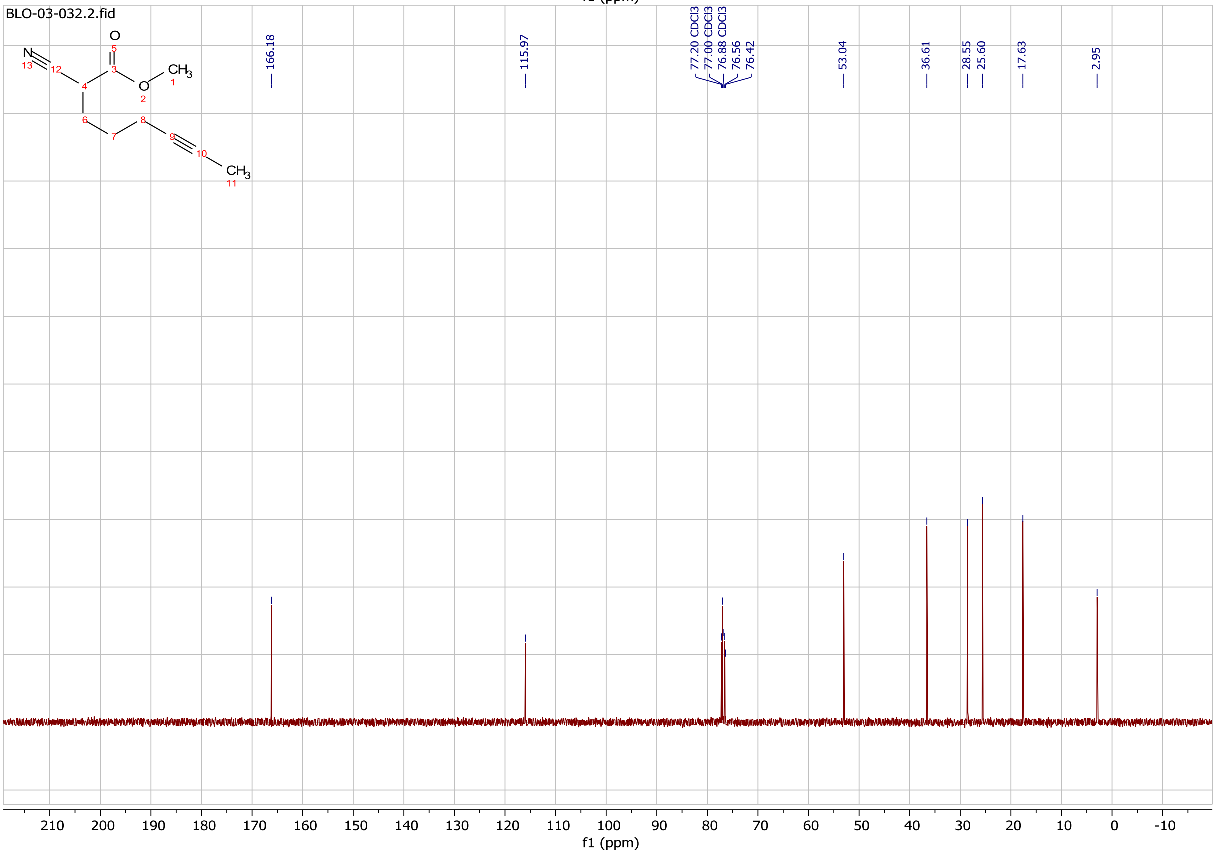

Methyl 2-isobutyryloct-6-ynoate (40)

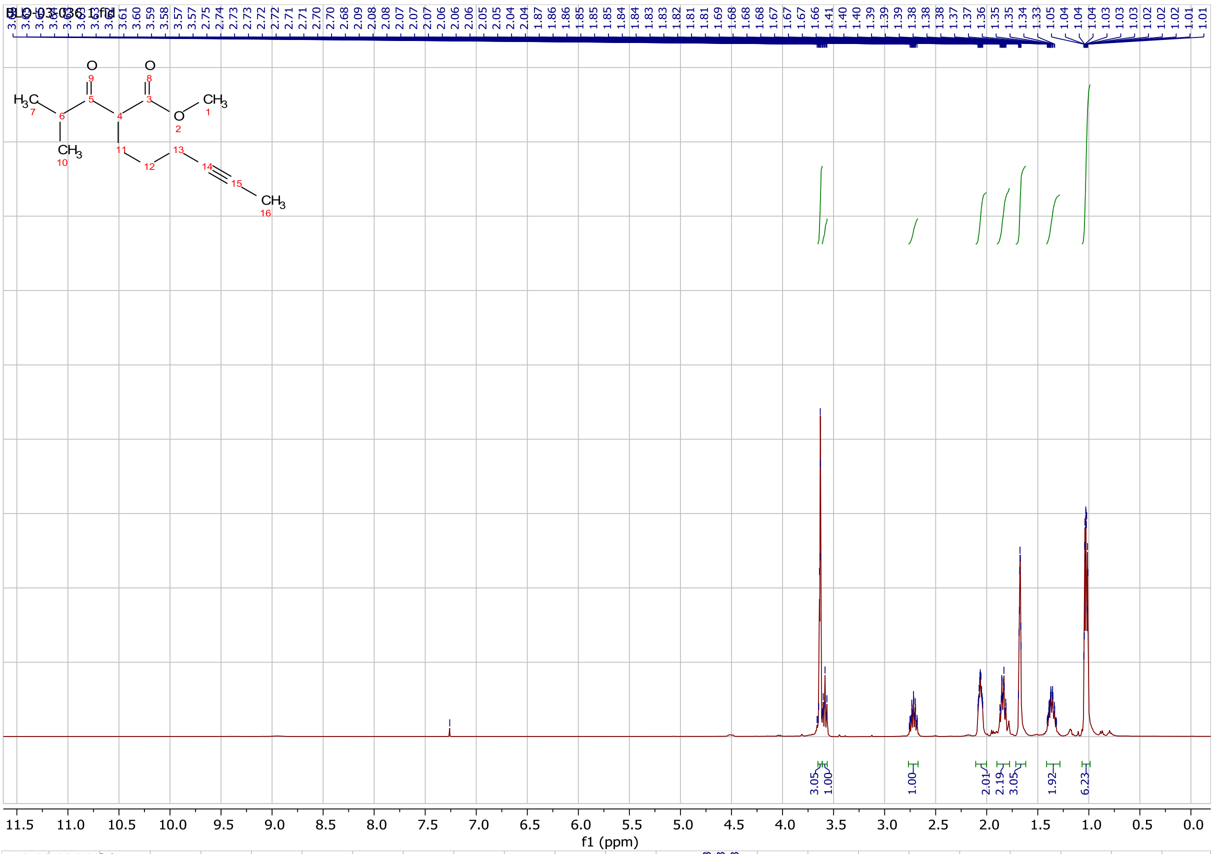

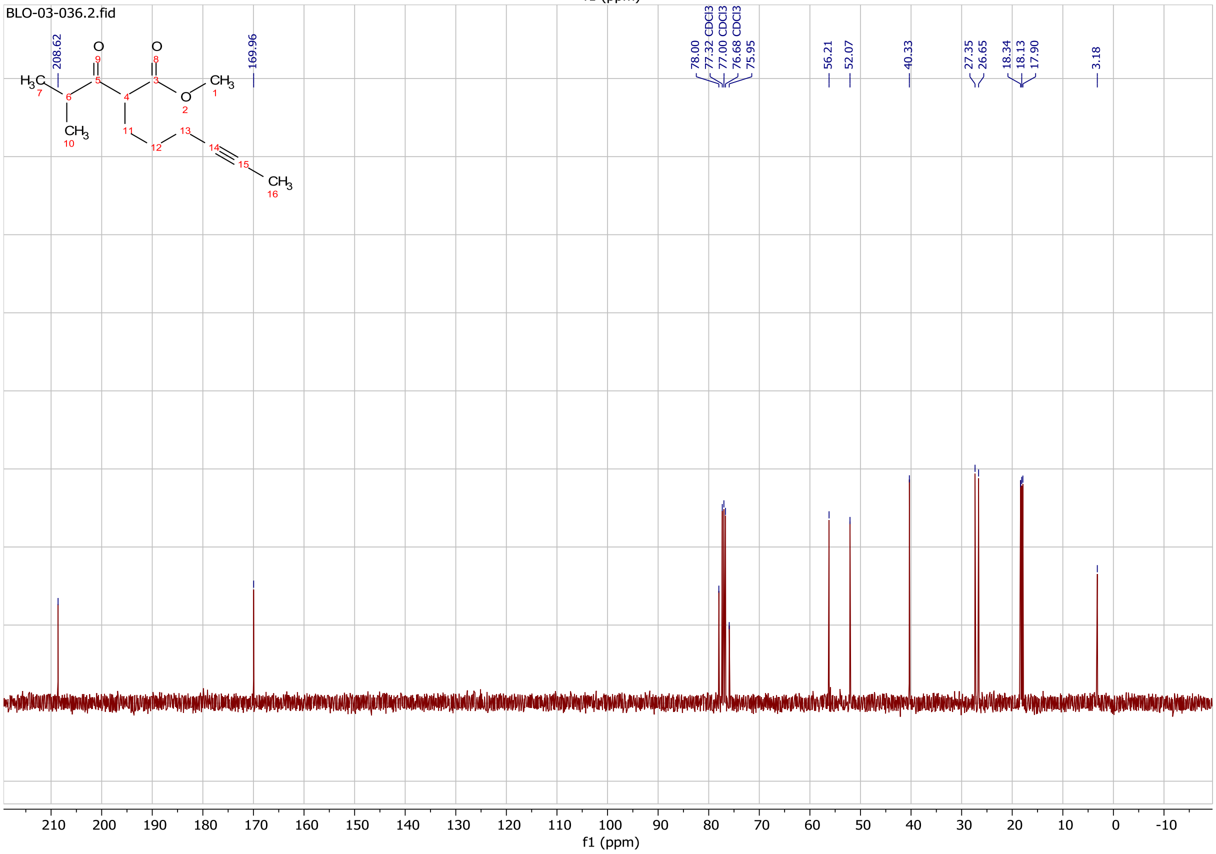

Isopropyl 2-acetyloct-6-ynoate (41)

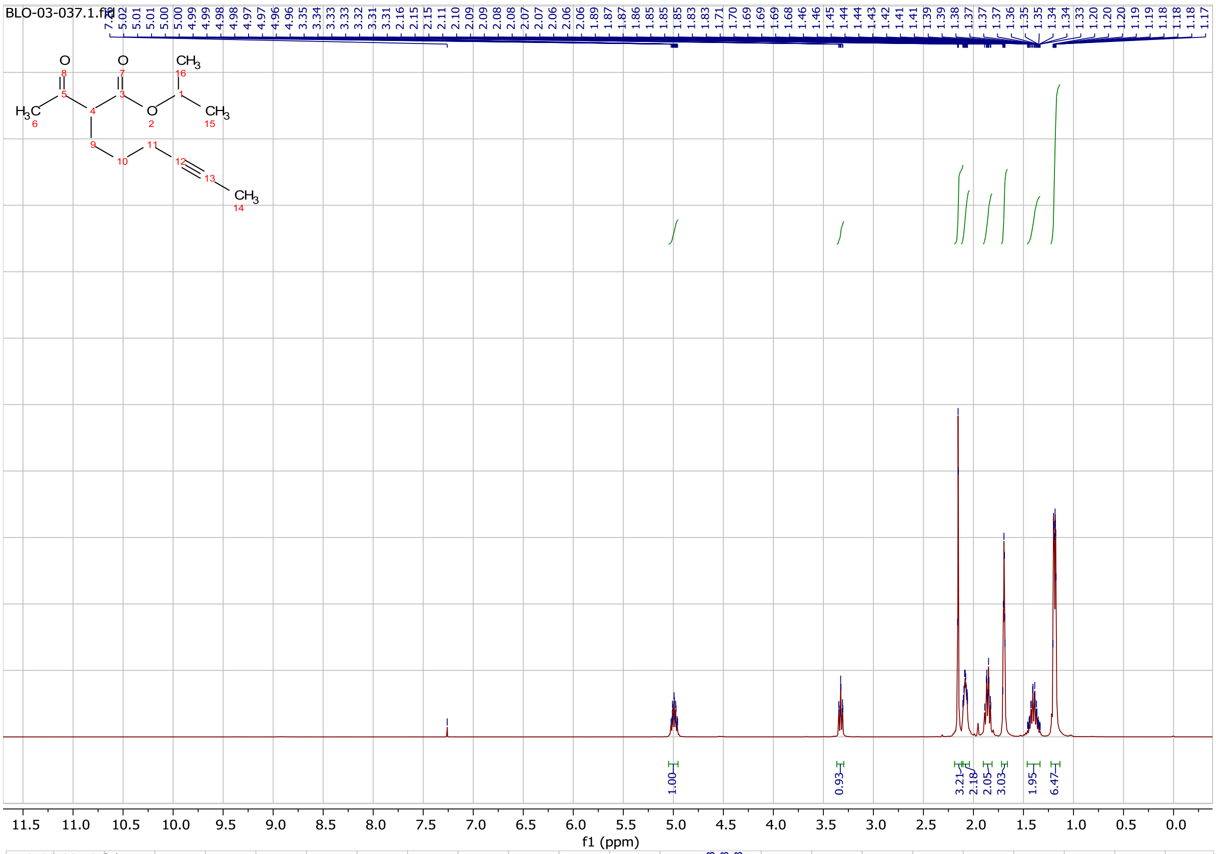

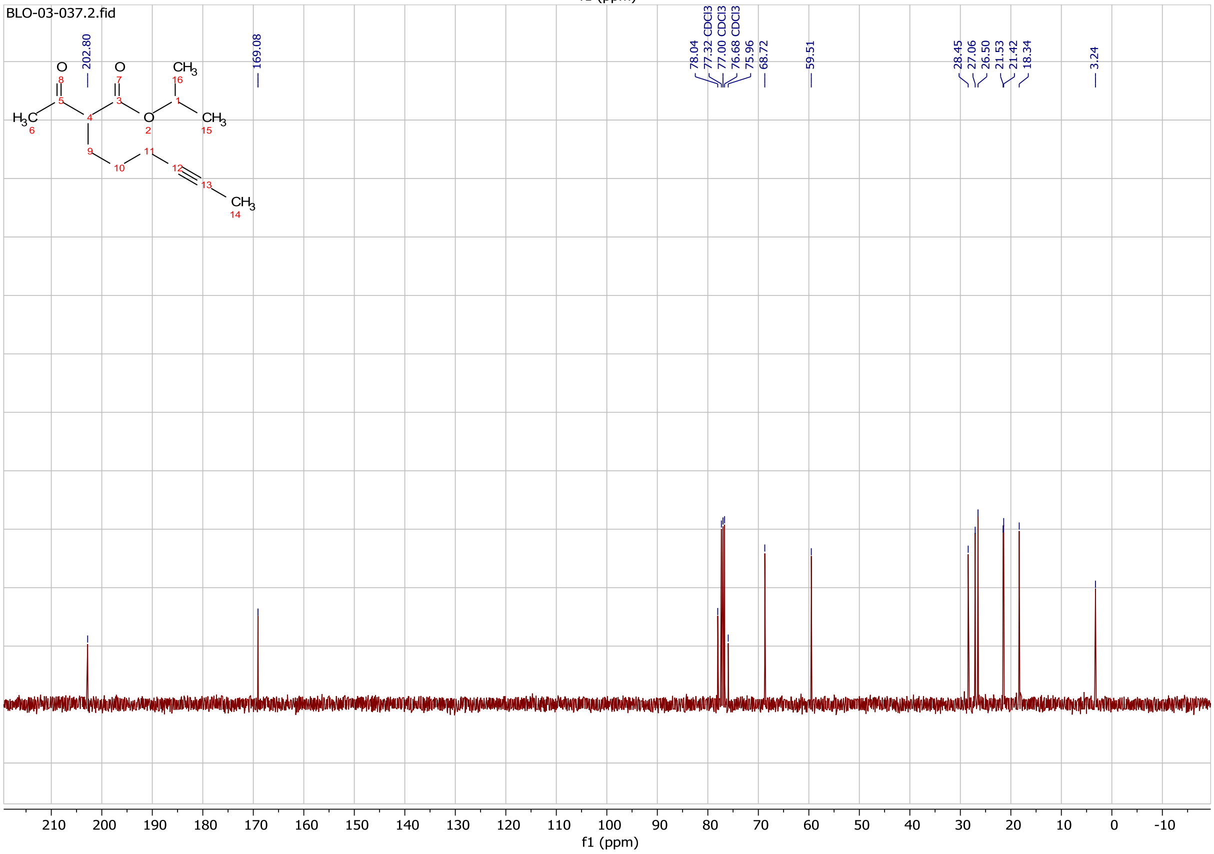

3-(phenylsulfonyl)non-7-yn-2-one (42)

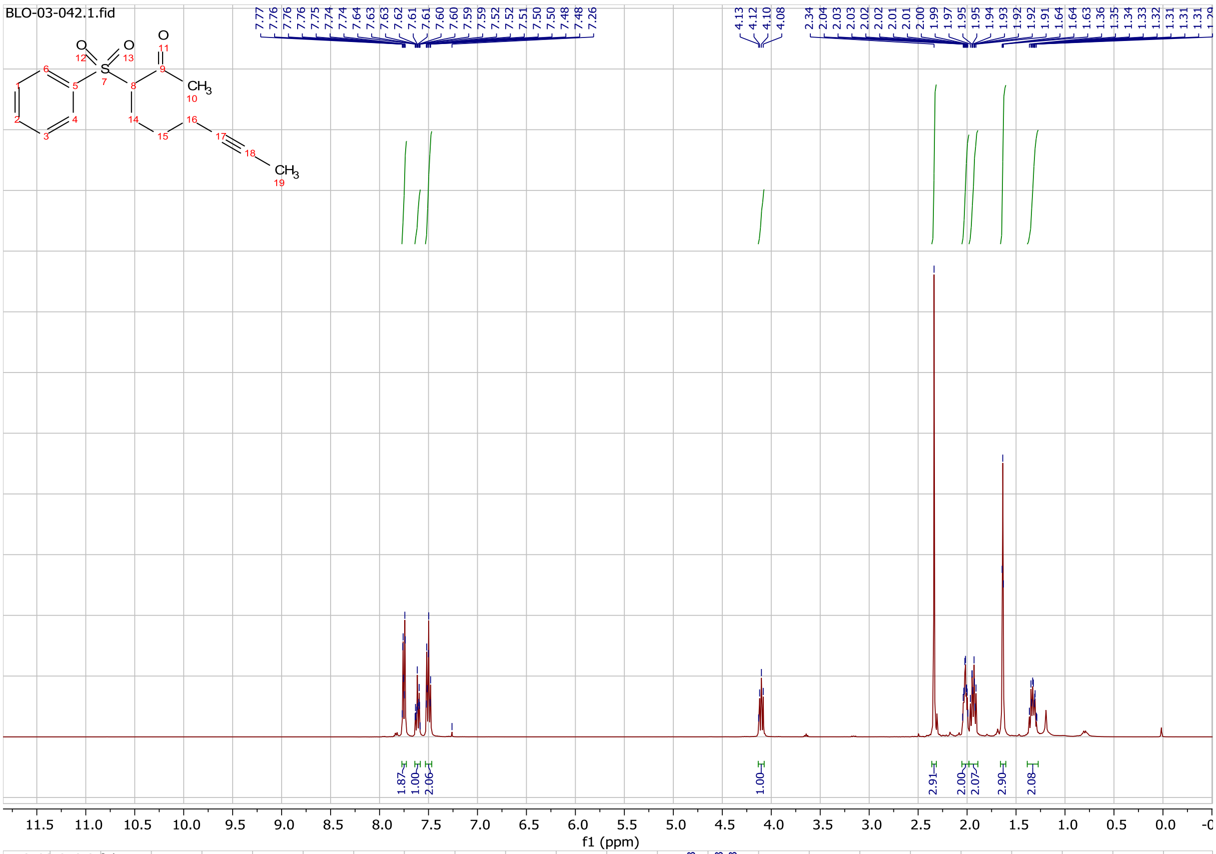

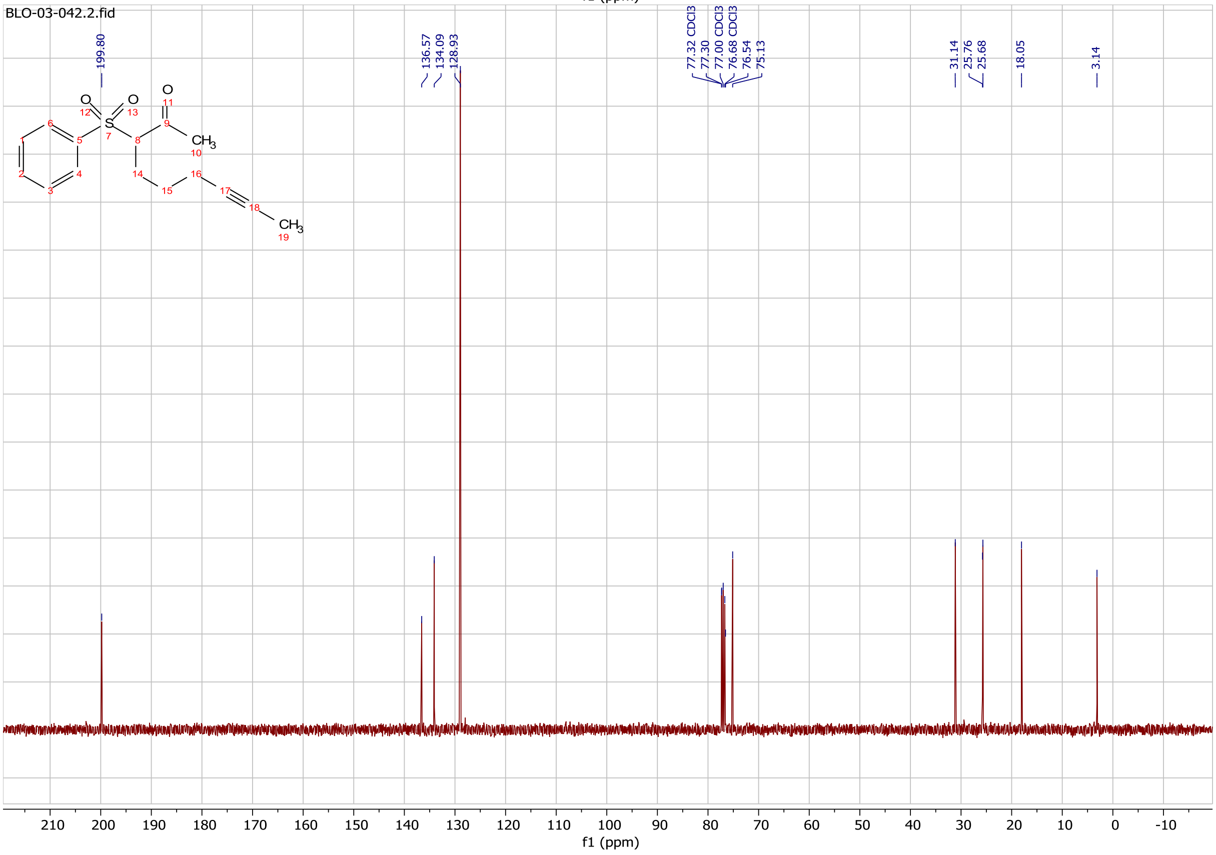

Ethyl 2-(methylsulfonyl)oct-6-ynoate (43)

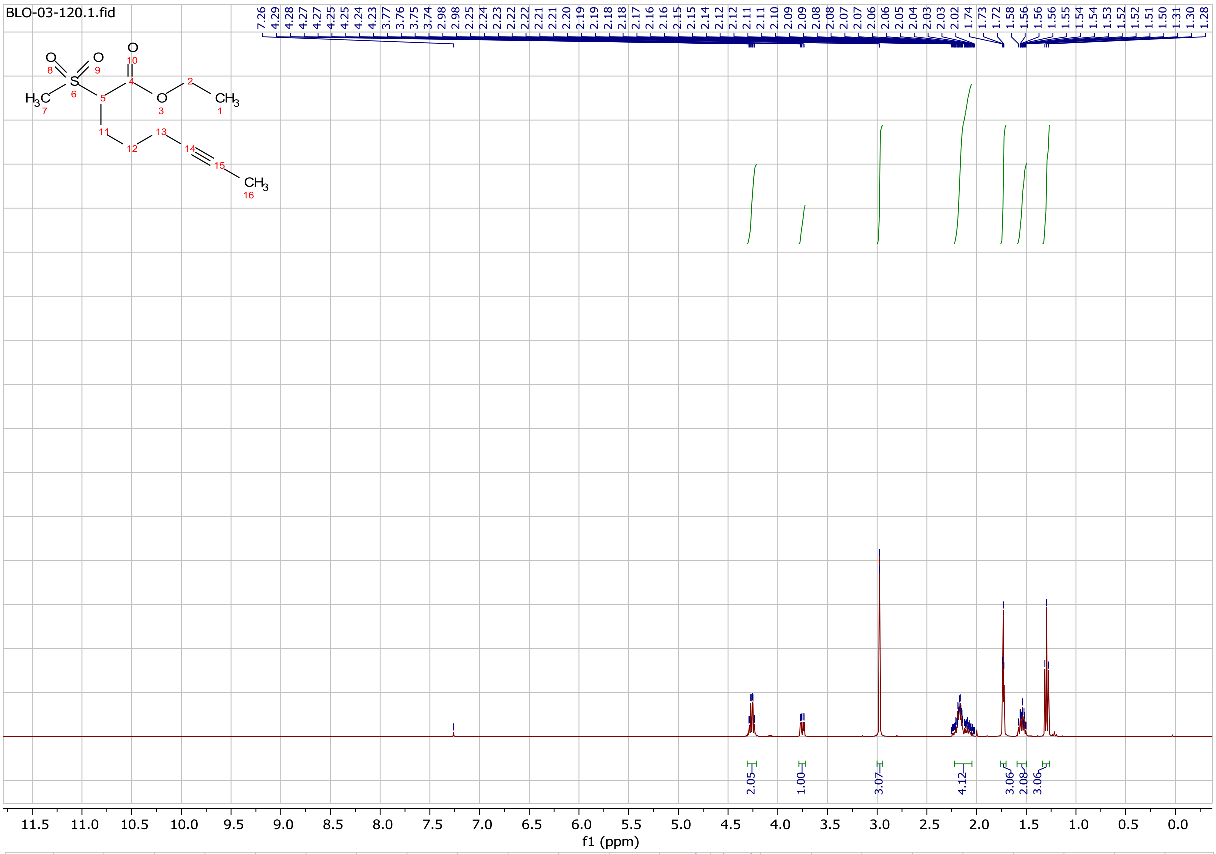

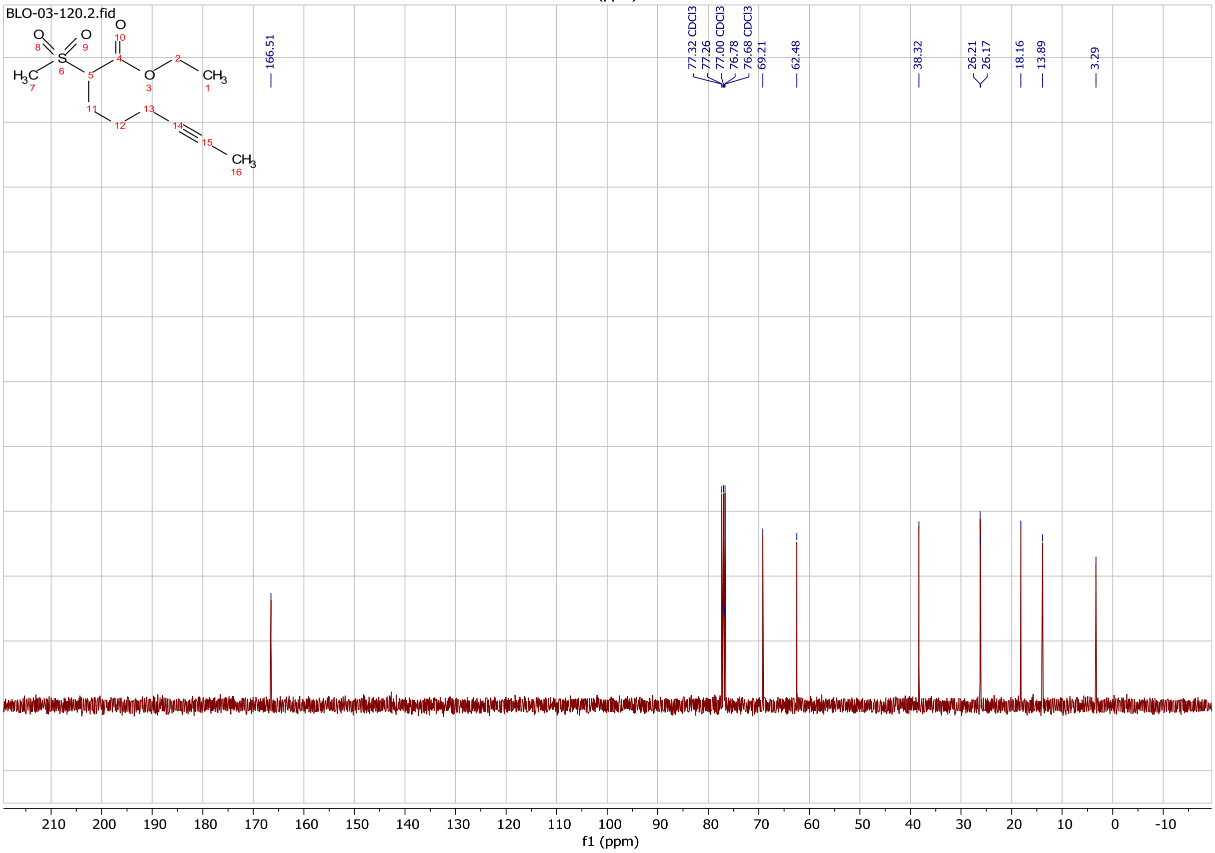

Dimethyl *(E)*-2-(1-phenylethylidene)cyclopentane-1,1-dicarboxylate (2)

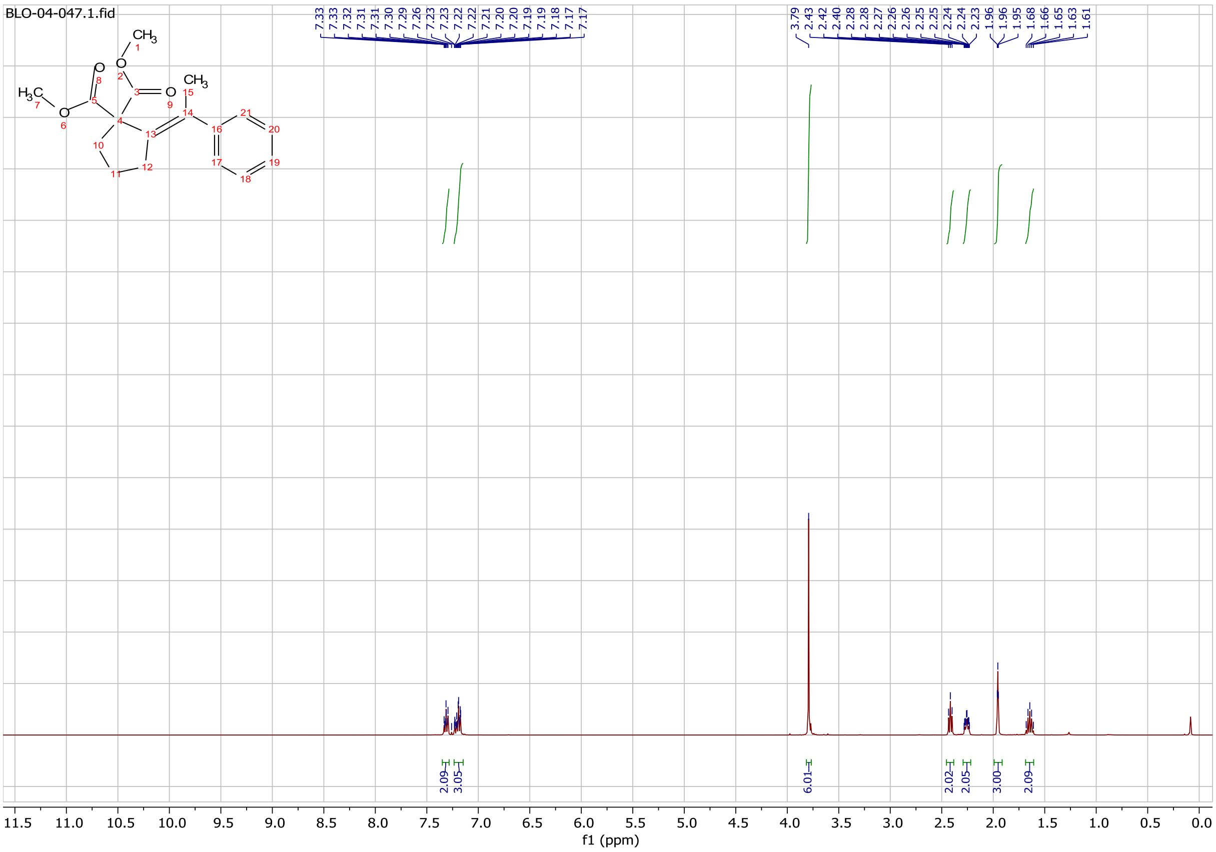

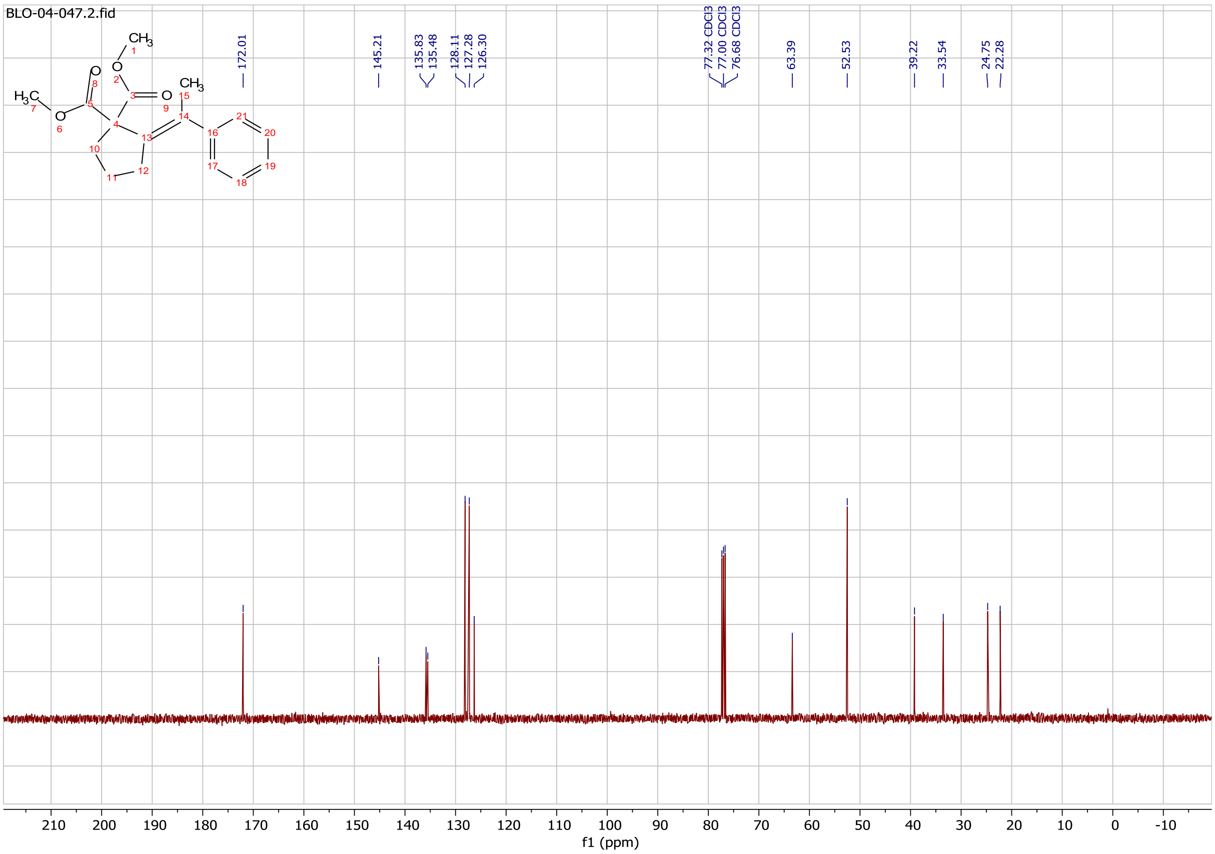

Dimethyl *(E)*-2-(1-(4-methoxyphenyl)ethylidene)cyclopentane-1,1-dicarboxylate (3)

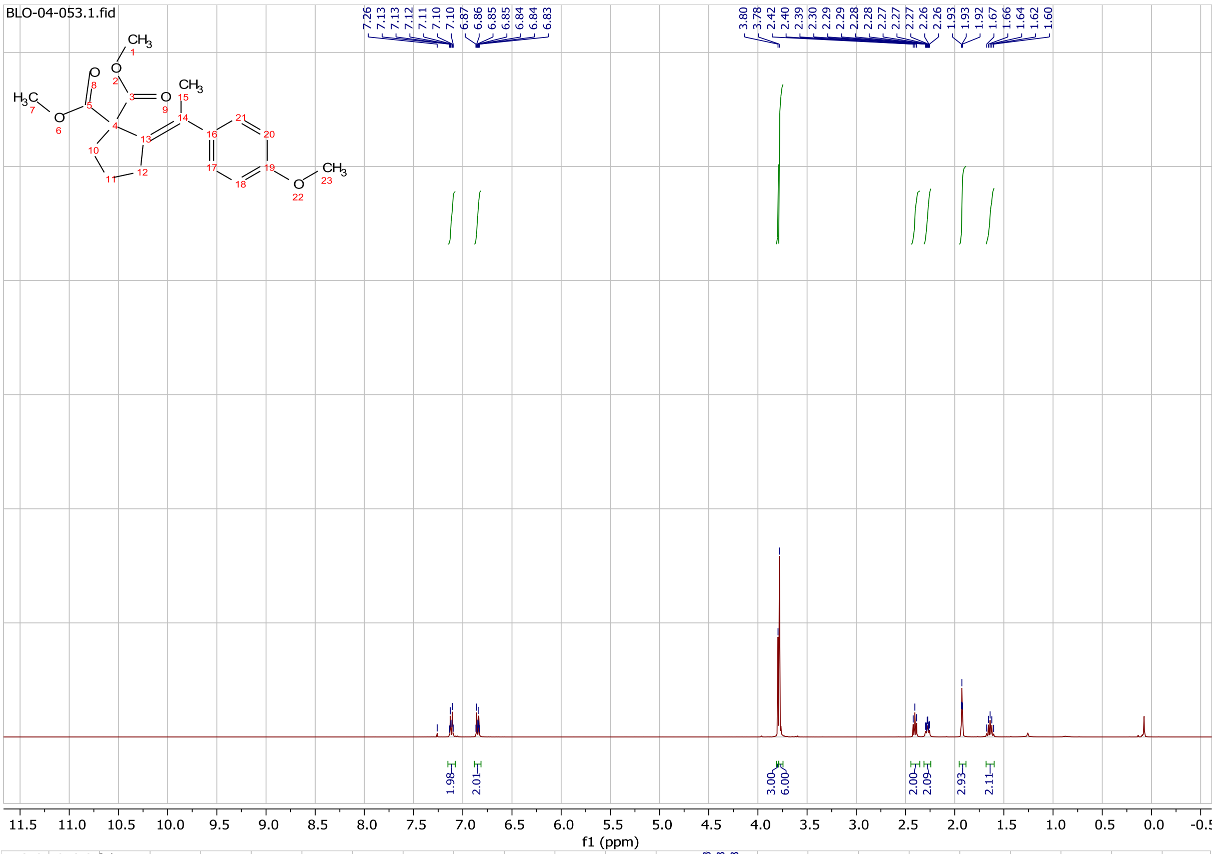

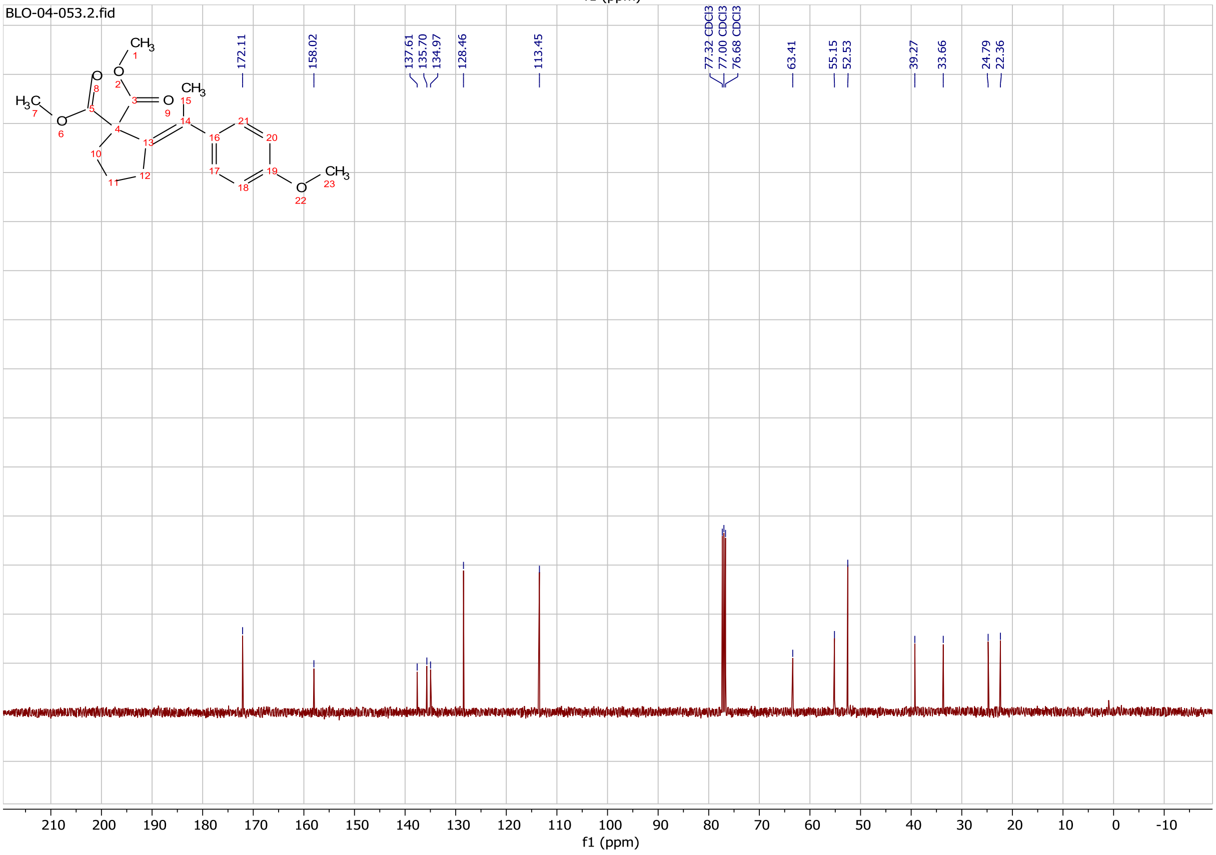

Dimethyl *(E)*-2-(1-(4-cyanophenyl)ethylidene)cyclopentane-1,1-dicarboxylate (4)

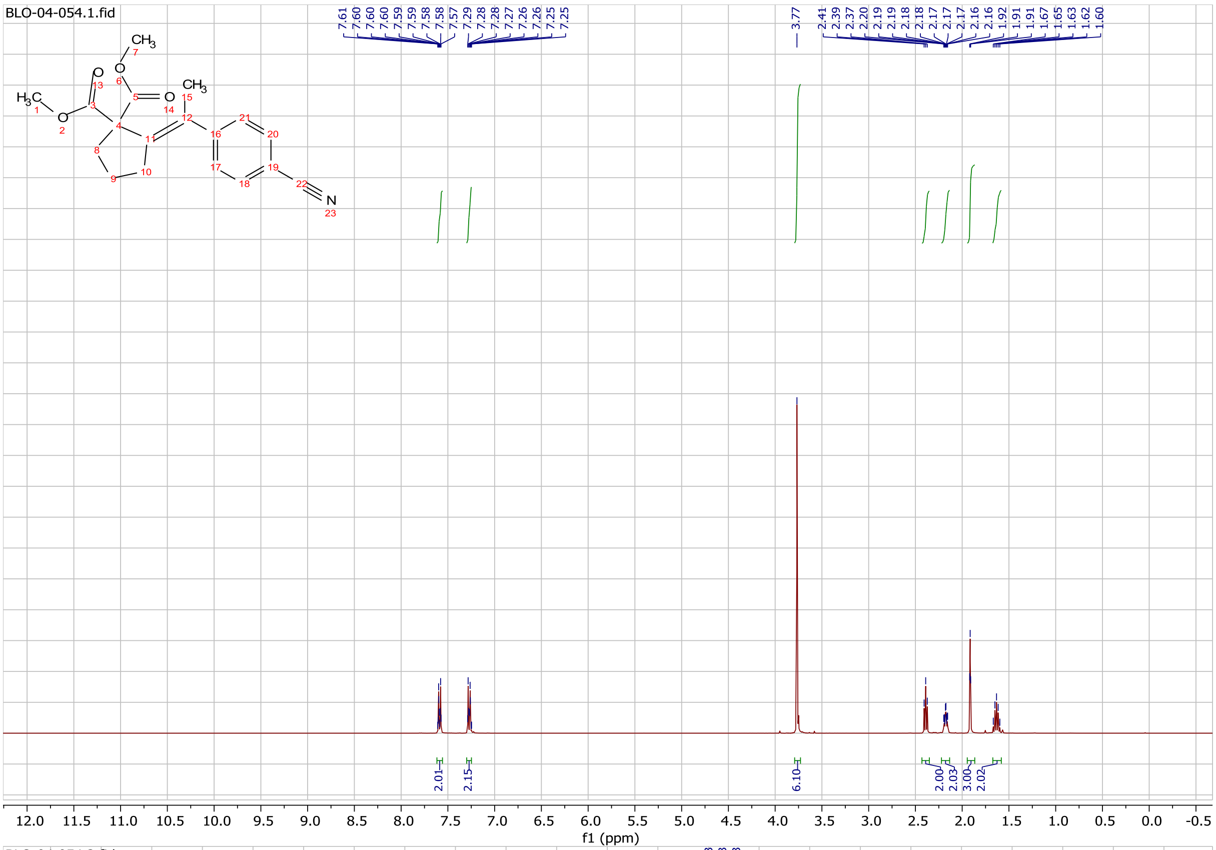

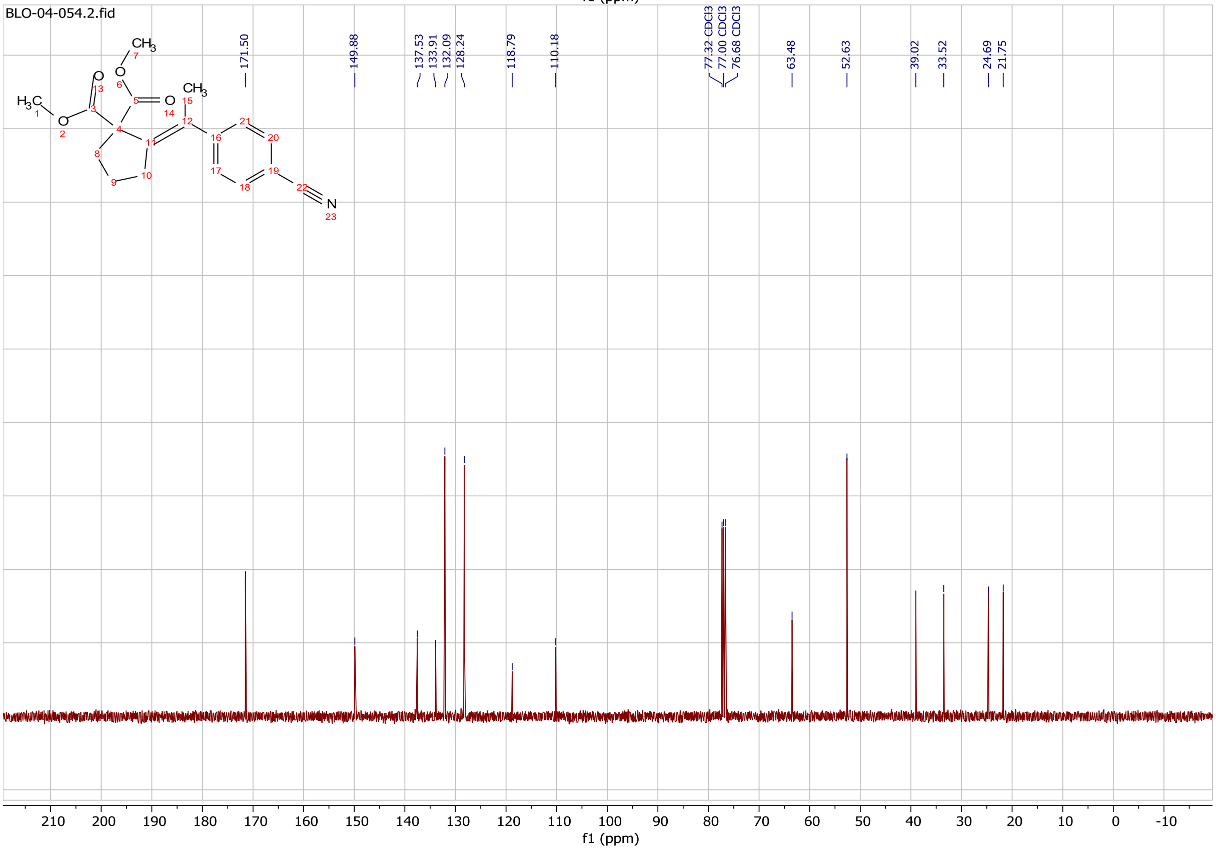

Dimethyl *(E)*-2-(1-(4-(trifluoromethyl)phenyl)ethylidene)cyclopentane-1,1-dicarboxylate (5)

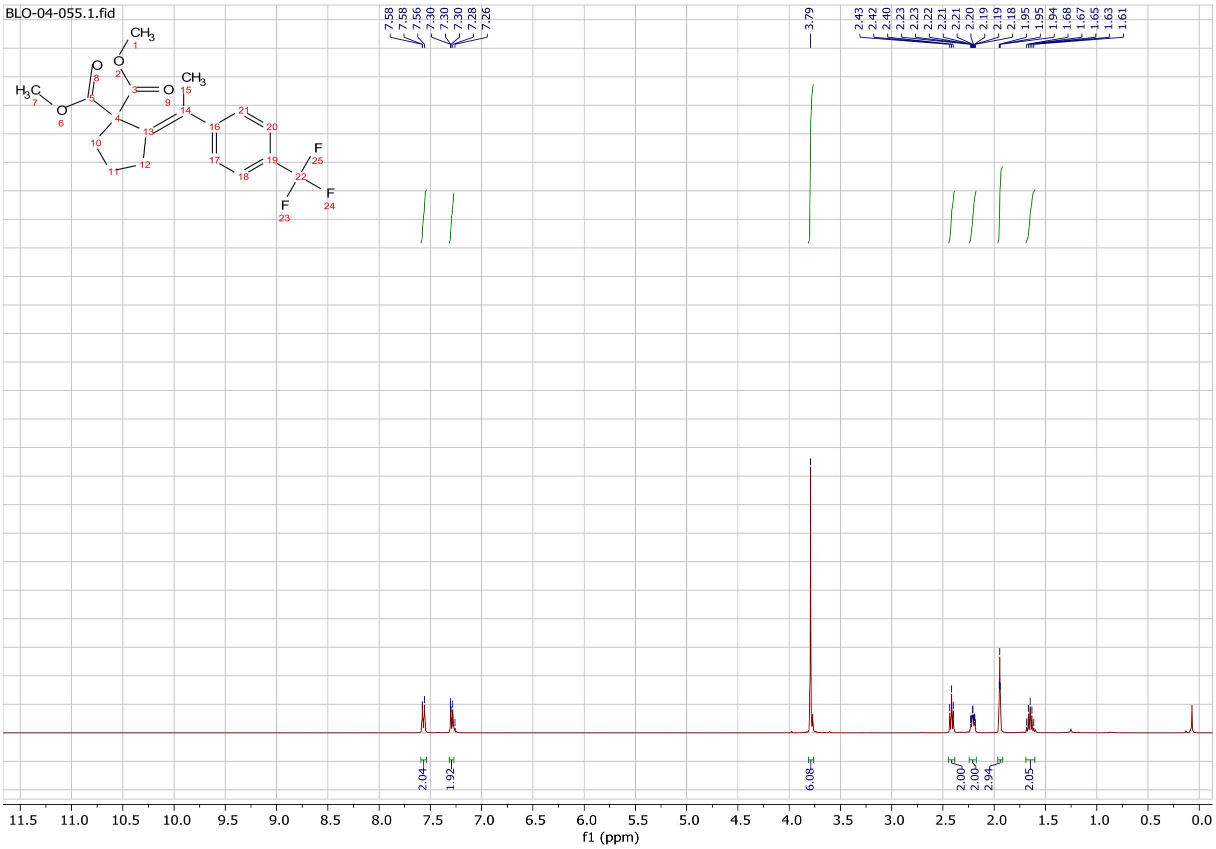

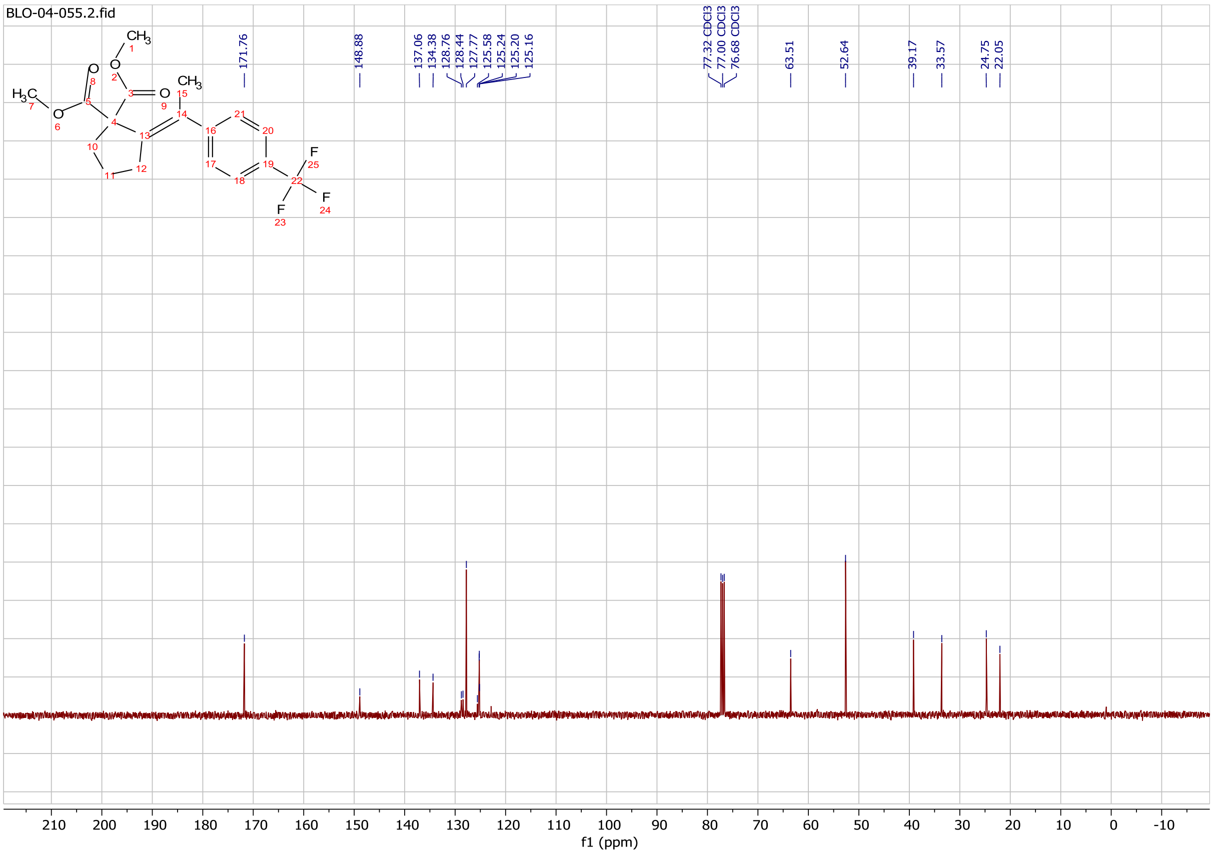

Dimethyl *(E)*-2-(1-(4-nitrophenyl)ethylidene)cyclopentane-1,1-dicarboxylate (6)

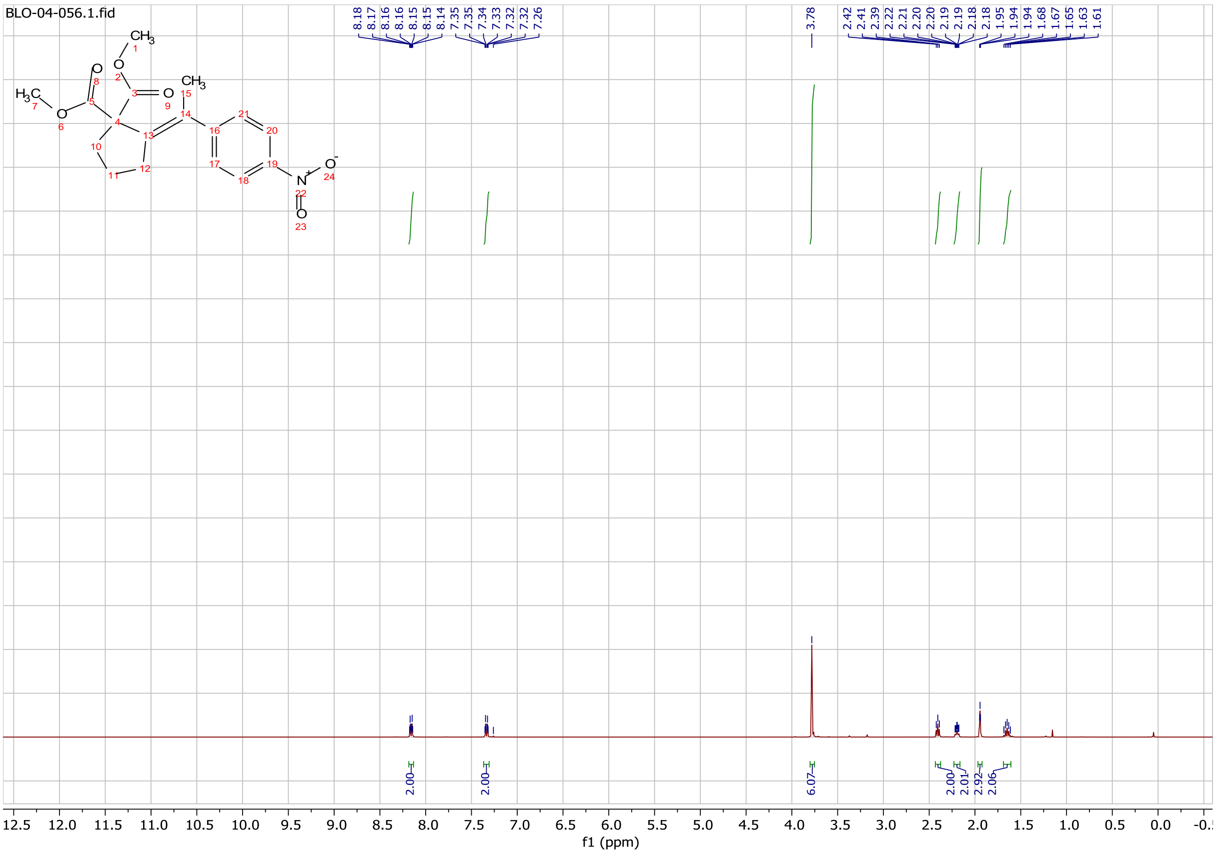

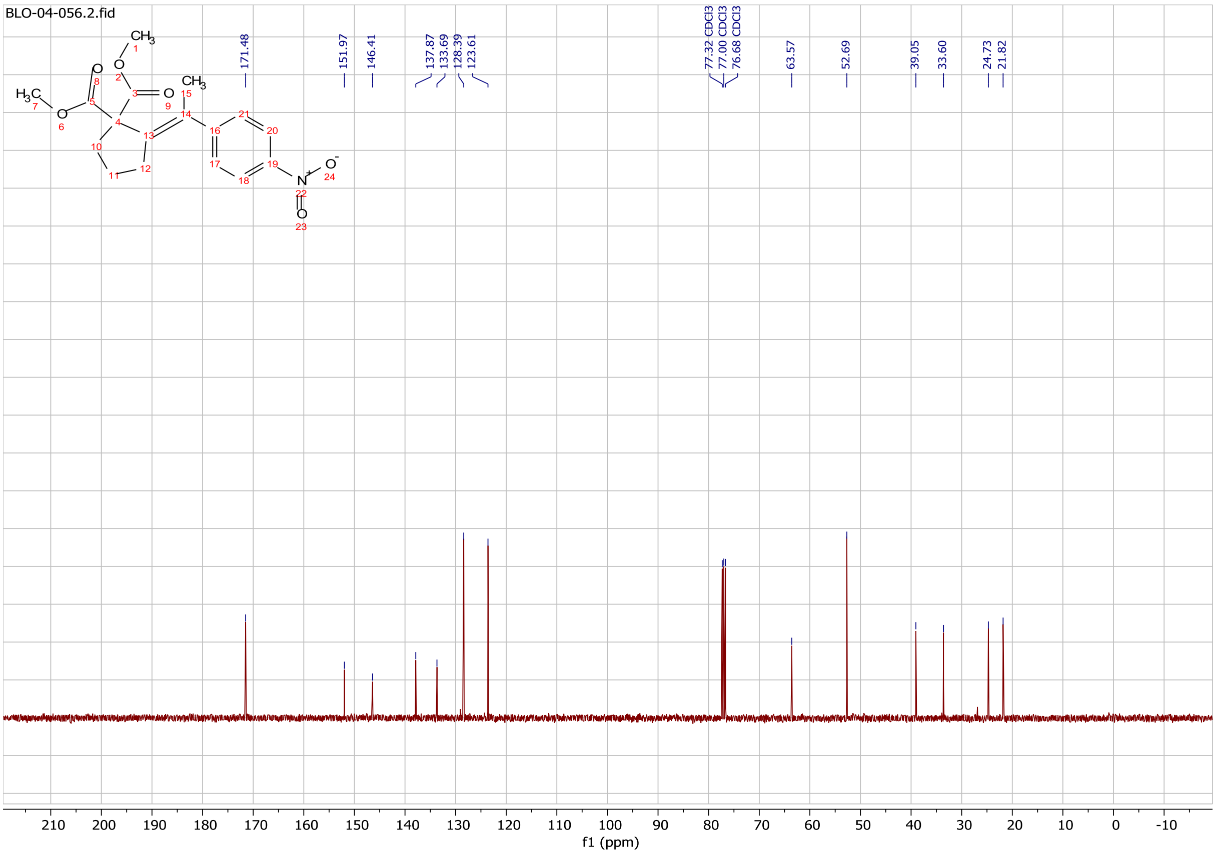

Dimethyl *(E)*-2-(1-(4-formylphenyl)ethylidene)cyclopentane-1,1-dicarboxylate (7)

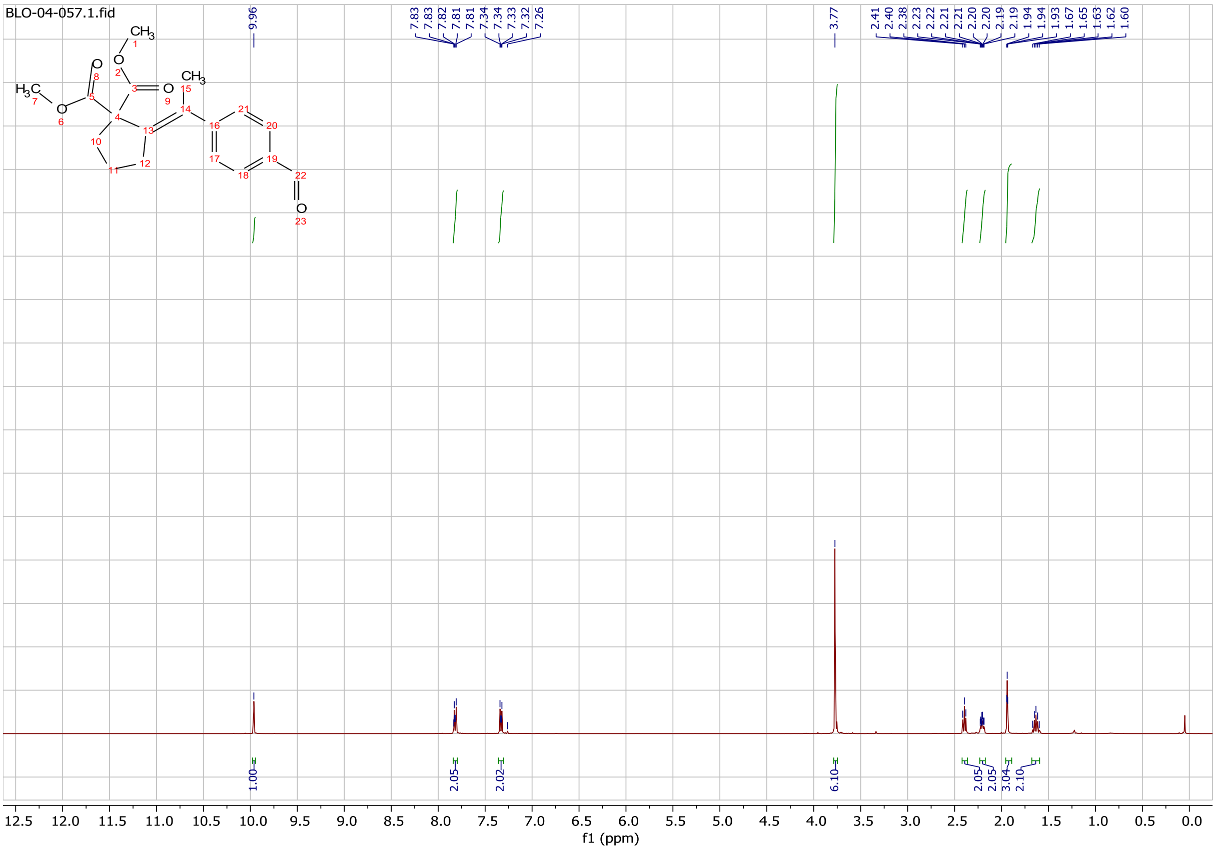

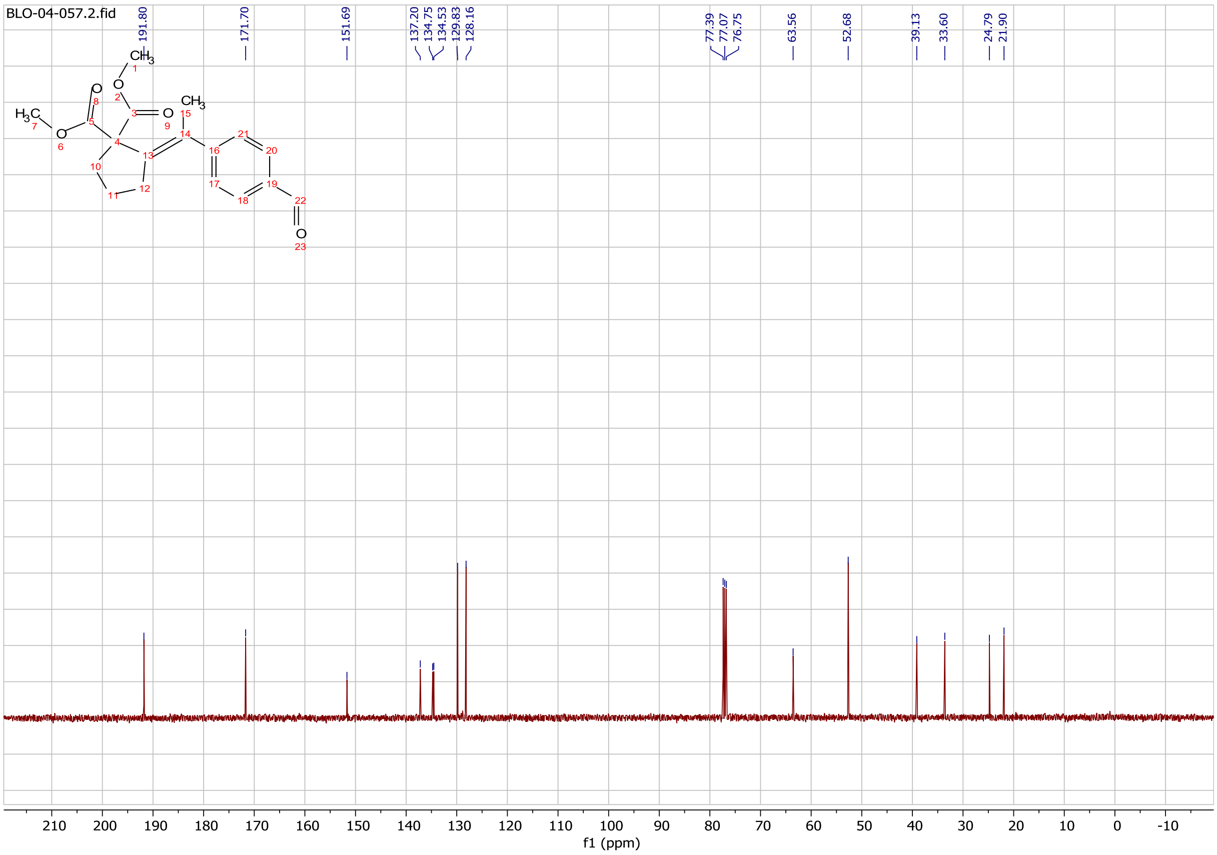

Dimethyl *(E)*-2-(1-(4-acetylphenyl)ethylidene)cyclopentane-1,1-dicarboxylate (8)

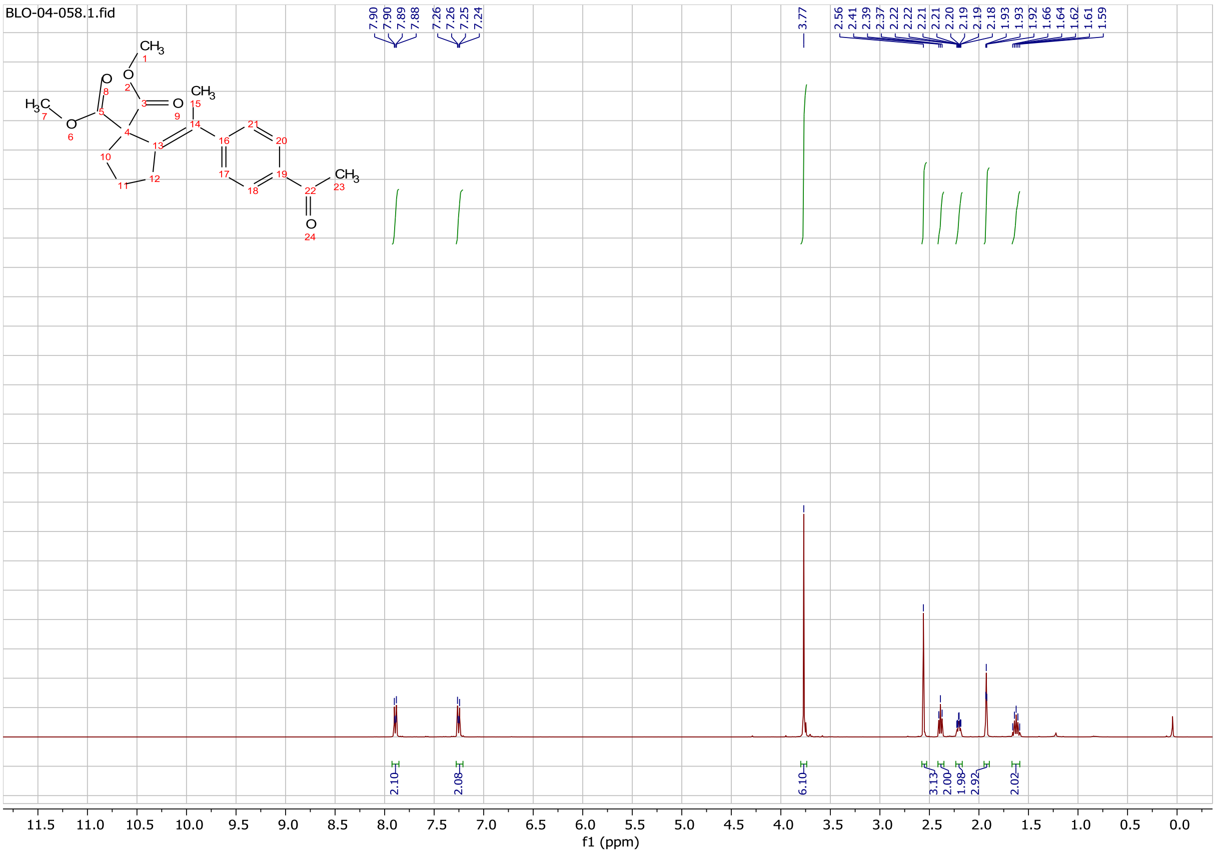

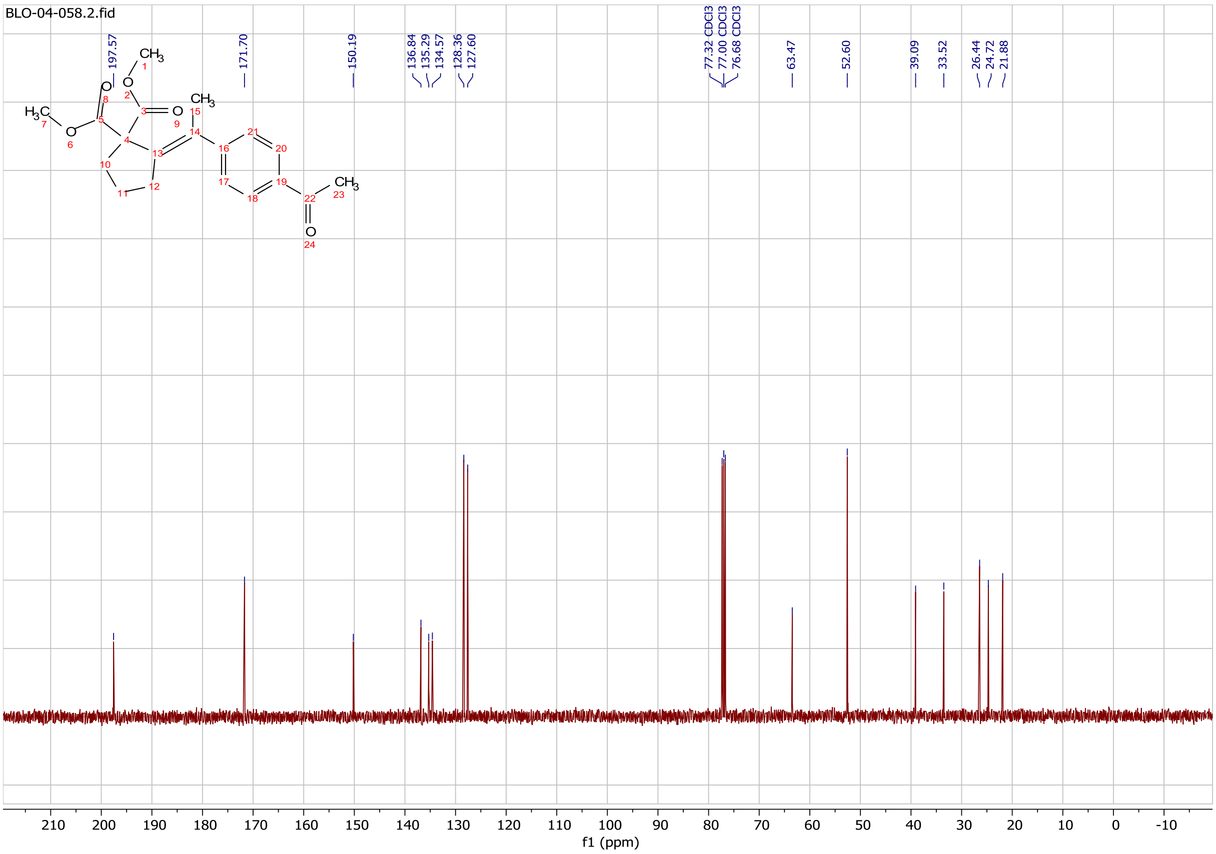

Dimethyl *(E)*-2-(1-(4-(methoxycarbonyl)phenyl)ethylidene) cyclopentane-1,1-dicarboxylate (9)

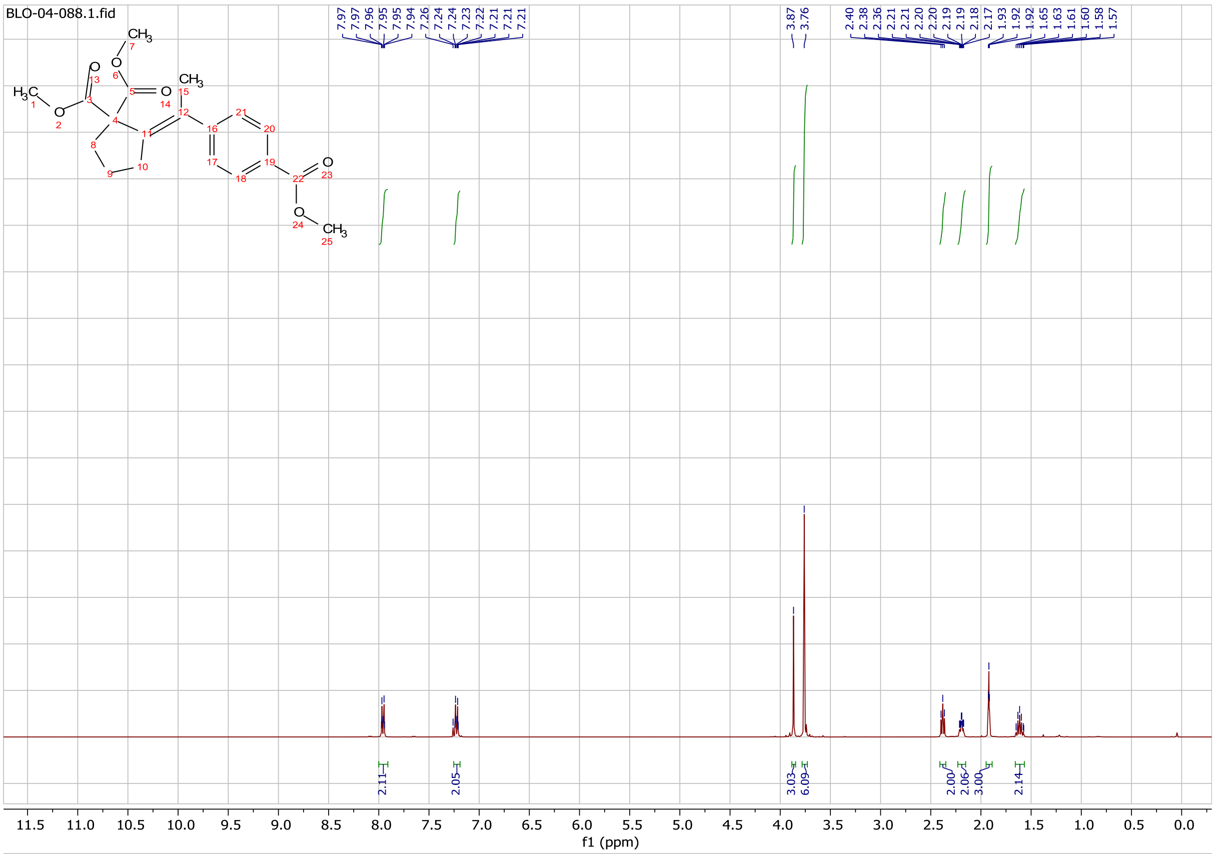

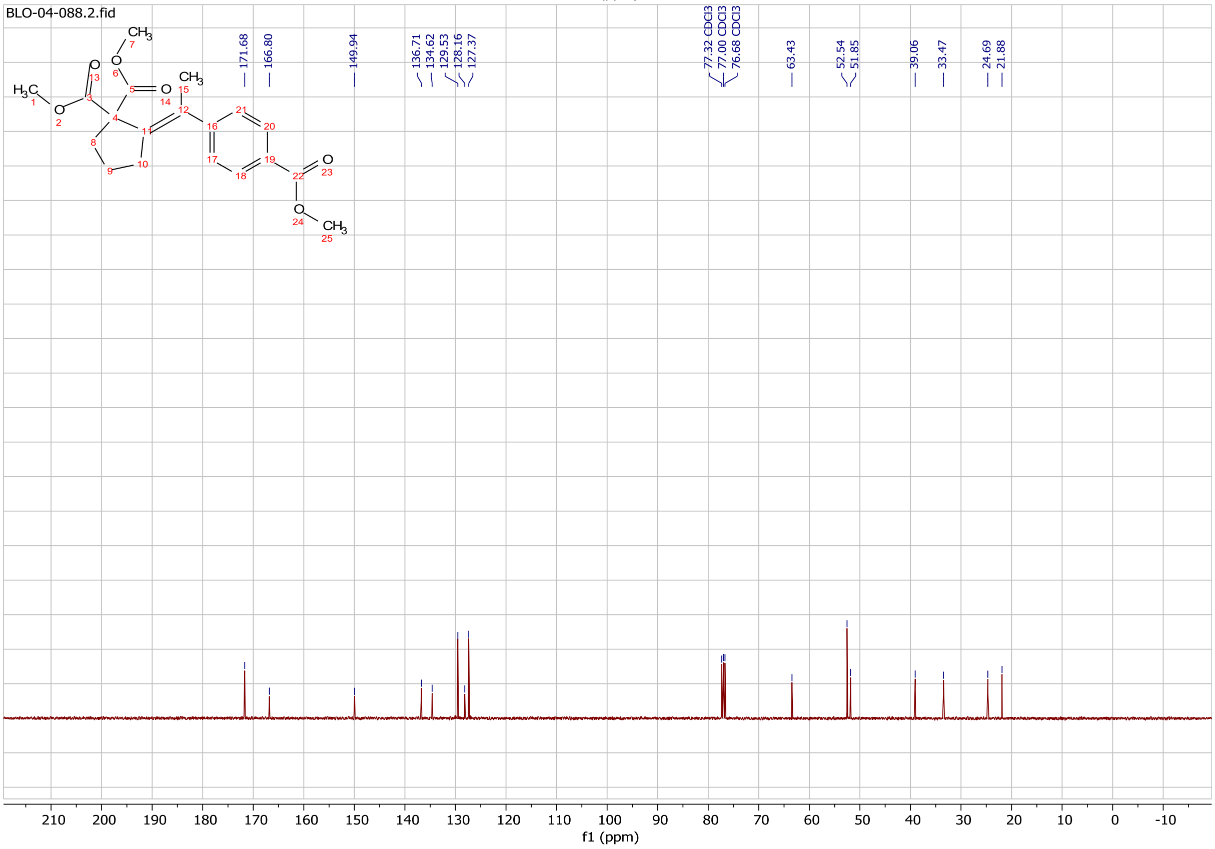

Dimethyl *(E)*-2-(1-(2-(methoxycarbonyl)phenyl)ethylidene)cyclopentane-1,1-dicarboxylate (10)

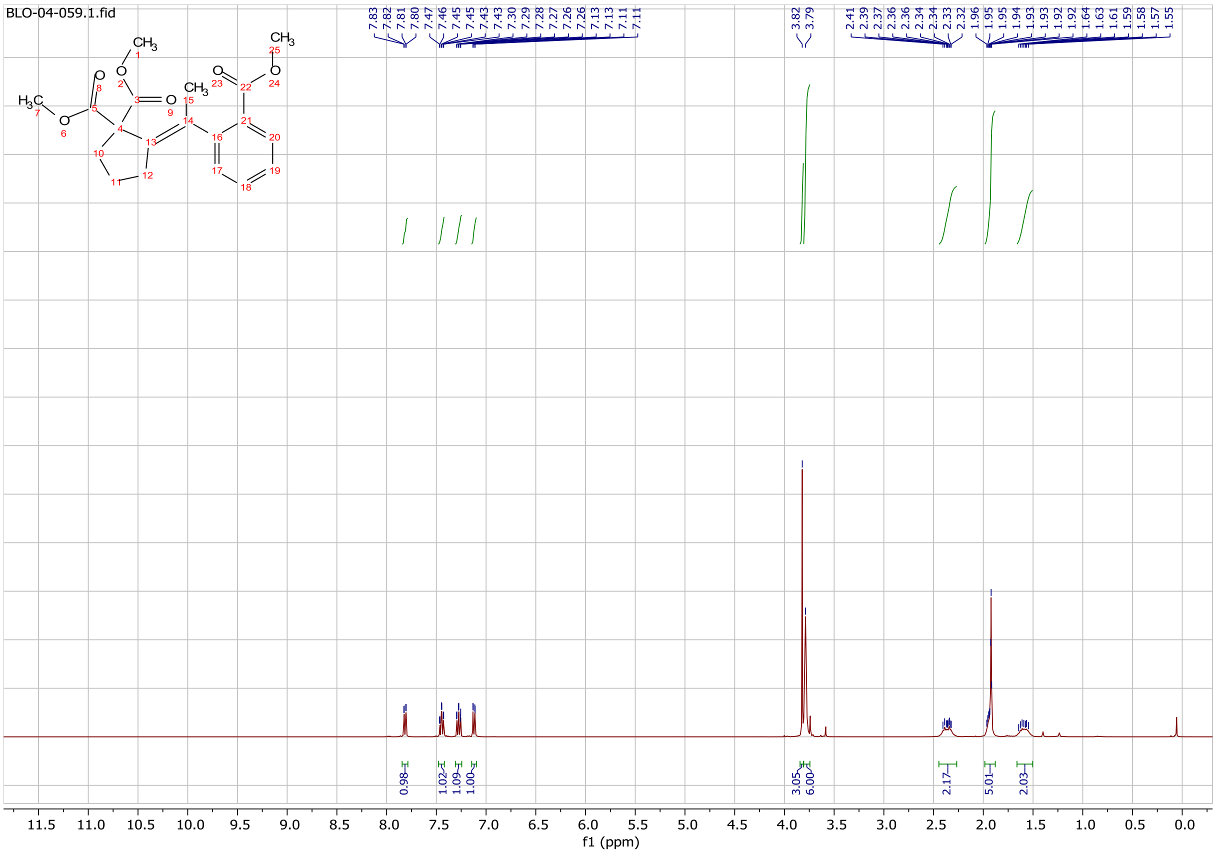

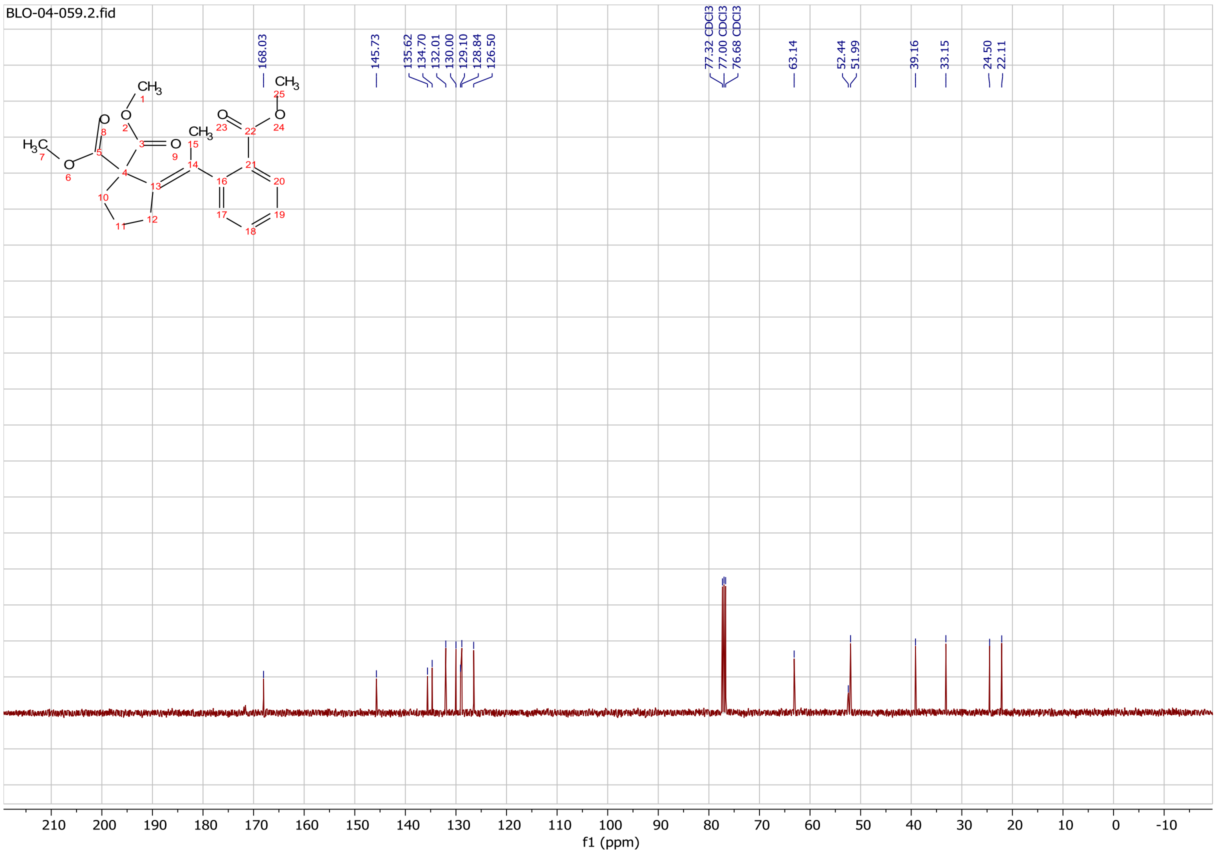

Dimethyl (E)-2-(1-(naphthalen-1-yl)ethylidene)cyclopentane-1,1-dicarboxylate (11)

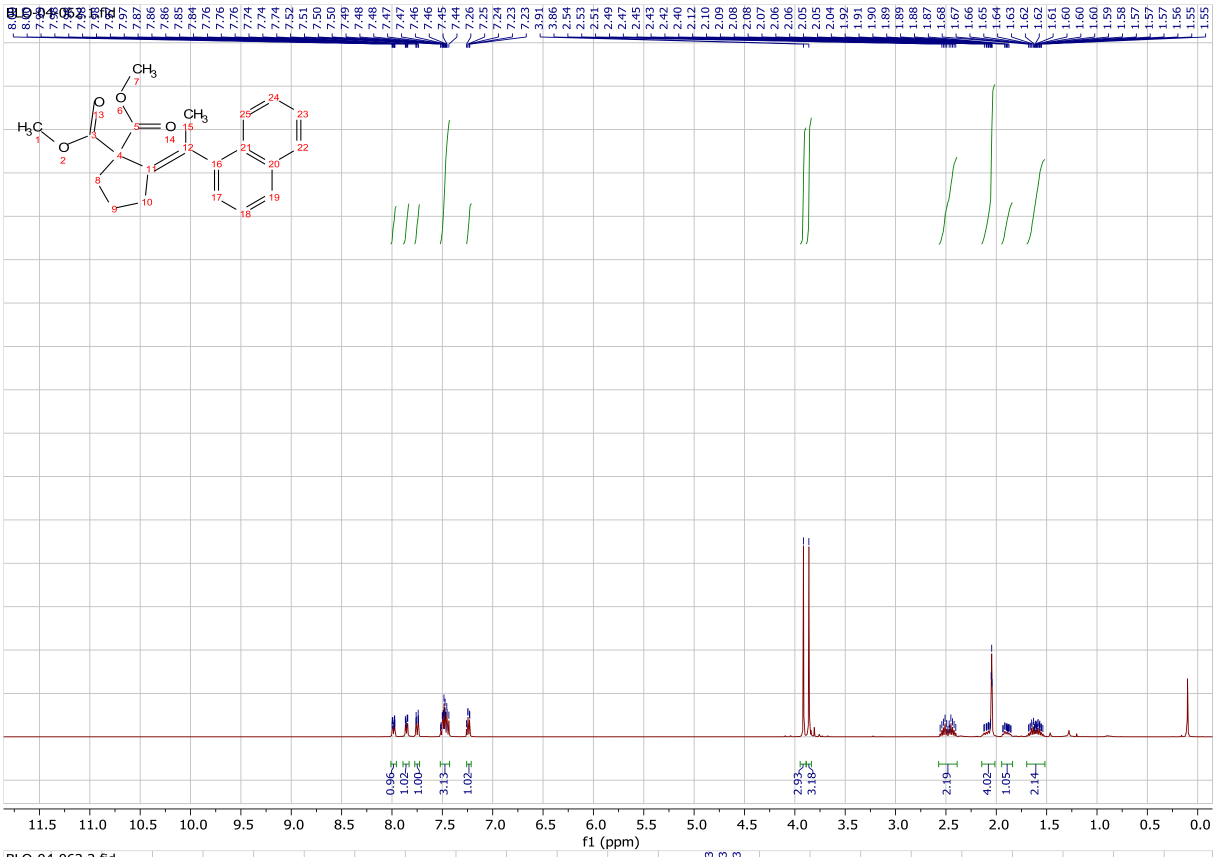

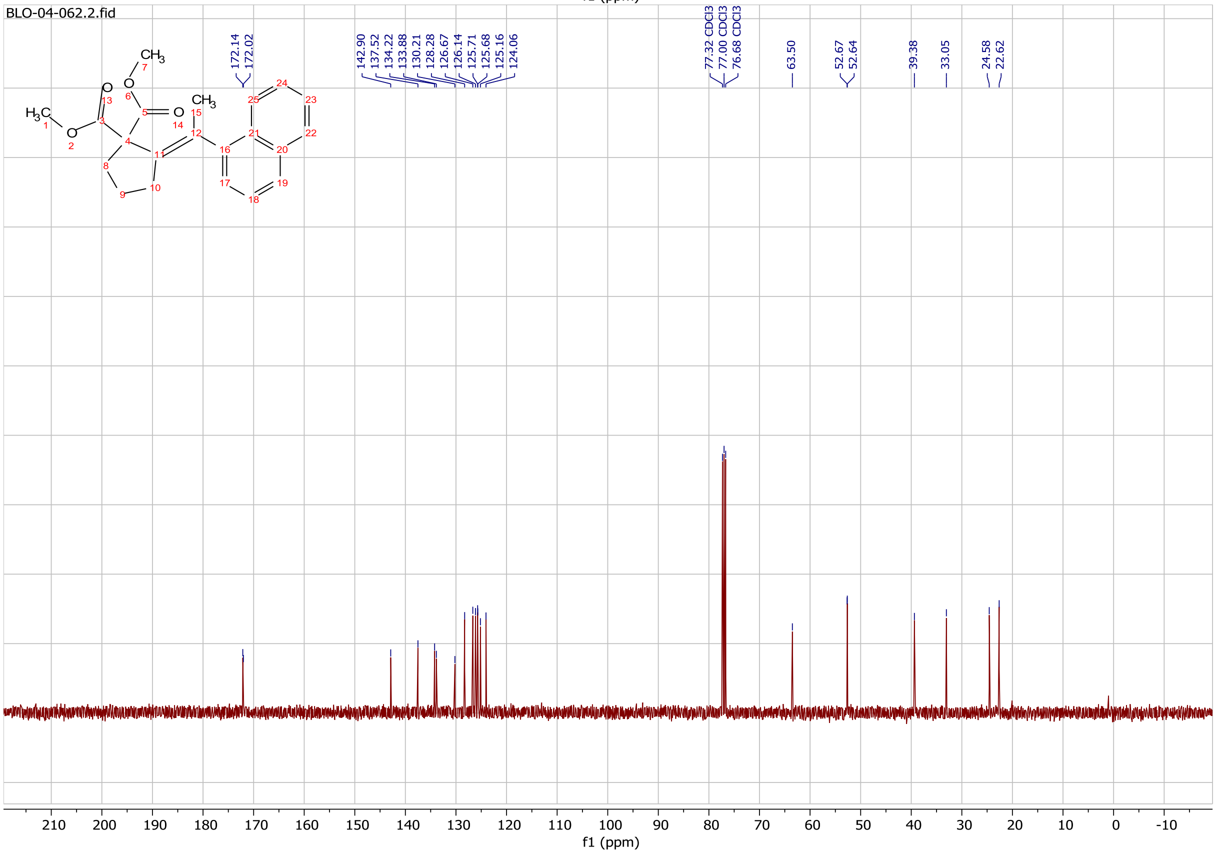

Dimethyl *(E)*-2-(1-(4-chlorophenyl)ethylidene)cyclopentane-1,1-dicarboxylate (12)

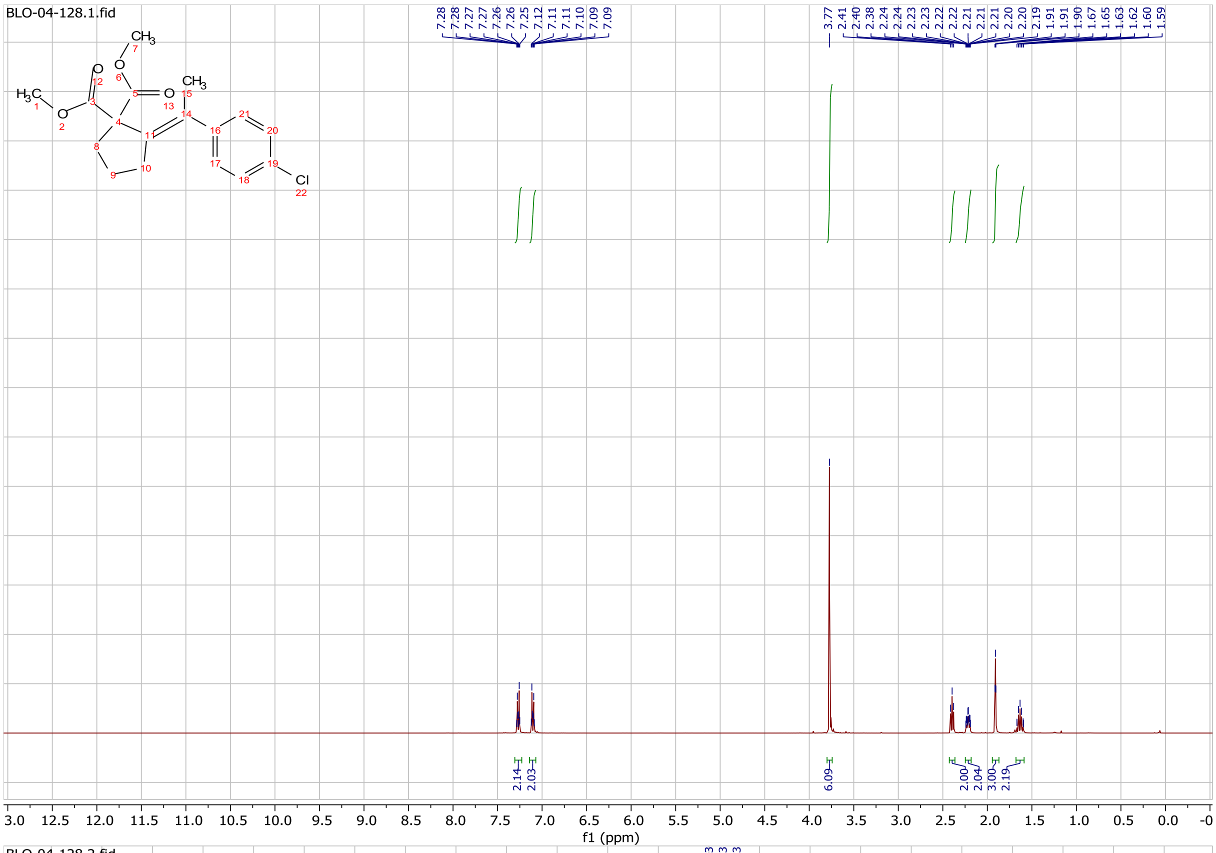

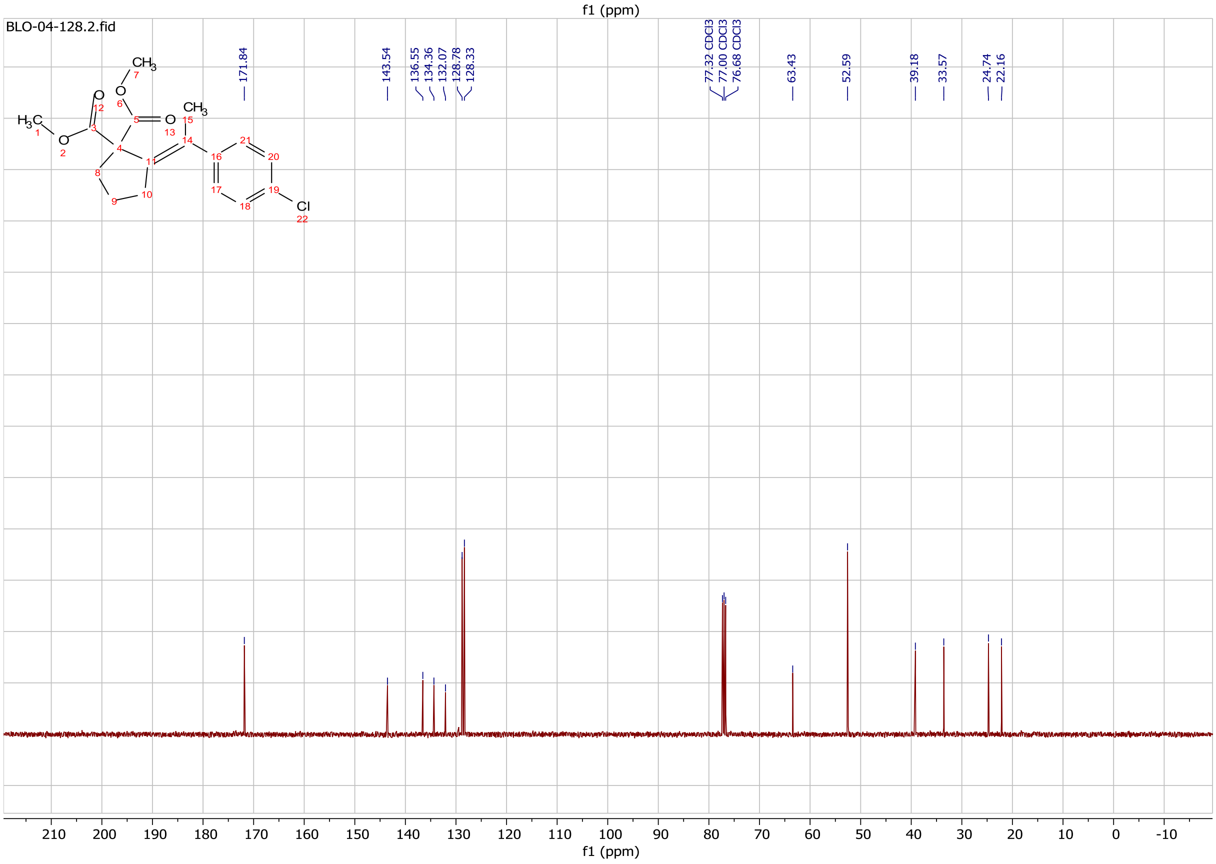

Dimethyl *(E)*-2-(1-(4-acetamidophenyl)ethylidene)cyclopentane-1,1-dicarboxylate (13)

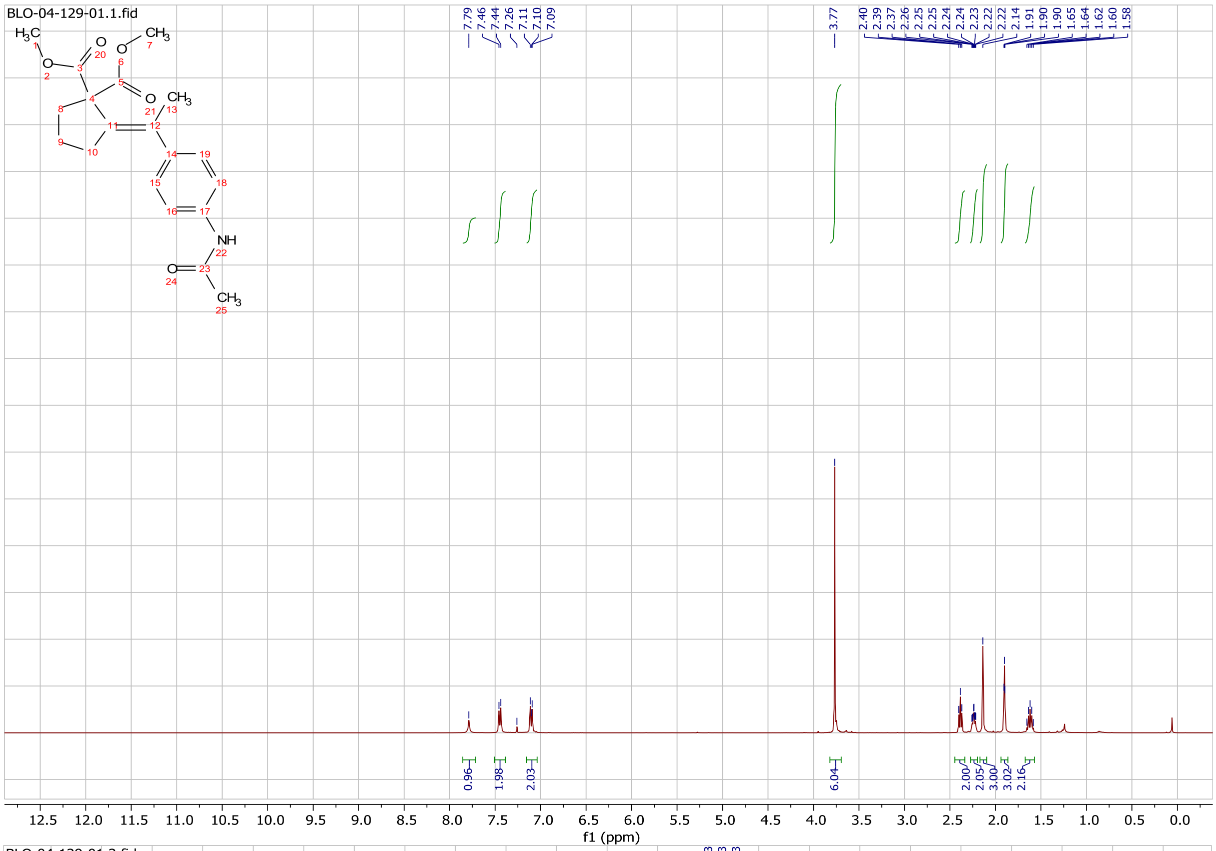

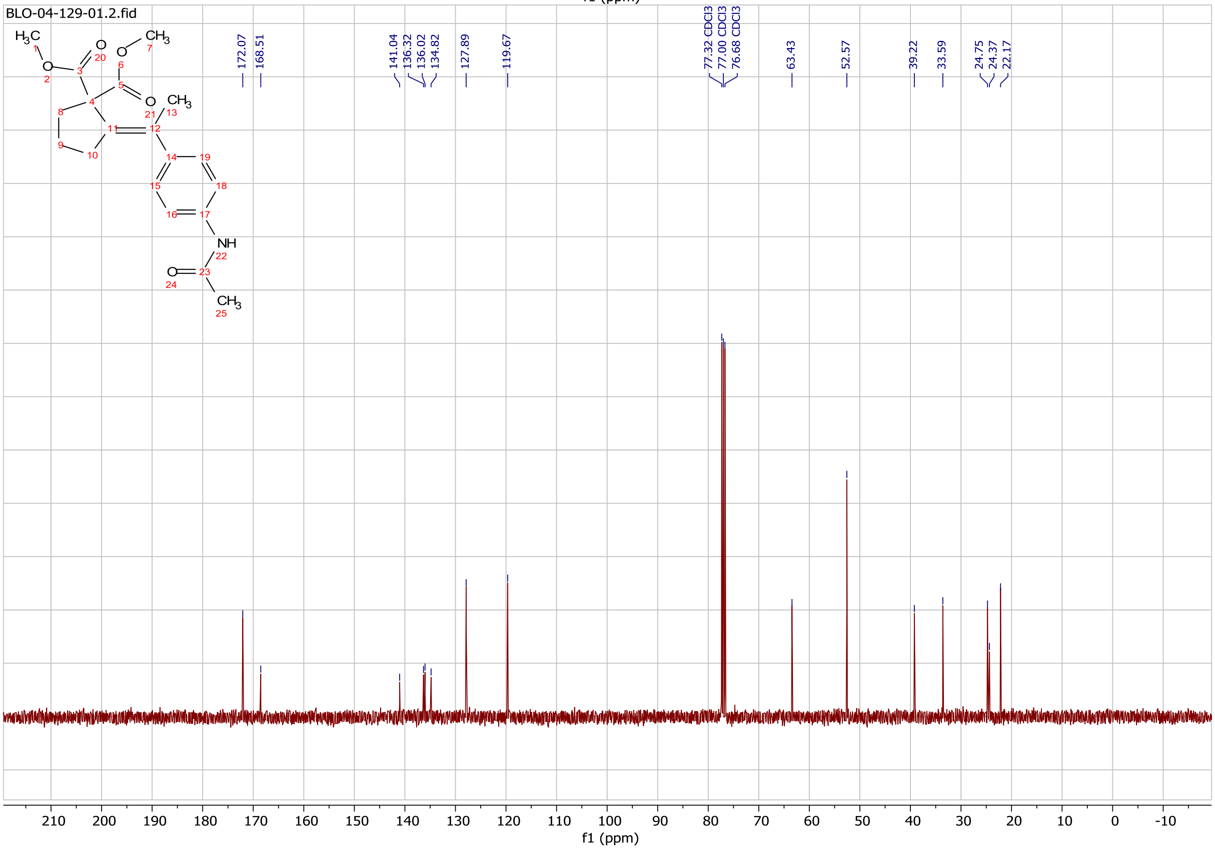

Dimethyl *(E)*-2-(1-(pyridin-3-yl)ethylidene)cyclopentane-1,1-dicarboxylate (14)

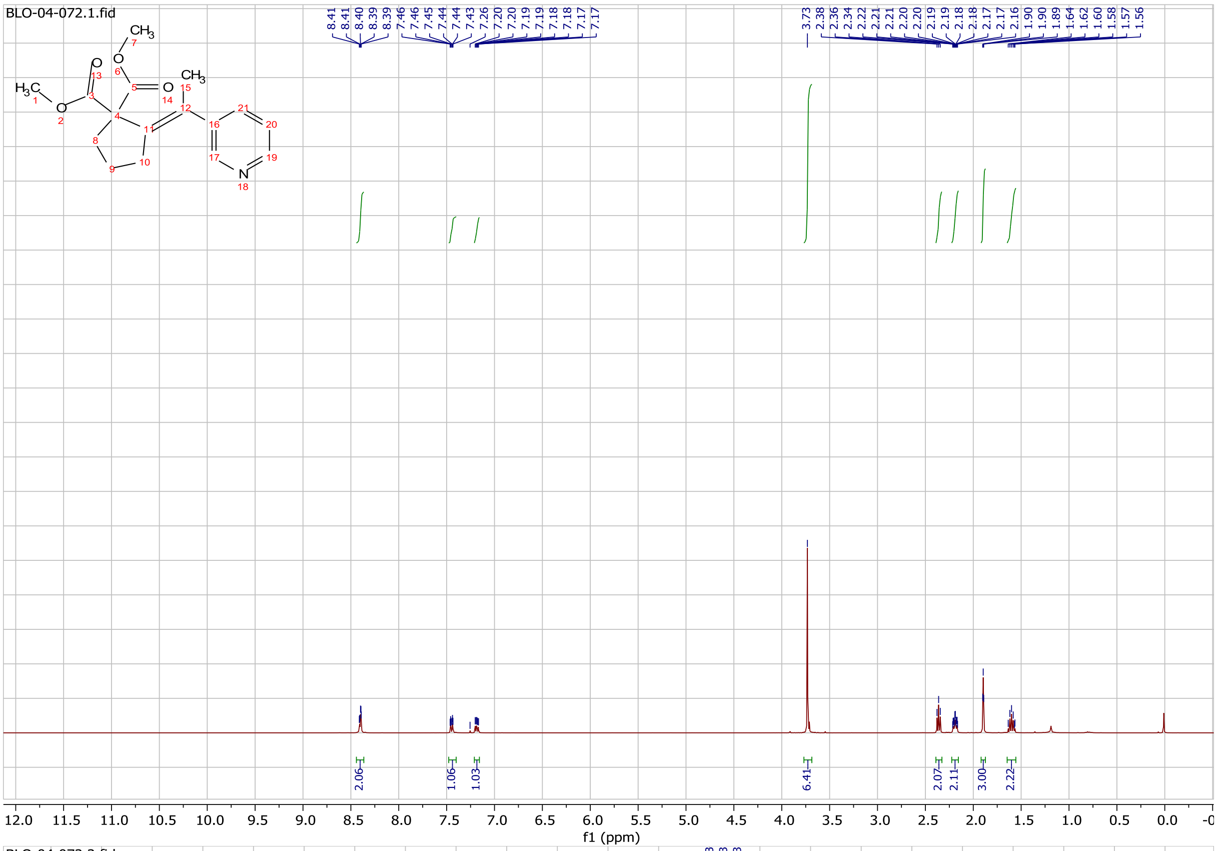

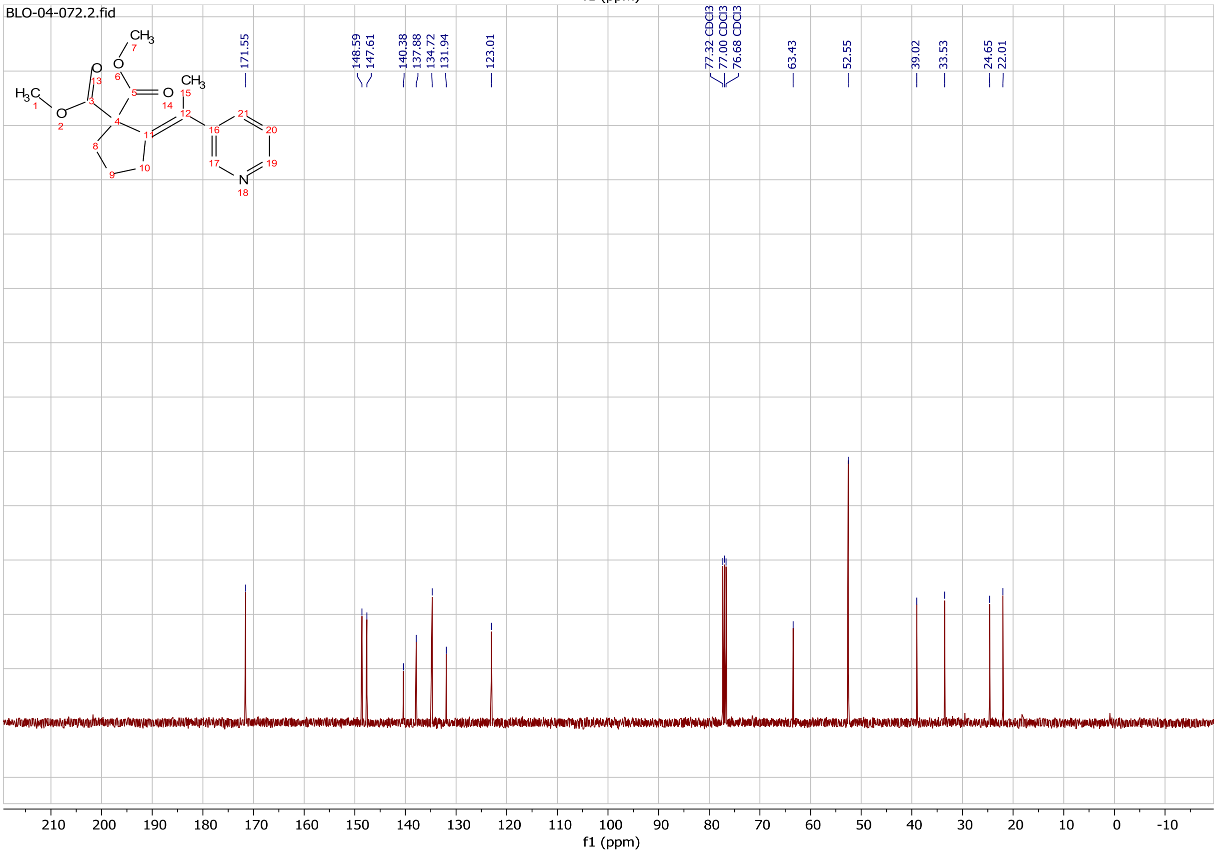

Dimethyl *(E)*-2-(1-(pyrimidin-5-yl)ethylidene)cyclopentane-1,1-dicarboxylate (15)

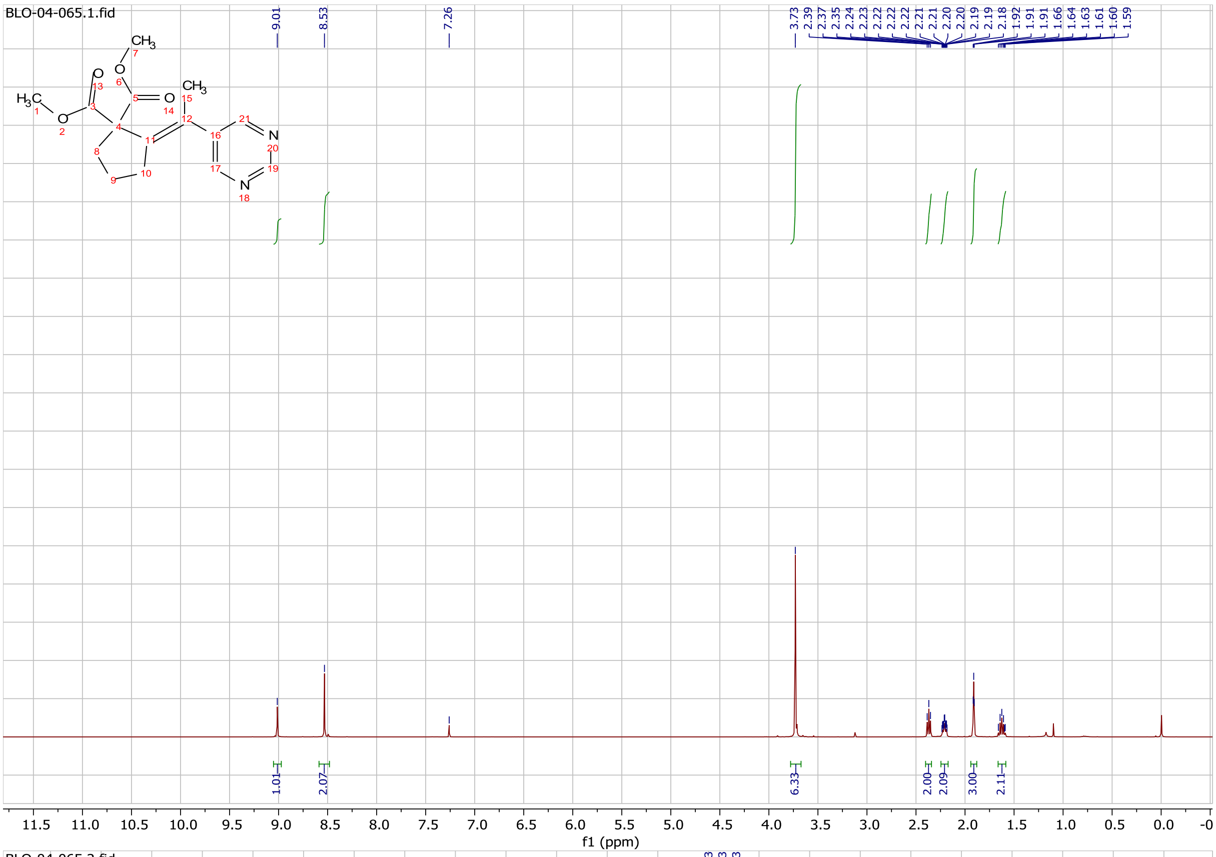

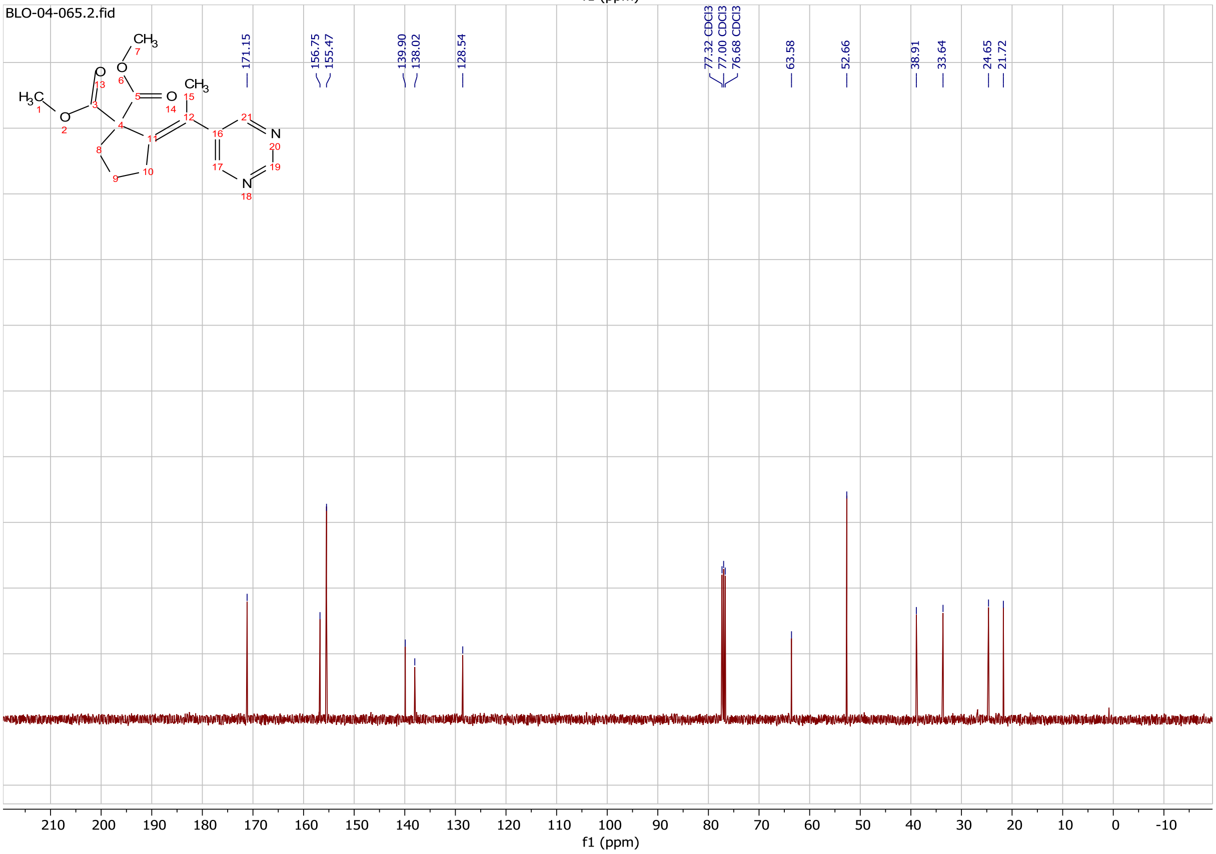

Dimethyl *(E)*-2-(1-(pyrazin-2-yl)ethylidene)cyclopentane-1,1-dicarboxylate (16)

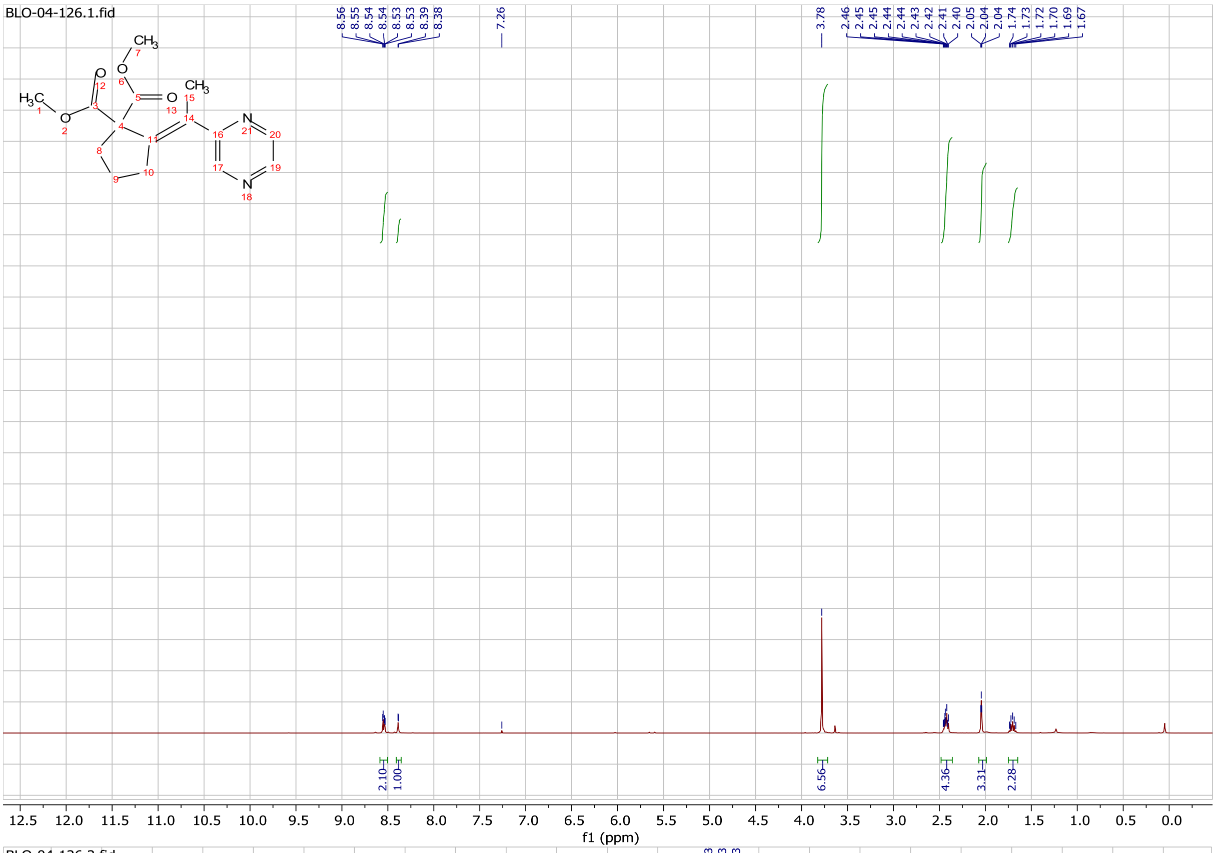

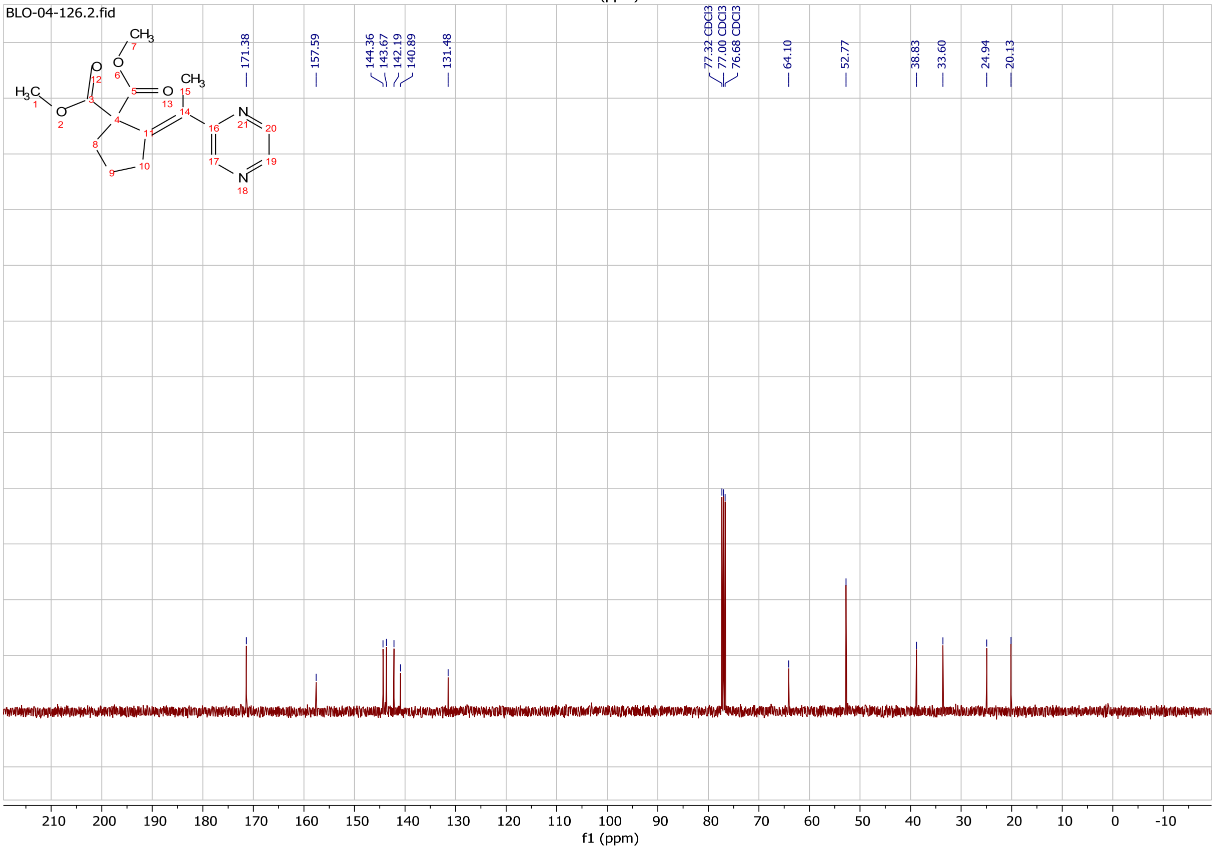

Dimethyl *(E)*-2-(1-(quinoxalin-6-yl)ethylidene)cyclopentane-1,1-dicarboxylate (17)

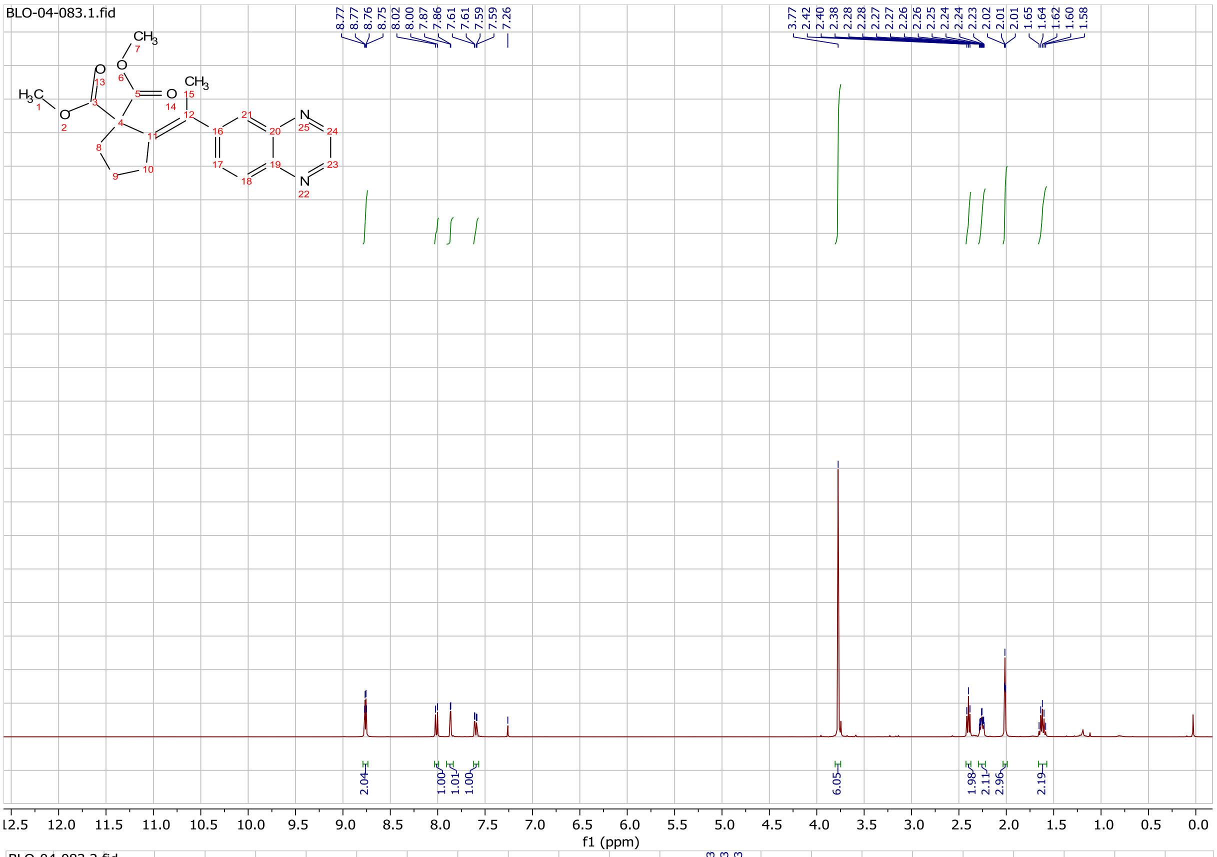

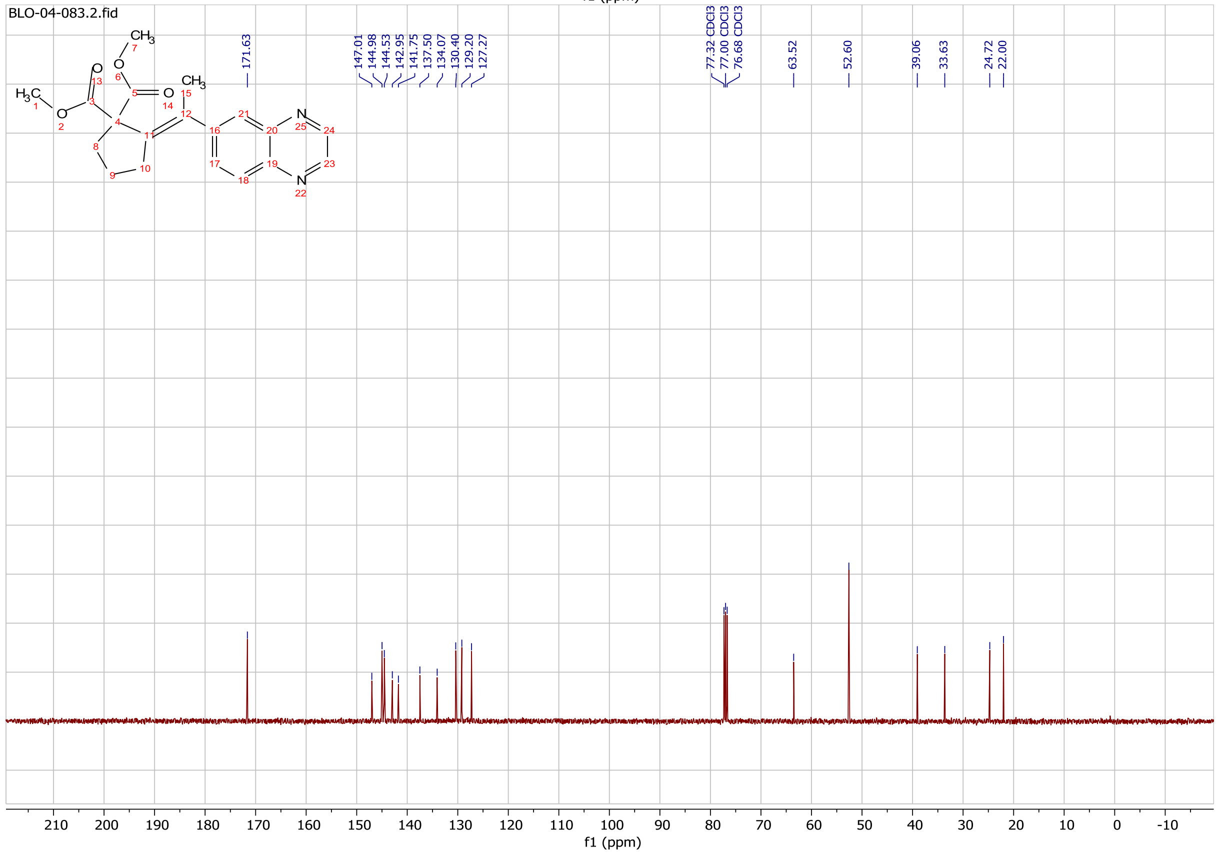

Dimethyl *(E)*-2-(1-(benzo[d]thiazol-5-yl)ethylidene)cyclopentane-1,1-dicarboxylate (18)

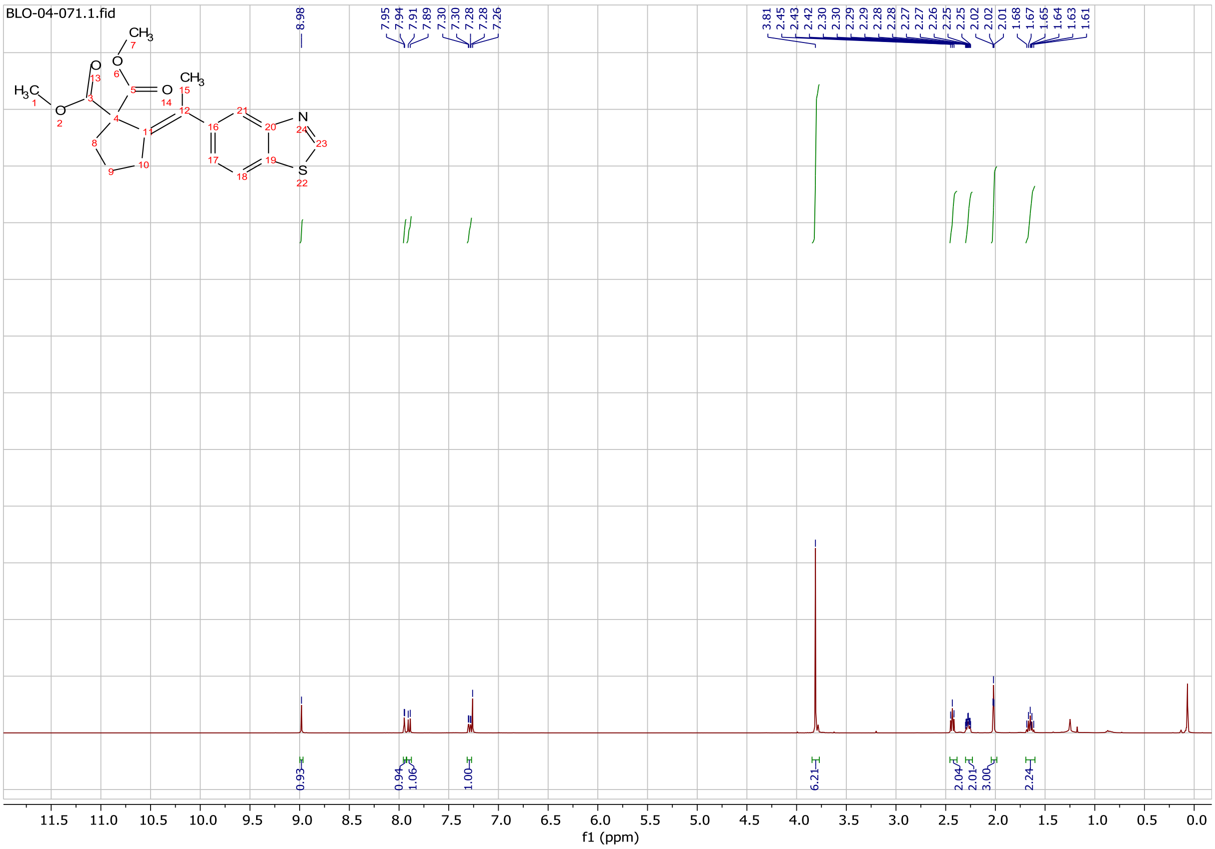

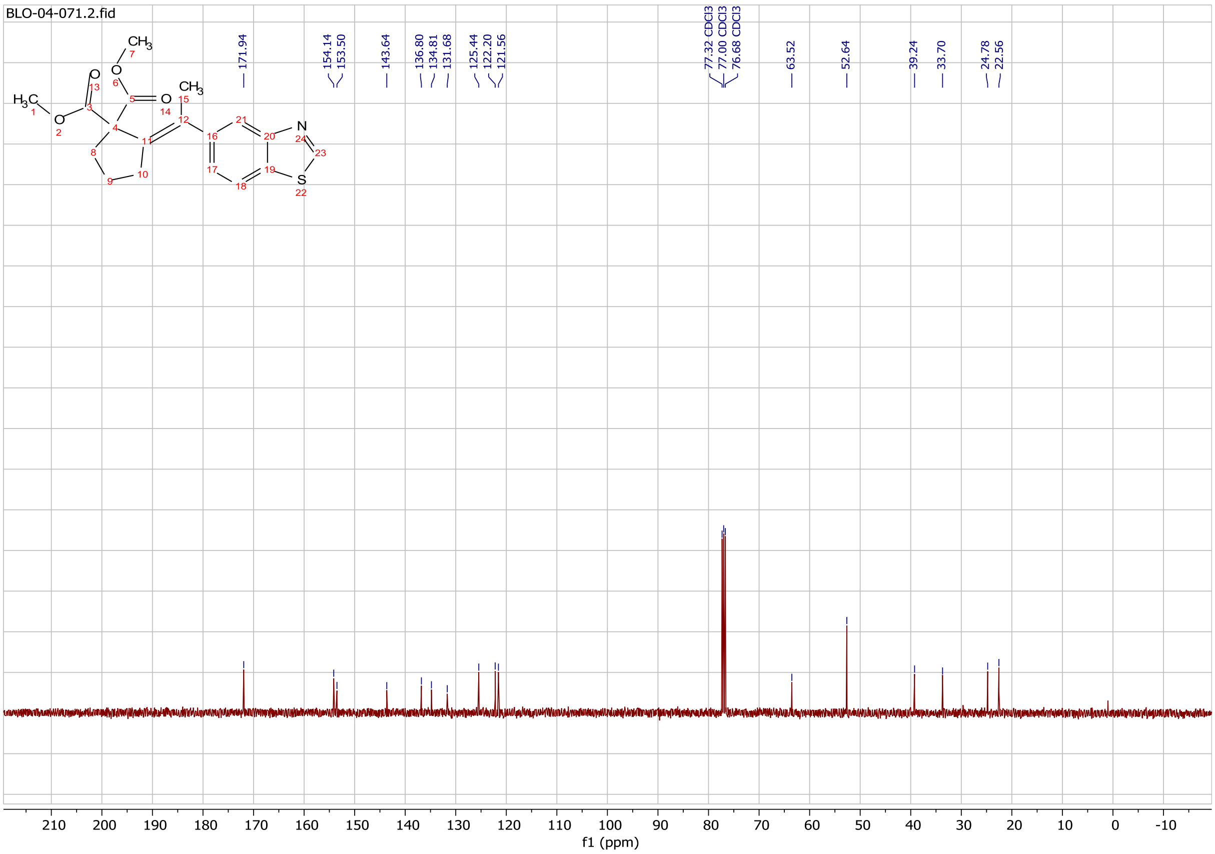

Dimethyl *(E)*-2-(1-(benzo[b]thiophen-5-yl)ethylidene)cyclopentane-1,1-dicarboxylate (19)

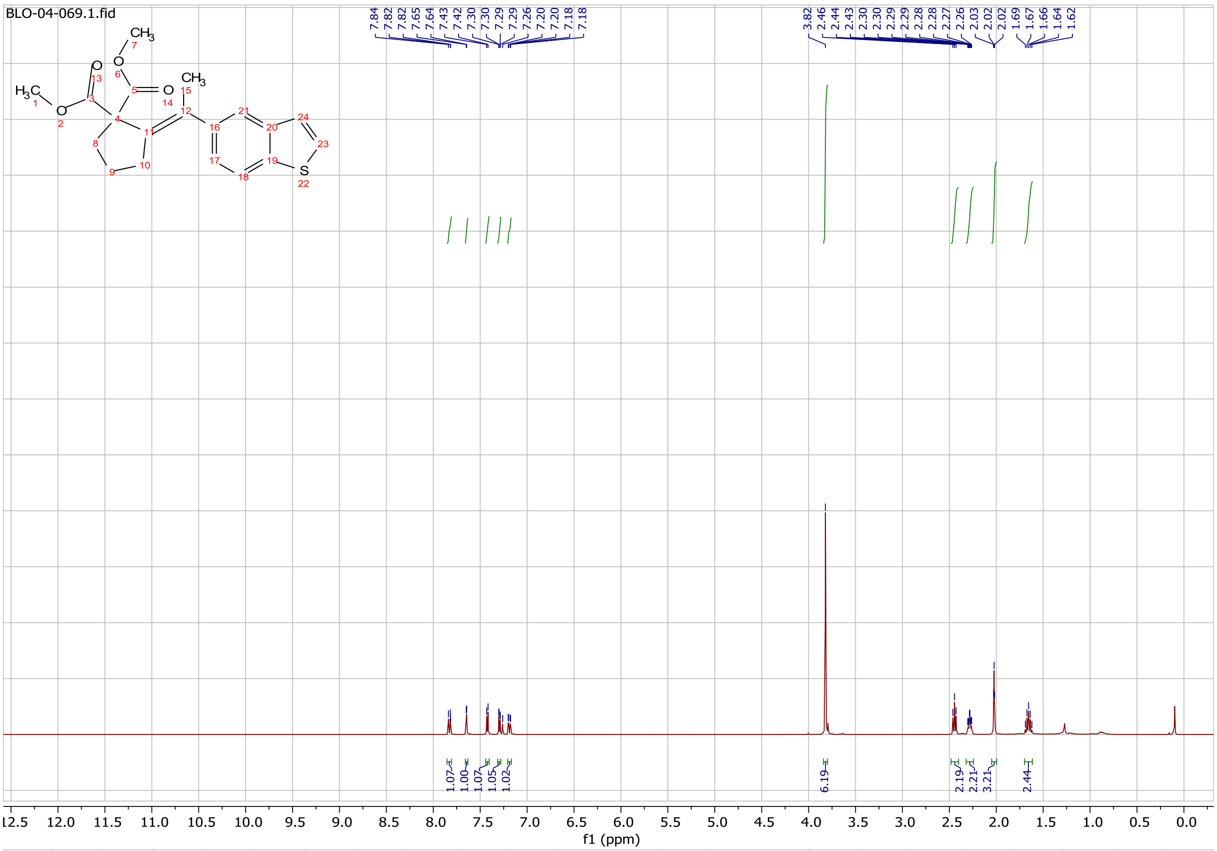

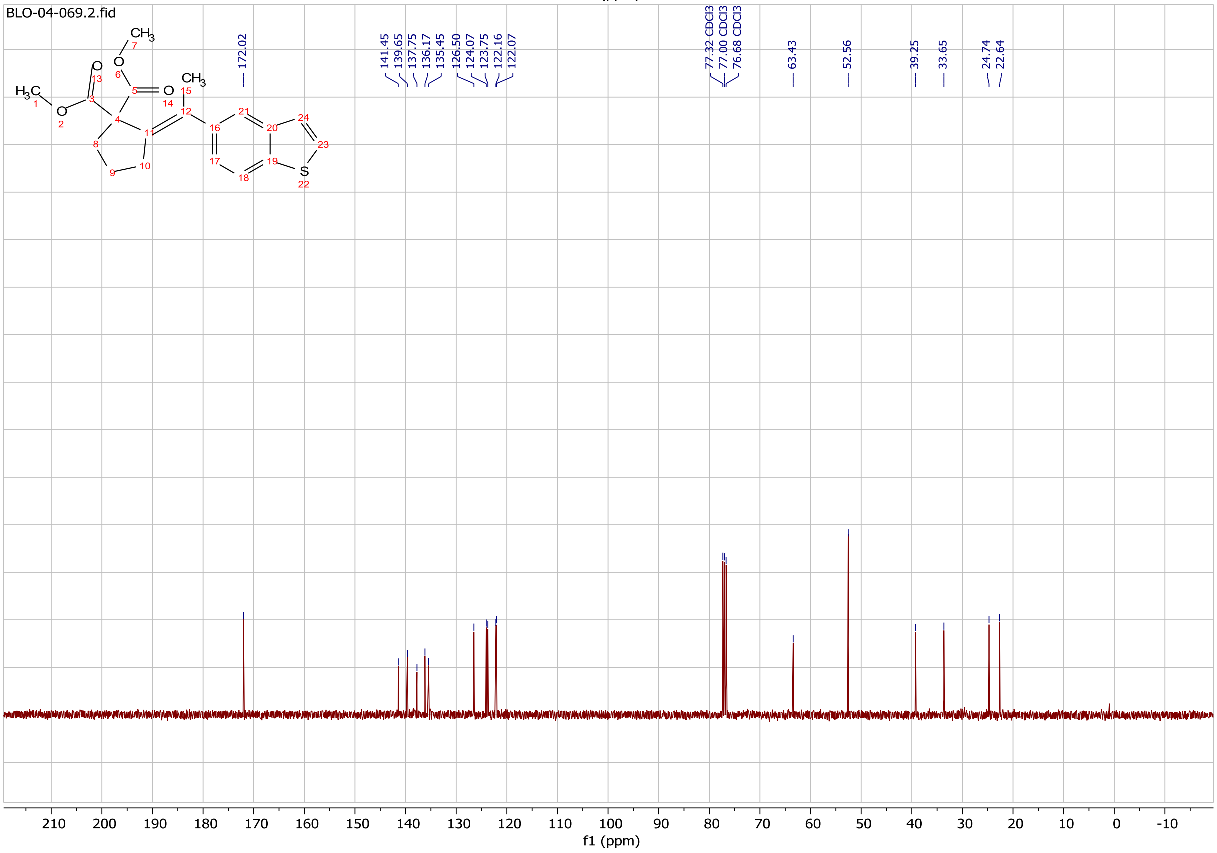

Dimethyl *(E)*-2-(1-(2-methylbenzo[d]oxazol-5-yl)ethylidene) cyclopentane-1,1-dicarboxylate (20)

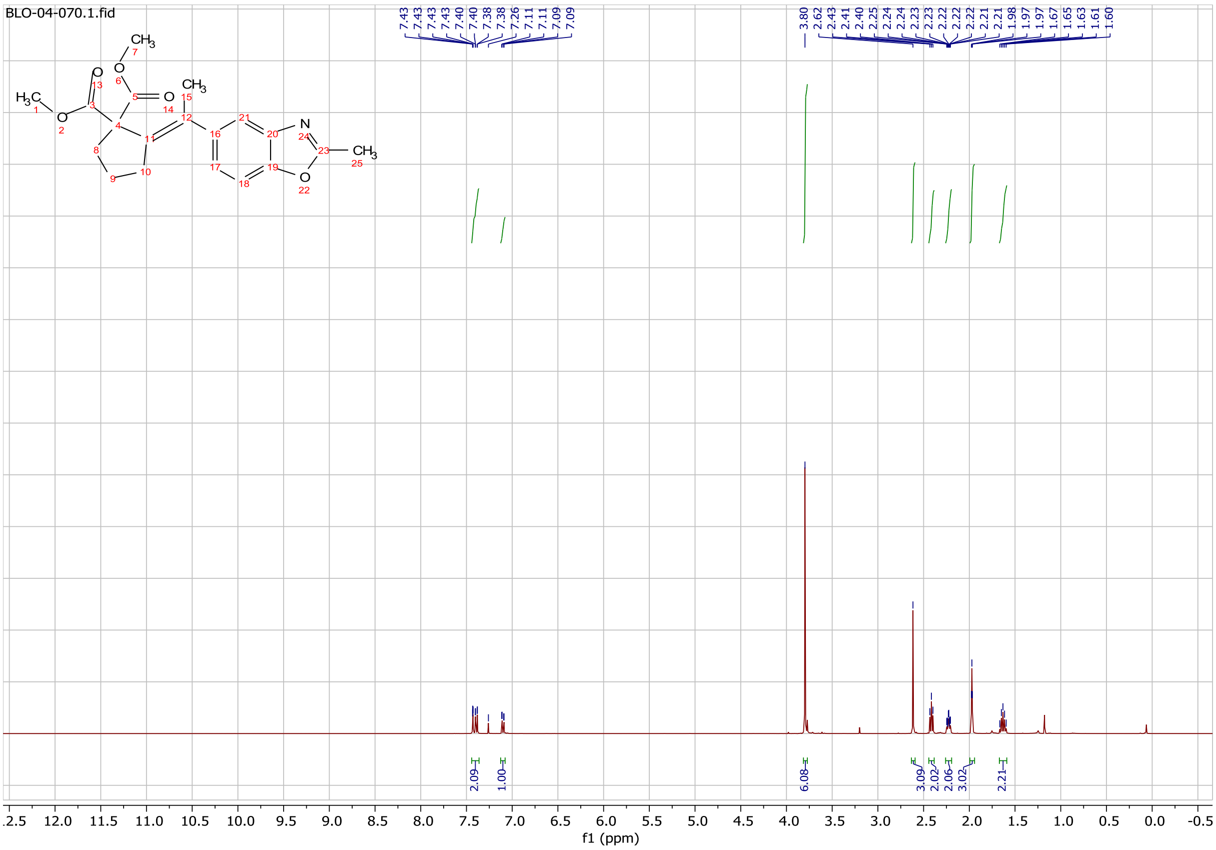

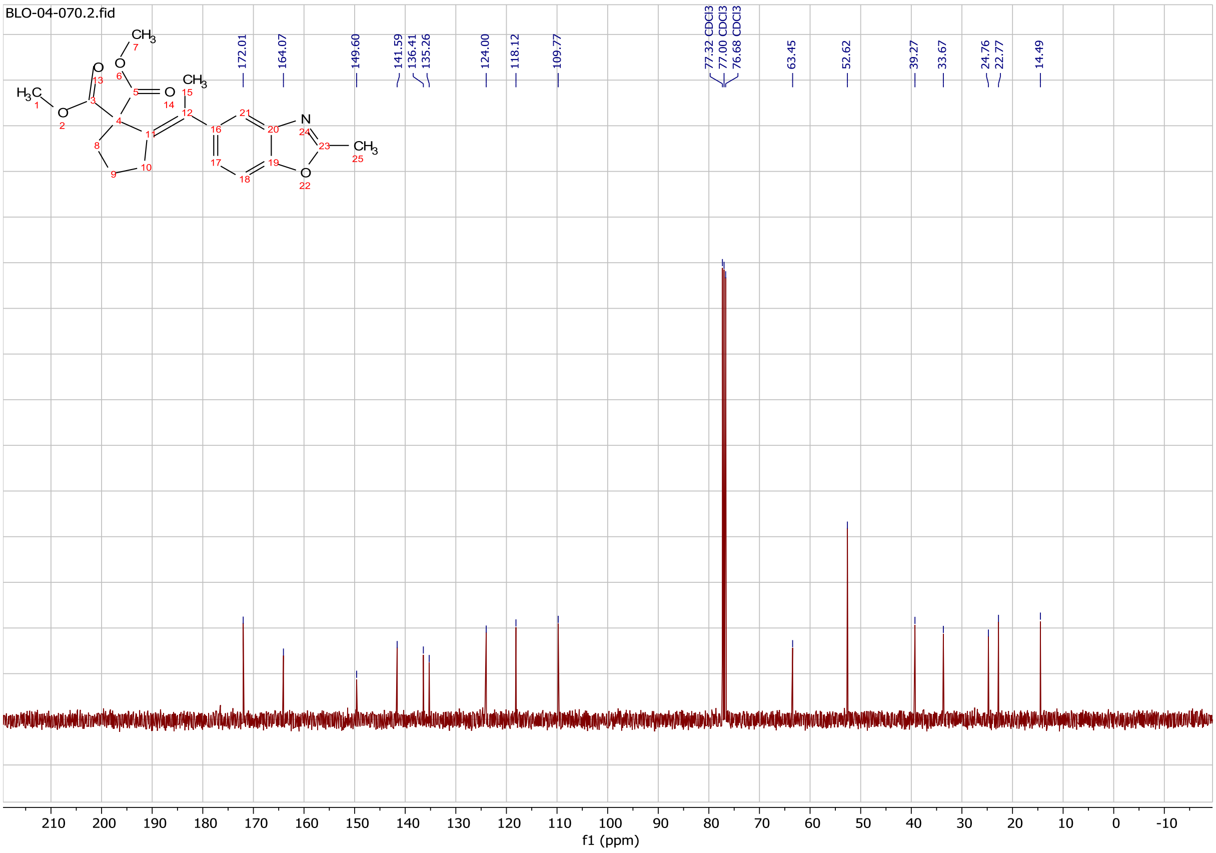

Dimethyl *(E)*-2-(1-(benzo[d][1,3]dioxol-5-yl)ethylidene)cyclopentane-1,1-dicarboxylate (21)

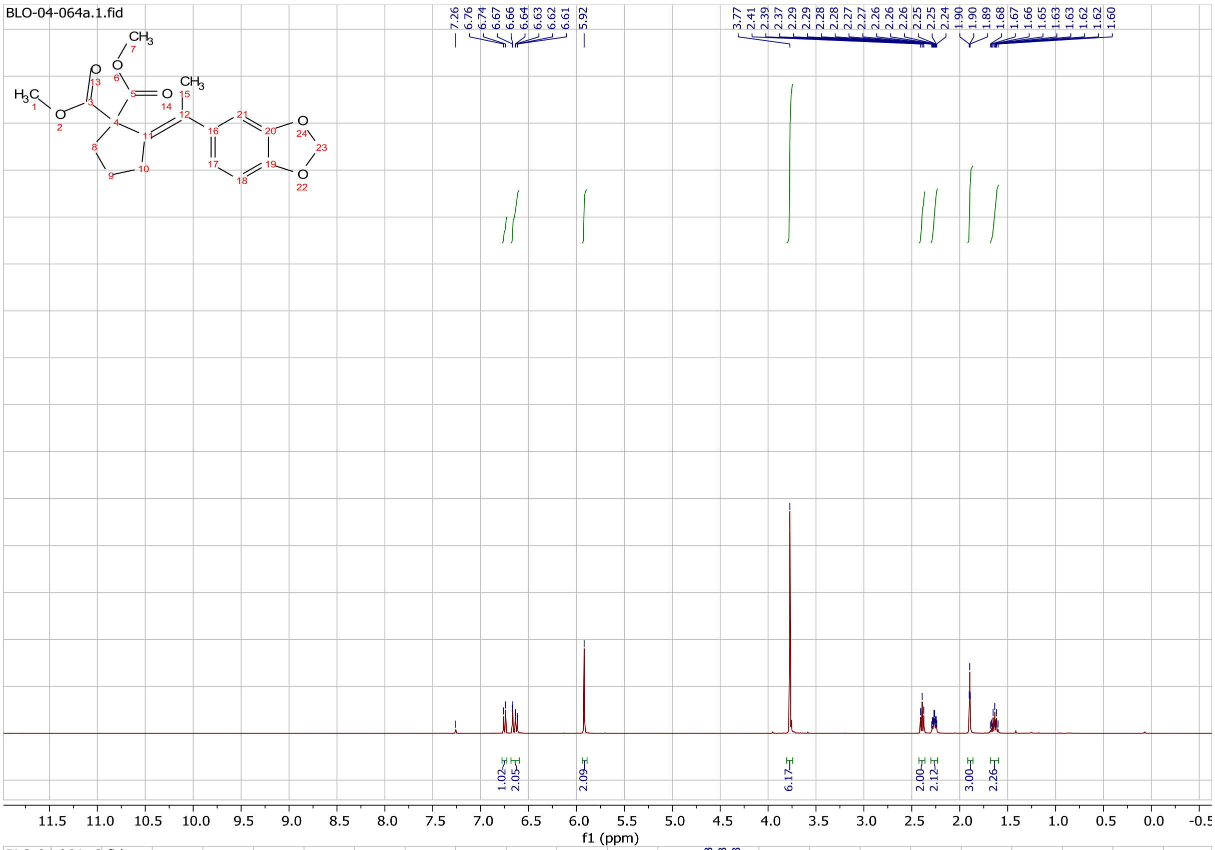

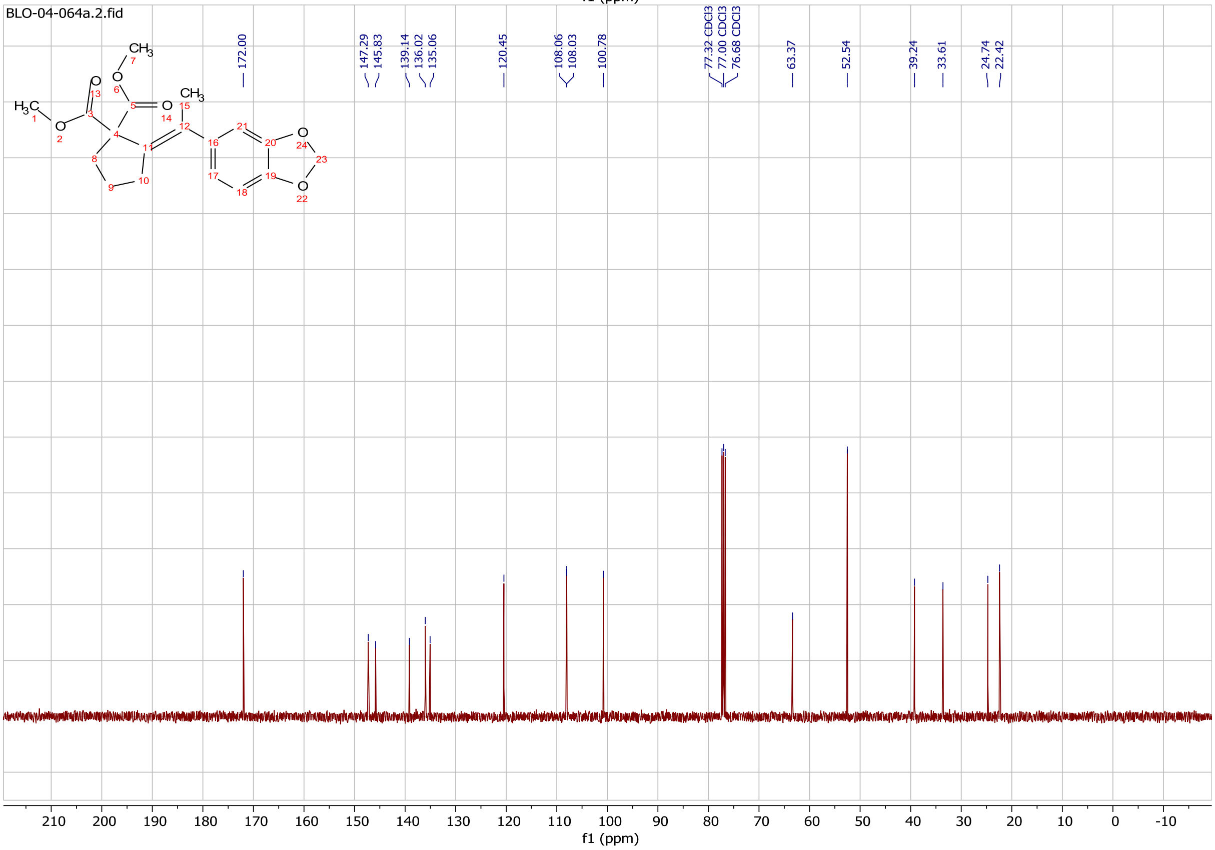

Dimethyl *(E)*-2-(1-(thiophen-2-yl)ethylidene)cyclopentane-1,1-dicarboxylate (22)

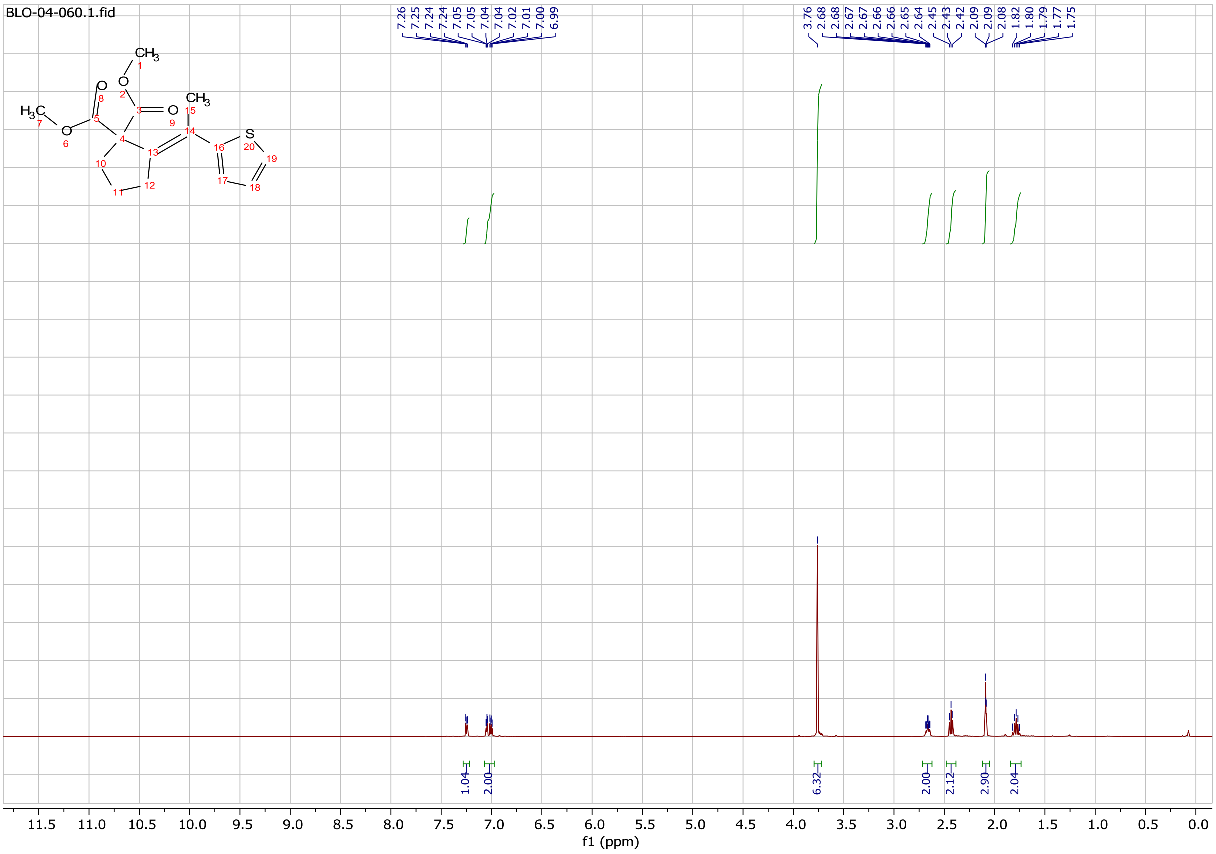

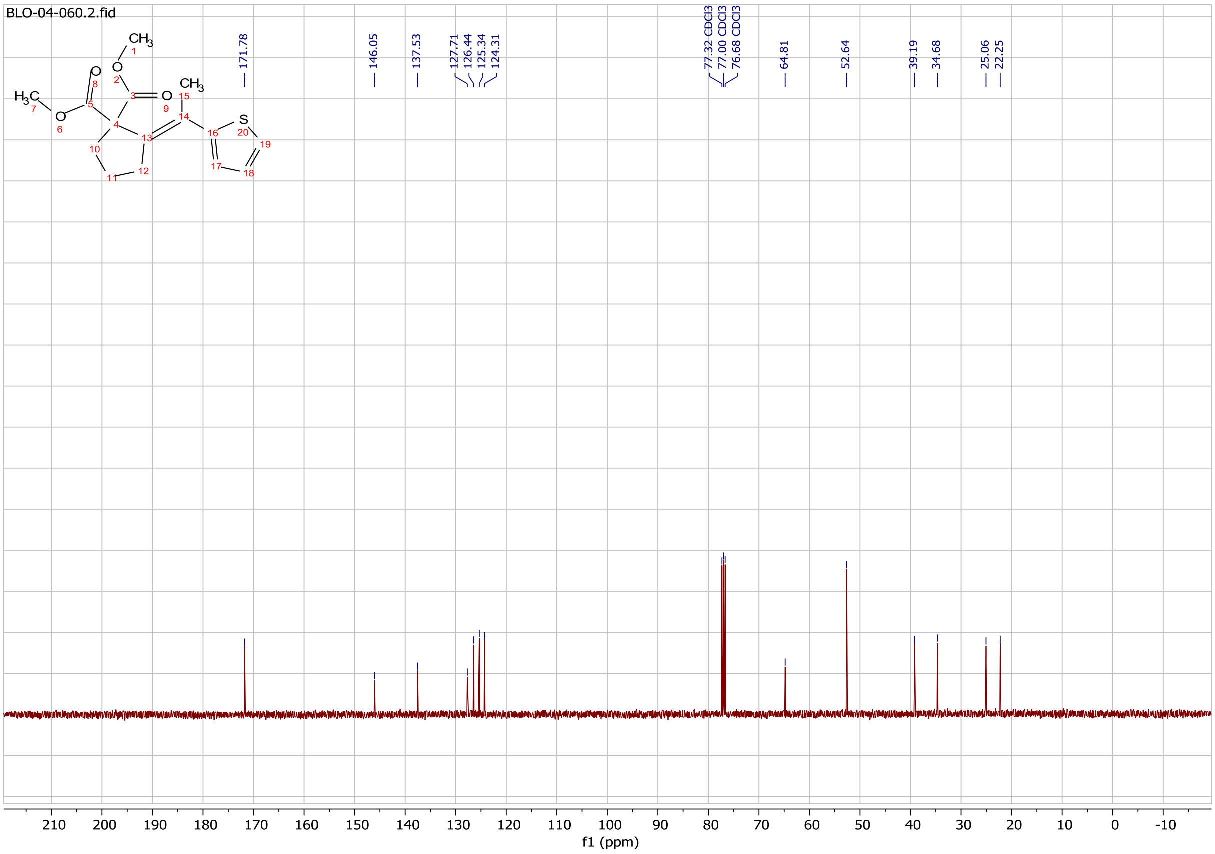

Dimethyl *(E)*-2-(1-(furan-2-yl)ethylidene)cyclopentane-1,1-dicarboxylate (23)

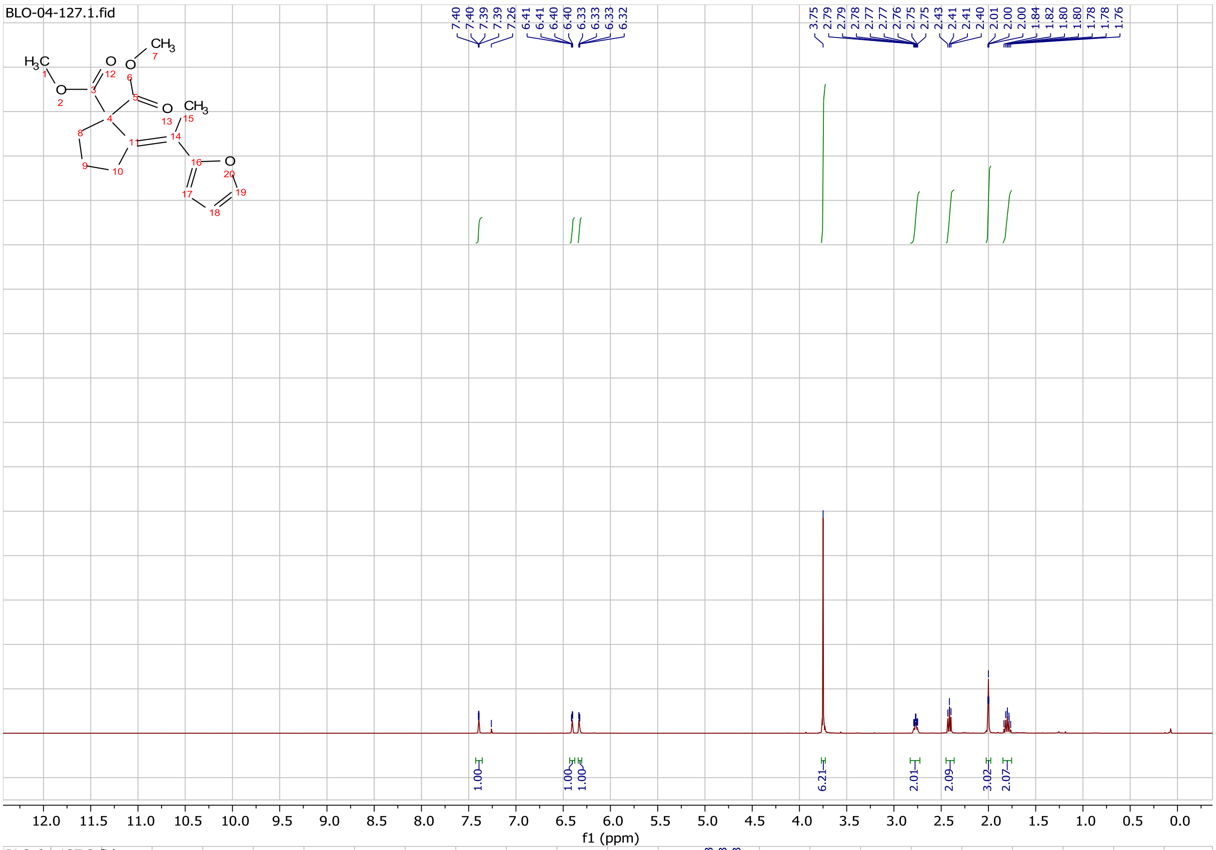

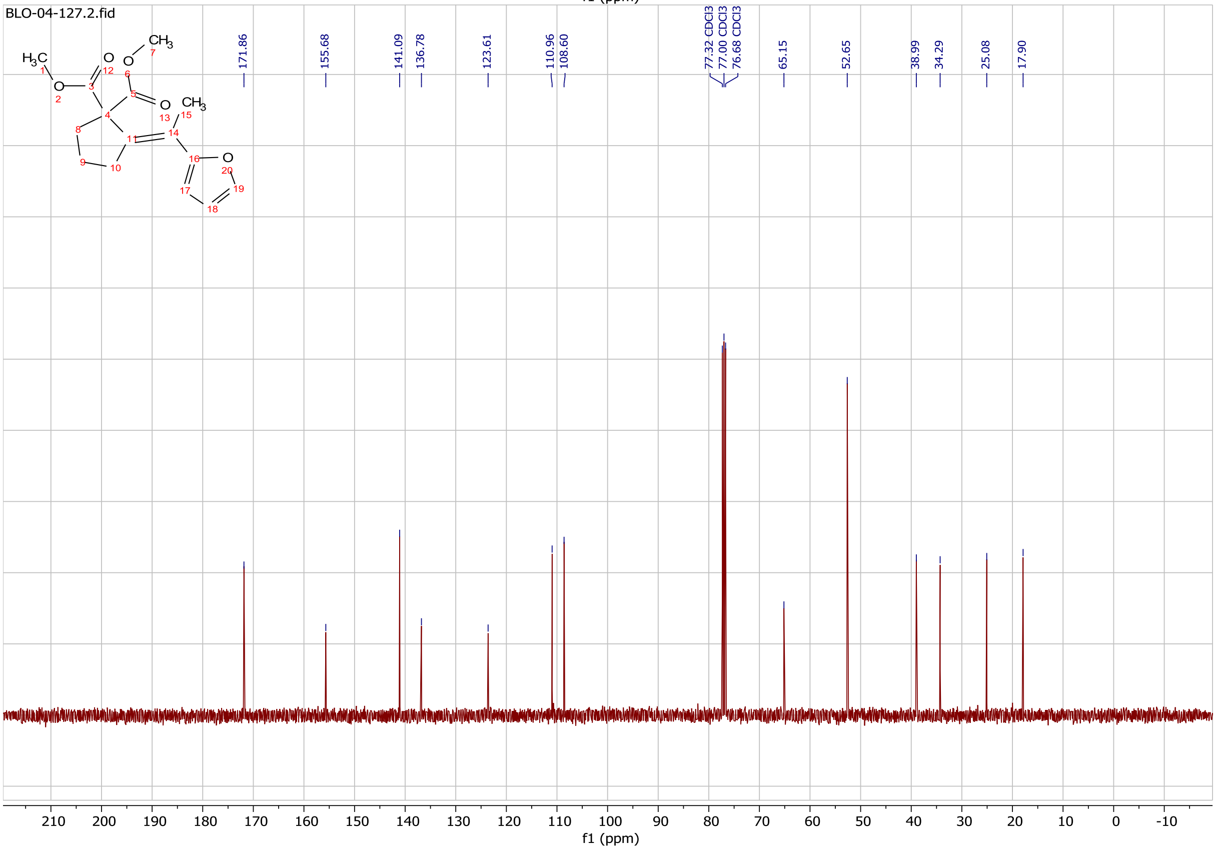

Dimethyl *(E)*-2-(1-(furan-3-yl)ethylidene)cyclopentane-1,1-dicarboxylate (24)

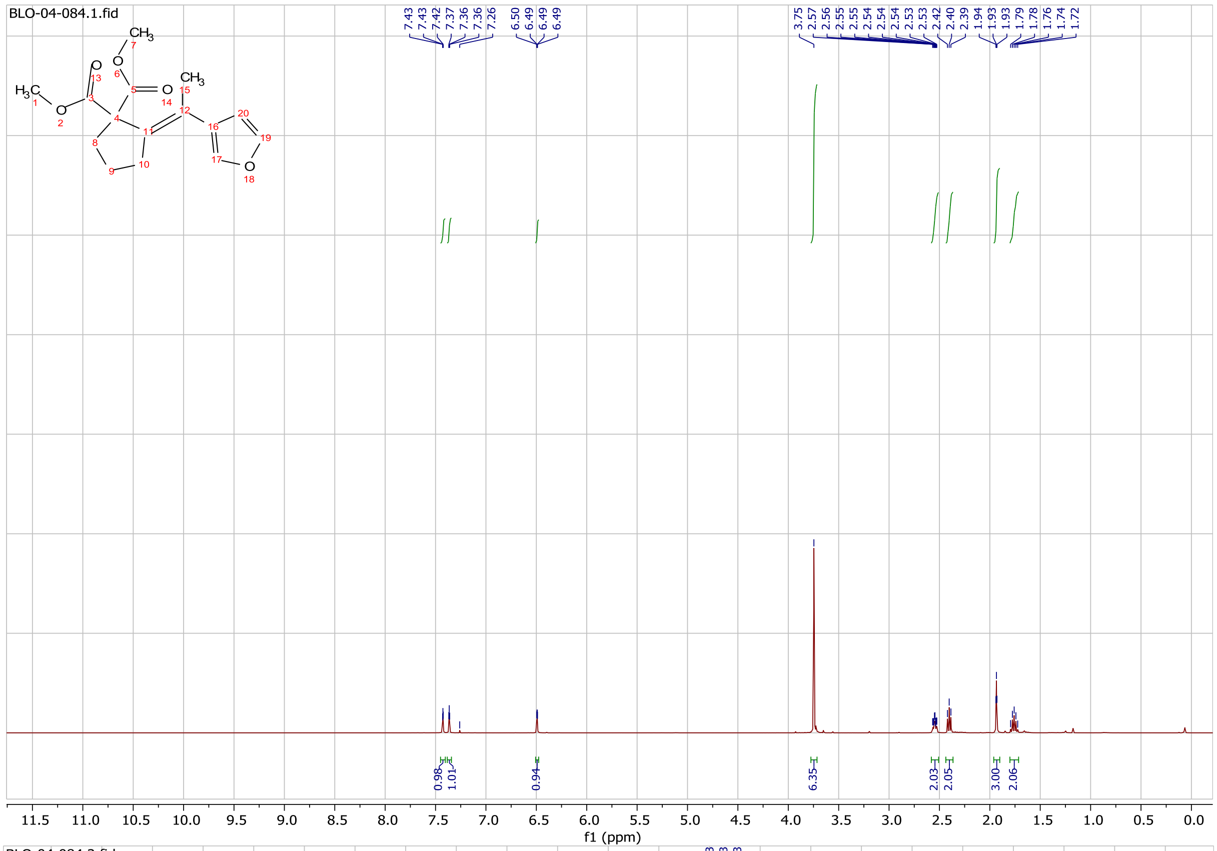

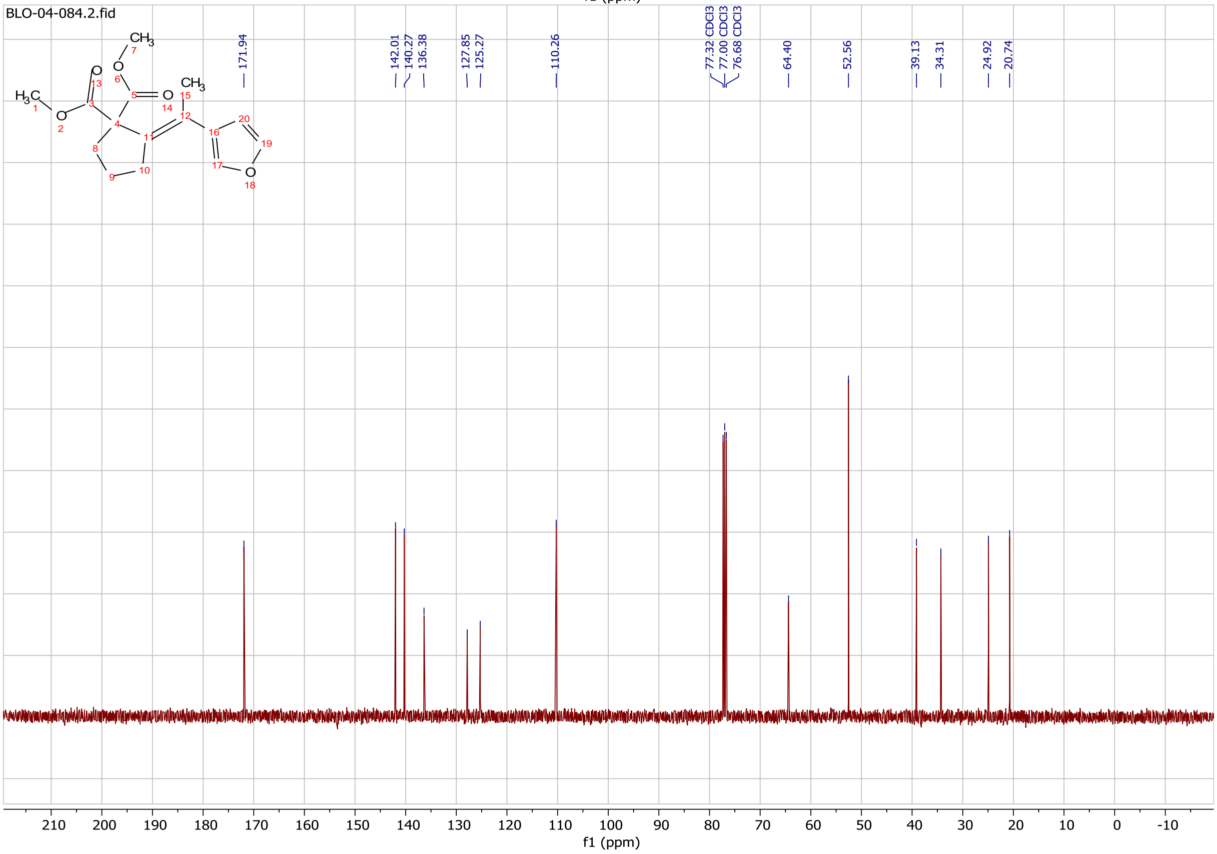

Dimethyl *(E)*-2-(pent-4-en-2-ylidene)cyclopentane-1,1-dicarboxylate (25)

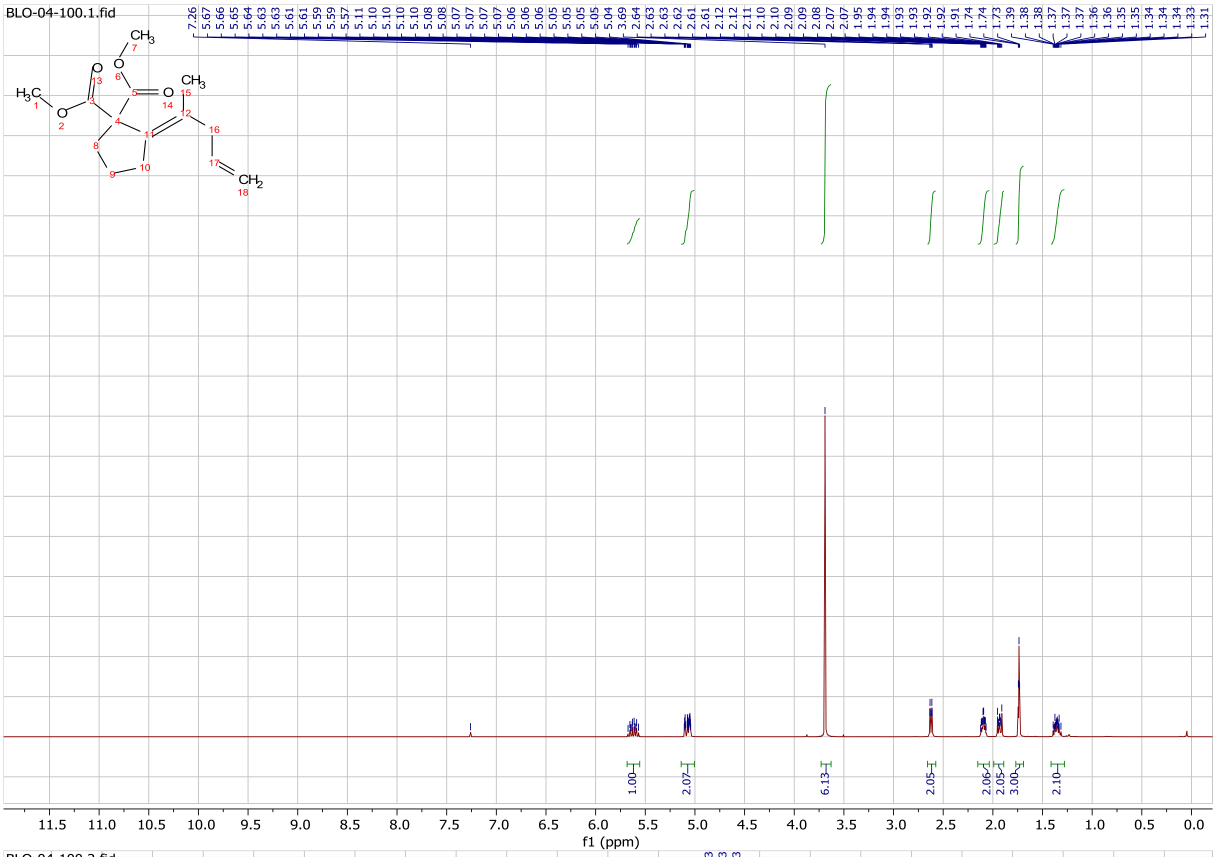

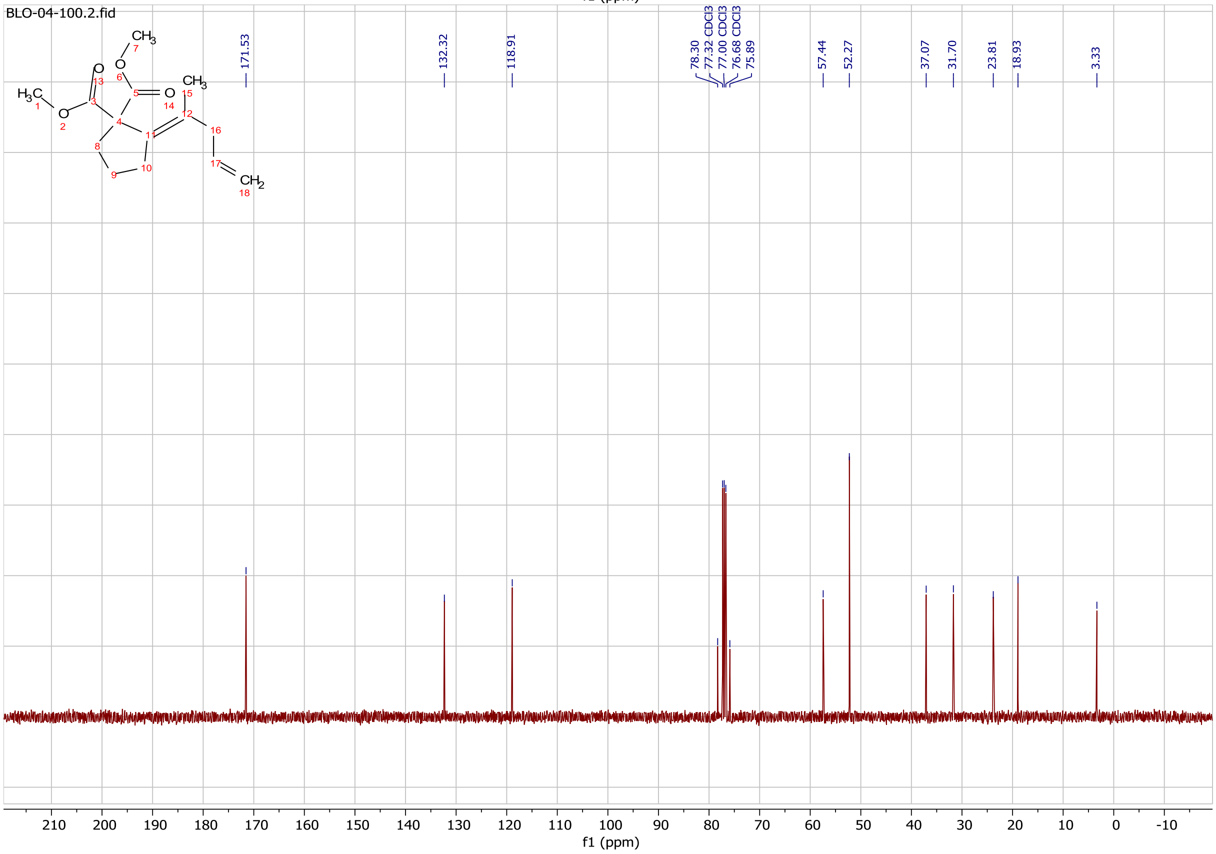

Methyl *(E)*-1-acetyl-2-(1-phenylethylidene)cyclopentane-1-carboxylate (26a)

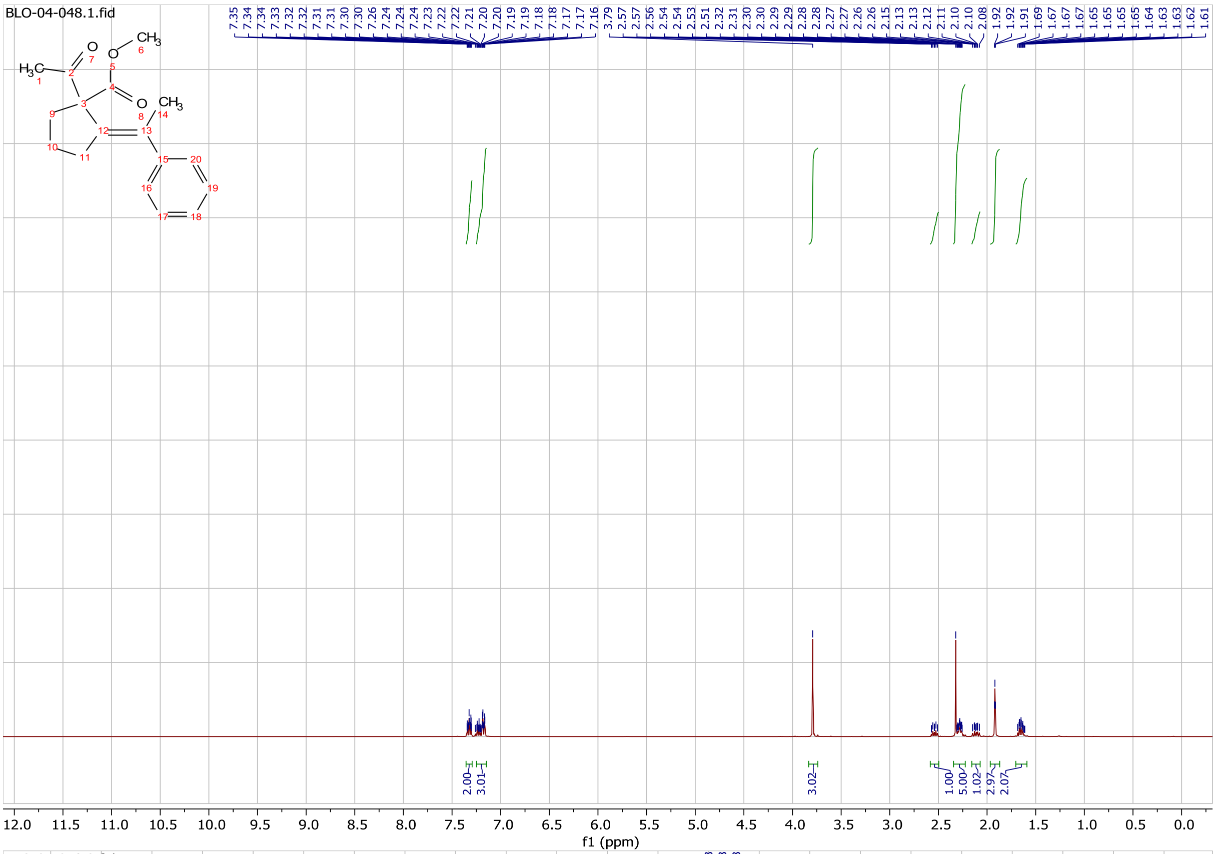

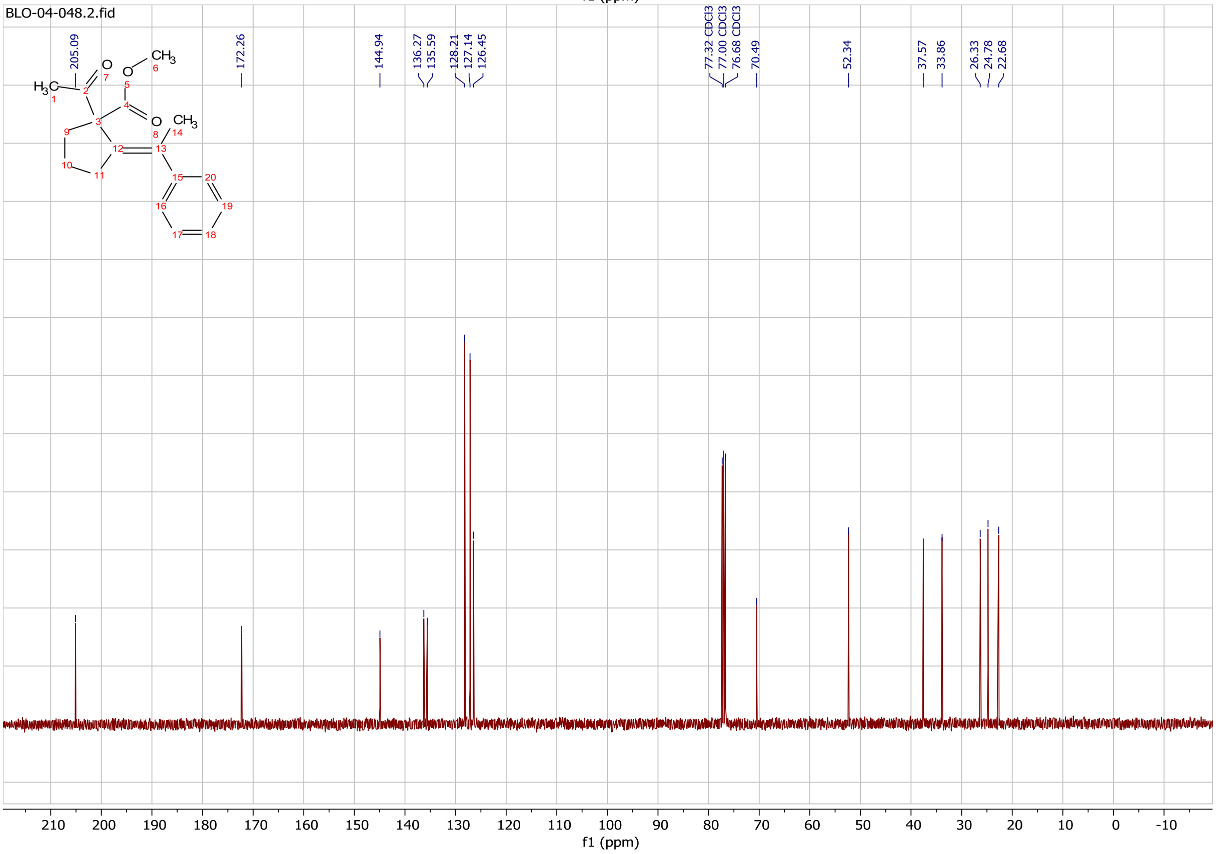

Methyl *(E)*-1-acetyl-2-(1-(4-methoxyphenyl)ethylidene)cyclopentane-1-carboxylate (26b)

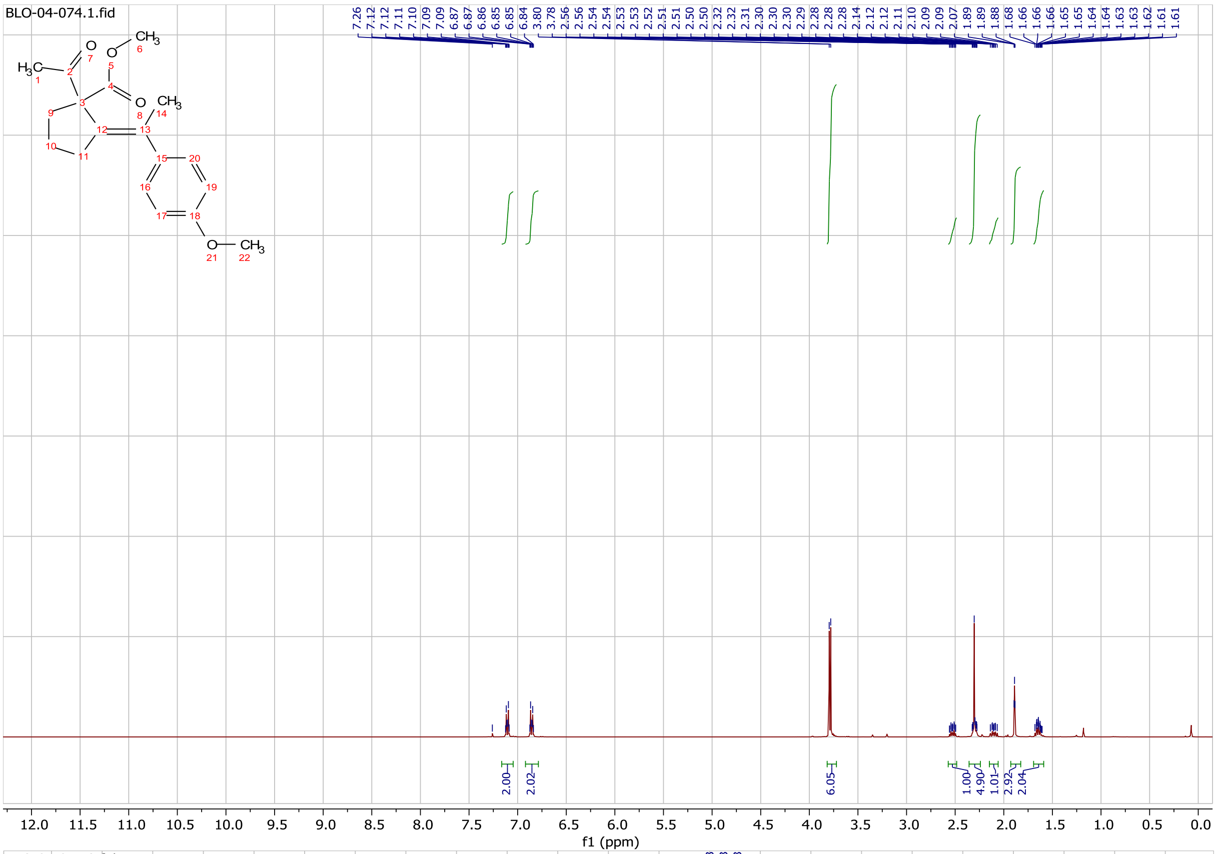

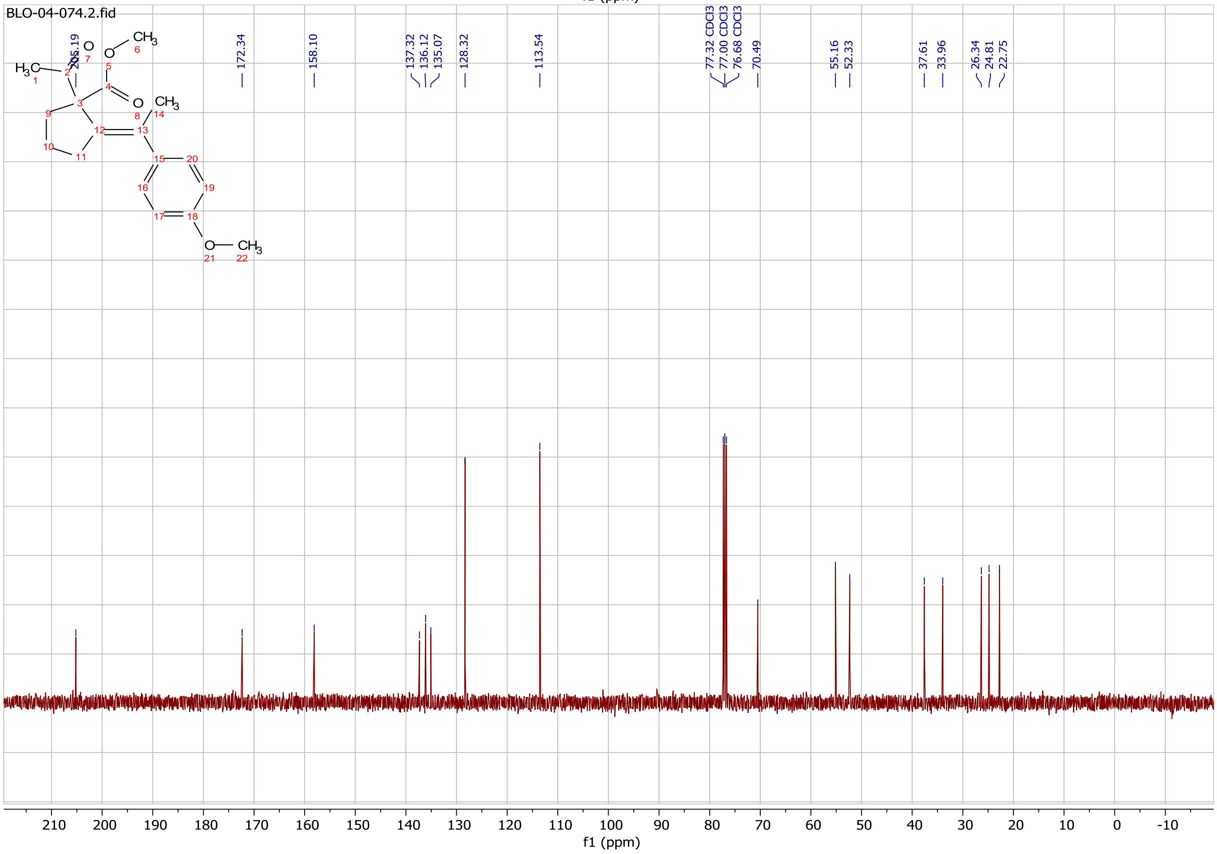

Methyl (*E*)-1-acetyl-2-(1-(4-cyanophenyl)ethylidene)cyclopentane-1-carboxylate (26c)

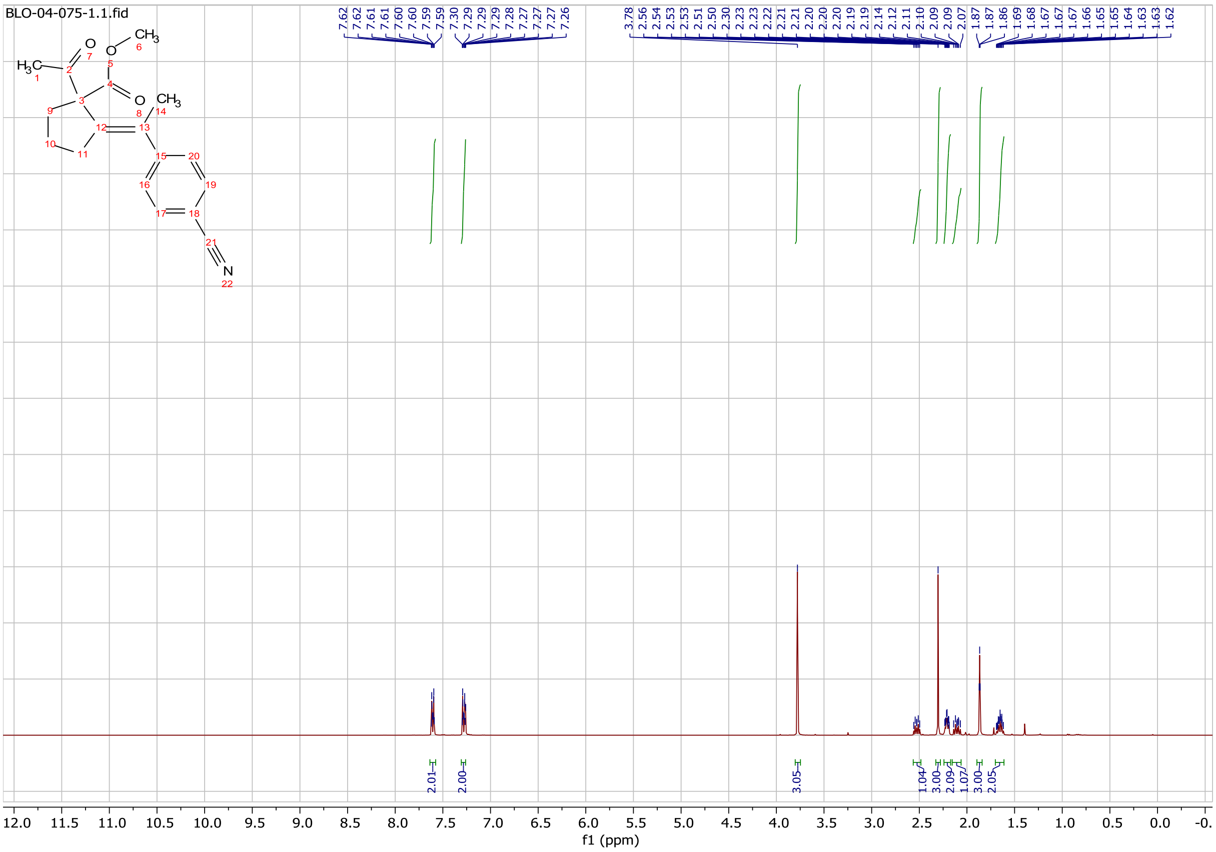

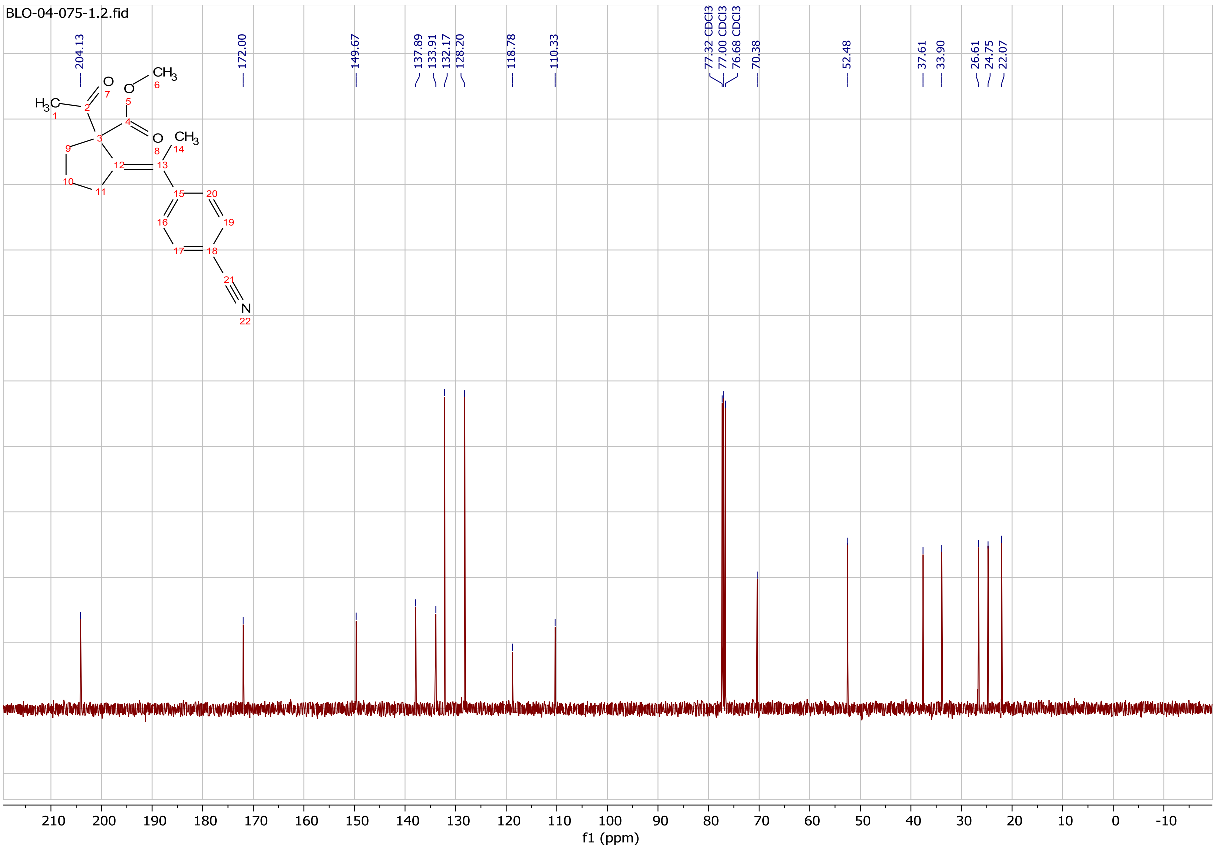

Isopropyl (*E*)-1-acetyl-2-(1-phenylethylidene)cyclopentane-1-carboxylate (27a)

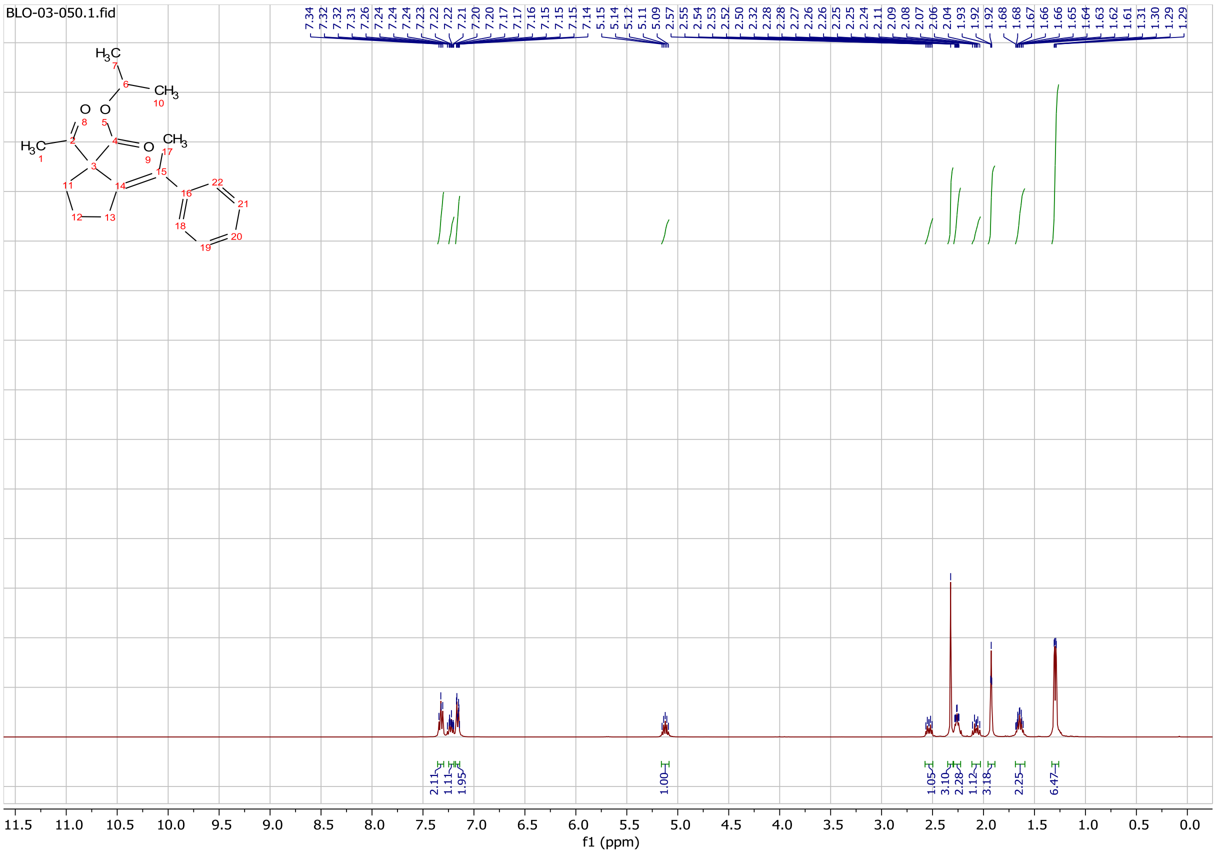

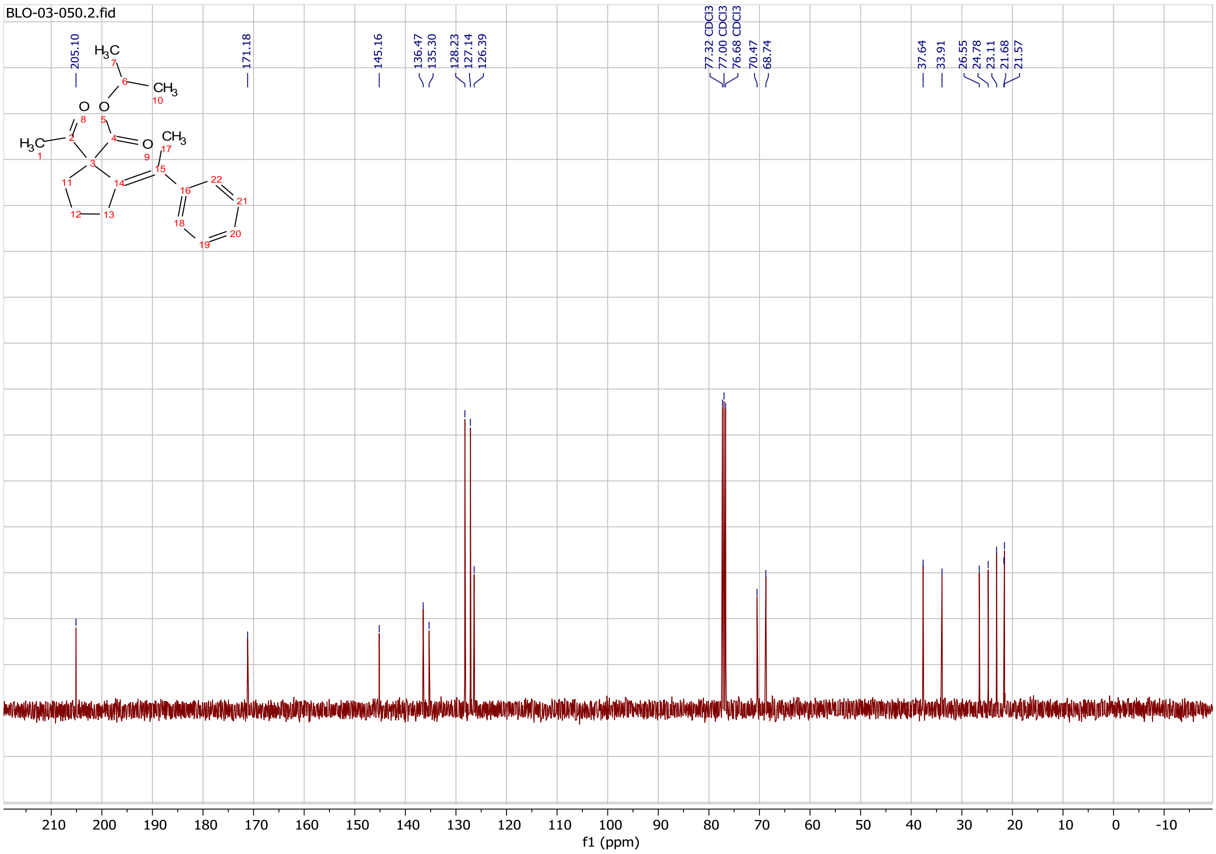

Isopropyl *(E)*-1-acetyl-2-(1-(4-methoxyphenyl)ethylidene)cyclopentane-1-carboxylate (27b)

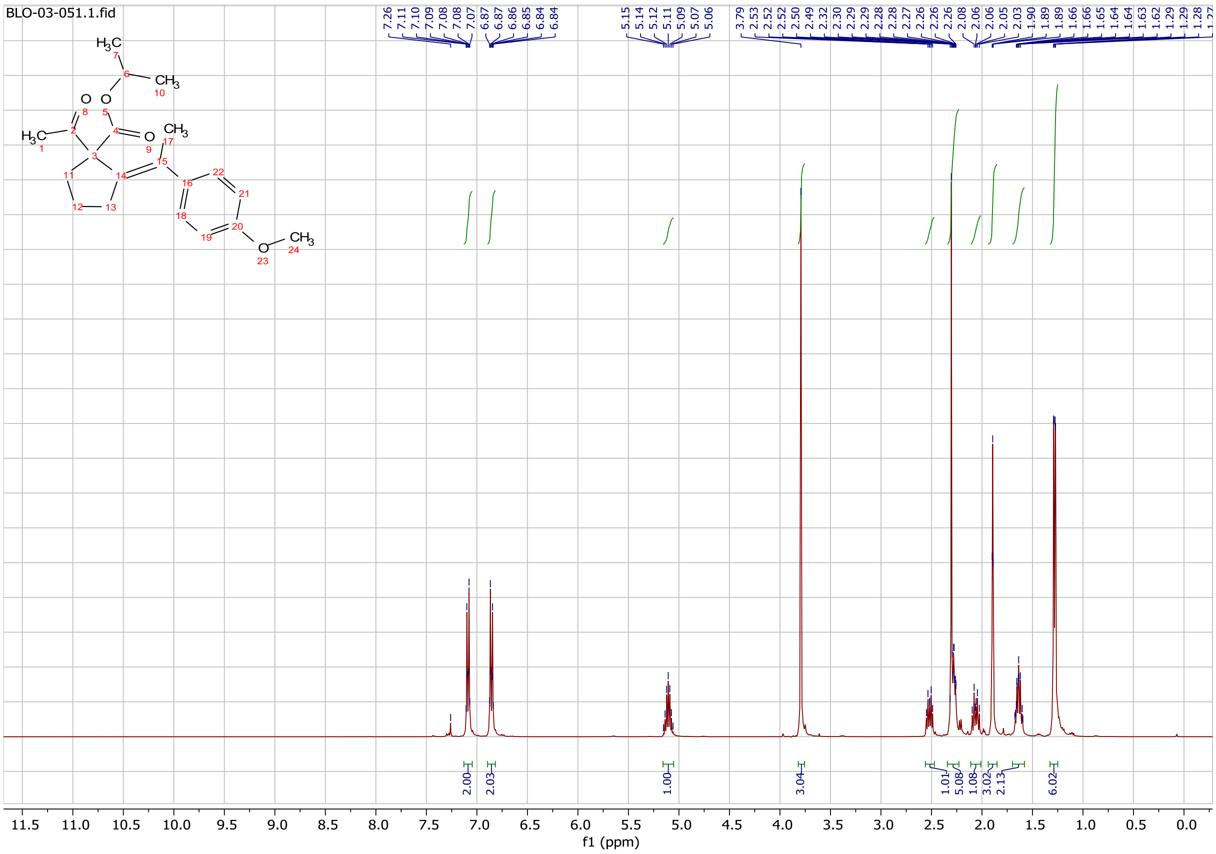

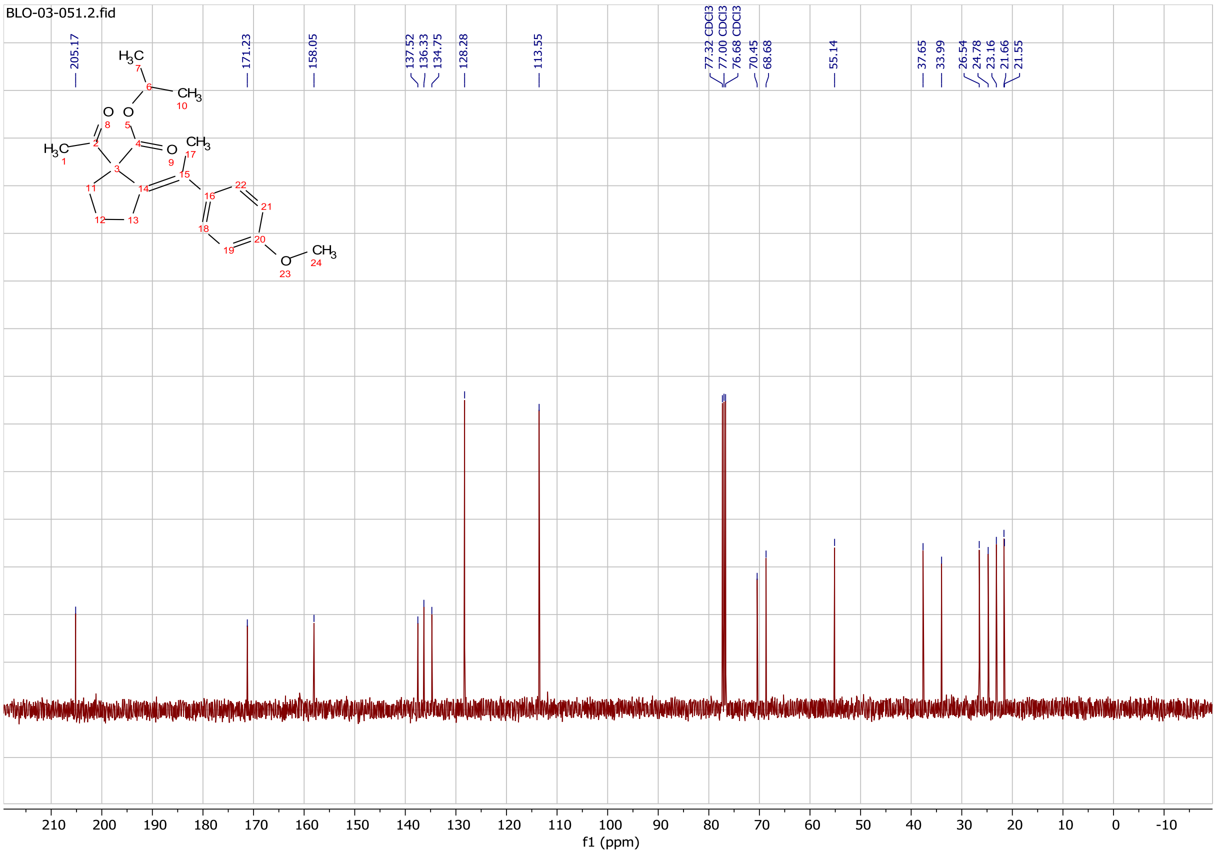

Isopropyl *(E)*-1-acetyl-2-(1-(4-cyanophenyl)ethylidene)cyclopentane-1-carboxylate (27c)

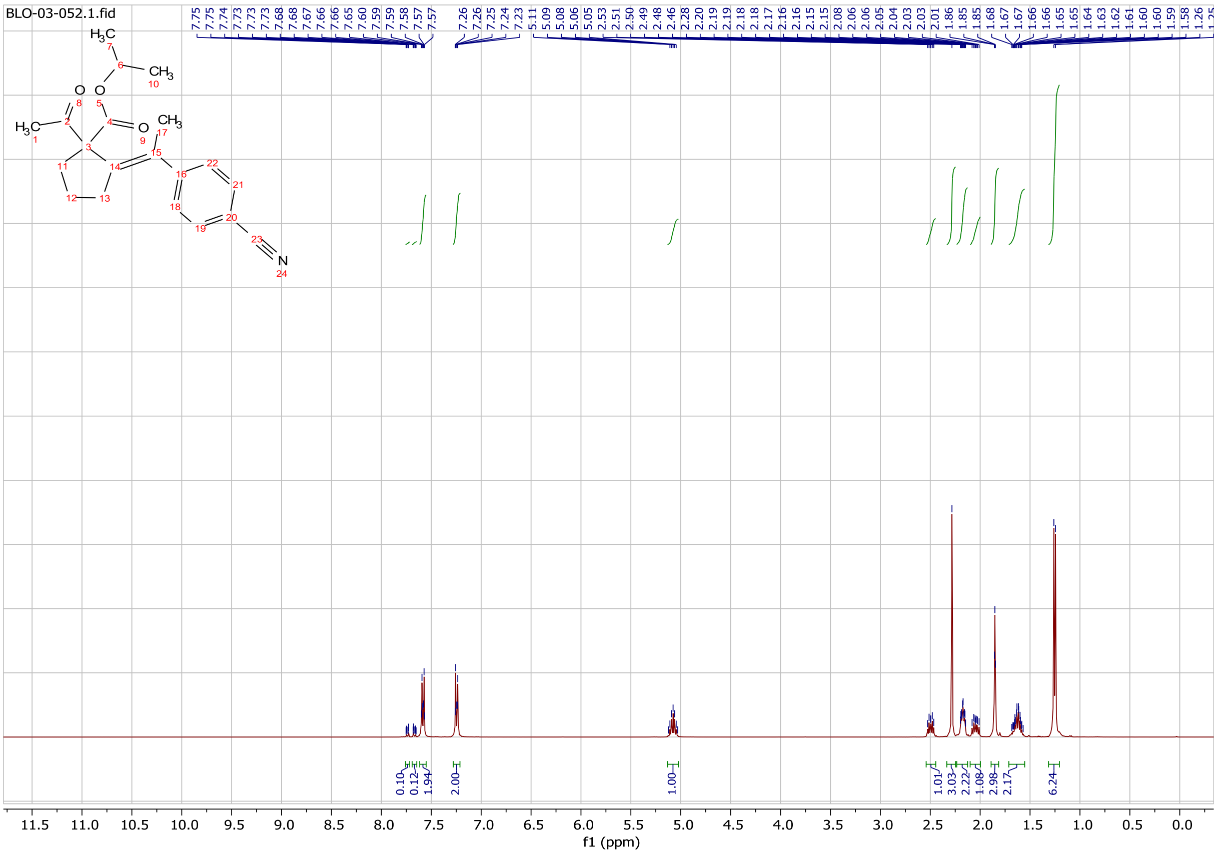
BLO-03-052.1.fid
7.75 7.75 7.74 7.73 7.73 7.73 7.68 7.68 7.67 7.66 7.66 7.65 7.60 7.59 7.59 7.58 7.57 7.57 7.57
7.26 7.26 7.25 7.24 7.23
5.11 5.09 5.08 5.06 5.05 2.53 2.51 2.50 2.49 2.48 2.46 2.28 2.20 2.19 2.19 2.18 2.18 2.17 2.16 2.16 2.15 2.15 2.08 2.06 2.06 2.05 2.04 2.03 2.03 2.01 1.86 1.85 1.85 1.68 1.67 1.67 1.66 1.66 1.65 1.65 1.64 1.63 1.62 1.61 1.60 1.60 1.59 1.58 1.26
0.10 0.12 1.94 2.00 1.00 1.01 3.03 2.22 1.08 2.98 2.17 6.24
11.5 11.0 10.5 10.0 9.5 9.0 8.5 8.0 7.5 7.0 6.5 6.0 5.5 5.0 4.5 4.0 3.5 3.0 2.5 2.0 1.5 1.0 0.5 0.0
f1 (ppm)

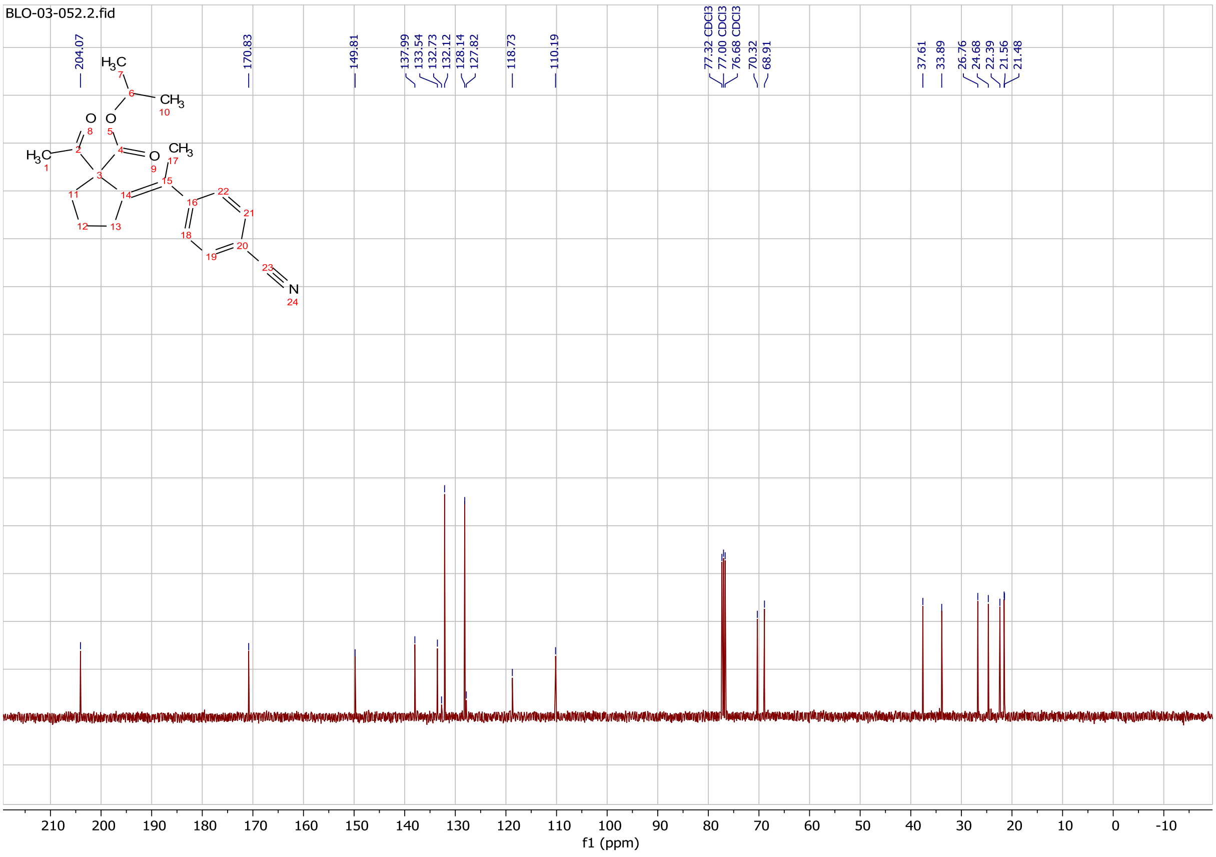
BLO-03-052.2.fid
204.07
170.83
149.81
137.99 133.54 132.73 132.12 128.14 127.82
118.73
110.19
77.32 CDCl3 77.00 CDCl3 76.68 CDCl3 70.32 68.91
37.61 33.89 26.76 24.68 22.39 21.56 21.48
210 200 190 180 170 160 150 140 130 120 110 100 90 80 70 60 50 40 30 20 10 0 -10
f1 (ppm)

Methyl *(E)*-1-isobutyryl-2-(1-phenylethylidene)cyclopentane-1-carboxylate (28a)

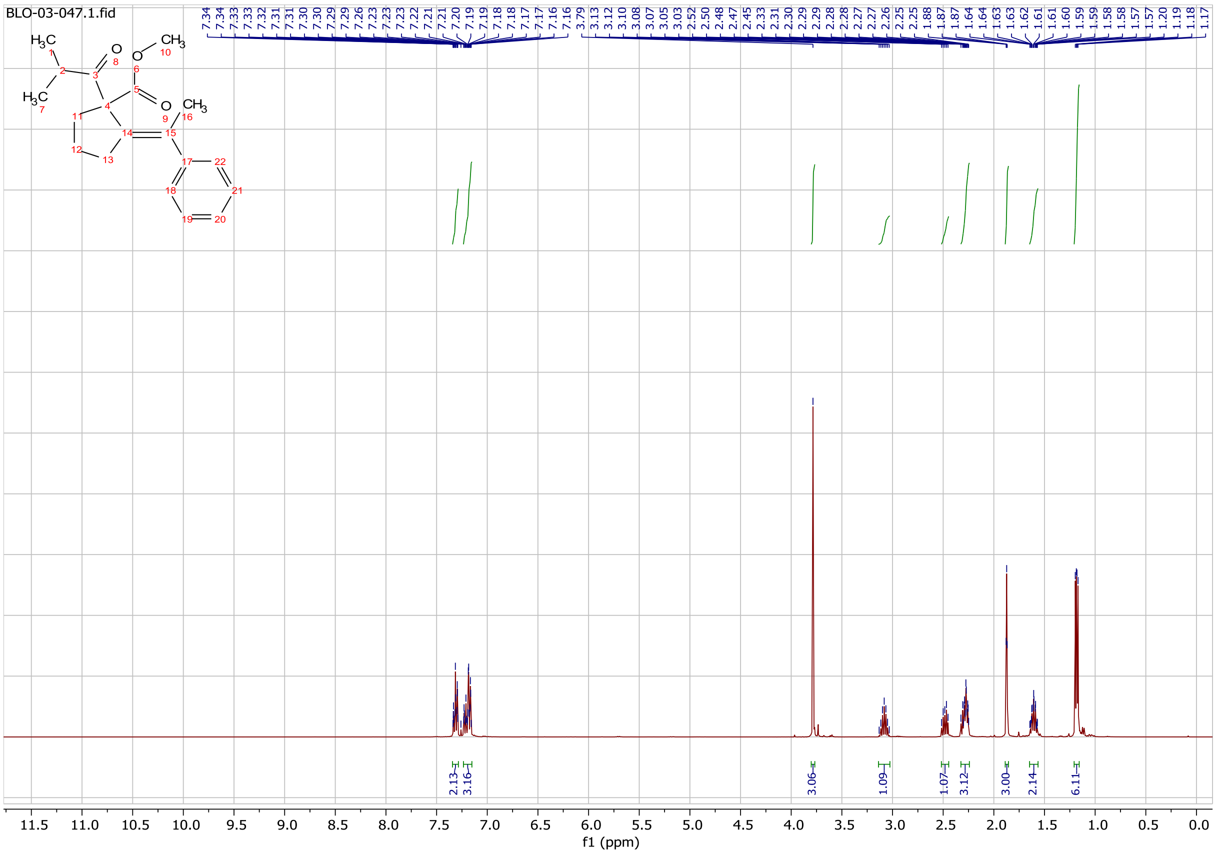

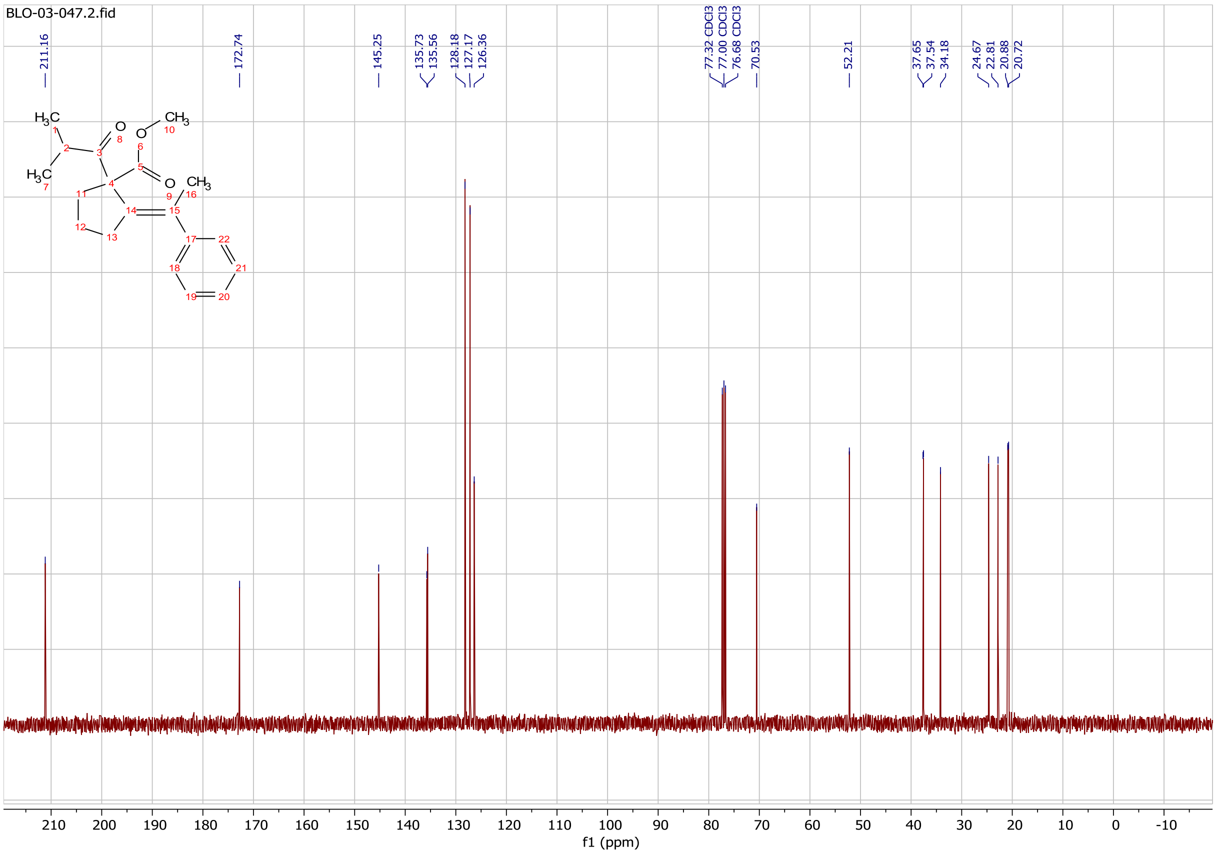

Methyl *(E)*-1-isobutyryl-2-(1-(4-methoxyphenyl)ethylidene)cyclopentane-1-carboxylate (28b)

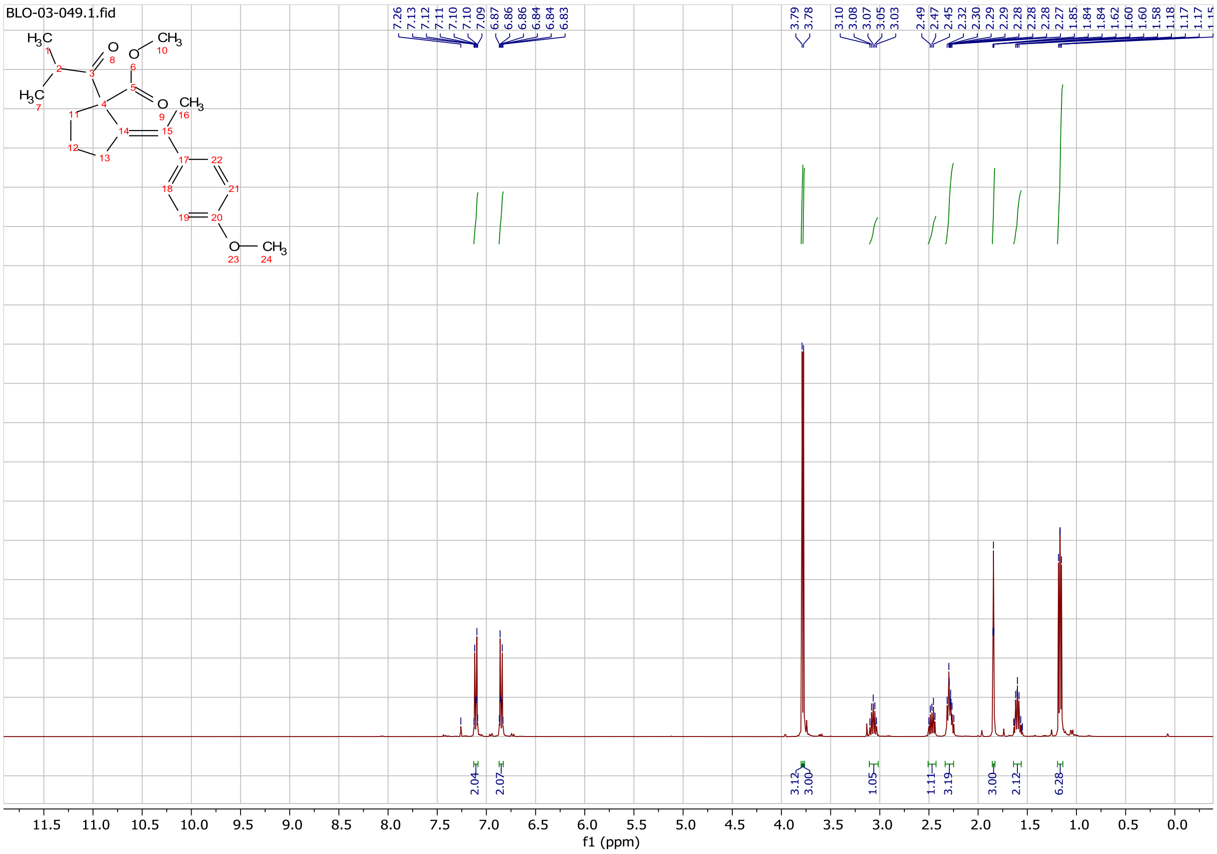

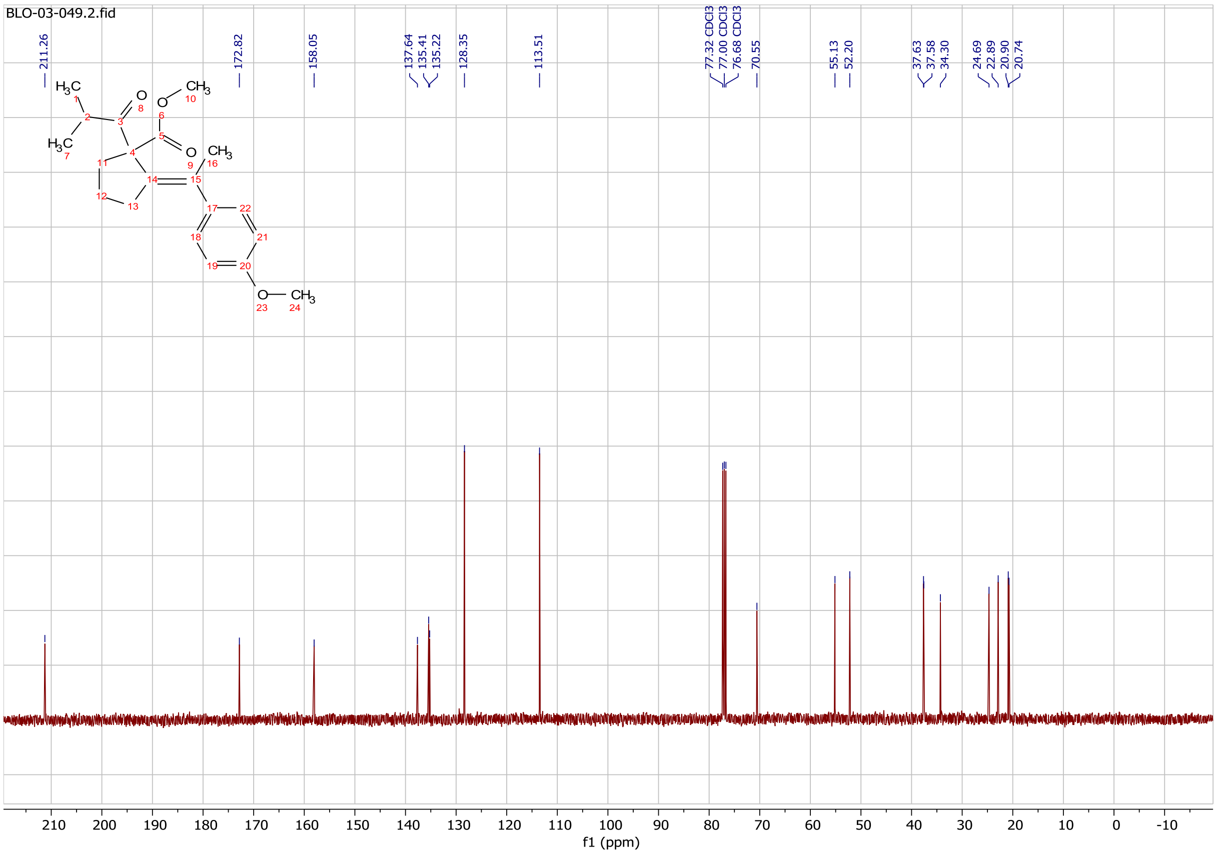

Methyl*(E)*-2-(1-(4-cyanophenyl)ethylidene)-1-isobutyrylcyclopentane-1-carboxylate (28c)

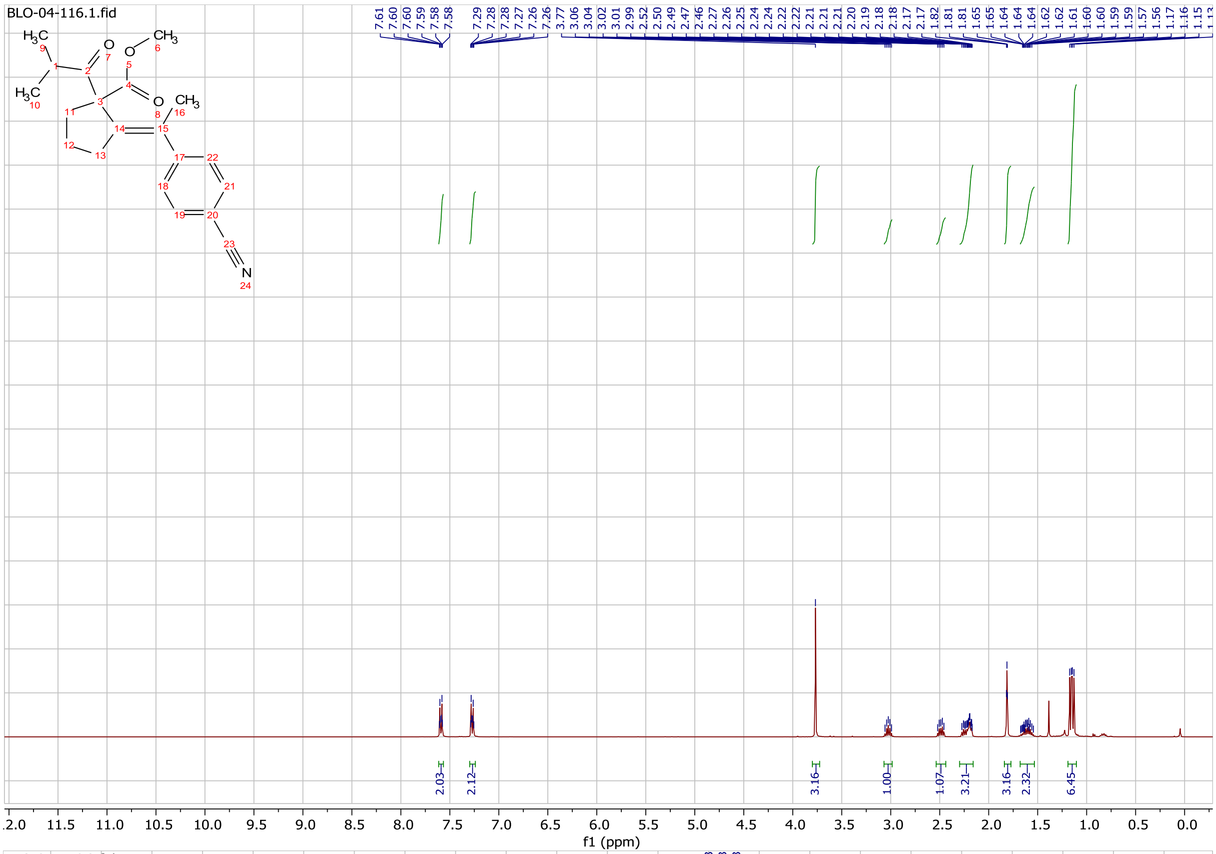

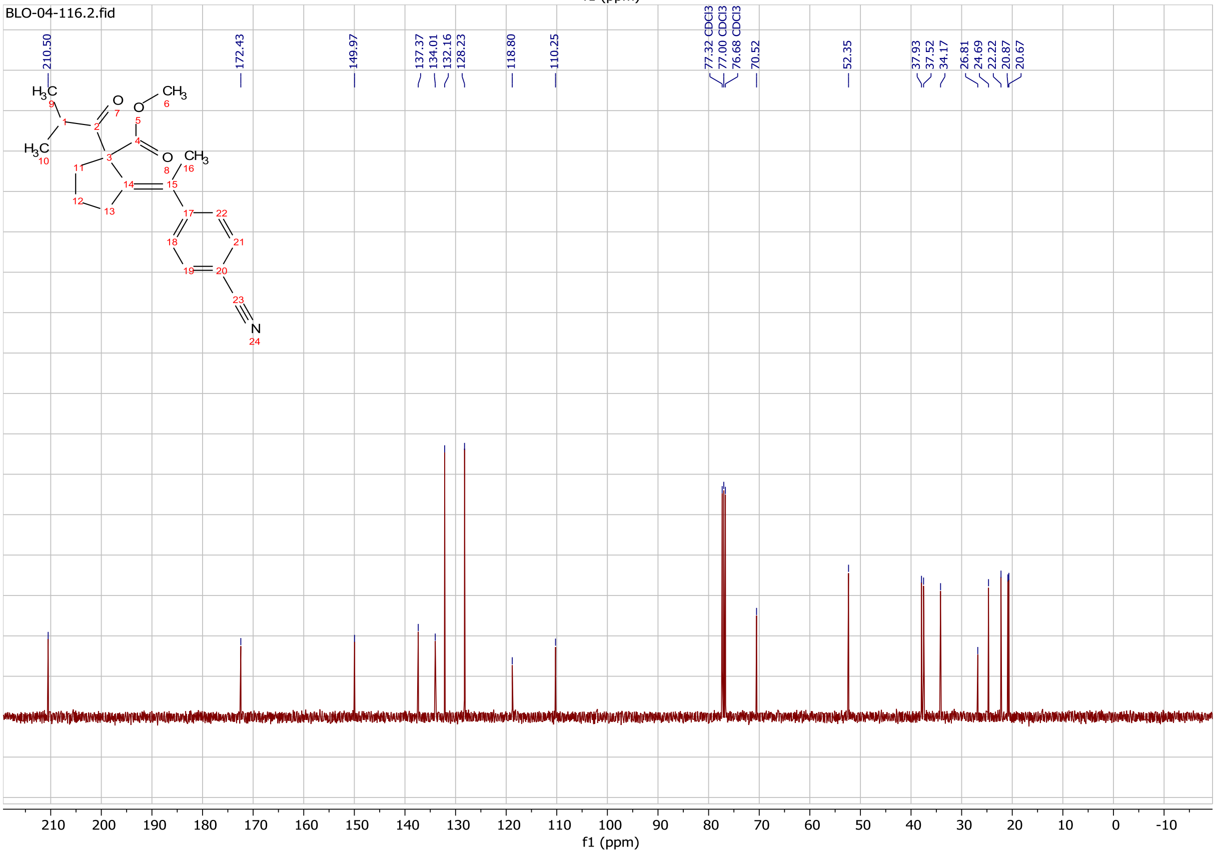

Methyl *(E)*-1-cyano-2-(1-phenylethylidene)cyclopentane-1-carboxylate (29a)

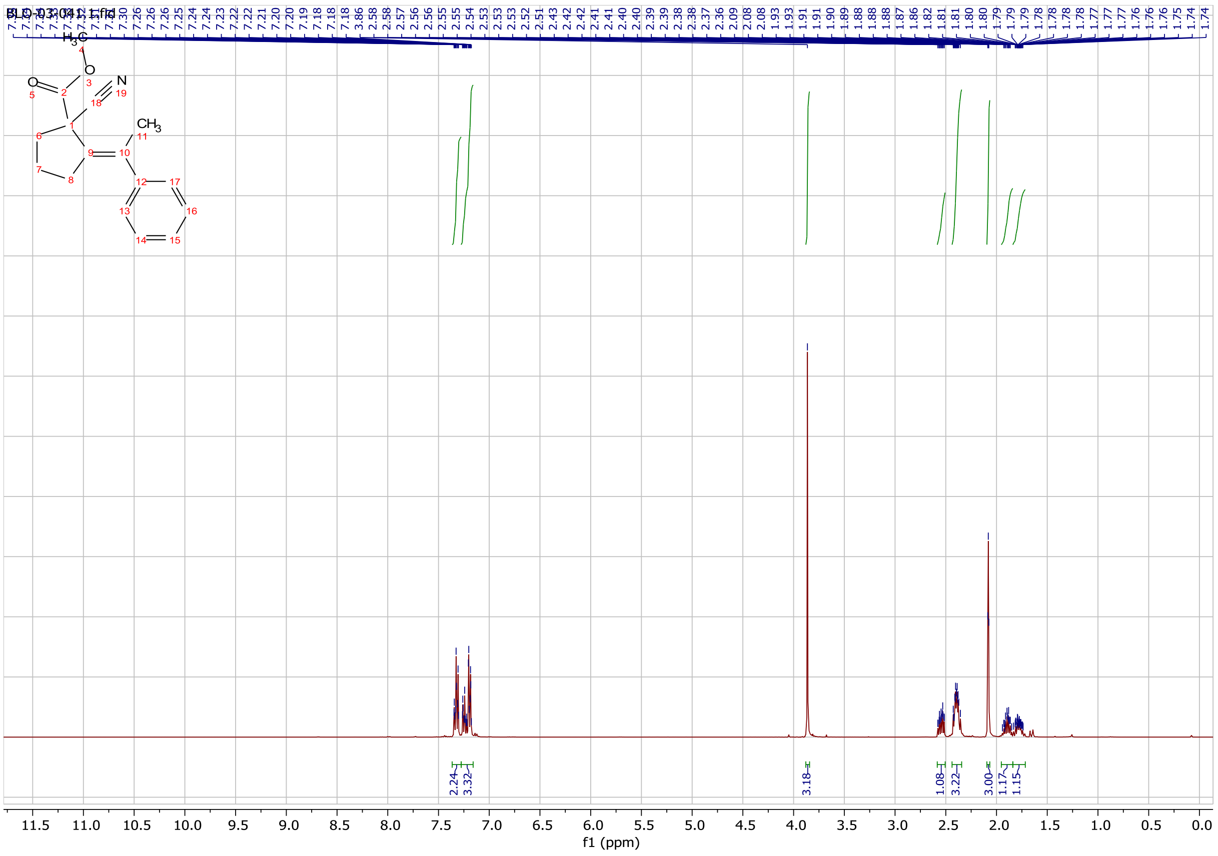

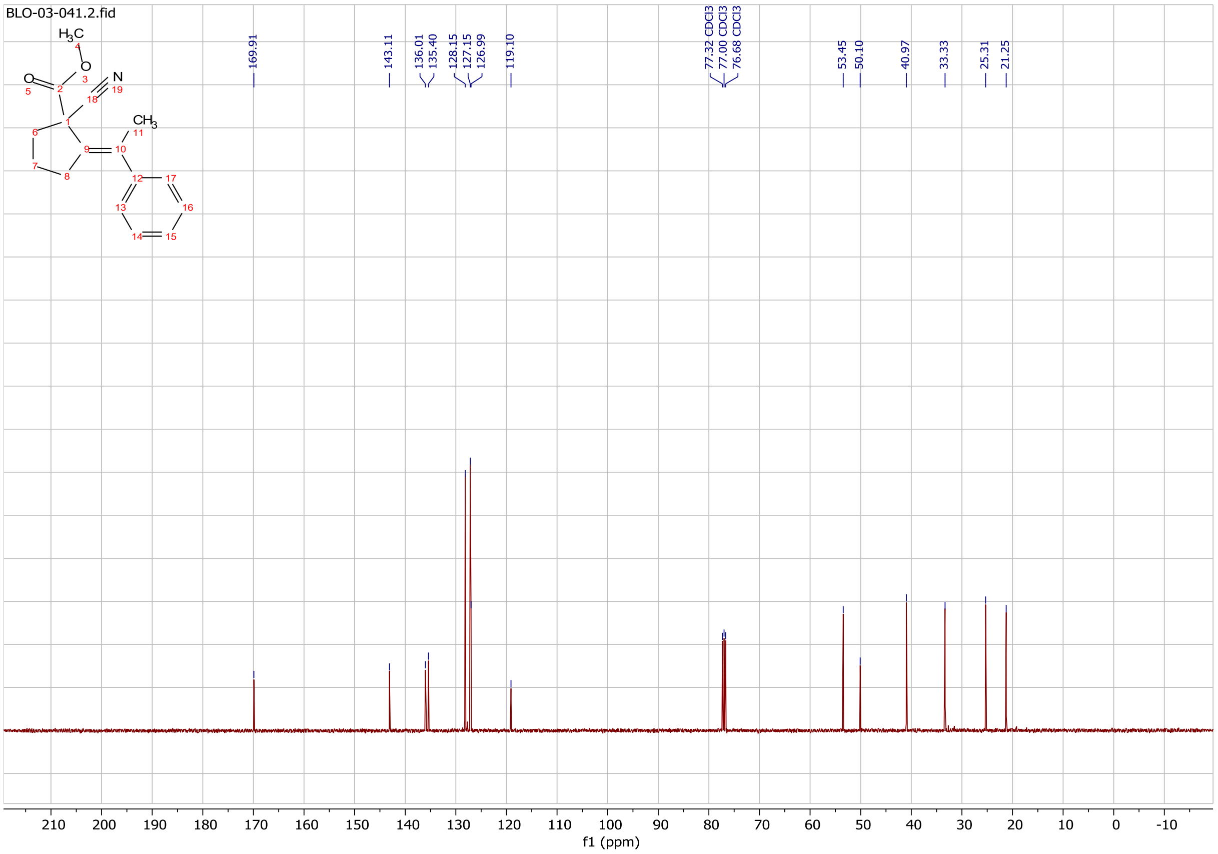

Methyl *(E)*-1-cyano-2-(1-(4-methoxyphenyl)ethylidene)cyclopentane-1-carboxylate (29a)

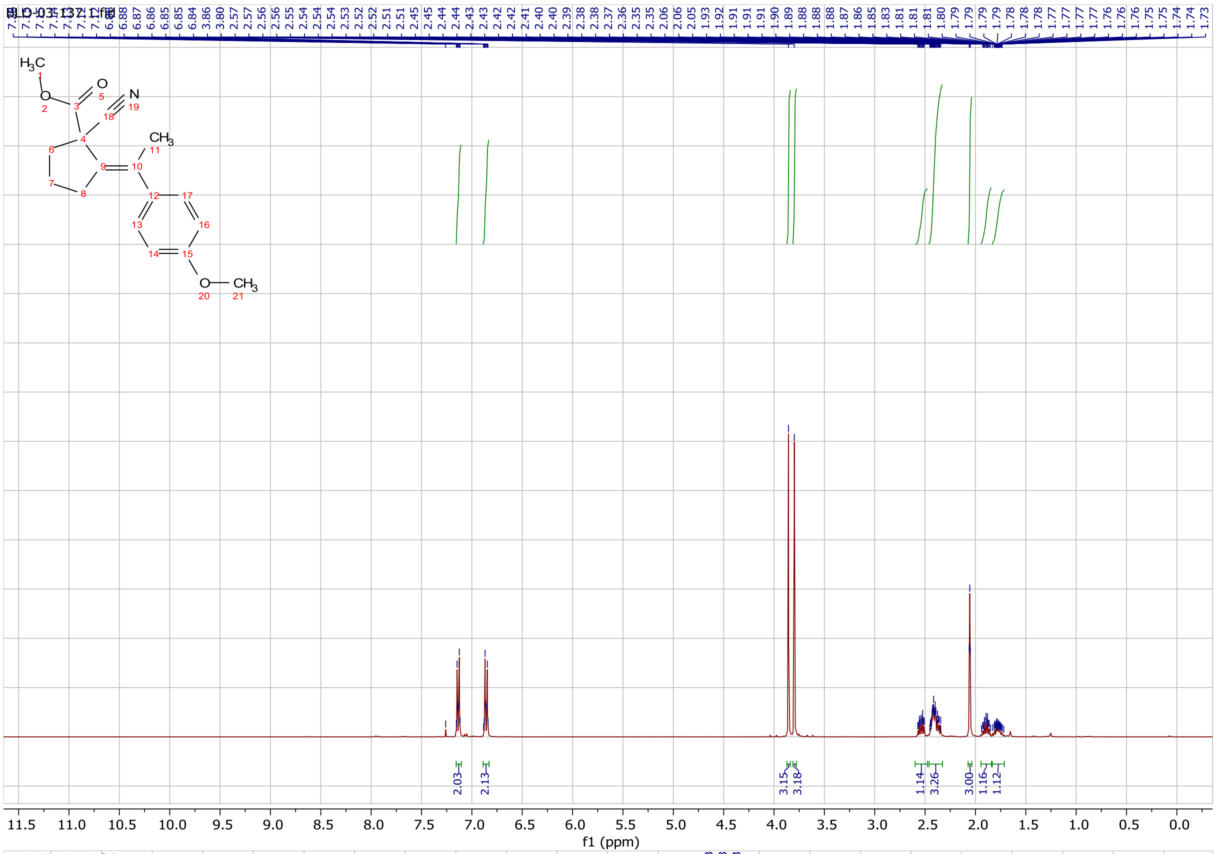

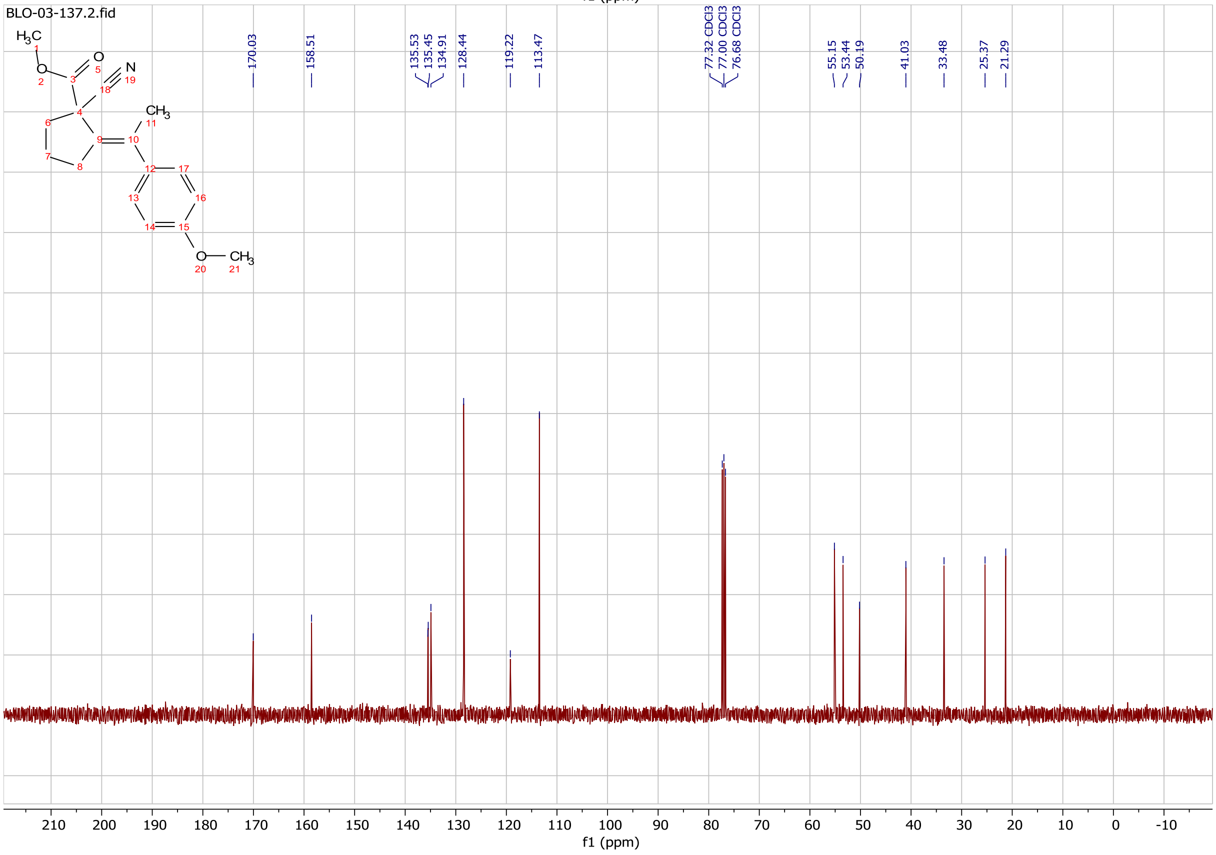

Methyl *(E)*-1-cyano-2-(1-(4-cyanophenyl)ethylidene)cyclopentane-1-carboxylate (29a)

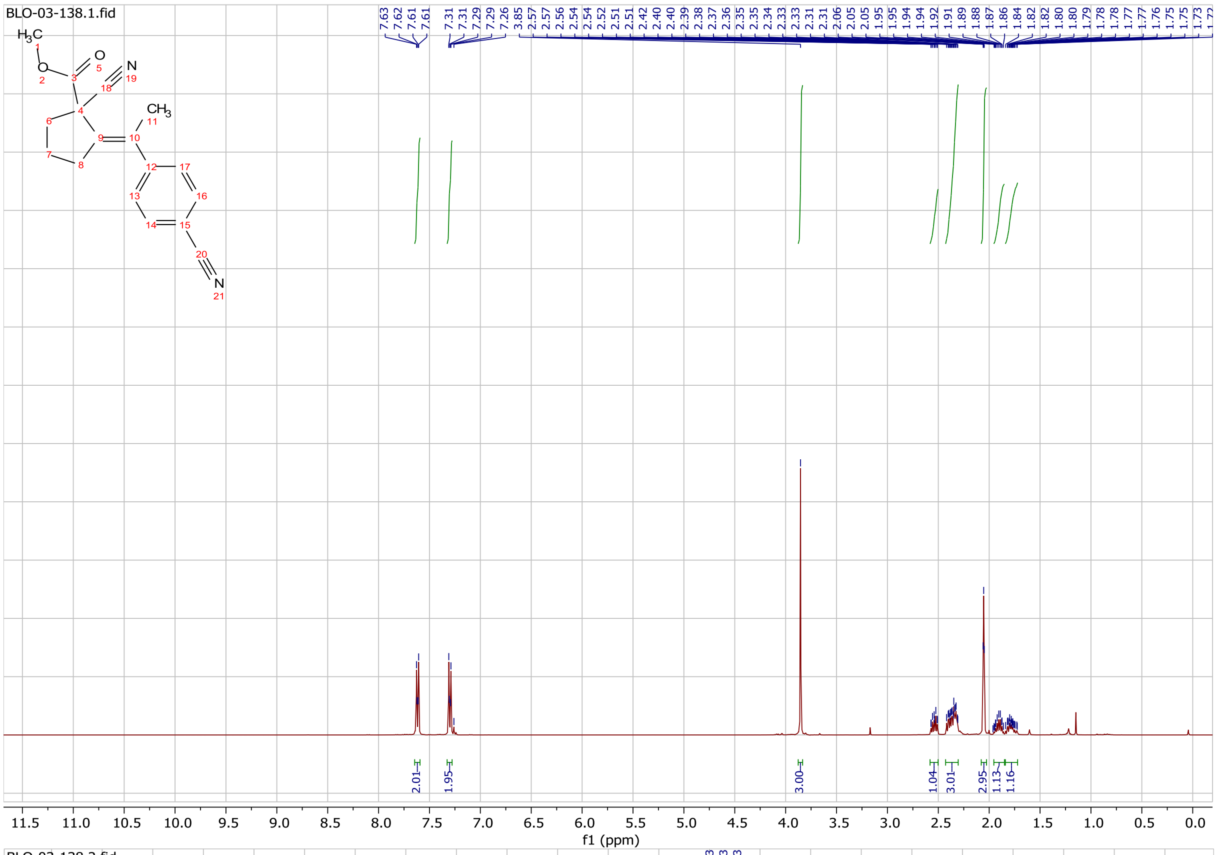

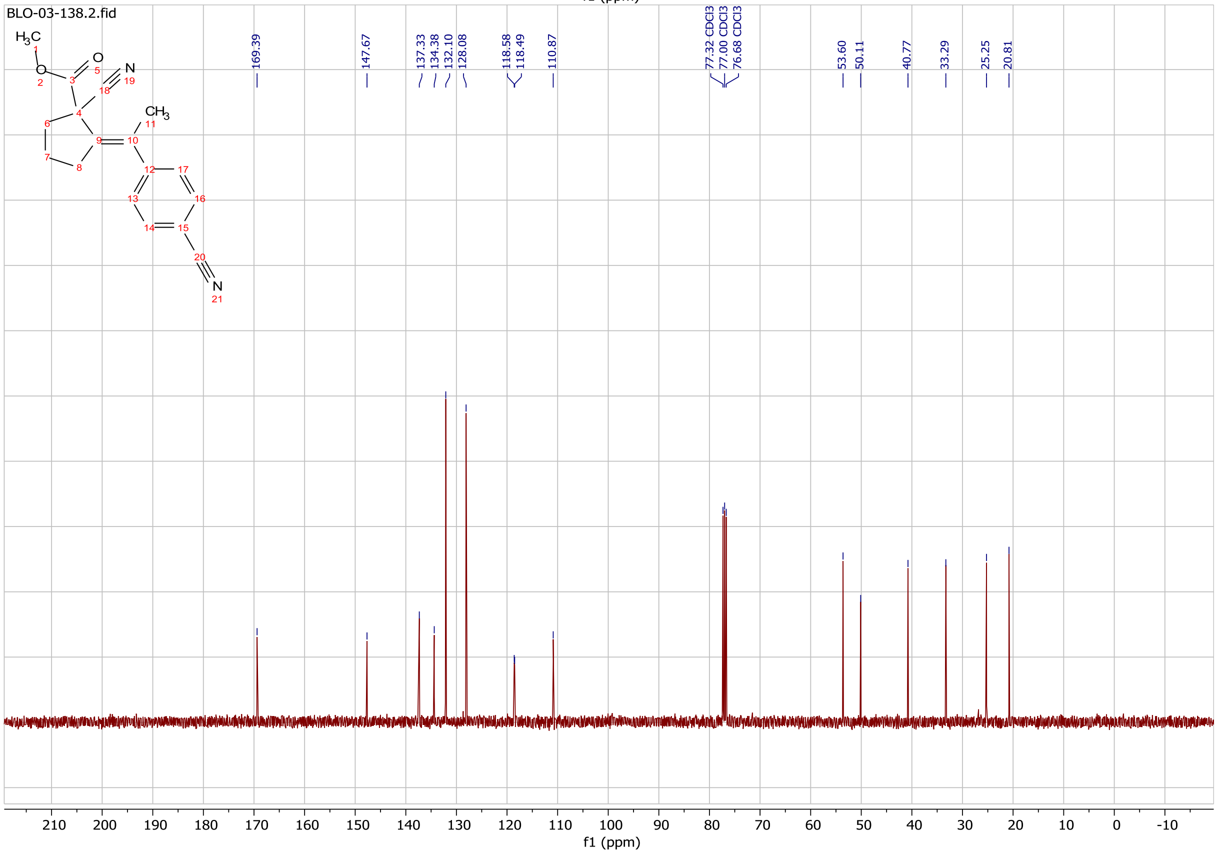

(E)-1-(2-(1-phenylethylidene)-1-(phenylsulfonyl)cyclopentyl)ethan-1-one (30a)

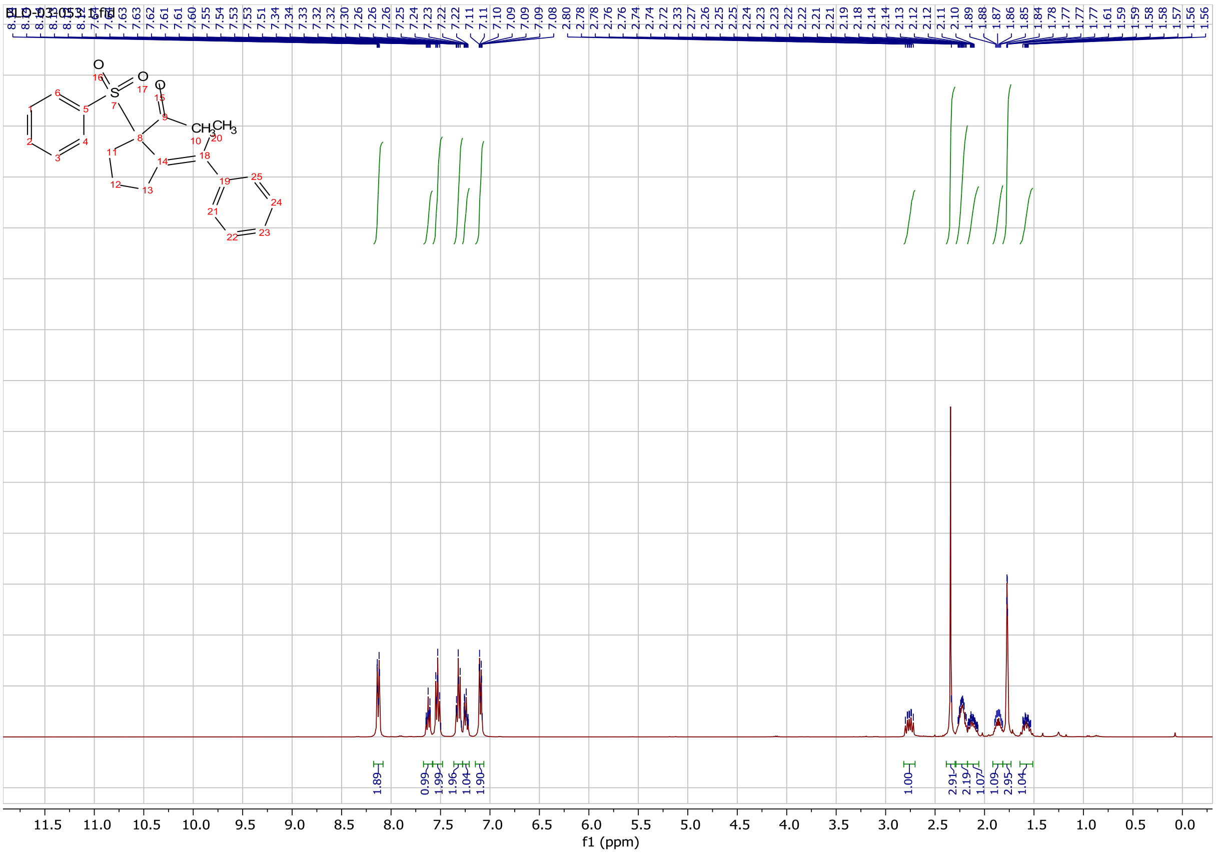

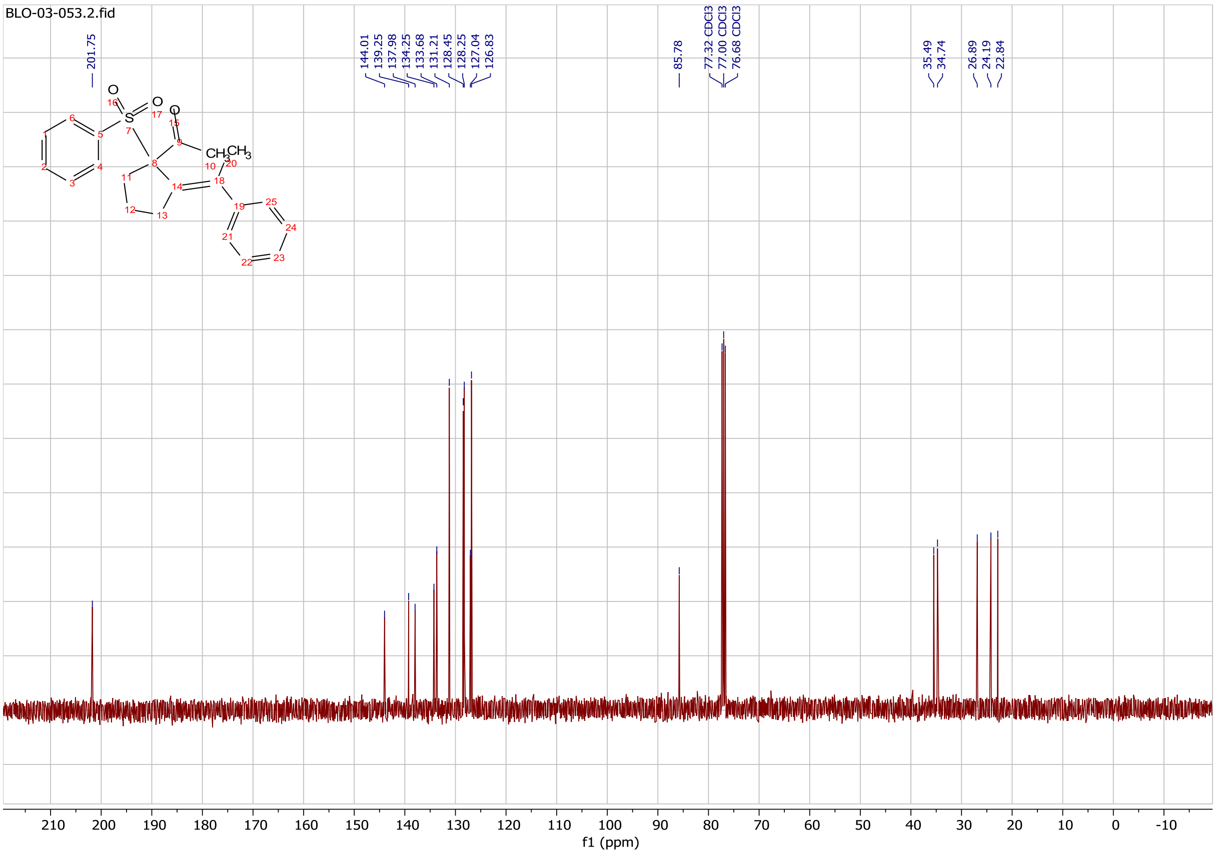

(*E*)-1-(2-(1-(4-methoxyphenyl)ethylidene)-1-(phenylsulfonyl) cyclopentyl)ethan-1-one (30b)

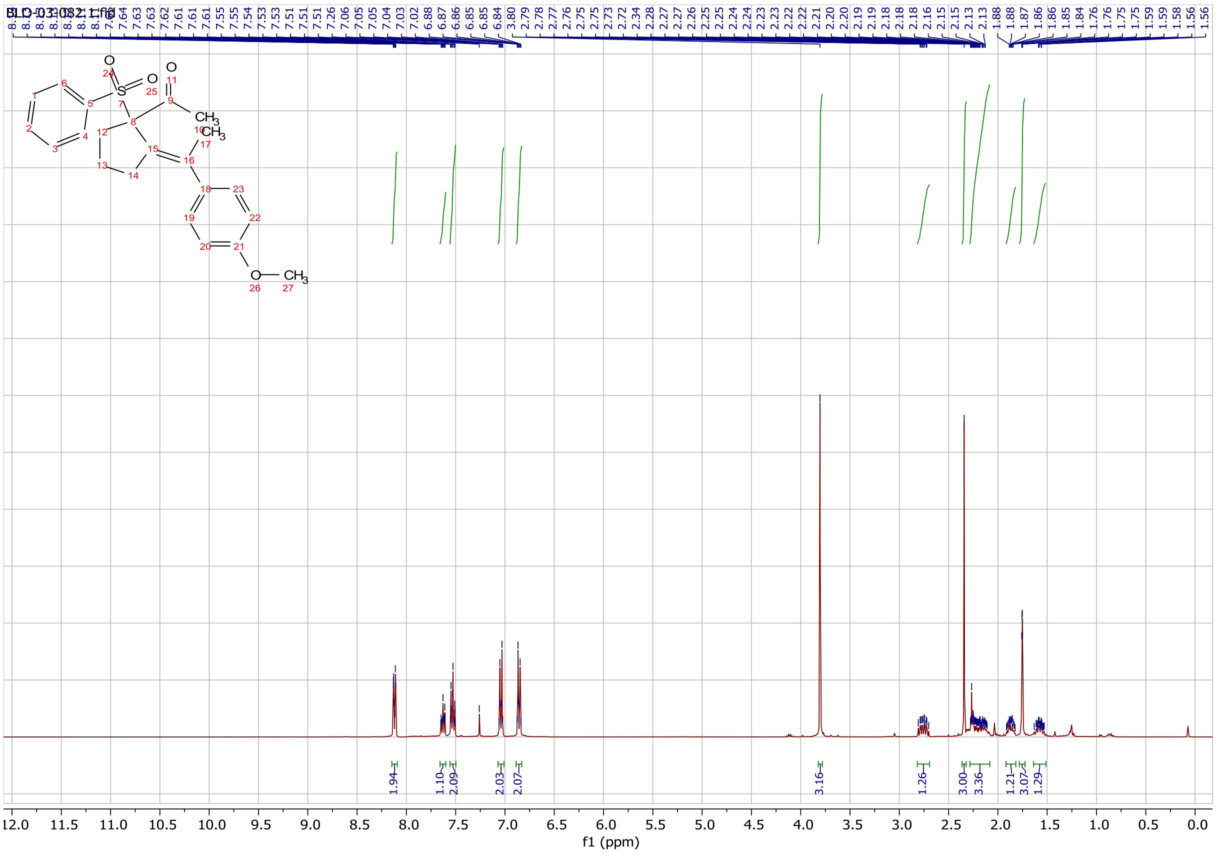

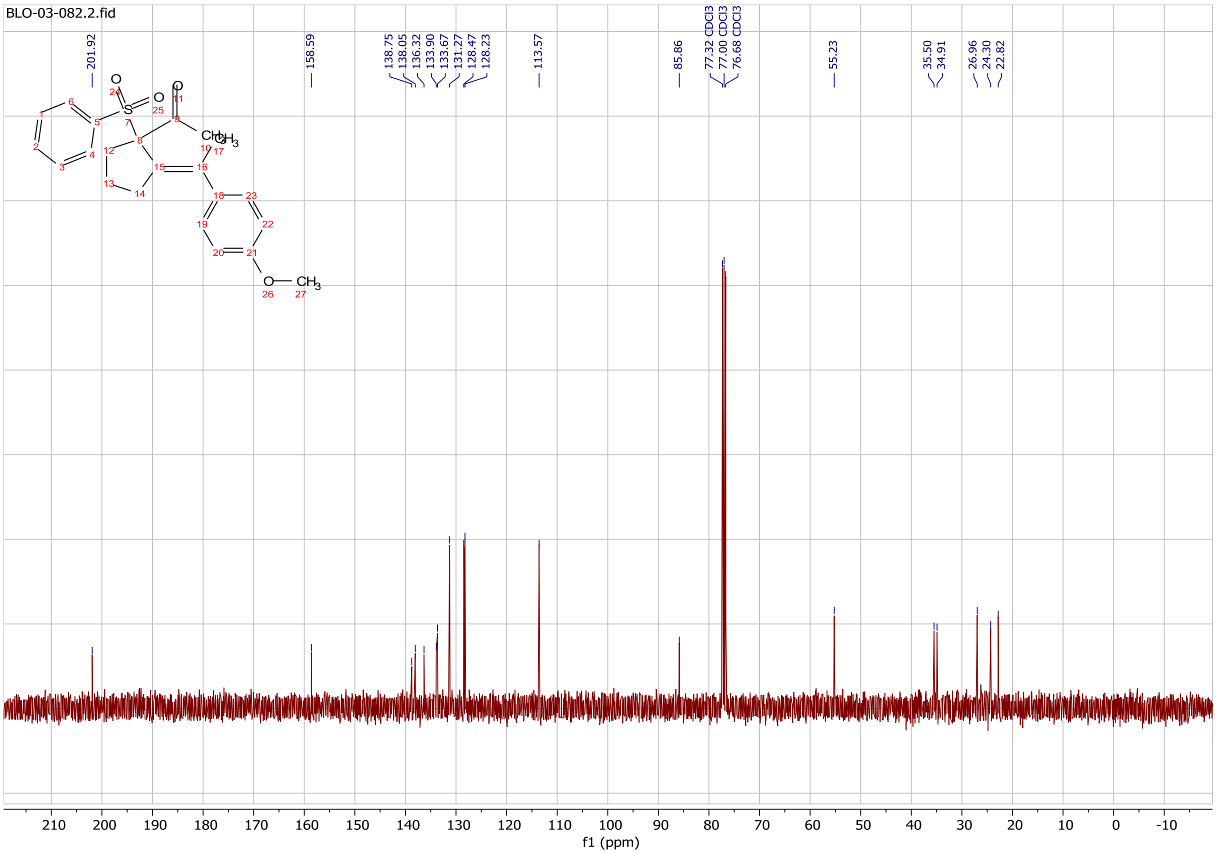

(*E*)-4-(1-(2-acetyl-2-(phenylsulfonyl)cyclopentylidene)ethyl)benzonitrile (30c)

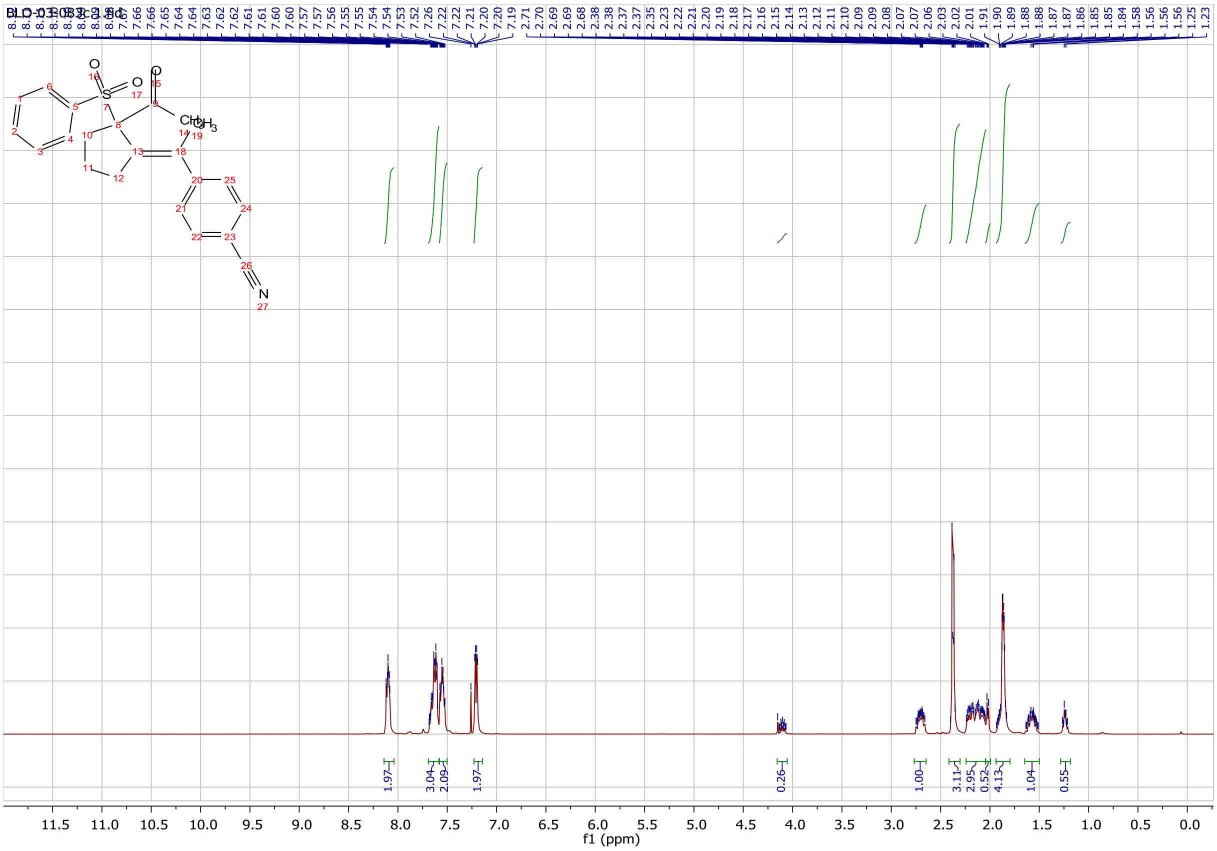

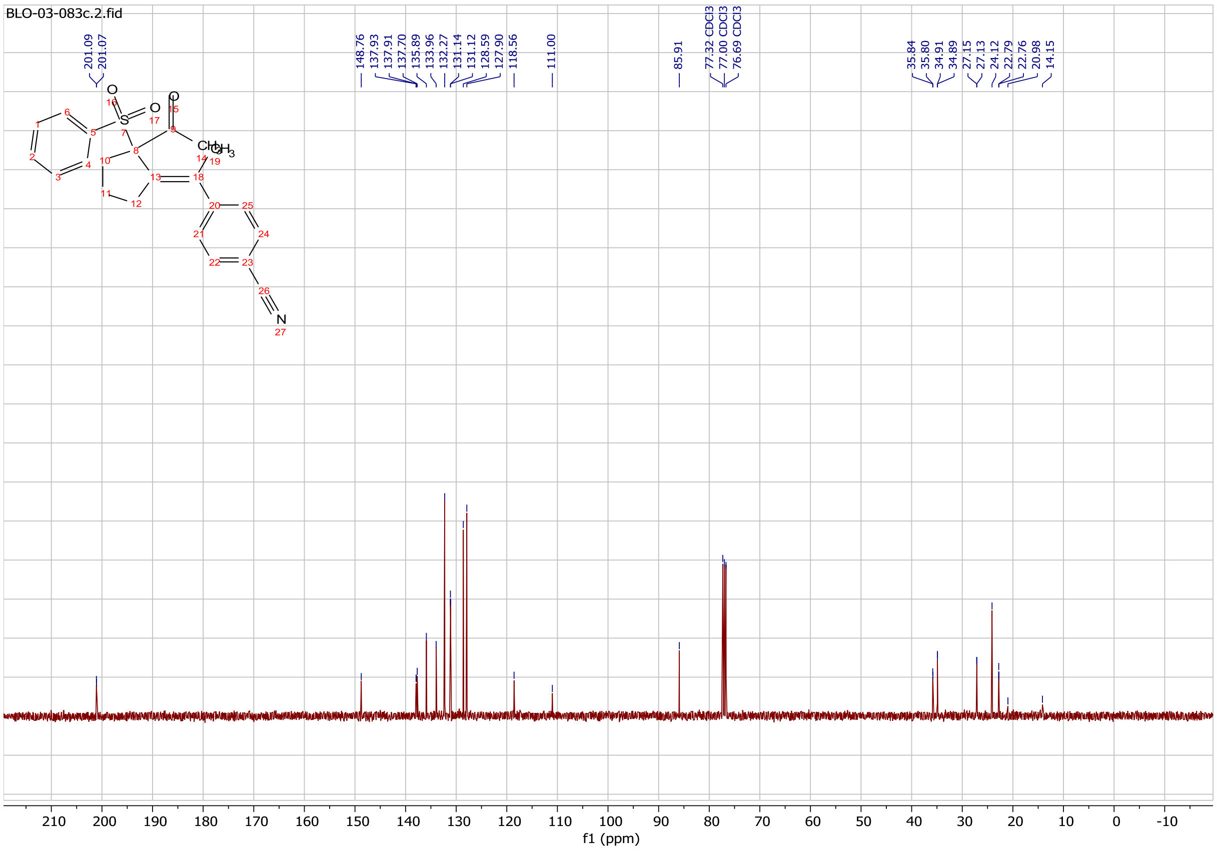

Ethyl *(E)*-1-(methylsulfonyl)-2-(1-phenylethylidene)cyclopentane-1-carboxylate (31a)

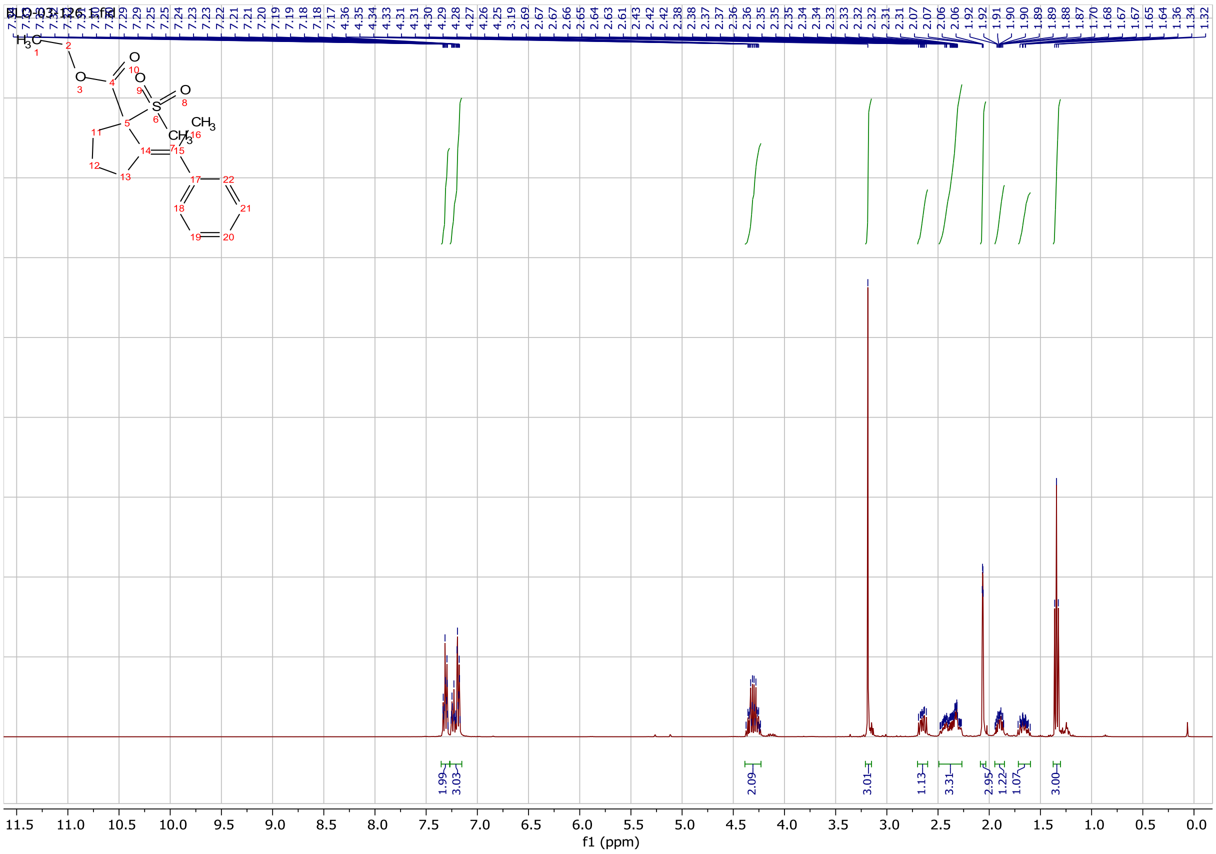

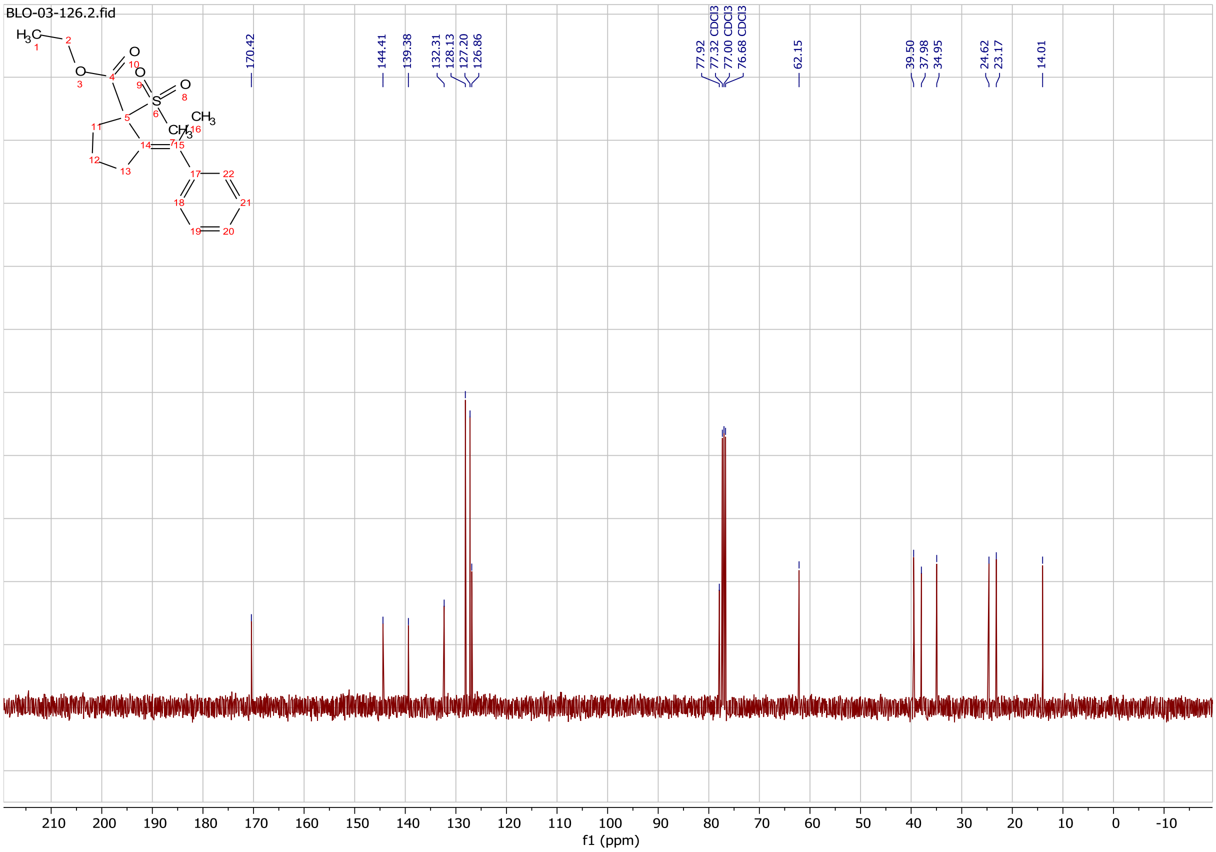

Ethyl (*E*)-2-(1-(4-methoxyphenyl)ethylidene)-1-(methylsulfonyl)cyclopentane-1-carboxylate (31b)

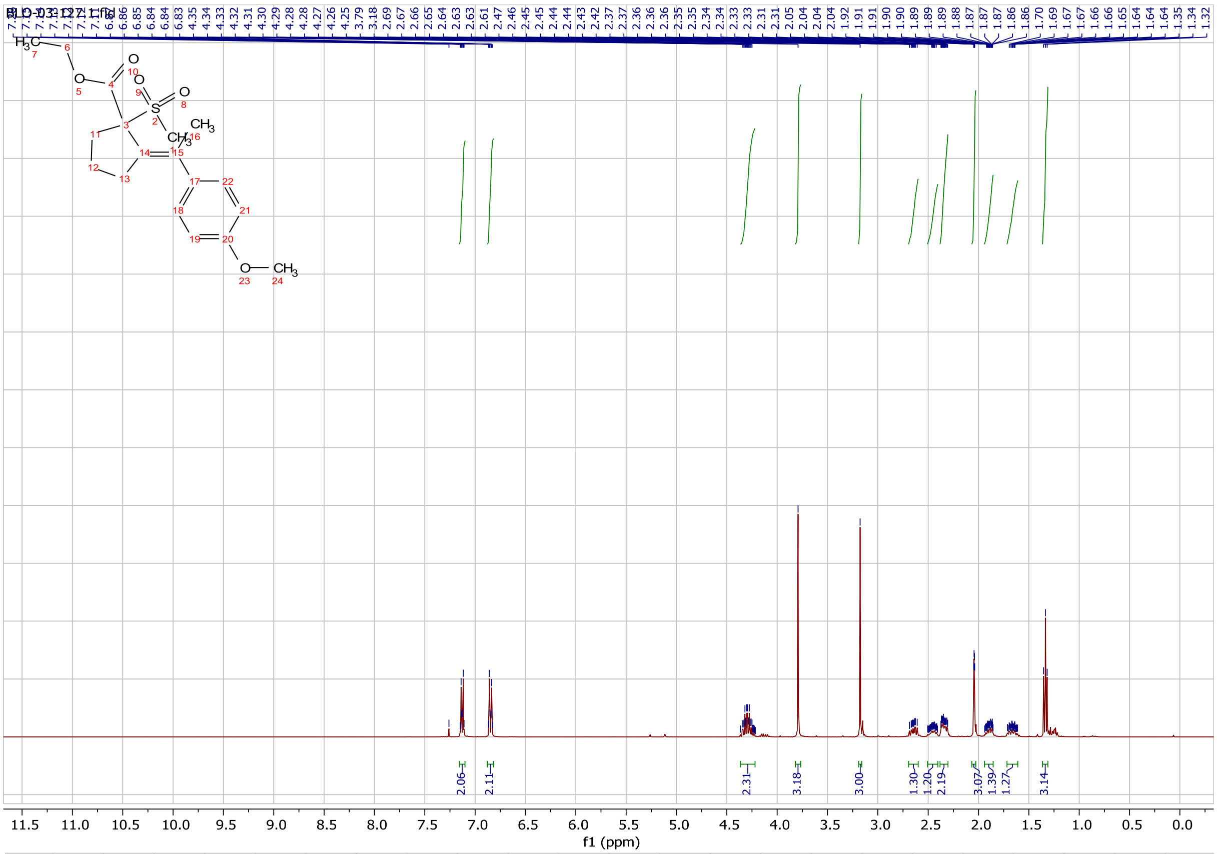

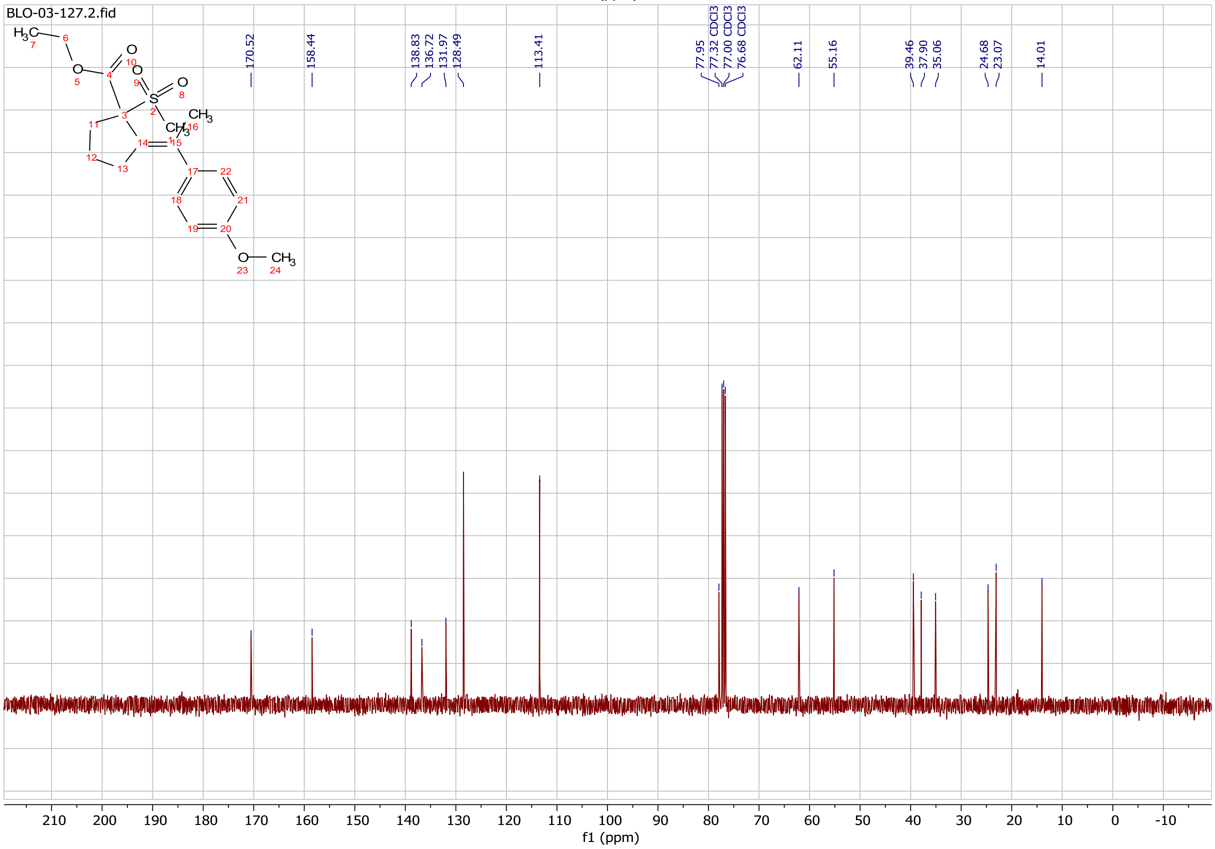

Ethyl *(E)*-2-(1-(4-cyanophenyl)ethylidene)-1-(methylsulfonyl)cyclopentane-1-carboxylate (31c)

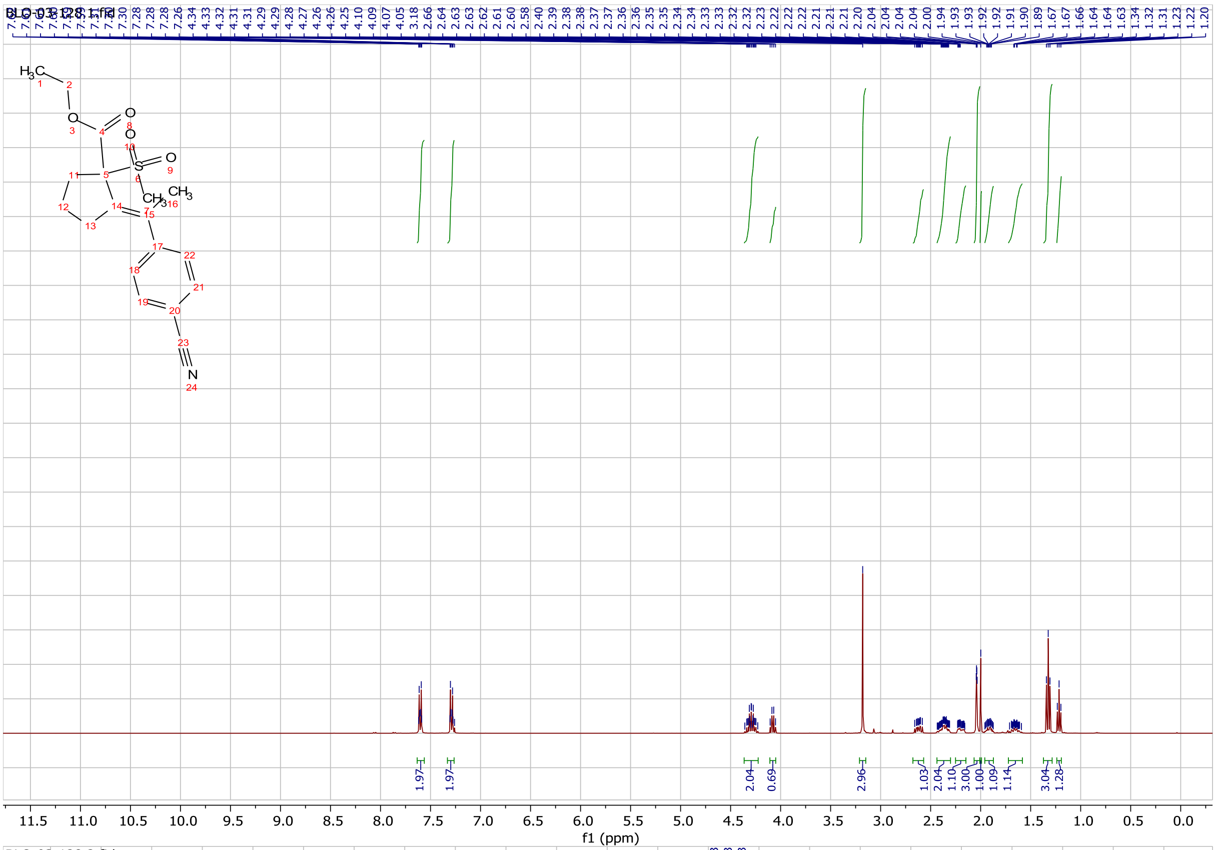

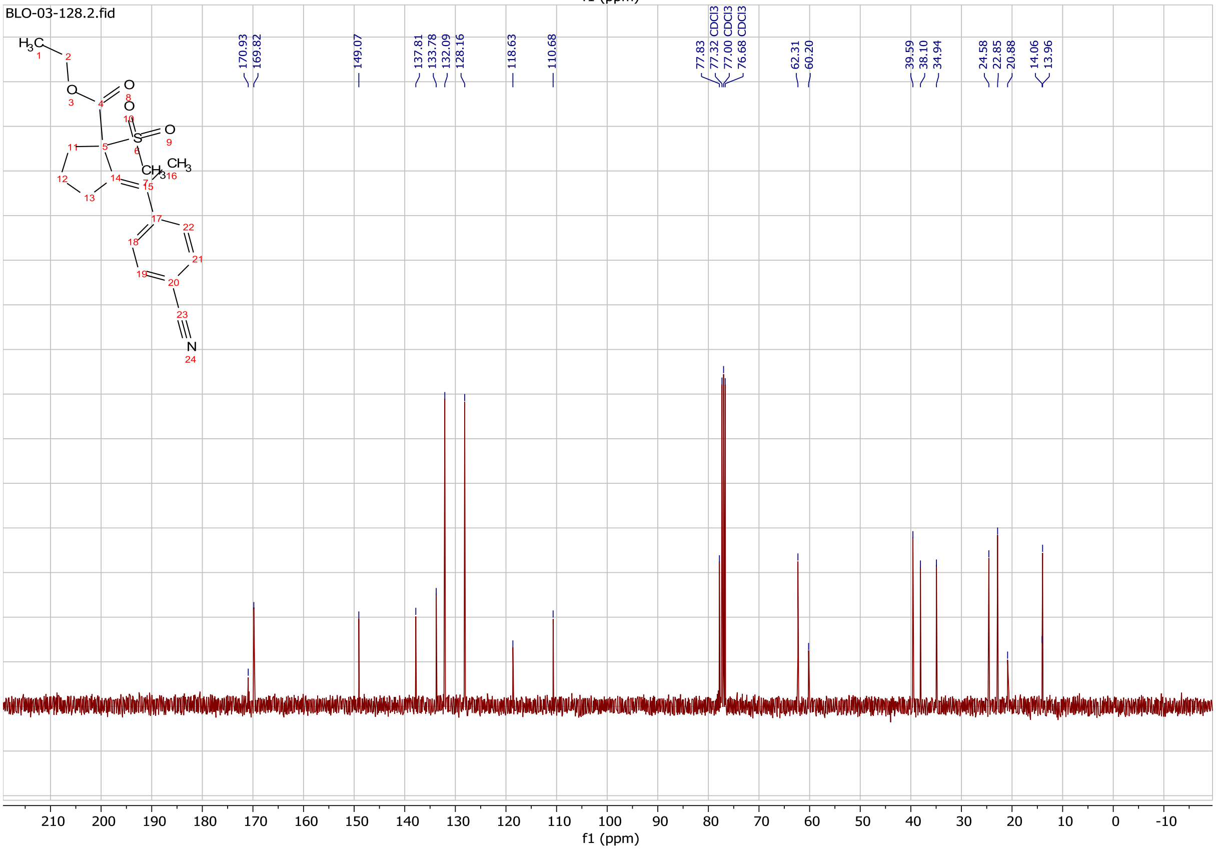

***(E)*-1,1'-(2-(1-phenylethylidene)cyclopentane-1,1-diyl)bis(ethan-1-one) (32a)**

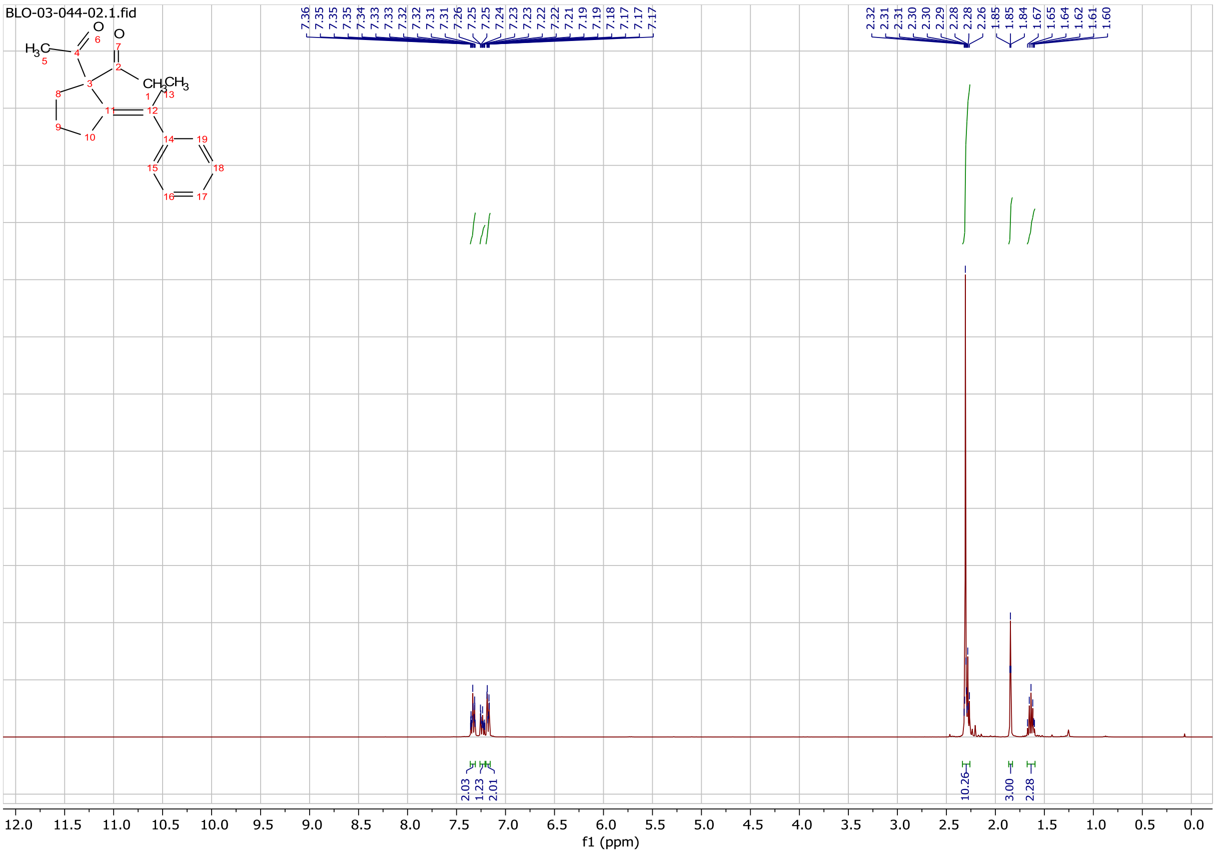

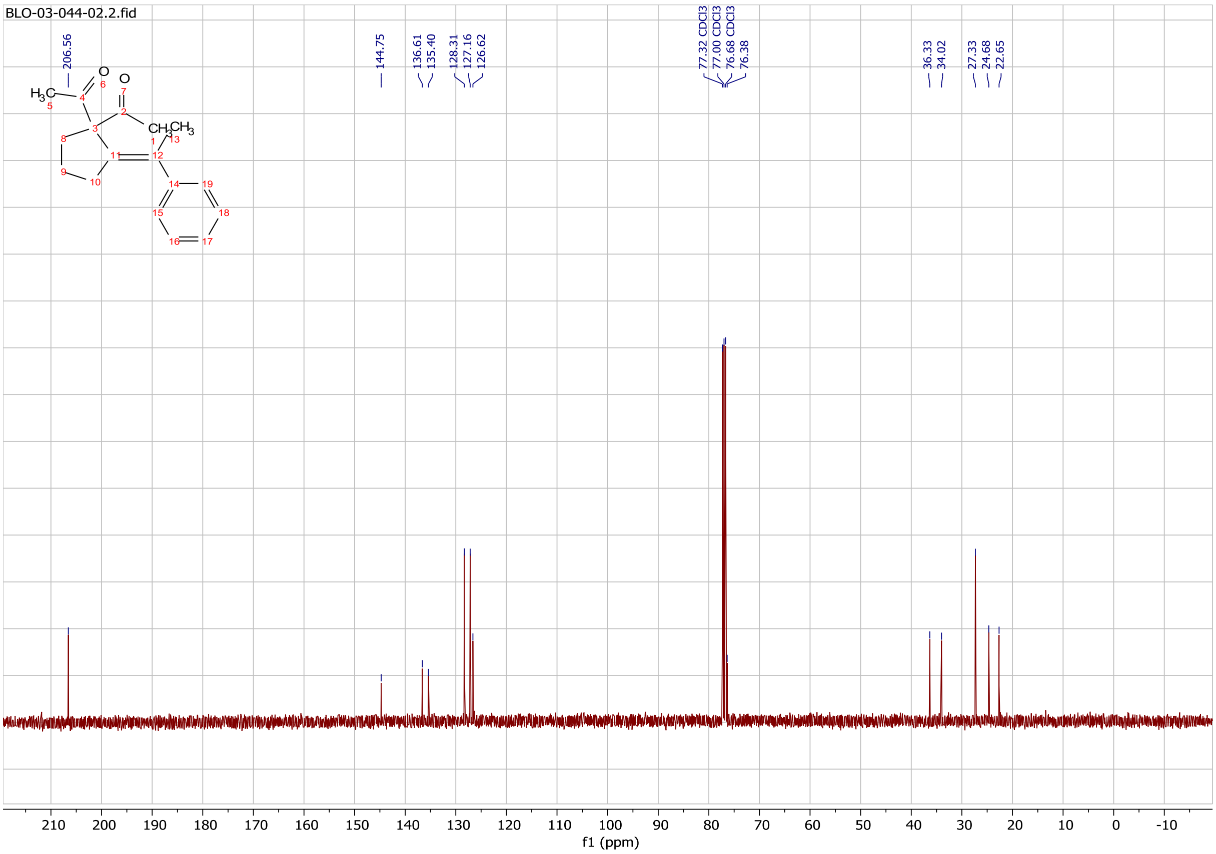

(E)-1,1'-(2-(1-(4-methoxyphenyl)ethylidene)cyclopentane-1,1-diyl)bis(ethan-1-one) (32b)

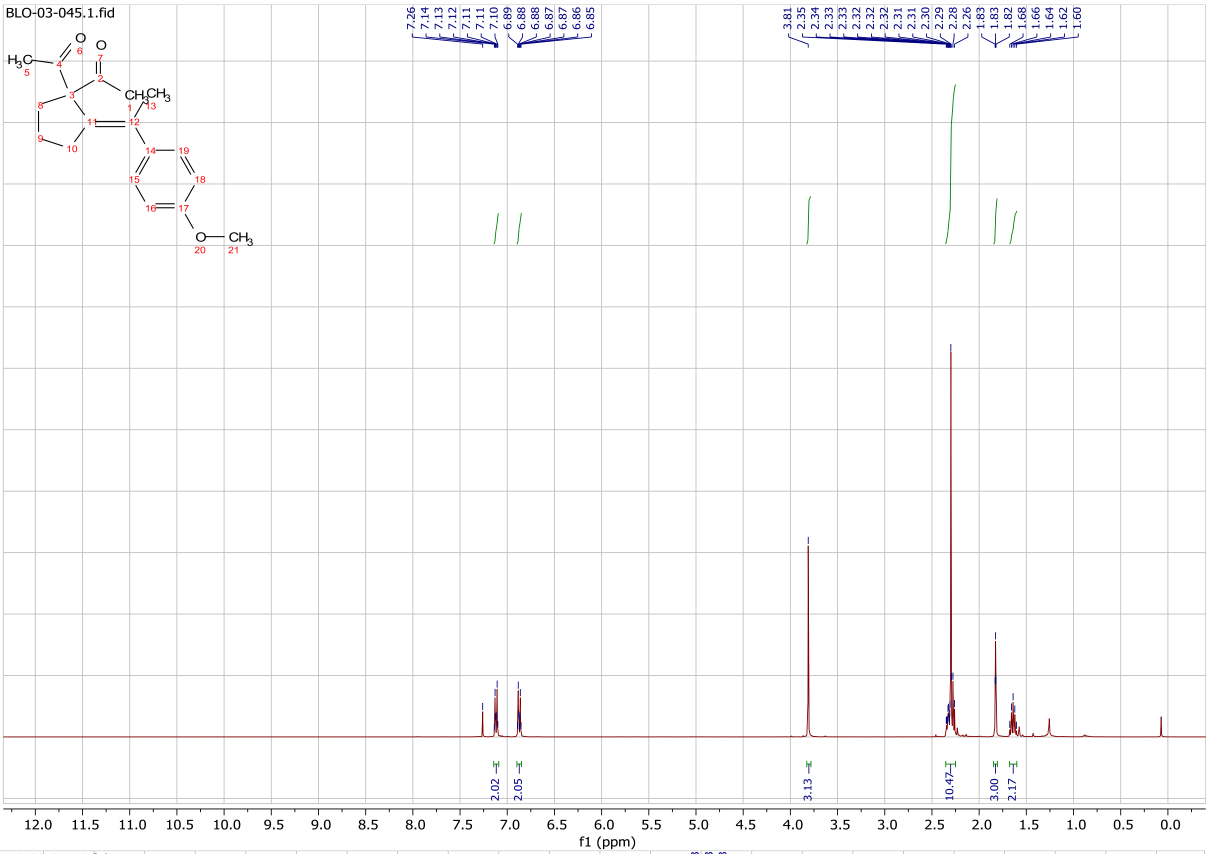

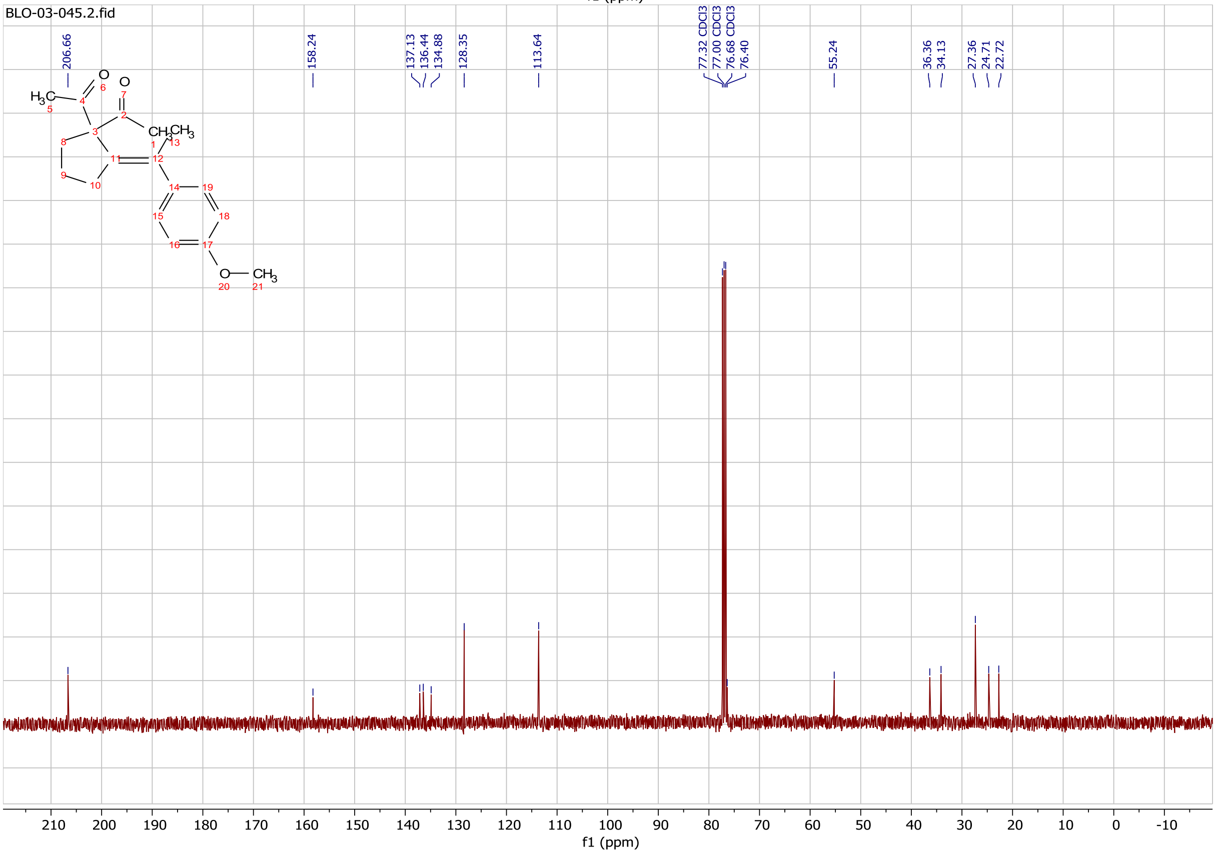

(*E*)-4-(1-(2,2-diacetylcyclopentylidene)ethyl)benzonitrile (32c)

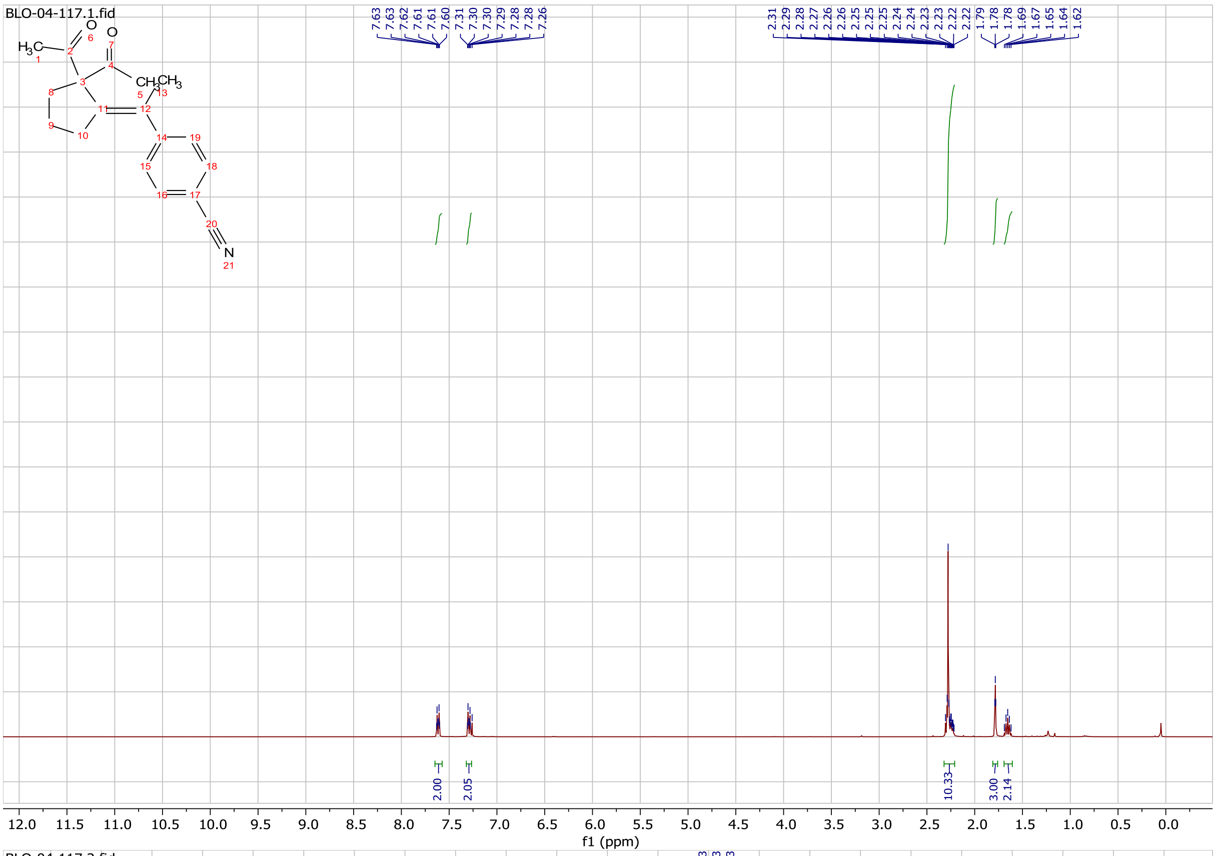

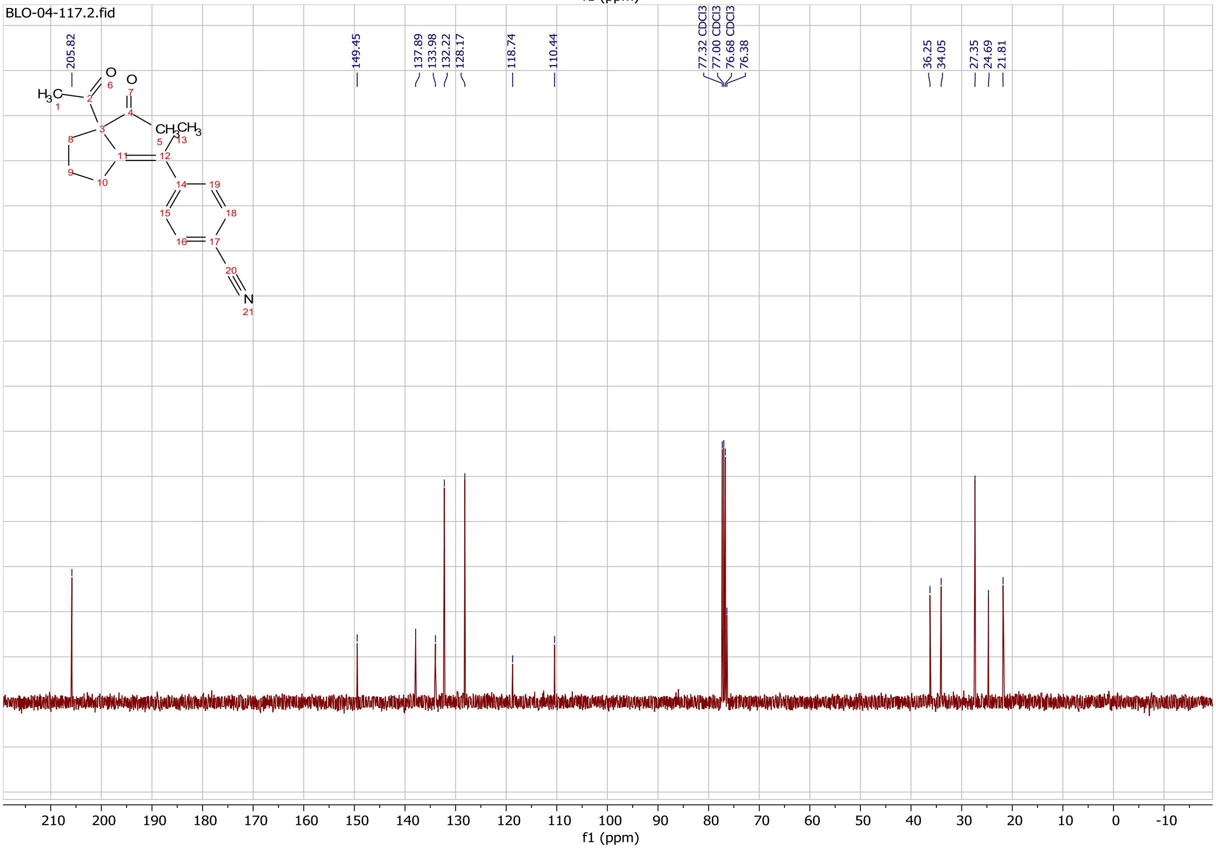

Dimethyl *(E)*-2-(1-phenylpropylidene)cyclopentane-1,1-dicarboxylate (33a)

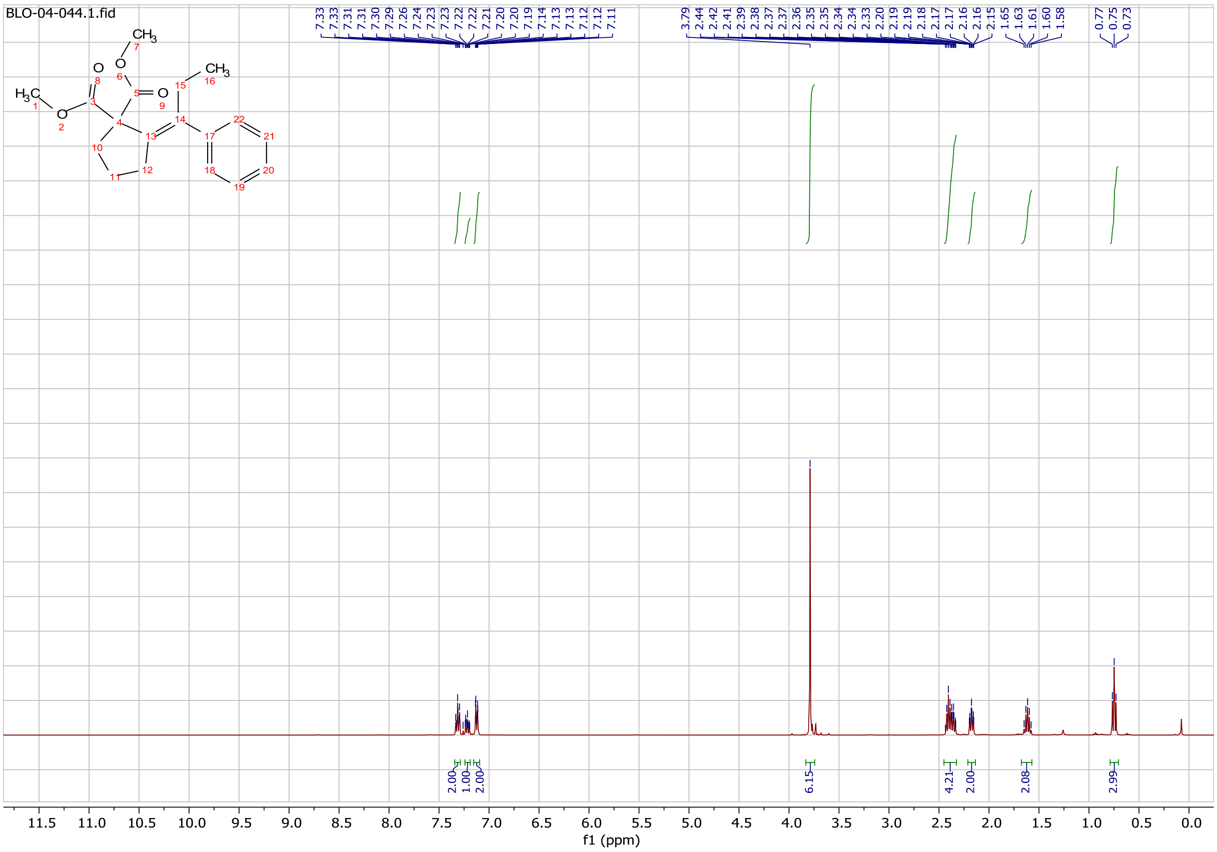

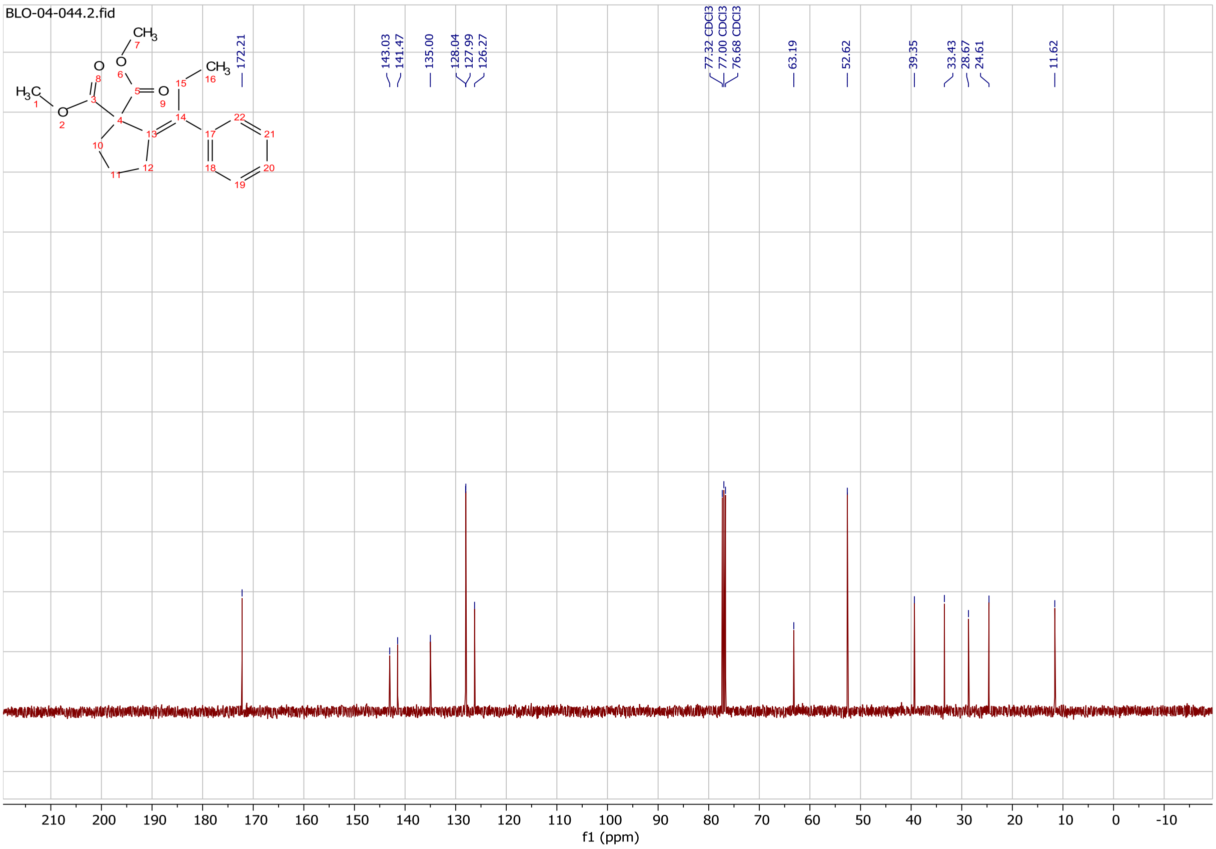

Dimethyl(*E*)-2-(1-(4-methoxyphenyl)propylidene)cyclopentane-1,1-dicarboxylate (33b)

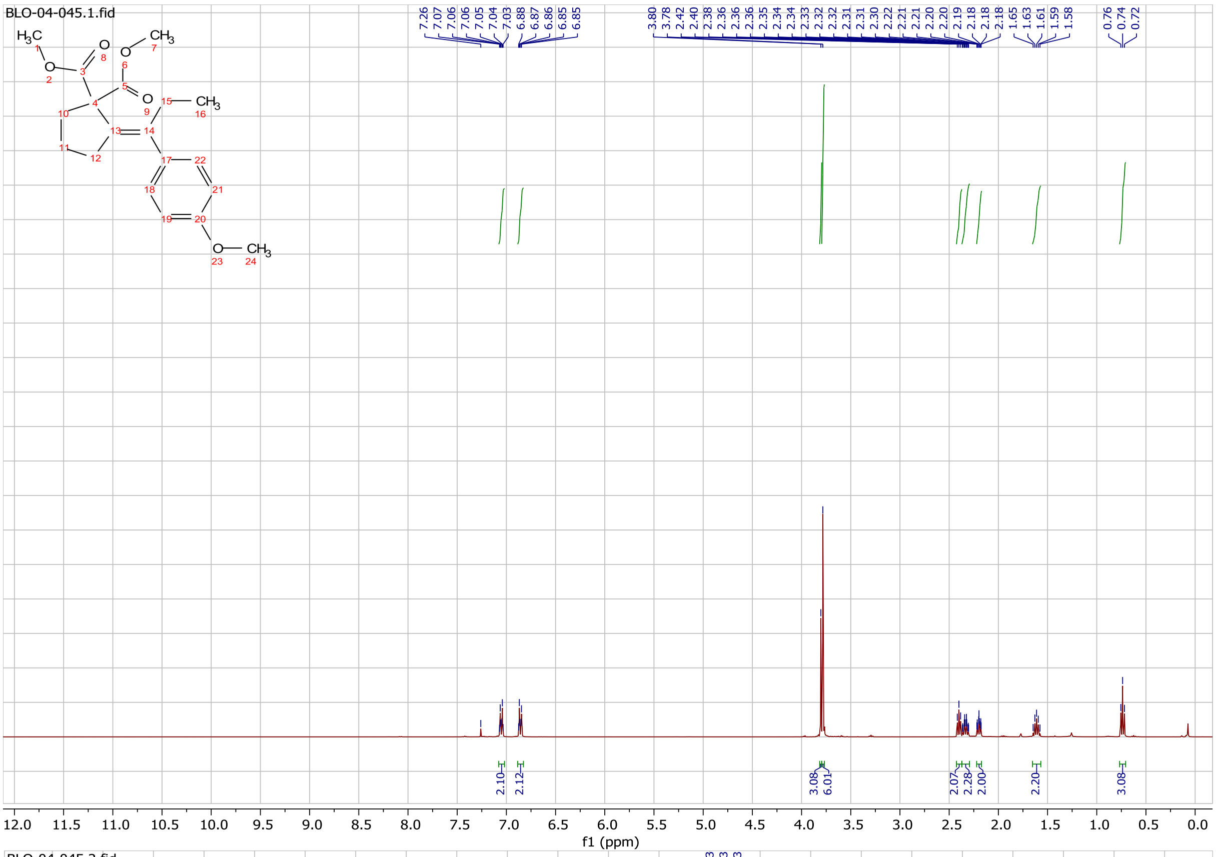

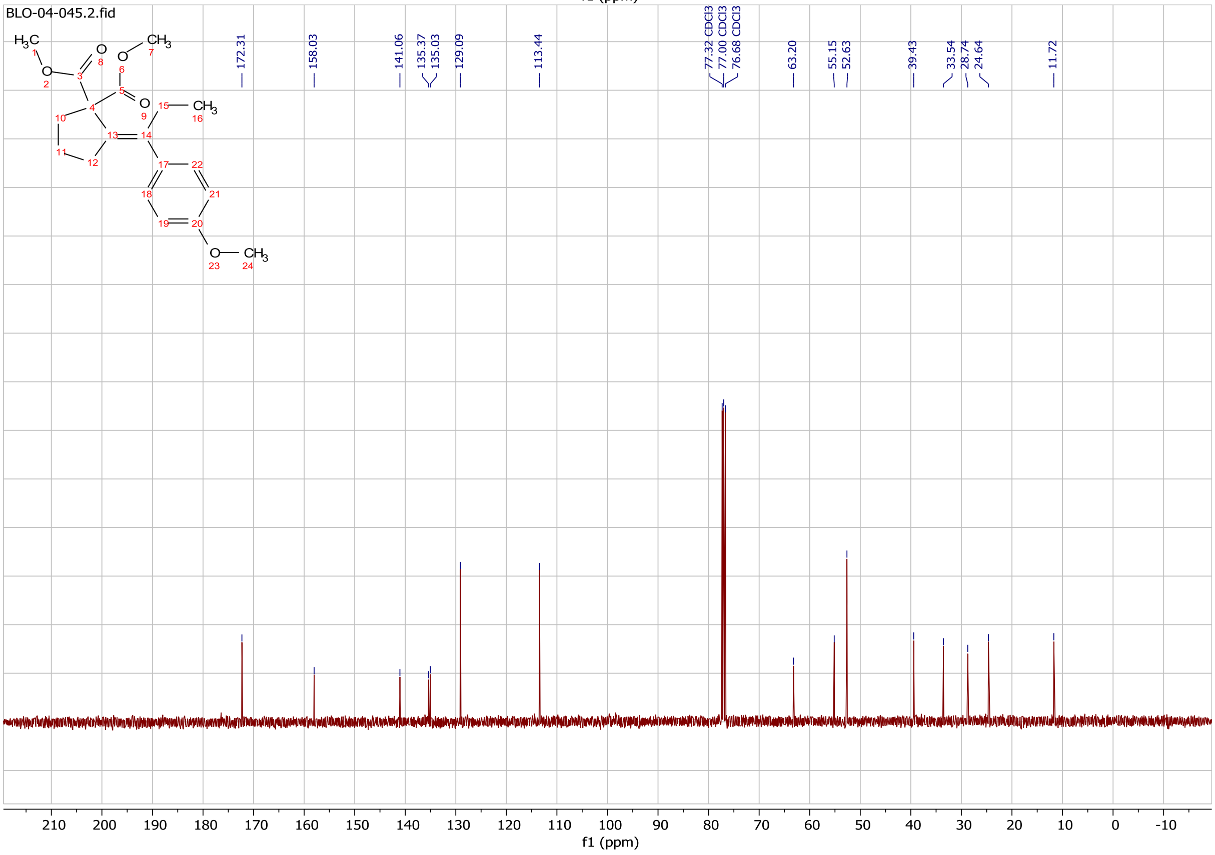

Dimethyl *(E)*-2-(1-(4-cyanophenyl)propylidene)cyclopentane-1,1-dicarboxylate (33c)

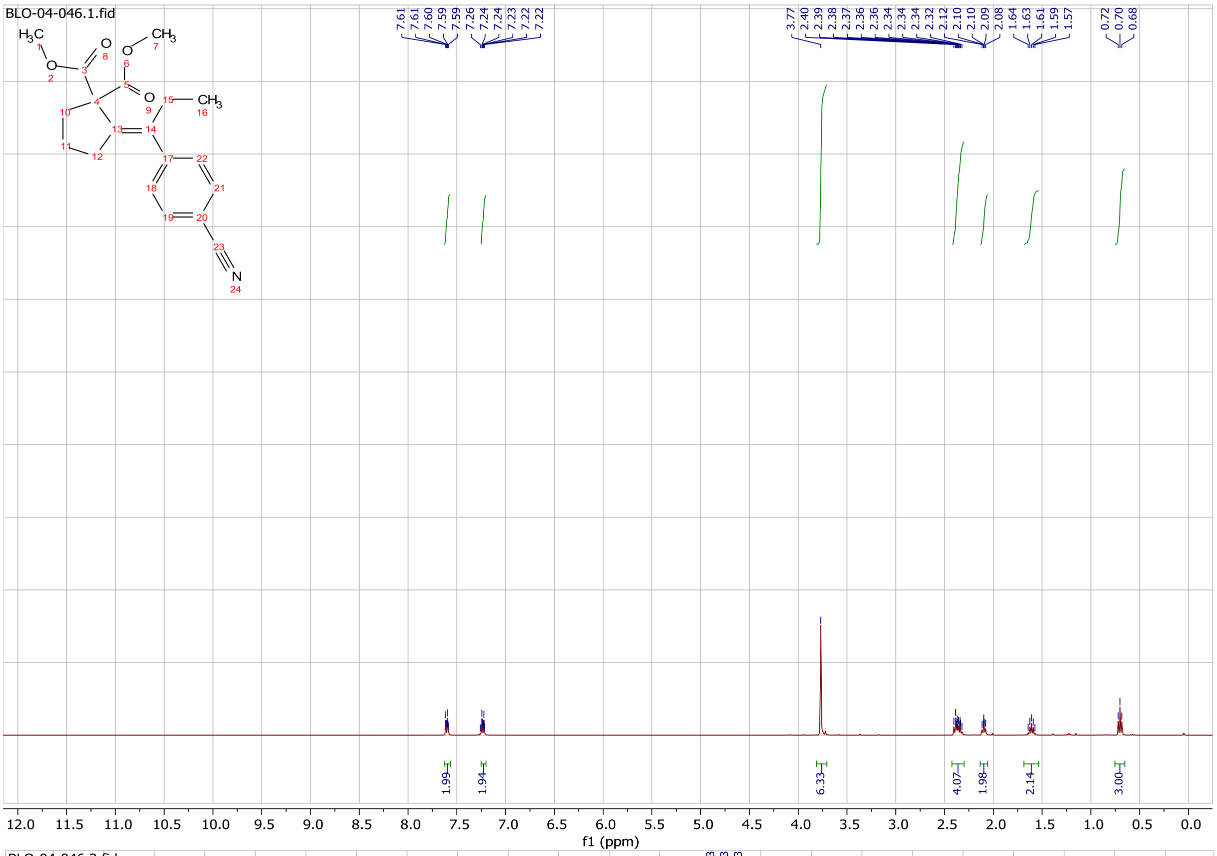

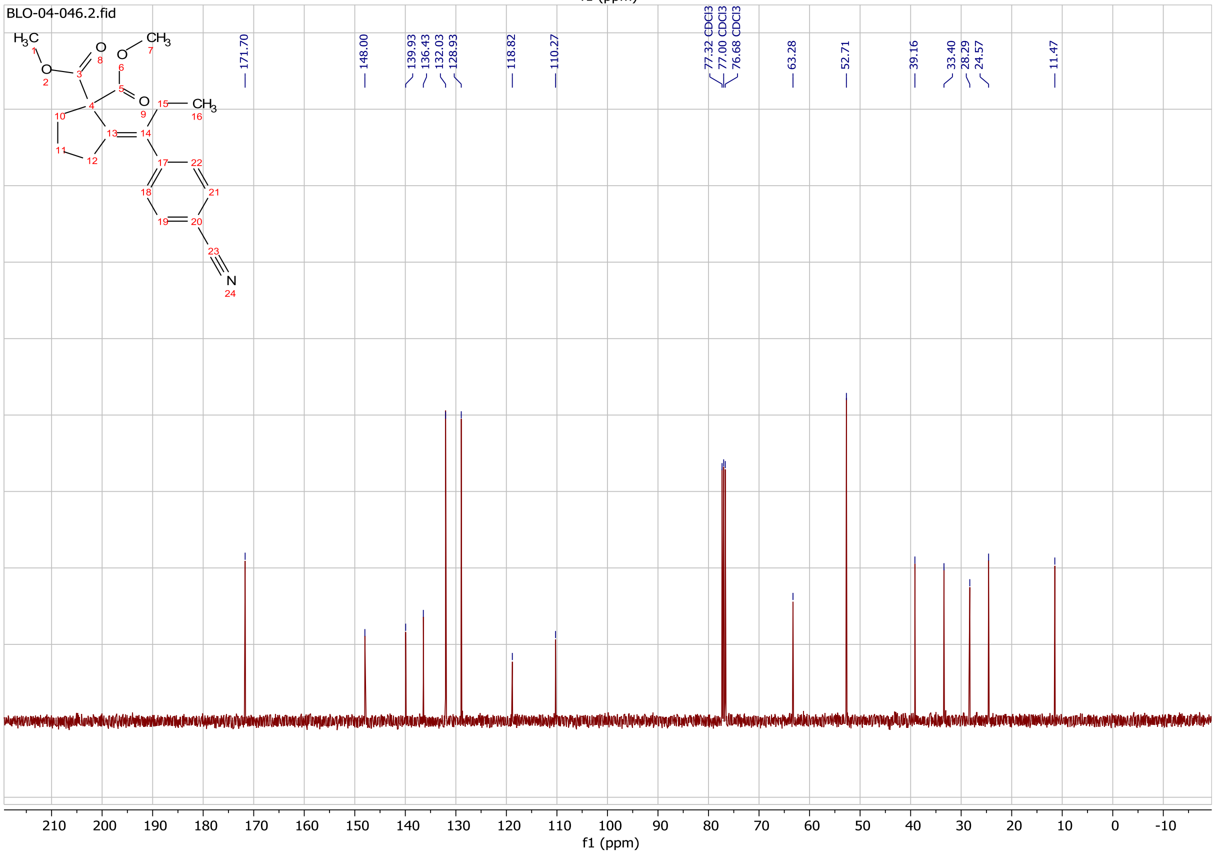

Dimethyl 2-(diphenylmethylene)cyclopentane-1,1-dicarboxylate (34a)

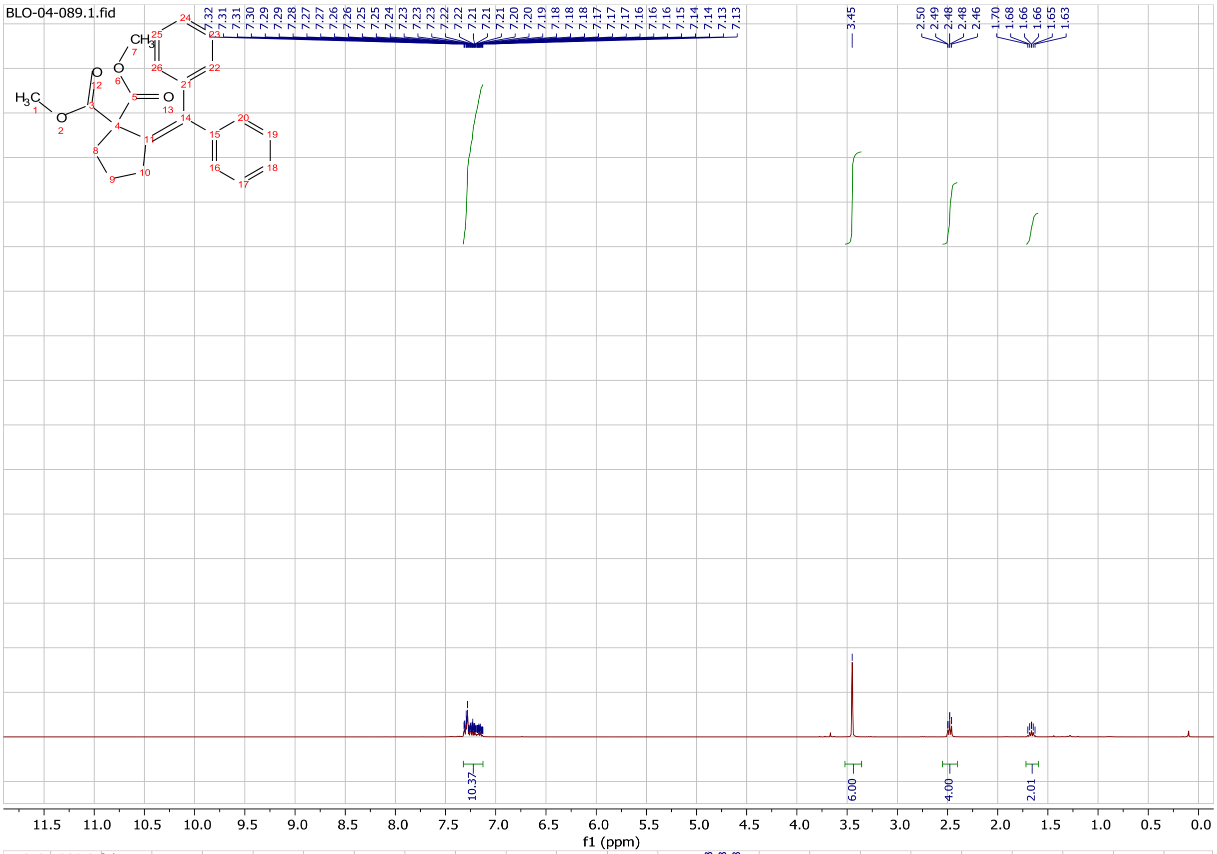

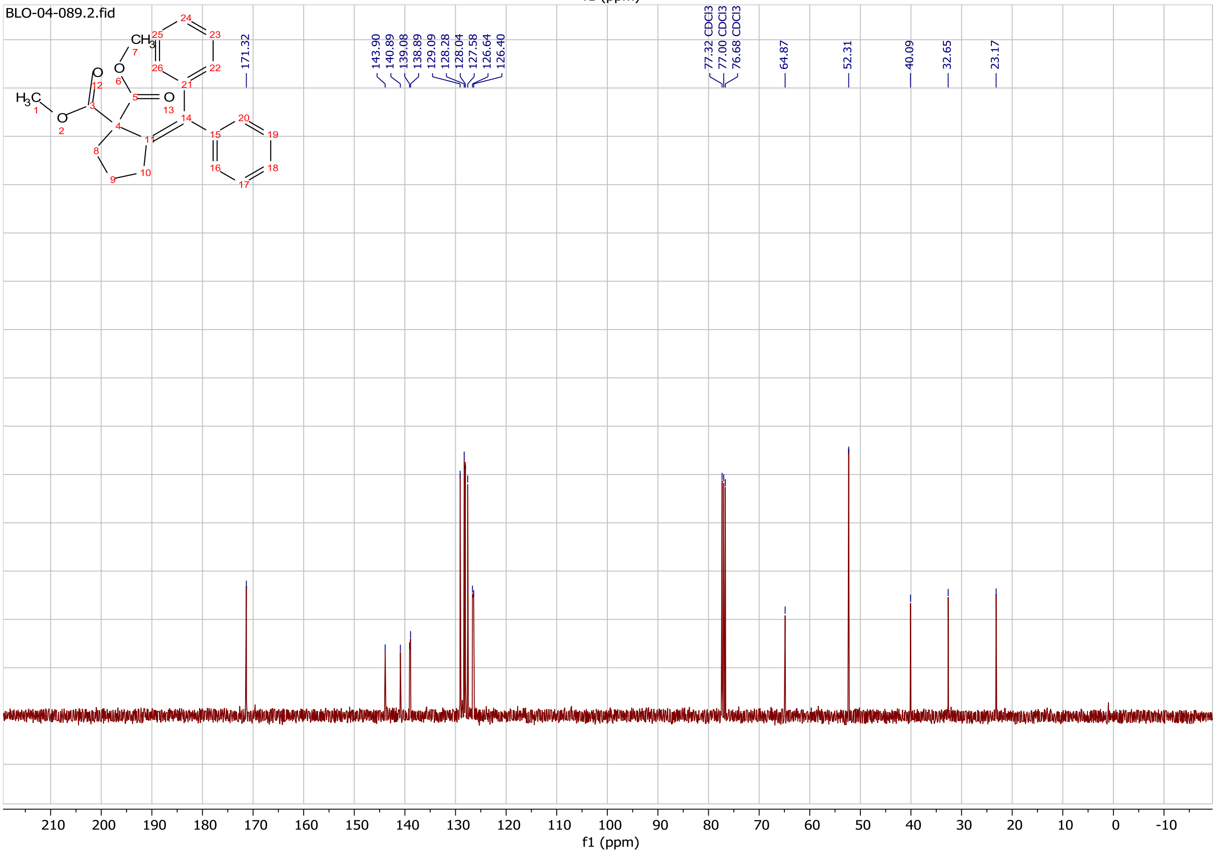

Dimethyl *(E)*-2-((4-methoxyphenyl)(phenyl)methylene)cyclopentane-1,1-dicarboxylate (34b)

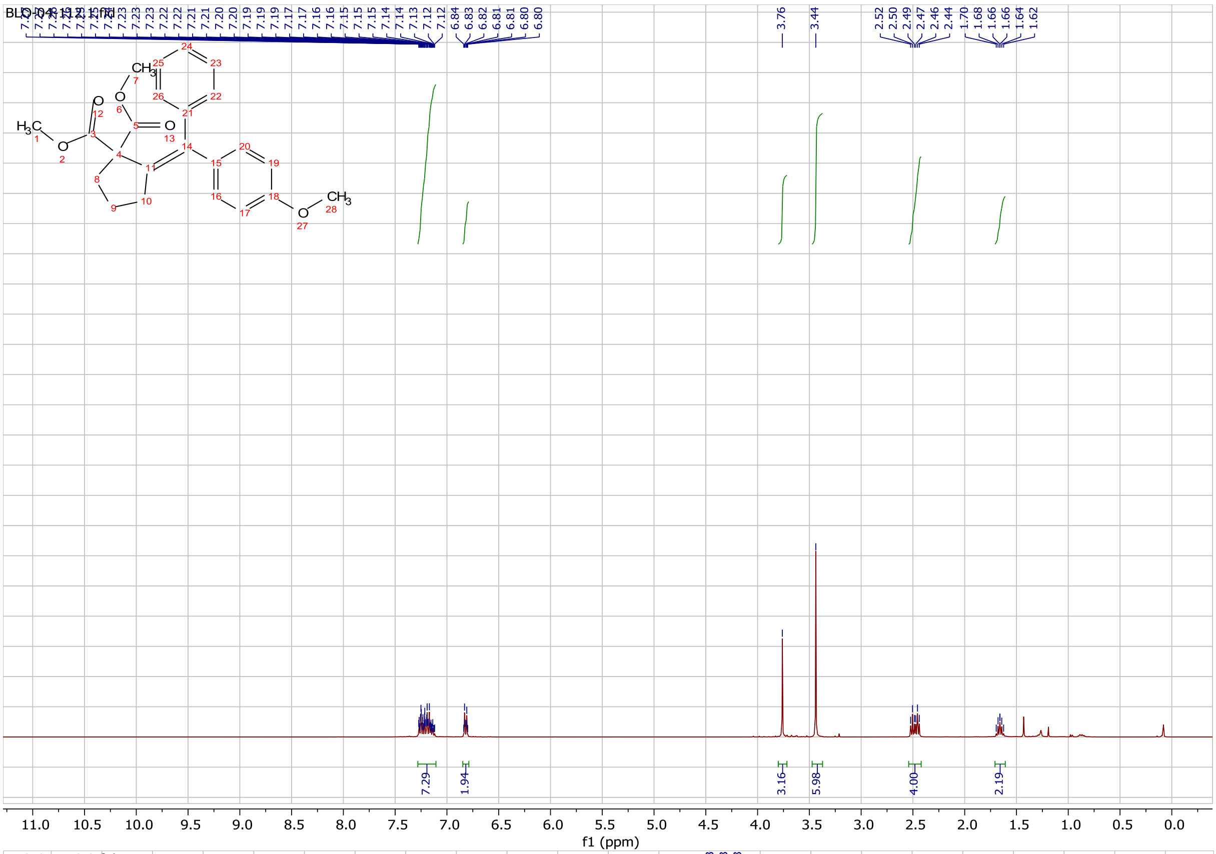

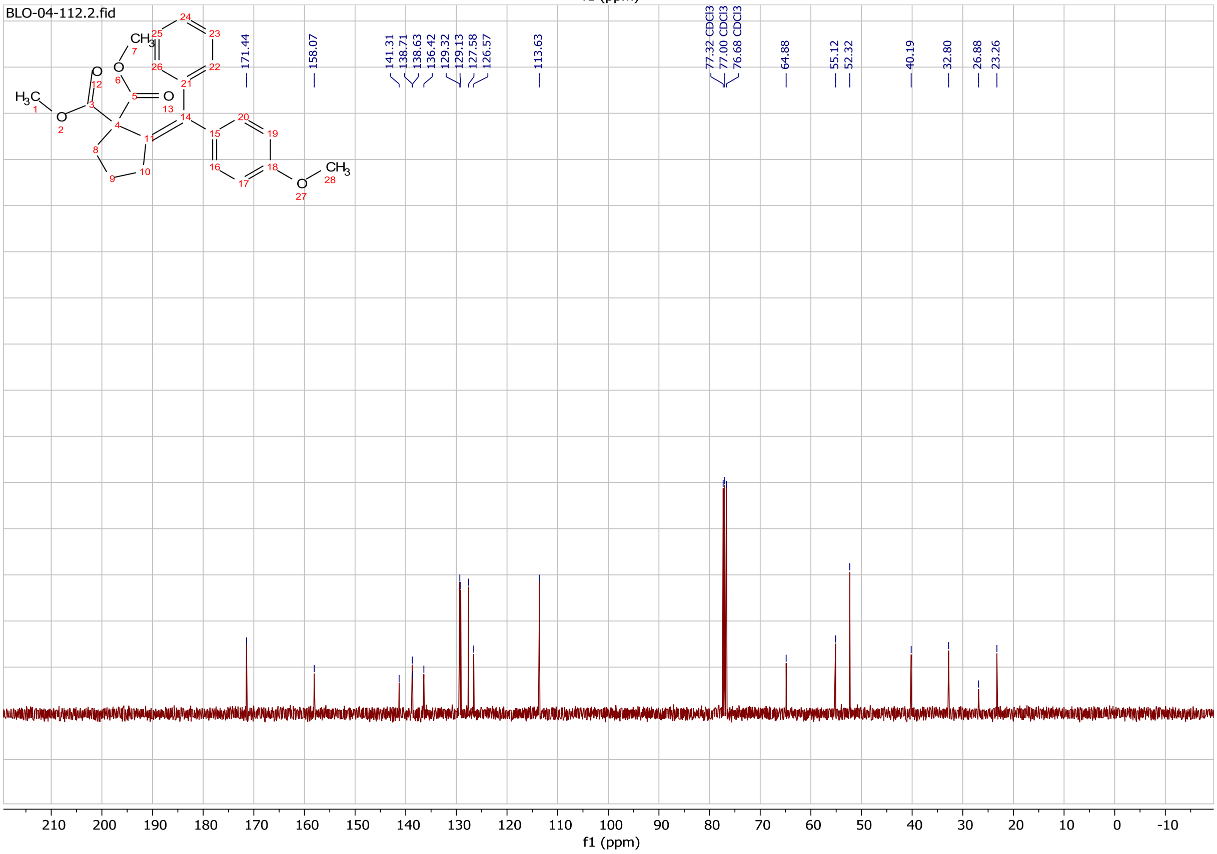

Dimethyl*(E)*-2-((4-cyanophenyl)(phenyl)methylene)cyclopentane-1,1-dicarboxylate (34c)

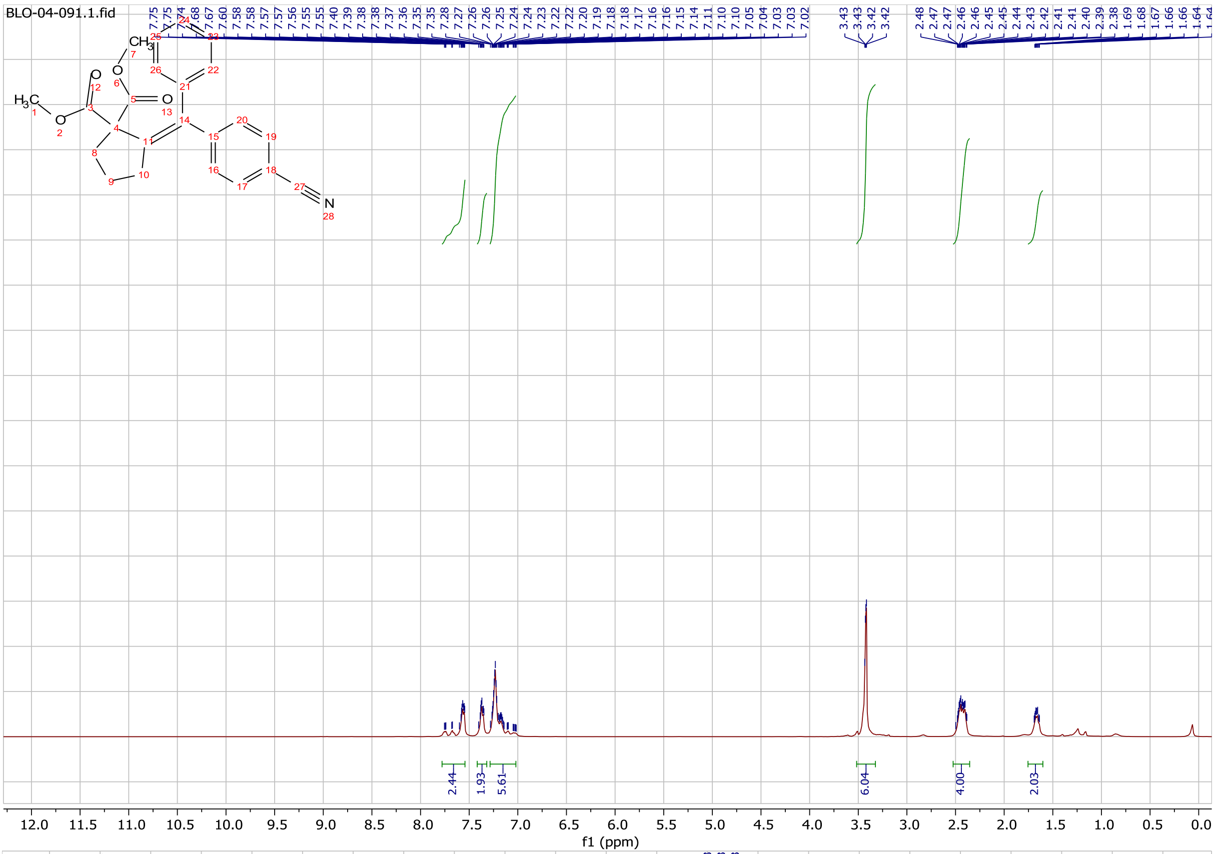

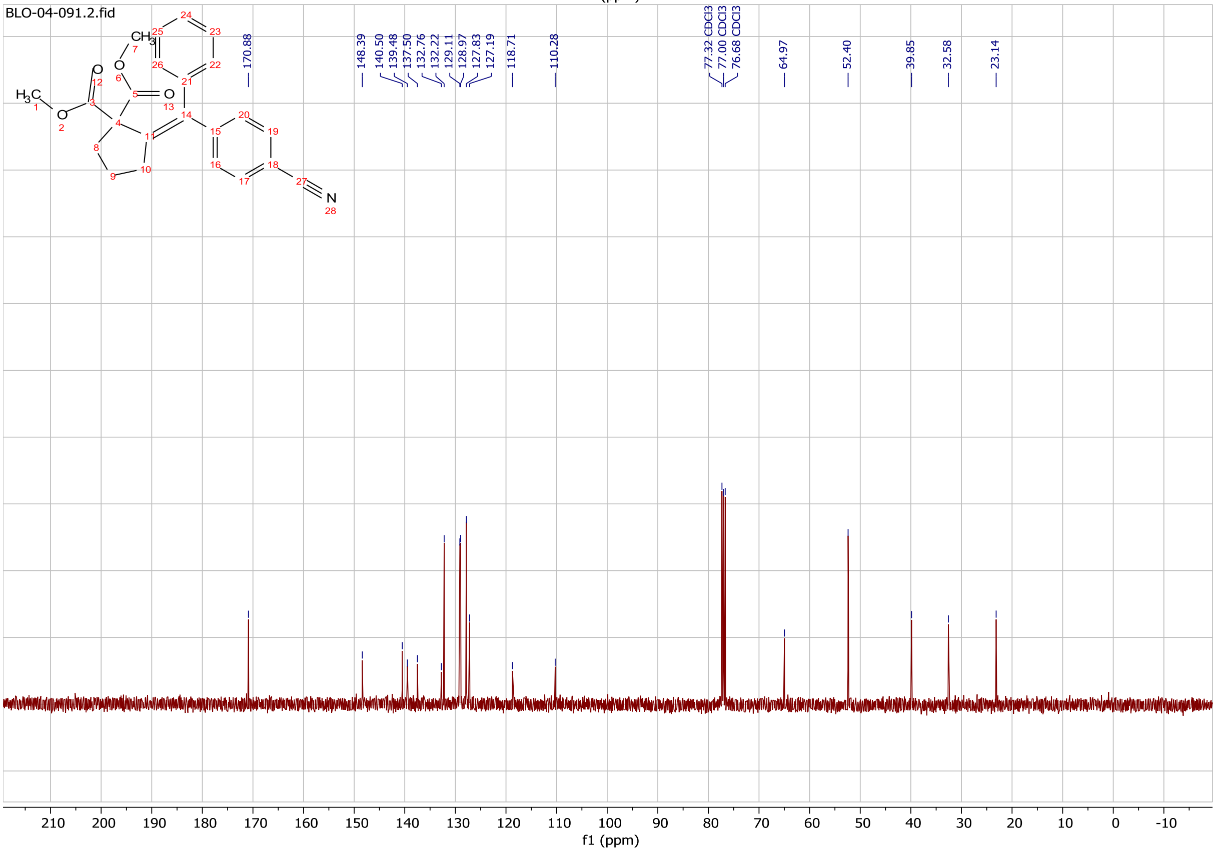

References

[1] Bruno, N. C.; Tudge, M. T.; Buchwald, S. L. *Chem. Sci.*, **2013**, *4*, 916-920.
